# Transcriptome analysis and identification of key genes involved in 1-deoxynojirimycin biosynthesis of mulberry (*Morus alba* L.)

**DOI:** 10.7717/peerj.5443

**Published:** 2018-08-23

**Authors:** Dujun Wang, Li Zhao, Dan Wang, Jia Liu, Xiaofeng Yu, Yuan Wei, Zhen Ouyang

**Affiliations:** 1 School of Food and Biological Engineering, Jiangsu University, Zhenjiang, China; 2 College of Oceanology and Bioengineering, Yancheng Institute of Technology, Yancheng, China; 3 School of Pharmacy, Jiangsu University, Zhenjiang, China

**Keywords:** Mulberry, Transcriptomic, 1-deoxynojirimycin, Biosynthesis, Key enzyme gene analysis

## Abstract

Mulberry (*Morus alba* L.) represents one of the most commonly utilized plants in traditional medicine and as a nutritional plant used worldwide. The polyhydroxylated alkaloid 1-deoxynojirimycin (DNJ) is the major bioactive compounds of mulberry in treating diabetes. However, the DNJ content in mulberry is very low. Therefore, identification of key genes involved in DNJ alkaloid biosynthesis will provide a basis for the further analysis of its biosynthetic pathway and ultimately for the realization of synthetic biological production. Here, two cDNA libraries of mulberry leaf samples with different DNJ contents were constructed. Approximately 16 Gb raw RNA-Seq data was generated and de novo assembled into 112,481 transcripts, with an average length of 766 bp and an N50 value of 1,392. Subsequently, all unigenes were annotated based on nine public databases; 11,318 transcripts were found to be significantly differentially regulated. A total of 38 unique candidate genes were identified as being involved in DNJ alkaloid biosynthesis in mulberry, and nine unique genes had significantly different expression. Three key transcripts of DNJ biosynthesis were identified and further characterized using RT-PCR; they were assigned to lysine decarboxylase and primary-amine oxidase genes. Five CYP450 transcripts and two methyltransferase transcripts were significantly associated with DNJ content. Overall, the biosynthetic pathway of DNJ alkaloid was preliminarily speculated.

## Introduction

Mulberry (*Morus alba* L.), a perennial shrub belonging to the family Moraceae, represents an extremely important economic plant given that its foliage is used in sericulture as the sole diet for the monophagous silkworm (*Bombyx mori*) (Wang et al., 2017). It also has long been used in traditional Chinese medicine to treat disease (Zhishen, Mengcheng & Jianming, 1999). Because of its high nutritive value, the rate of growth, and adaptability, mulberry is also used for other purposes including as a nutritional fruit and food (Srivastava et al., 2003). Mulberry produces multiple secondary metabolites, of which the major bioactive constituents comprise alkaloids, flavonoids, and terpenoids. Each compound has different pharmacological effects including anti-diabetic (Konno et al., 2006), anti-hyperlipidemia (Liu et al., 2009), anti-inflammatory (Park et al., 2013), anti-viral (Lee et al., 2014). Mulberry leaves, as the traditional medicine collected after the frost, were used to treat colds, coughs and other diseases, and these bioactivities may be related to flavonoids ingredients (Wang et al., 2017; Zhang et al., 2015). In particular, the mulberry polyhydroxylated alkaloid 1-deoxynojirimycin (DNJ)—the major compound facilitating the reduction of blood glucose (Li et al., 2011)—is a potent α-glucosidase inhibitor that promotes insulin secretion and glycogenolysis inhibition, among other effects (Kimura et al., 2007). DNJ also counters HIV infection by inhibiting viral glycoprotein production, thus blocking HIV-1–induced formation of syncytial cells (Pollock et al., 2008). Owing to its extensive pharmacological effects, DNJ has garnered the attention of scholars worldwide.

Researchers isolated the DNJ from *Mori Corex*, but the content was very low (Yagi et al., 1976). Kim et al., (1999) isolated and identified DNJ from *Commelina communis* L., but its content was only 0.01125%. Asano et al. (1998) isolated DNJ from the methanol extract of bulbs of hyacinths, and its content was 0.0012%. Ezure et al. (1985) isolated *Streptomyces lavendulae* GC-148 from the soil and isolated DNJ in the culture filtrate, the DNJ content was 4,200 μg/mL. Therefore, the natural source of DNJ was very low, making it very difficult to obtain a large quantity of natural DNJ by traditional extraction methods. At present, DNJ and its derivatives were mainly obtained by chemical synthesis and the combination of chemical synthesis and microbial transformation (Song & Hollingsworth, 2007; Danieli, Lalot & Murphy, 2007; Comins & Fulp, 2001). However, some isomers formed during the preparation process making the separation more difficult, low conversion efficiency and the resultant pharmacological activities were different from those of natural DNJ. Therefore, identification of key genes involved in DNJ alkaloid biosynthesis will provide a basis for the further analysis of its biosynthetic pathway and ultimately for the realization of synthetic biological production. Currently, the key genes involved in DNJ biosynthesis in mulberry leaves have not been identified, as most studies were geared towards DNJ bioactivities, total content, and effective separation and purification methods (Iwashina, 2003; Ouyang & Chen, 2004; Yan et al., 2013).

1-Deoxynojirimycin belongs to the group of piperidine ring alkaloids derived from lysine (Robinson, 1917) and are d-glucose analogs with an NH group substituted for the oxygen atom of the pyranose ring. The two main biosynthesis pathways of lysine, involving more than 32 enzymes, were identified using the Kyoto Encyclopedia of Genes and Genomes (KEGG) database. The diaminopimelic acid (DAP) pathway, which is utilized by most bacteria and green plants to produce lysine (Bhattacharjee, 1985), begins with l-aspartate as the reaction substrate, whereas the (α-aminoadipate (AAA) pathway, with (α-ketoglutarate as the initiator, is preferentially used by fungi, euglena, and some bacteria (Kosuge & Hoshino, 1998). Certain key enzymes in lysine biosynthesis have been reported previously, such as aspartate kinase in the DAP pathway (Vauterin, Frankard & Jacobs, 1999) and homocitrate synthase, homoaconitate hydratase, and saccharopine dehydrogenase in the AAA pathway (Bhattacharjee, 1985). Only two enzymes have been identified in a single pathway involved in the biosynthesis of piperidine rings: lysine decarboxylase (LDC) (Gale & Epps, 1944) and primary-amine oxidase (AOC) (Wilce et al., 1998). In this pathway, lysine is first converted to cadaverine through the catalytic effect of LDC, followed by the AOC-catalyzed formation of a piperidine ring structure and subsequent multi-step reactions to produce the corresponding products.

**Figure 1:**
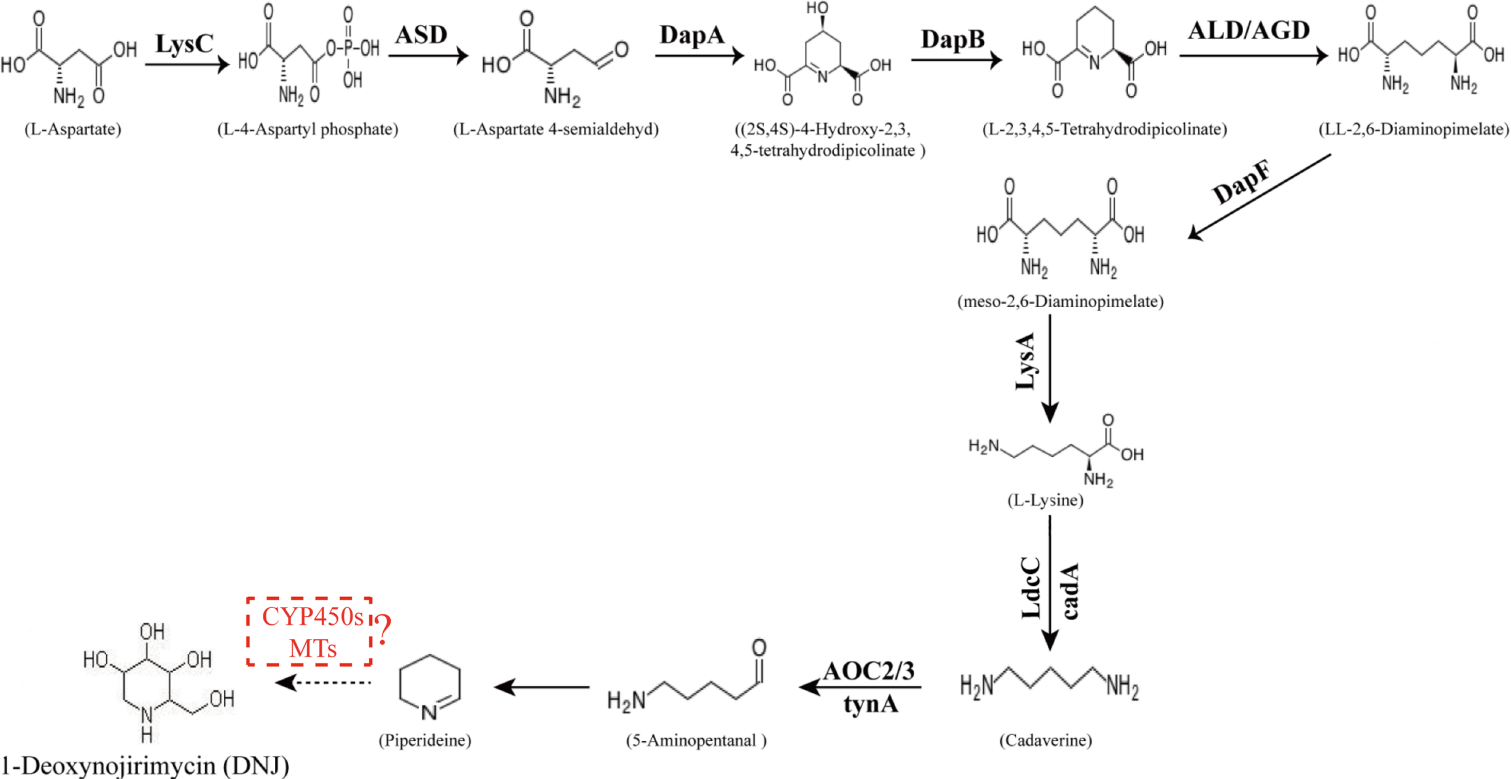
Proposed pathway for the biosynthesis of DNJ alkaloids in mulberry (*Morus alba* L.). LysC, aspartate kinase; ASD, aspartate-semialdehyde dehydrogenase; DapA, 4-hydroxy-tetrahydrodipicolinate synthase; DapB, 4-hydroxy-tetrahydrodipicolinate reductase; ALD/AGD, LL-diaminopimelate aminotransferase; DapF, diaminopimelate epimerase; LysA, diaminopimelate decarboxylase; LdcC/cadA, lysine decarboxylase; AOC2/3, primary-amine oxidase; CYP 450s, cytochrome P450s; MTs, methyltransferases.

The conversion of the piperidine ring to DNJ in the biosynthetic pathway is still unclear. DNJ has a piperidine ring structure with a hydroxymethyl group at position 2 and hydroxyl groups at positions 3, 4, and 5. Thus, DNJ is possibly formed through the methylation and hydroxylation of the 1-piperidine structure by methyltransferases and cytochrome P450 (CYP450) enzymes. CYP450 enzymes exist in plant cells in both soluble and membrane-bound forms and are widely involved in plant secondary metabolic reactions, including hydroxylation, alkylation, and alkenyl epoxides, hydrocarbon oxidation, and dealkylation of nitrogen, sulfur, oxygen sites, and hydroxylation and oxidation of nitrogen sites. Studies have shown that CYP72A224, CYP76B6, and CYP71BJ1 can catalyze the hydroxylation of 19 methylene and monoterpene bases at position 6 of 7-deoxyglucuronic acid cyclopentane, glycyrrhizin, and locaine, respectively (Salim et al., 2013). CYP450 enzymes were found to exhibit hydroxylation activity in *Sphingomonas* sp. HXN-200, and it catalyzes *N*-substituted pyrrolidine, piperidine, azetidine, 2-pyrrolidone, and 2-piperidine. Through hydroxylation of ketones, *N*-substituted azacyclic piperidines and butanes, as well as six-membered heterocyclic compounds, can be catalyzed into the corresponding 4-hydroxy piperidines and 3-hydroxy azetidines (Chang et al., 2002). Methylation reactions are mostly catalyzed by methyltransferases and these reactions are widespread in various species of organisms. For example, in the formation of huperzine A, *N*-methyltransferase catalyzes the transfer of methyl groups on the N atom of huperzine to form huperzine A (Chen & Zhang, 2013). In the synthesis of berberine, *N*-methyltransferase catalyzes the formation of *N*-methylcoclaurine (Morishige et al., 2000). The predicted DNJ biosynthetic pathway in mulberry is shown in Fig. 1.

Our previous study (Ouyang & Chen, 2004) showed that seasonal temperature variation had a significant effect on DNJ content in different growth seasons. Low DNJ content was found between April and May with low temperature; however, the highest DNJ content was found in summer with higher temperature between July and August, and then the DNJ content decreased gradually with temperature after September. Lower DNJ content was found after the frost in October and November. These findings suggested that higher temperature is conducive for DNJ alkaloid synthesis and accumulation. Moreover, some related studies have indicated that temperature is an important factor on alkaloids content in that higher temperature can promote the accumulation of alkaloids (Guo et al., 2007; Iba, 2002). In this study, mulberry leaf samples collected in July, high temperature season (i.e., with the highest DNJ content) and November, lower temperature season (i.e., with the lowest DNJ content) were selected for transcriptome sequencing and subsequent analysis of genes related to DNJ alkaloid biosynthesis.

Recently, the sequencing and analysis of transcriptomes have become primary tools for the identification of genes involved in plant secondary metabolism as well as for the discovery of novel genes in some non-model plants (Asamizu, 2000; Martin & Wang, 2011; Yang et al., 2013). These tools have been previously used in the study of mulberry, with the main focus being on discovering the candidate genes and potential pathways in plants’ responses to cold (Peng et al., 2015) and drought stress (Wang et al., 2014). Furthermore, transcriptome data have been published for *M. atropurpurea* (Dai et al., 2015) and mulberry roots have been analyzed using transcriptome technology, which identified some genes that might be associated with root formation from mulberry stem cuttings (Du et al., 2016). Some genic simple sequence repeat markers from Indian mulberry have also been found (Mathi Thumilan et al., 2016). However, few genes involved in DNJ biosynthesis and accumulation have been identified.

In the current study, Illumina HiSeq 2500 RNA sequencing followed by de novo assembly was used to analyze the transcriptome data of two mulberry leaves samples with significant differences in DNJ contents. Differentially expressed genes (DEGs) were screened, and the correlation analysis between differential gene expression and DNJ content was carried out to identify the key enzyme genes of DNJ alkaloid biosynthesis. The results presented here and those of previous studies would provide a fertile ground toward exploration of the biosynthetic pathway of DNJ in the future.

## Materials and Methods

### Plant materials

Mulberry leaf samples were obtained from a mulberry plantation in Jiangsu University (Zhenjiang, China). Mulberry tender leaves from top of branches of the same tree were collected on July 31, 2016 (M7), November 15, 2016 (M11) for the first time, and on July 25, 2017 (Ma7), November 15, 2017 (Ma11) for the second time—DNJ contents were significantly different between the two samples in each collection time, 2017 samples were collected from a different tree than 2016 samples. The collected samples were immediately frozen in liquid nitrogen, and then stored at −80 °C until transcriptome analysis. Fresh mulberry leaves were collected from April 15, to November 25, from the same location on each branch (from the top 6–10 leaves); the samples were collected every 10 days, and each independent sample was made up with ten mulberry leaves, the leaves were dried for DNJ content determination.

### Determination of DNJ content

1-Deoxynojirimycin was extracted as previously described (Ouyang, Chen & Li, 2005), with minor modifications. Fresh leaves were dried at 60 °C and ground to powder prior to analysis. A total of 200 micrograms of the powder was added to 10 mL aqueous 0.05M HCl, extracted by vortexing for 20 s, and centrifuged at 9,000×*g* for 15 min. The supernatant was collected in a 50 mL volumetric flask, the sediment was extracted a second time, and the final supernatant was added to the volumetric flask and diluted to 50 mL with water. A 10 μL aliquot of the extracting solution was mixed with 10 μL of 0.4M potassium borate buffer (pH 8.5), and 20 μL of 5 mM fluorenylmethyloxycarbonyl chloride (FMOC-Cl) in acetonitrile was added to the mixture and allowed to react at 20 °C for 20 min. Then, 10 μL of 0.1M glycine was added to neutralize the excess FMOC-Cl and 950 μL of 0.1% (v/v) aqueous acetic acid was added to stabilize the reaction compounds. The reactant solution was filtered through a 0.22 μm Millipore filter (Millipore Corp., Billerica, MA, USA), and 10 μL of the filter liquor was used for subsequent analysis by high performance liquid chromatography (HPLC) with a fluorescence detector (FLD). An Agilent 1260 Infinity HPLC system (Agilent Technologies Inc., Palo Alto, CA, USA) with a HIQ Sil C18-10 column (size 4.6 × 250 mm, no. 00V00194; Kya Technologies, Tokyo, Japan), and a 1260 FLD (excitation 254 nm, emission 322 nm). The mobile phase consisted of acetonitrile and 0.1% aqueous acetic acid (1:1, v/v) at one mL/min for 20 min, and the column was maintained at 25 °C. The data were analyzed using an Agilent 1260 Open Lab workstation. All the determinations were performed in triplicate.

### Total RNA extraction, cDNA library construction, and RNA-Seq

Total RNA was extracted using TRIzol reagent (Sangon Biotech, Shanghai, China). RNA quality and quantity were analyzed using 1% agarose gel electrophoresis and a Qubit 2.0 RNA kit (Life Technologies, Carlsbad, CA, USA). Independent total RNA extracts were prepared from multiple leaves (more than three leaves) and equal amounts of RNA from each sample were used to generate RNA-Seq libraries. The library was constructed using the VAHTSTM mRNA-seq v.2 Library Prep kit for Illumina (Vazyme Biotech Co., Nanjing, China). Briefly, one μg total RNA was purified using poly-T oligo-conjugated magnetic beads, and cleaved RNA was used for cDNA synthesis with random primers. The cDNA fragments were purified, the ends blunted, and a poly-A tail added, followed by adapter ligation. Polymerase chain reaction (PCR) amplification of cDNA fragments was carried out using PCR Primer and Amplification Mix (Vazyme Biotech Co., Ltd., Nanjing, China). The reaction conditions were 15 cycles (98 °C for 30 s, 98 °C for 10 s, 60 °C for 30 s, 72 °C for 30 s, 72 °C for 5 min, and holding at 4 °C). Reaction products were purified using a Qubit 2.0 DNA kit, and double-stranded cDNAs were used for HiSeq 2500 paired-end sequencing.

### De novo transcriptome data processing and assembly

A perl script was written to remove vector sequences and PolyA (T) tails from the raw sequence data using cutadapt software (http://pypi.python.org/pypi/cutadapt/1.2.1, version: 1.2.1). Low-quality reads (<20 bases) and those with *N*-portions >35 bp were removed using Prinseq software (http://prinseq.sourceforge.net/, version: 0.19.5). Reads were merged after eliminating repeats. The high-quality reads were assembled using Trinity software (http://trinityrnaseq.github.io/, version: r20140717) to construct unique transcripts (Grabherr et al., 2011).

### Function annotation and classification

Unigenes were compared using the basic local alignment search tool (BLAST) against the National Center for Biotechnology Information non-redundant nucleotide (NT, ftp://ftp.ncbi.nlm.nih.gov/blast/db/nt.*tar.gz, using BlastN) and non-redundant protein (NR, ftp://ftp.ncbi.nlm.nih.gov/blast/db/nr.*tar.gz, using BlastX) databases (January 2013; *E*-value ≤ 1e^−5^). SWISS-PROT (downloaded from the European Bioinformatics Institute on January 2013; *E*-values ≤ 1e^10^, using BlastX) was used for annotation and classification. The Clusters of Orthologous Groups of Proteins database (COG; *E*-values ≤ 1e^10^, using rpsBlast), (KEGG, release 58; *E*-values ≤ 1e^10^), and InterProScan Release 36.0 annotated protein domains and functional assignments were mapped onto Gene Ontology (GO, http://www.geneontology.org/) by using the BlastX algorithm.

### Identification of differentially expressed unigenes

Unigenes that were differentially expressed between the two cDNA libraries were identified by DESeq and edgeR software (http://www.bioconductor.org/) according to a previously reported method (Anders & Huber, 2010). DEGs were considered as genes having differential expression with a *P*-value ≤ 0.01 and an expression ratio of ≥2. DESeq software was used to analyze genes with a *q*-value < 0.001 and fold change >2.

### Quantitative real-time (qRT)-PCR analysis of differentially expressed unigenes

A total of 20 unigenes (Table S1) that were significantly differentially expressed were randomly selected to validate the accuracy of RNA-Seq data using qRT-PCR. Total RNA was reverse transcribed using the RevertAid™ First Strand cDNA Synthesis Kit (Thermo Fisher Scientific, Waltham, MA, USA). The real-time PCR experiments were performed using a Fast Start Essential DNA Green Master kit and a Light Cycle 96 Real-Time PCR system (Roche, Basel, Switzerland). The primers were designed using Primer Premier 5.0 (Premier Biosoft Ltd., Palo Alto, CA, USA) and were obtained from Sangon Biotech (Shanghai, China) (Table S2A; Tables 2B and 2C). The RT-PCR program comprised a reverse transcription step at 48 °C for 30 min and a Taq polymerase activation step at 95 °C for 300 s, followed by PCR: 45 cycles at 95 °C for 15 s, 61 °C for 20 s, and 72 °C for 30 s, followed by a melting cycle. The 2^−ΔΔCt^ (Livak & Schmittgen, 2001) method was used to calculate the relative changes in target gene expression from the qRT-PCR results, using the mulberry β-actin gene as the reference gene. All experiments were carried out in technical and biological triplicate (independent leaves).

### Biological validation of RNA-Seq results

The same strategy was used to perform transcriptome studies on samples (M7, M11) and (Ma7, Ma11). BLASTN was used to compare the coverage of sequences. The number and coverage of DEGs were screened to evaluate the biological repeatability of the experiment.

### Statistical analysis

Data are expressed as the mean ± standard deviation (SD). Data were evaluated for statistically significant difference by using Student’s *t*-test. SPSS 13.0 (IBM SPSS, Armonk, NY, USA) was used for analysis, and *P* < 0.05 was considered statistically significant. Correlation analysis was conducted using GraphPad Prism 5.0 software (San Diego, CA).

## Results

### DNJ content in mulberry samples

**Figure 2:**
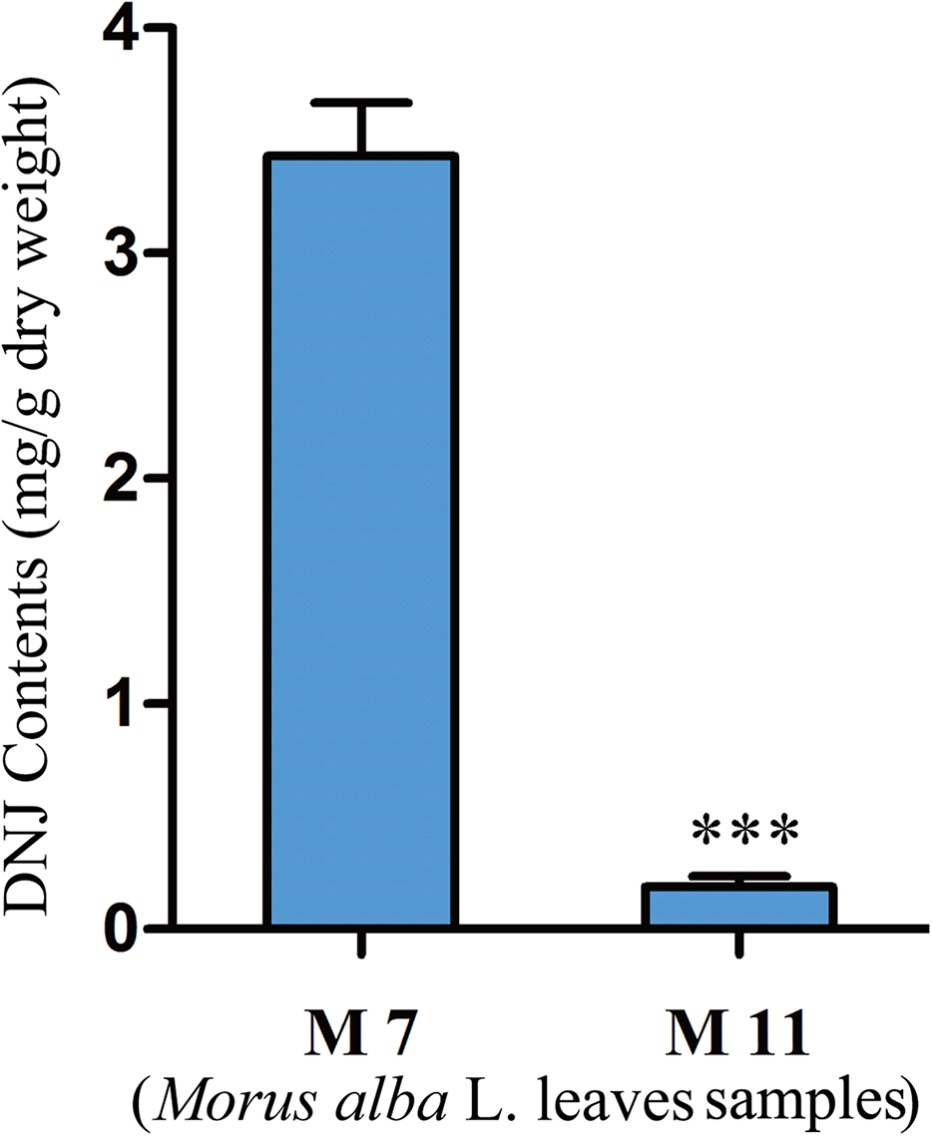
DNJ content in *Morus alba* L. leaves collected on July 31, 2016 (M7) and November 15, 2016 (M11). Error bars represent standard deviation. ***, Student *t*-test significant at *P* < 0.001.

To investigate the relationship between DNJ content and gene expression, the DNJ contents of different developmental staged mulberry plants (*M. alba* L.) were determined by HPLC-FLD. The DNJ content in M7 was 3.43 ± 0.15 mg/g dry weight, as compared to 0.19 ± 0.05 mg/g dry weight in M11 (*P* < 0.001; Fig. 2).

### Mulberry transcriptome sequencing and de novo assembly

As shown in Table 1, mulberry leaf samples of two different stages of development were sequenced using the Illumina Hiseq2500 system, which generated 105,189,326 and 65,166,690 raw reads for M7 and M11, respectively. The average length of a single read was 150 bp; after filtering out low-quality reads, 104,988,228 and 64,878,089 reads (99.81% and 99.56% of raw sequences, respectively) were obtained for M7 and M11, respectively, with Q20 values of 97.52% and 94.42%, respectively. High-quality reads were assembled from 112,481 unique transcripts with a mean length of 766 bp and an N50 value of 1,392. Transcript lengths ranged from 201–18,301 bp (Fig. S1).

**Table 1:** Summary of RNA-Seq data of *Morus alba* L. leaves.

Library	No. of reads	Single length (bp)	Paired-end? (Y/N)	Total length (bp)	Q 20		High-quality reads*		
					Length (bp)	%	Number	Length (bp)	%
M7	105,189,326	150	Y	15,778,398,900	15,386,817,957	97.52	104,988,228	15,283,135,888	99.81
M11	65,166,690	150	Y	9,775,003,500	9,229,450,858	94.42	64,878,089	9,248,580,466	99.56

**Note:**

* High-quality ≥20 bp; Length cutoff ≥35 bp.

### Functional annotation of the mulberry transcriptome

**Figure 3:**
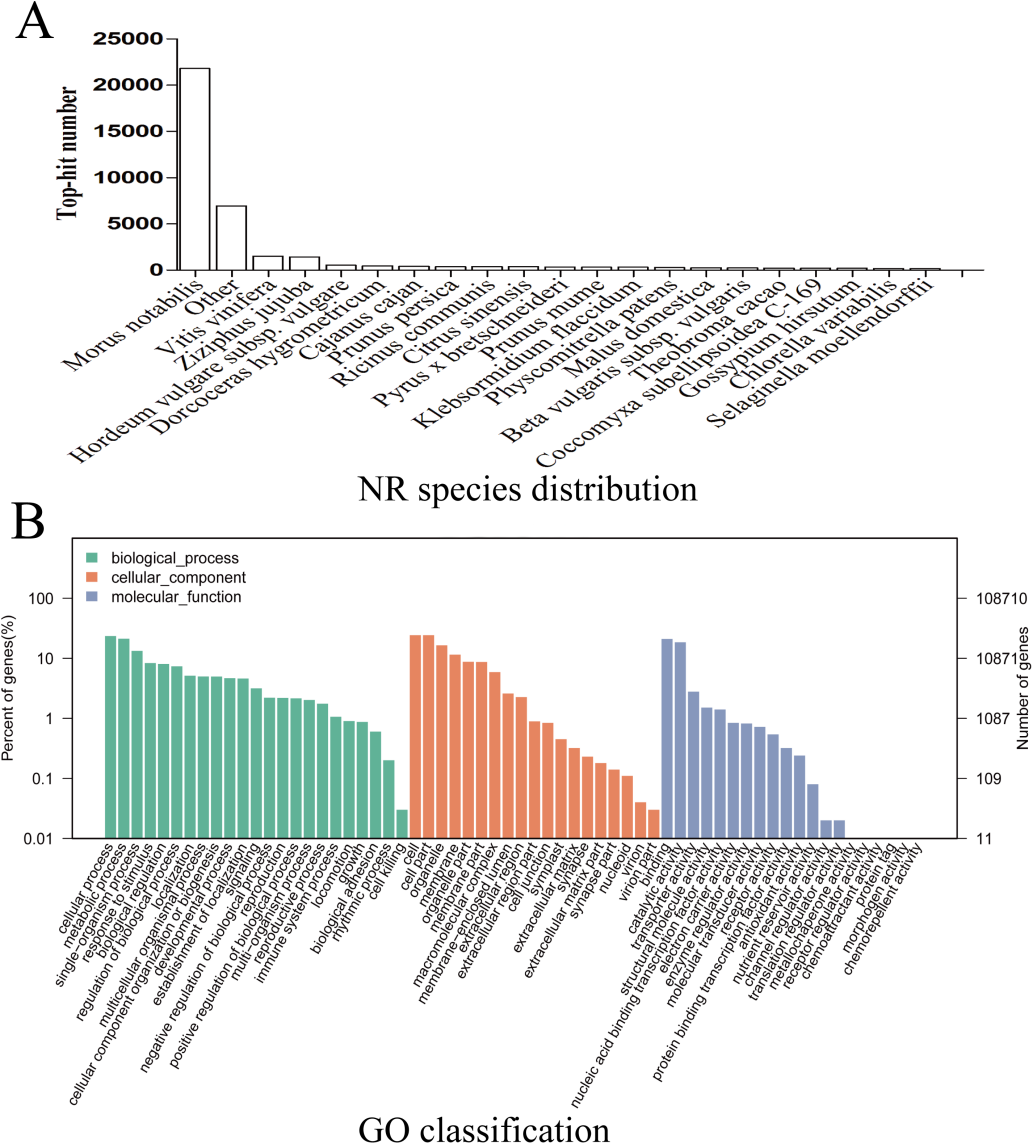
NR species distribution and GO classification of RNA-Seq data. (A) Species distribution of the top 20 BLAST hits for total homologous sequences. (B) Percentage of sequences enrichment in GO classification.

Given the lack of a reference genome for mulberry, 112,481 transcripts were searched using BLAST against nine public databases (Conserved Domains Database, Eukaryotic Orthologous Groups (KOG), NR, NT, Protein Families database, Swiss-Prot, TrEMBL, GO, and KEGG), and 85,949 transcripts were annotated (Fig. S2). The species distribution map according to annotation by BLAST against the NR database is shown in Fig. 3A, and includes *M. notabilis* (58.93%), *Vitis vinifera* (4.06%), *Ziziphus jujuba* (3.87%), *Hordeum vulgare* subsp. vulgare (1.45%), *Dorcoceras hygrometricum* (1.26%), *Cajanus cajan* (1.14%), *Prunus persica* (1.03%), *Ricinus communis* (1.03%), *Citrus sinensis* (1.02%), and *Pyrus bretschneideri* (0.95%). A total of 20,725 unigenes were annotated by BLAST against the COG/KOG database (Fig. S3), and 189,838 transcripts were categorized as a biological process, cellular component, or molecular function in the GO classification (Fig. 3B); 8,655 were assigned to pathway branches for cellular processes, environmental information processing, genetic information processing, metabolism, organismal systems, and human diseases, and were further divided into 323 KEGG pathways (Table S3).

### Analysis of transcripts differently expressed between samples M7 and M11

**Figure 4:**
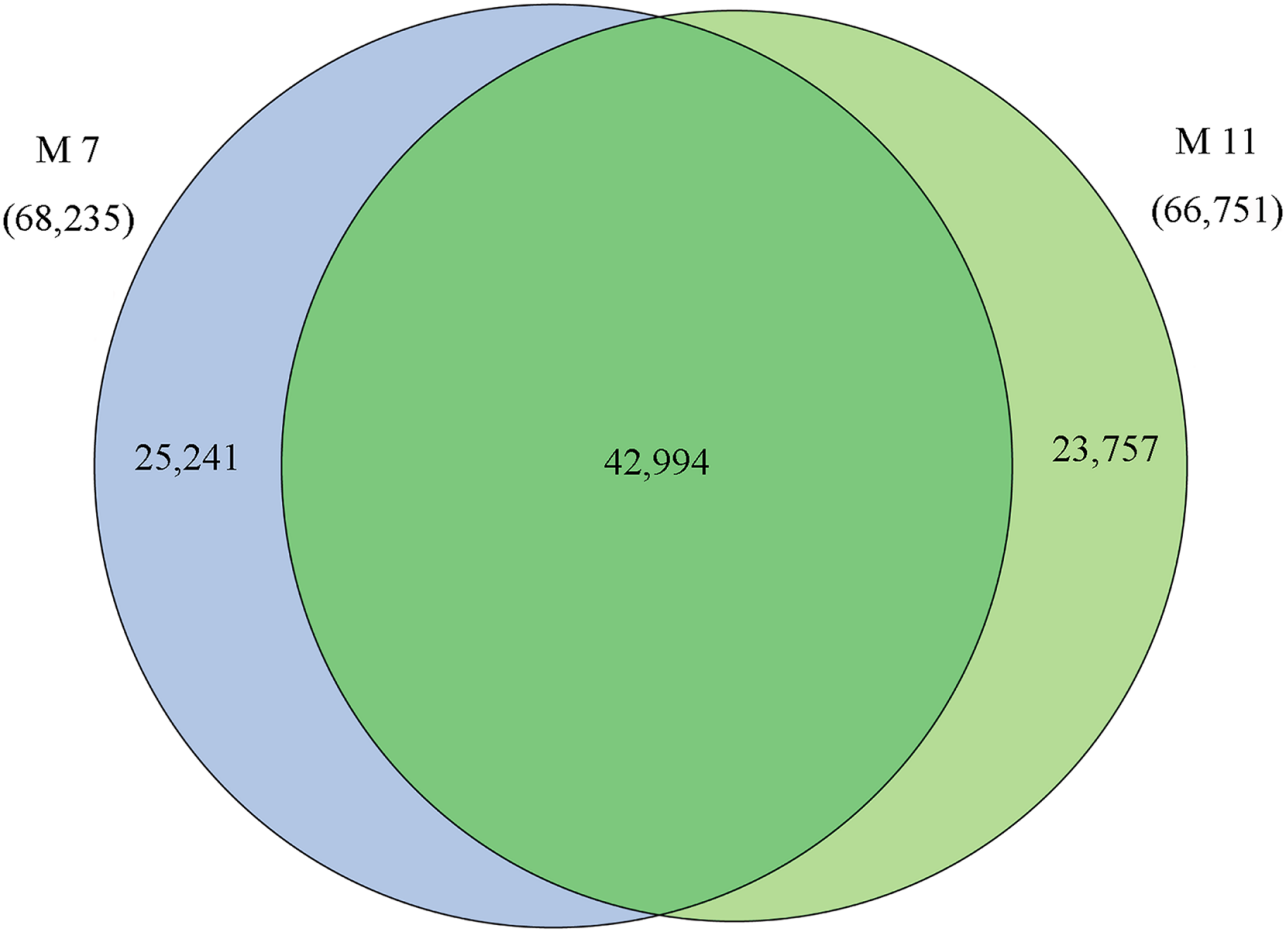
Venn diagram of the statistics of genes expressed in M7 and M11. Overlaps (dark green) represent homologous genes; non-overlapping (blue and light green) parts represent unique genes of two samples, respectively.

**Figure 5:**
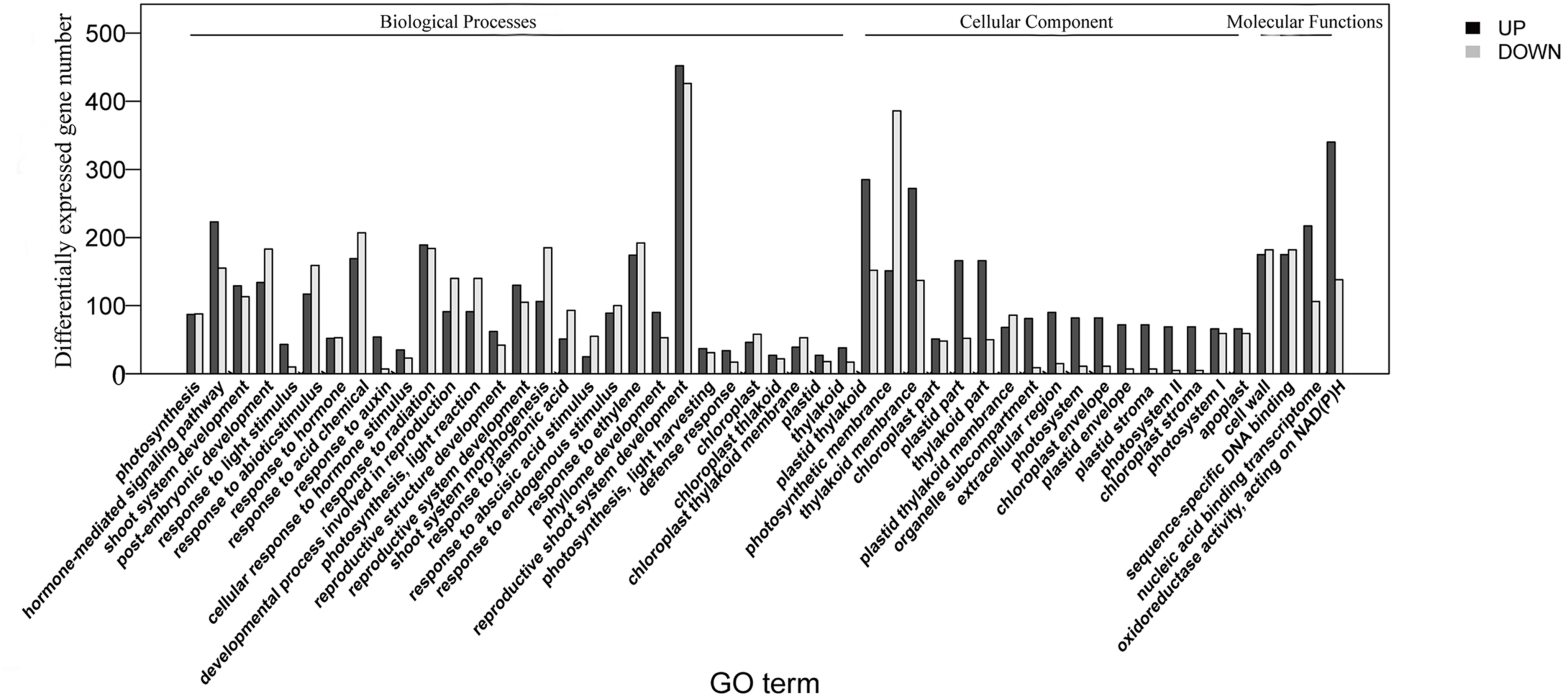
Top 50 enriched GO terms for transcripts differentially expressed between M7 and M11. Black and white stripes indicate up- and down-regulated transcripts, respectively.

The homologies of expressed unigenes from the M7 and M11 datasets are shown in Fig. 4 and Table S4; 68,235 and 66,751 transcripts were expressed in M7 and M11, respectively, with 42,994 homologous unigenes and 25,241 and 23,757 nonhomologous genes in M7 and M11, respectively. Unigenes that were differentially expressed between the two cDNA libraries were compared, with *q*-value < 0.001 and fold change >2 used as thresholds. A total of 11,318 unigenes were found to be differentially expressed, with 6,606 and 4,712 unigenes being up- and downregulated, respectively. The differentially expressed unigenes were annotated as biological processes, cellular components, and molecular functions, and were among the top 50 terms enriched in the GO classification (Fig. 5). Unigenes showing the greatest difference in expression were associated with biological processes, followed by genes corresponding to cellular component. Genes that fell under the molecular functions category did not exhibit large differences in expression between the two samples, and were mostly associated with sequence-specific DNA binding, nucleic acid binding, and oxidoreductase activity. According to the KEGG annotation, these 11,318 differentially expressed unigenes were assigned to 294 KEGG pathways (Table S5), with the highest distribution category being carbon metabolism and biosynthesis of amino acids, followed by oxidative phosphorylation process.

### Identification of candidate genes involved in mulberry DNJ biosynthesis

A DNJ alkaloid biosynthetic pathway biosynthetic pathway was outlined on the basis of differentially expressed transcripts and KEGG pathway assignments. Firstly, lysine was generated using aspartic acid as a substrate, and then the lysine was used as a substrate to generate the DNJ alkaloid; 10 enzymes, encoded by 38 genes, were annotated (Table 2; Table S6). Eight enzymes were annotated in the process of aspartic acid to lysine. The homoserine dehydrogenase (EC: 1.1.1.3) can catalyze L-aspartate 4-semialdehyde to L-homoserine. We did not analyze the enzyme because it does not contribute to the production of lysine. Only two enzymes, LysC (EC: 2.7.2.4) (aspartate kinase) and DapA (EC: 4.3.3.7) (4-hydroxy-tetrahydrodipicolinate synthase), exhibited significantly low expression. LysC (EC: 2.7.2.4), was encoded by 10 transcripts. Two transcripts showed significant downregulation, suggesting that the gene may be related to DNJ alkaloid biosynthesis; four transcripts were annotated as DapA with one showing significant downregulation, suggesting that this transcript may reflect DNJ biosynthesis. Another enzyme, aspartate semialdehyde dehydrogenase (ASD) (EC: 1.2.1.11), was encoded by two transcripts, whereas DapB (EC: 1.17.1.8) was encoded by one transcript. Three transcripts were annotated as ALD/AGD (EC: 2.6.1.83), of which one was downregulated significantly. Three transcripts were annotated as DapF (EC: 5.1.1.7). LysA (EC: 4.1.1.20) was encoded by two transcripts.

**Table 2:** Candidate genes related to DNJ alkaloid biosynthesis in *Morus alba* L. leaves.

Gene	Enzyme	KO id (EC: No)	No. All^a^	No. Up^b^	No. Down^c^
LysC	aspartate kinase	K00928 (2.7.2.4)	10	0	2
ASD	aspartate-semialdehyde dehydrogenase	K00133 (1.2.1.11)	2	0	0
DapA	4-hydroxy-tetrahydrodipicolinate synthase	K01714 (4.3.3.7)	4	0	1
DapB	4-hydroxy-tetrahydrodipicolinate reductase	K00215 (1.17.1.8)	1	0	0
ALD/AGD	LL-diaminopimelate aminotransferase	K10206 (2.6.1.83)	3	0	1
DapF	diaminopimelate epimerase	K01778 (5.1.1.7)	3	0	0
Lys A	diaminopimelate decarboxylase	K01586 (4.1.1.20)	2	0	0
LdcC	Lysine decarboxylase	K00960 (4.1.1.18)	1	0	1
AOC2/3	primary-amine oxidase	K00276 (1.4.3.21)	12	1	3
Total number of Transcripts			38	1	8

**Notes:**

a The total number of transcripts relaetd to DNJ biosynthesis.

b The number of transcripts with up regulated expression significantly in mulberry samples M7 and M11.

c The number of transcripts with down regulated expression significantly in mulberry samples M7 and M11.

In contrast, two downregulated enzymes, LdcC (EC: 4.1.1.18) and AOC2/3 (EC: 1.4.3.21), were assigned to the pyridine ring backbone biosynthetic pathway. LdcC (EC: 4.1.1.20) was encoded by one transcript, showing significant downregulation, suggesting that this transcript may be involved in DNJ biosynthesis. AOC2/3 was represented by 12 transcripts, three of which were significantly downregulated and one of which was significantly upregulated. These four transcripts may thus be involved in the DNJ biosynthesis process. Through the above analysis, nine DEGs were obtained as candidate genes for the DNJ alkaloid biosynthetic pathway. The correlation between the expression level of these genes and the DNJ content was further analyzed to screen the key enzyme genes.

In the conversion of 1-piperidinene to DNJ, CYP450 and methyltransferase genes are speculated to play important roles. In this study, 103 CYP450-related genes in total, including 20 DEGs, were identified (Table S7). A total of 11 DEGs were upregulated and nine were downregulated. In total, 110 methyltransferase genes, including 17 DEGs (Table S8), eight DEGs were upregulated and nine were downregulated. The correlation between DEGs expression and DNJ content was further analyzed.

### Validation of the accuracy of RNA-Seq results

A total of 20 transcripts were randomly selected to validate the accuracy of the RNA-Seq data using qRT-PCR. The validation results shown in Table 3 suggest that the RNA-Seq data are reliable, two fold-change values were greater than one or less than one at the same time, indicating that they have the same trend. This method could be used to identify the DEGs involved in specific pathways of metabolism, and thus, that the data could be used for subsequent analyses. The gene IDs and fragment per kilobases per million mapped reads (FPKM) values are shown in Tables S1, S6 and S9.

**Table 3:** The fold-change of qRT-PCR data and FPKM value between M7 and M11.

Transcripts ID	qRT-PCR (M7/M11)		RNAseq FPKM (M7/M11)	
	Fold-change	*P* Value	Fold-change	*P* Value
c37429_g1	2.59323	0.0328	2.279061	1.03892E-51
c34113_g1	0.186921	0.0023	0.136691	1.64263E-08
c33508_g1	3.827975	0.0093	3.821429	1.56409E-06
c28625_g1	0.145885	0.0051	0.063291	9.90097E-13
c41034_g1	0.273498	0.1268	0.445896	6.55698E-13
c41300_g1	2.49243	0.0030	2.043988	1.49454E-25
c40108_g1	0.305209	0.0169	0.45614	1.98974E-12
c38736_g1	0.319124	0.0183	0.540359	2.72375E-10
c35044_g1	6.510452	0.0002	9.755849	0
c35856_g1	0.27389	0.0050	0.268815	1.81611E-06
c35064_g1	6.161042	0.0004	12.12092	0
c41450_g1	0.130423	0.0302	0.133898	3.10161E-08
c15702_g1	11.42027	0.0001	33.40525	0
c42576_g1	0.33936	0.0548	0.215129	1.38046E-07
c22708_g1	2.88498	0.0011	5.18929	0
c17096_g1	2.166394	0.0033	3.746931	0
c37284_g1	2.067757	0.0041	3.900531	0
c33693_g1	3.684276	0.0023	5.166667	0.548371588
c34152_g1	1.027973	0.7766	1.052699	0
c40176_g1	0.462142	0.0822	0.388889	1.85817E-10

The biological replication result of mulberry RNAseq was shown in Table 4, nine DEGs were found in M7 and M11, and seven DGEs between Ma7 and Ma11 were identified. The BLAST result showed that seven DEGs matched each other with 99–100% coverage, and the changes of the matching sequence were consistent.

**Table 4:** Biological replication of *Morus alba* L. leaves RNA-Seq.

M7 vs M11 (samples in 2016)			Coverage (%)	Ma7 vs Ma11 (samples in 2017)		
Genes ID	Length (bp)	change		Genes ID	Length (bp)	change
**LysC (2.7.2.4)**						
c8863_g1	382	Down	100%	c10822_g1	335	Down
c40311_g1	1,449	Down	100%	c31621_g1	778	Down
**DapA (4.3.3.7)**						
c43595_g1	995	Down	100%	c34113_g1	995	Down
**ALD/AGD (2.6.1.83)**						
c44849_g1	1,559	Down				
**LDCc (4.1.1.18)**						
c83396_g1	492	Down	100%	c32499_g1	1,009	Down
**AOC2/3 (1.4.3.21)**						
c47185_g1	2,160	Down				
c48882_g1	2,157	Down	99%	c40108_g1	2,312	Down
c47618_g1	3,374	Up	99%	c33763_g1	4,956	Up
c32423_g1	1,073	Down	100%	c20125_g1	1,073	Down

**Note:**

M7 and M11 samples were collected on July 31, 2016, and November 15, 2016, respectively. Ma7 and Ma11 samples were collected on July 25, 2017, and November 15, 2017, respectively. Genes were compared using BLASTN.

### Correlation between candidate gene expression and DNJ content of M7 and M11

**Figure 6:**
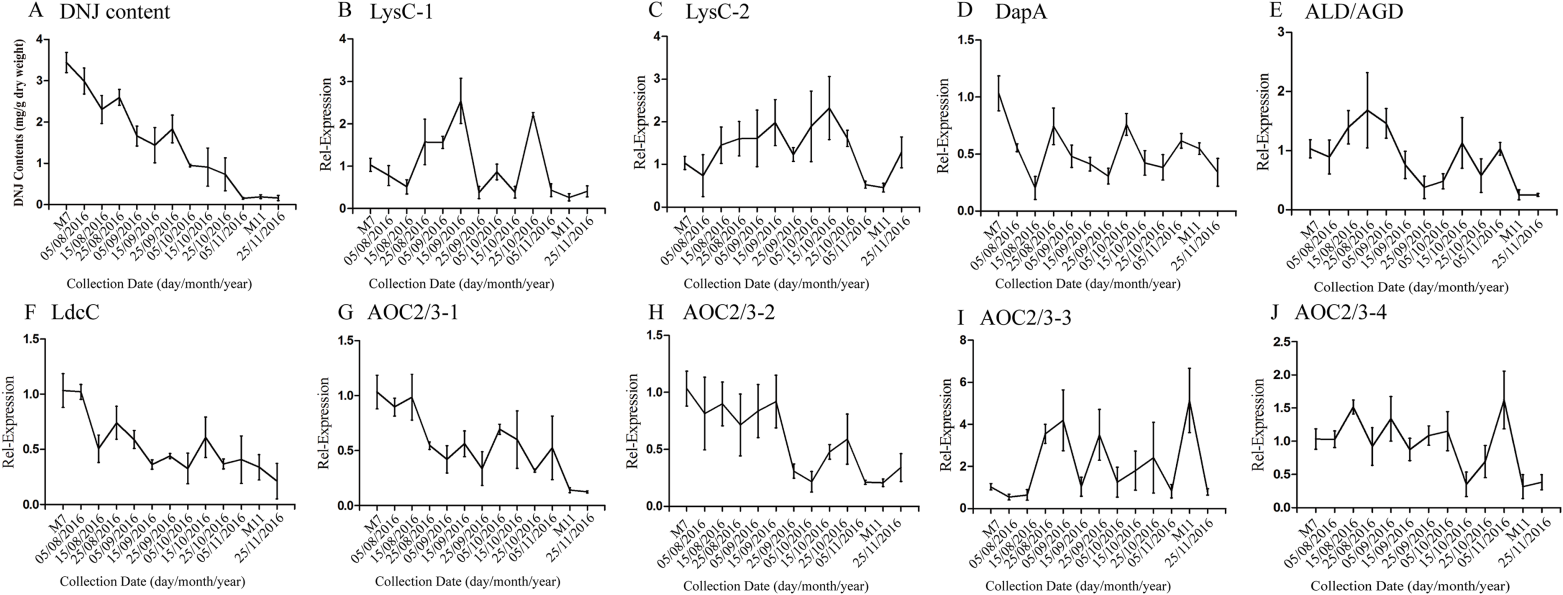
Expression analyses of candidate transcripts related to DNJ biosynthesis at different developmental stages by qRT-PCR. Error bars represent standard deviation. (A) DNJ content; (B) LysC-1, *c8863_g1*; (C) LysC-2, *c40311_g1*; (D) DapA, *c43595_g1*; (E) ALD/AGD, *c44849_g1*; (F) LdcC, *c83396_g1*; (G) AOC2/3-1, *c47185_g1*; (H) AOC2/3-2, *c48882_g1*; (I) AOC2/3-3, *c47618_g1*; and (J) AO2/3C-4, *c32423_g1*.

According to a previous study by our research group, DNJ content exhibits certain regularity with respect to the different growth stages (Ouyang & Chen, 2004); specifically, from April to November the level of DNJ showed a trend from rise to decline, with the peak level occurring at July and August. In this study, mulberry samples were collected from April 15, 2016, to November 25, 2016 for DNJ content determination. The results showed that the DNJ content was higher in M7 than in M11. DNJ contents, the expression of the nine candidate genes, and the relationship between the two are shown in Fig. 6 and Table 5. The expression of three of the transcripts (*c83396_g1*, *c47185_g1*, and *c48882_g1*) showed a decreasing trend that was consistent with the DNJ content. The correlation results indicated that *c83396_g1* expression was significantly positively correlated with DNJ content (*P* < 0.001, Fig. 6F); *c47185_g1* and *c48882_g1* expression levels were positively correlated with DNJ content (*P* < 0.01, Figs. 6G and 6H). The expression of the remaining six transcripts did not correlate with DNJ content. The transcripts *c8863_g1* and *c44849_g1* showed no significant change in August, but increased in September, and fluctuated during the late stages (Figs. 6B and 6E); *c40311_g1* was increased from August to October, and decreased during the late stages (Fig. 6C); similar trends were observed for *c43595_g1*, *c47618_g1*, and *c32423_g1* (Figs. 6D, 6I and 6J).

**Table 5:** The correlation analysis of candidate transcripts and DNJ content.

Correlation		c8863_g1	c40311_g1	c43595_g1	c44849_g1	c83396_g1	c47185_g1	c48882_g1	c47618_g1	c32423_g1
DNJ content	*R* squared	0.0325	0.0002	0.1309	0.2860	0.7544	0.5668	0.6049	0.0248	0.1207
	*P* values	0.5554	0.9629	0.2245	0.0597	0.0001***	0.0030**	0.0017**	0.6075	0.2448

**Notes:**

** Significantly correlated at 0.01 level (two-tailed).

*** Significantly correlated at 0.001 level (two-tailed).

**Figure 7:**
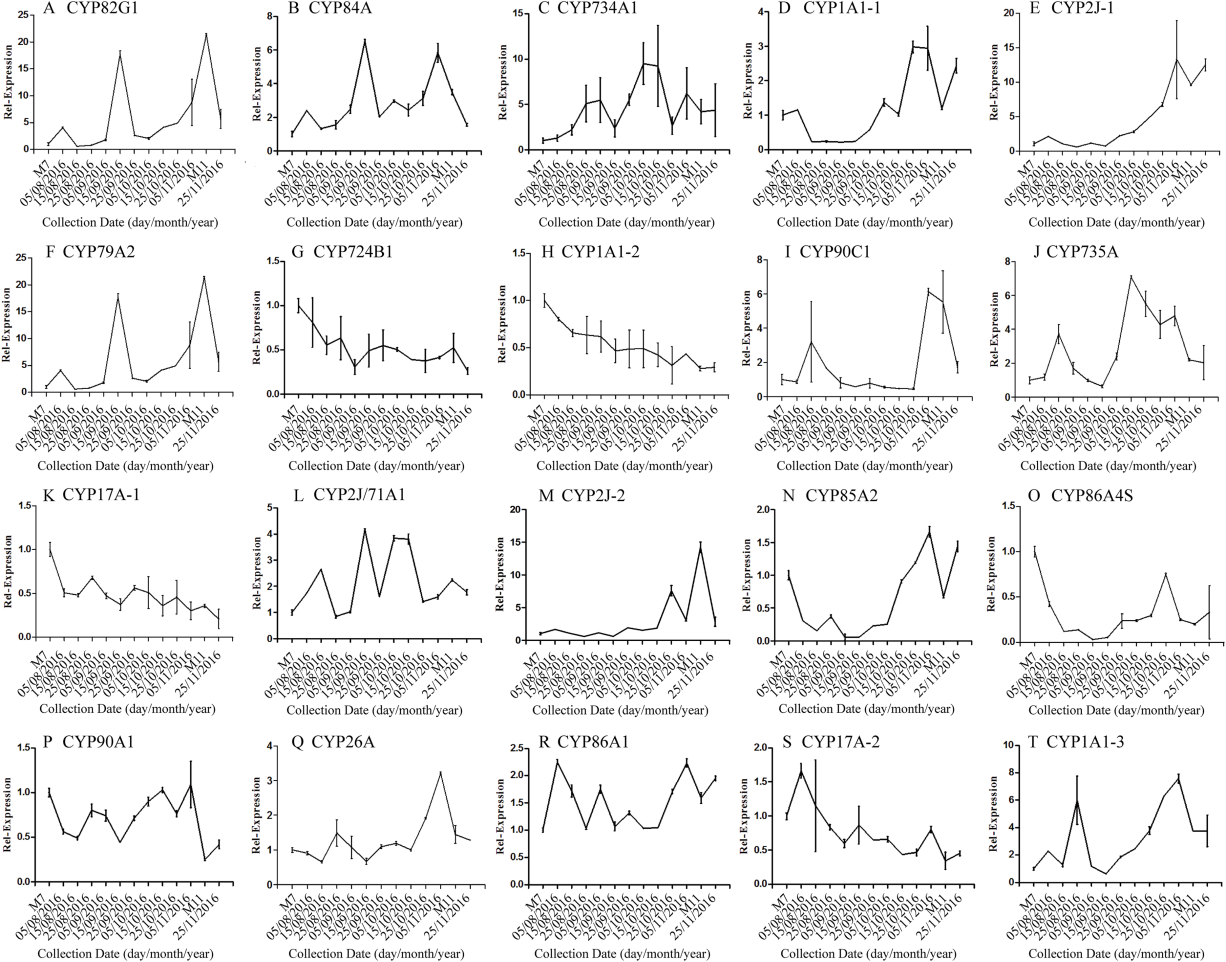
Expression analyses of CYP450 candidate transcripts by qRT-PCR. Error bars represent standard deviation. (A) CYP82G1 c52682_g1, (B) CYP84A c50289_g1, (C) CYP734A1 c47881_g1, (D) CYP1A1-1 c52287_g1, (E) CYP2J-1 c44921_g1, (F) CYP79A2 c41596_g2, (G) CYP724B1 c39525_g1, (H) CYP1A1-2 c29603_g1, (I) CYP90C1 c47981_g2, (J) CYP735A c46601_g1, (K) CYP17A-1 c33960_g1, (L) CYP2J/71A1 c47159_g1, (M) CYP2J-2 c47901_g1, (N) CYP85A2 c51106_g7, (O) CYP86A4S c93311_g1, (P) CYP90A1 c42019_g1, (Q) CYP26A c39327_g1, (R) CYP86A1 c104225_g1, (S) CYP17A-2 c105322_g1, (T) CYP1A1-3 c47780_g1.

**Figure 8:**
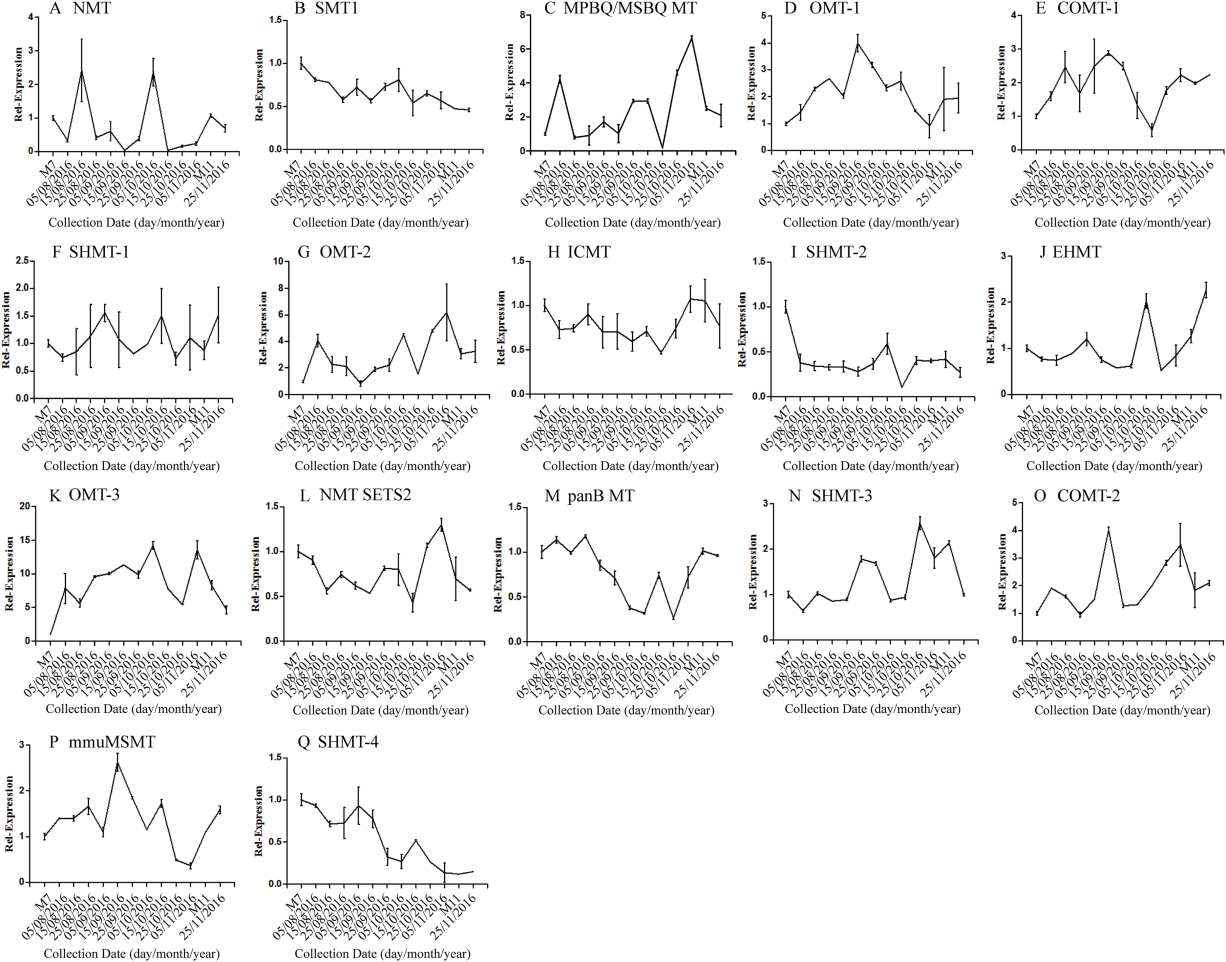
Expression analyses of methyltransferase candidate transcripts by qRT-PCR. Error bars represent standard deviation. (A) NMT c40930_g1, (B) SMT1 c41333_g1, (C) MPBQ/MSBQ MT c61297_g1, (D) OMT-1 c16508_g1, (E) COMT-1 c330_g1, (F) SHMT-1 c14995_g1, (G) OMT-2 c41159_g1, (H) ICMT c50995_g1, (I) SHMT-2 c15006_g1, (J) EHMT c9559_g1, (K) OMT-3 c49082_g2, (L) NMT SETS2 c51779_g2, (M) panB MT c28557_g1, (N) SHMT-3 c45960_g7, (O) COMT-2 c45319_g2, (P) mmuMSMT c16410_g1, (Q) SHMT-4 c43454_g1.

The correlation analysis showed that the transcript level of five CYP450 genes was significantly correlated to DNJ content (Fig. 7; Table 6). The level of the transcript c44921_g1 was significantly and negatively correlated with DNJ content (*P* < 0.001, Fig. 7E). The transcript levels of *c39525_g1*, *c29603_g1*, and *c33960_g1* showed that the expression was significantly postively correlated with DNJ content (*P* < 0.001, Figs. 7G, 7H and 7K). *c105322_g1* expression levels was positively correlated with DNJ content (*P* < 0.01, Fig. 7S). The correlation analysis results showed that two methyltransferase trancripts were significantly correlated to DNJ content (Fig. 8; Table 7). The expression of the transcripts c41333_g1 (Fig. 8B) and c43454_g1 (Fig. 8Q) was significantly and postively correlated with DNJ content (*P* < 0.01, Fig. 8B). The expression of transcript *c43454_g1* was significantly postively correlated with DNJ content (*P* < 0.001, Fig. 8Q). No significant correlations were found between other genes and DNJ content.

**Table 6:** The correlation analysis between CYP450 differently expressed transcripts and DNJ content.

Correlation		c52682_g1	c50289_g1	c47881_g1	c52287_g1	c44921_g1	c41596_g1	c39525_g1	c29603_g1	c47981_g1	c46601_g1
DNJ content	Pearson *r*	−0.5497	−0.4842	−0.5021	−0.5462	−0.8045	−0.5139	0.8113	0.9290	−0.4101	−0.4747
	*R* squared	0.3022	0.2345	0.2521	0.2983	0.6472	0.2641	0.6583	0.8631	0.1682	0.2253
	*P* values	0.0516	0.0936	0.0804	0.0535	0.0009***	0.0724	0.0008***	<0.0001***	0.1640	0.1012

Correlation		c33960_g1	c47159_g1	c47901_g1	c51106_g1	c93311_g1	c42019_g1	c39327_g1	c104225_g1	c105322_g1	c47780_g1
DNJ content	Pearson r	0.8203	−0.3037	−0.5374	−0.4884	0.2797	0.1254	−0.5135	−0.2481	0.7388	−0.4875
	*R* squared	0.6730	0.09221	0.2888	0.2386	0.07821	0.01573	0.2637	0.06156	0.5459	0.2377
	*P* values	0.0006***	0.3131	0.0582	0.0903	0.3548	0.6830	0.0726	0.4137	0.0039**	0.0910

**Notes:**

** Significantly correlated at 0.01 level (two-tailed).

*** Significantly correlated at 0.001 level (two-tailed).

**Table 7:** The correlation analysis between methyltransferase differently expressed transcripts and DNJ content.

Correlation		c40930_g1	c41333_g1	c61297_g1	c16508_g1	c330_g1	c14995_g1	c41159_g1	c50995_g1	c15006_g1
DNJ content	Pearson *r*	0.1007	0.7552	−0.3640	0.0003	−0.1778	−0.2792	−0.4982	−0.0427	−0.0427
	*R* squared	0.0101	0.5704	0.1325	0.0000	0.0316	0.0779	0.2482	0.8898	0.0018
	*P* values	0.7434	0.0028**	0.2215	0.9992	0.5612	0.3556	0.0831	0.018	0.8898

Correlation		c9559_g1	c49082_g2	c51779_g2	c28557_g1	c45960_g1	c45319_g2	c16410_g1	c43454_g1	
DNJ content	Pearson *r*	−0.3701	−0.3696	0.0190	0.4106	−0.5214	−0.4948	0.2199	0.8631	
	*R* squared	0.1369	0.1366	0.0003	0.1686	0.2719	0.2449	0.0484	0.7450	
	*P* values	0.2133	0.2139	0.9509	0.1634	0.0676	0.0856	0.4703	0.0001***	

**Notes:**

** Significantly correlated at 0.01 level (two-tailed).

*** Significantly correlated at 0.001 level (two-tailed).

## Discussion

1-Deoxynojirimycin alkaloid is one of the major secondary metabolites in mulberry (*M. alba* L.), and it has hypoglycemic, antiviral, and other pharmacological effects. However, the DNJ content in mulberry is very low, making it difficult to obtain a large amount of natural DNJ by traditional extraction methods. Therefore, obtaining a large amount of DNJ remains an urgent challenge. With the successful application of artemisinin (Ro et al., 2006), paclitaxel (Ajikumar et al., 2010), tanshinone (Zhou et al., 2012), and other important active ingredients, synthetic biology has become an important approach to obtaining active ingredients for traditional Chinese medicine. However, the premise of using this method is the clarification of the biosynthetic pathway and its key enzyme genes. Therefore, the DNJ alkaloid biosynthetic pathway and its key enzyme gene analysis have become particularly important.

Transcriptomics represents a powerful tool for the analysis of gene expression or to discover valuable information related to the specific genes transcribed. This method has been widely used to identify genes involved in plant metabolism and discover new genes in non-model species (Asamizu, 2000; Martin & Wang, 2011; Yang et al., 2013). In the current study, we compared the transcriptomes of two mulberry leaf samples, with significantly different DNJ contents. In total, 104,988,228 and 64,878,089 high-quality reads were obtained and de novo assembled into 112,481 transcripts, with a final number of unigenes greater than those obtained in previous reports (Du et al., 2016; Wang et al., 2014). To confirm the accuracy of the results, the RNA-Seq data was validated by qRT-PCR. Our results indicated that the RNA-Seq data were reliable and could be used for further study. In this study, the biosynthetic pathway of DNJ alkaloid in mulberry leaves was preliminarily deduced. By analyzing the difference of transcription between the two samples, 10 enzymes and their candidate genes in the biosynthesis pathway of DNJ alkaloid were identified and further analyzed.

On the basis of correlation analysis of candidate gene expression and DNJ content, seven enzymes and corresponding genes were excluded as candidates for DNJ biosynthesis. In particular, LysC (EC: 2.7.2.4) has been identified as an aspartate kinase, the first enzyme in the DAP pathway. This enzyme functions to catalyze the reaction of adenosine triphosphate and L-aspartate to adenosine diphosphate and 4-phospho-L-aspartate (Véron, Falcoz-Kelly & Cohen, 1972). Studies have shown that LysC activity and its RNA expression levels are higher in *Arabidopsis thaliana* and tobacco tender leaves than in the old leaves and are associated with lysine accumulation (Galili, 1995; Vauterin, Frankard & Jacobs, 1999). In the current study, ten transcripts were found that encoded this enzyme, showed no correlation to DNJ biosynthesis (Figs. 6B and 6C). ASD (EC: 1.2.1.11) can catalyze L-aspartate 4-semialdehyde and phosphate to L-4-aspartyl phosphate (Black & Wright, 1955). Thus, ASD is responsible for the conversion of aspartate to lysine (Cremer et al., 1988). Two transcripts were found encoding this enzyme, although no obvious differences were found between the different mulberry growth stages. DapA (EC: 4.3.3.7) participates in lysine biosynthesis (Cremer, Eggeling & Sahm, 1990) by catalyzing the conversion of pyruvate and l-aspartate-4-semialdehyde to (2S,4S)-4-hydroxy-2,3,4,5-tetrahydrodipicolinate and H_2_O (Soares Da Costa et al., 2010). In this study, four transcripts were found to encode 4-hydroxy-tetrahydrodipicolinate synthase, of which *c43595_g1* showed low expression in M11. DapB (EC: 1.17.1.8) participates in (2S,4S)-4-hydroxy-2,3,4,5-tetrahydrodipicolinate synthesis (Farkas & Gilvarg, 1965). One transcript (*c45119_g1*) was identified that encoded this enzyme, which showed a somewhat but not significantly decreased level of expression. ALD/AGD (EC: 2.6.1.83), a pyridoxal-phosphate enzyme, functions in one of the final steps in the lysine-biosynthesis pathway of plants (Hudson et al., 2006). Among three transcripts related to this enzyme, one (*c44849_g1*) was downregulated in M11. DapF (EC: 5.1.1.7) has been defined as diaminopimelate epimerase, the main function of which is to catalyze LL-2, 6-diaminoheptanedioate to meso-diaminoheptanedioate. Notably, this enzyme activity has been detected in plant extracts (Hudson et al., 2005). We identified three transcripts that encoded this enzyme, although no correlation to DNJ biosynthesis was found. LysA (EC: 4.1.1.20) comprises one of the enzymes involved in the last step of lysine biosynthesis, as it was identified as a diaminopimelate decarboxylase and catalyzes meso-2, 6-diaminoheptanedioate to L-lysine (Wallsgrove & Mazelis, 1980). In the current study, two transcripts were found to contribute to this enzyme, for which no correlations with DNJ biosynthesis were detected.

Only LdcC and AOC2/3 and the corresponding genes were considered as candidate key enzymes/genes for DNJ biosynthetic pathways. LdcC (EC: 4.1.1.18) is the first key enzyme in a variety of alkaloid biosynthesis. It requires pyridoxal phosphate as a coenzyme to catalyze lysine decarboxylation to form cadaverine. Studies have shown that LDC activity is significantly correlated with the amount of alkaloid produced (Pelosi, Rother & Edwards, 1986). In this study, one gene (*c83396_g1*) encoding LdcC, showed significantly reduced expression. Correlation analysis of the gene expression level and DNJ content showed a significant correlation (*P* < 0.001, Fig. 6F), suggesting that this gene may encode the key enzyme gene in DNJ biosynthesis in mulberry leaves. AOC2/3 (EC: 1.4.3.21) is a AOC in the DNJ biosynthesis pathway and belongs to a group of enzymes that oxidize primary monoamines but have little or no activity toward diamines (Tipping & McPherson, 1995). The enzyme catalyzes the decarboxylation of lysine to produce cadaverine, further resulting in a piperidine ring structure (Sun et al., 2012). In this study, the expression of this enzyme was significantly decreased, which was consistent with the accumulation of DNJ alkaloid, suggesting that it could be a key enzyme for DNJ biosynthesis. The enzyme activity and its correlation with DNJ content will be further analyzed. Among 12 transcripts annotated to this enzyme, transcripts *c47185_g1* and *c48882_g1* showed significantly positive correlation to DNJ content (*P* < 0.01, Figs. 6G and 6H), suggesting that they are the key genes in the DNJ alkaloid biosynthesis pathway. Further work is being carried out to clarify the gene cloning, function and the role in DNJ biosynthesis.

On the basis of previous reports, we speculated that CYP450 and methyltransferase might be involved in the hydroxylation and methylation of the 1-piperidine structure in the final reaction of the DNJ synthetic pathway. Based on our transcriptome data, five CYP450 genes and two methyltransferase genes were found to be significantly correlated with DNJ content, suggesting that they might be key genes in the DNJ biosynthetic pathway, and our teams are currently validating their function to lay the foundation for elucidating the DNJ biosynthetic pathway. To overcome the limitations of the experimental design, we conducted an additional RNA-Seq study on two samples, Ma7 and Ma11, collected from an independent tree on July 2017 and November 2017. Seven DEGs identified in the main 2016 experiment were validated in 2017 samples, and only two DEGs were not confirmed. Such a good reproducibility indicates that our transcriptome data are robust and valid for *M. alba* species.

## Conclusions

In this study, RNA-Seq was used to identify key genes involved in the DNJ biosynthetic pathway in mulberry leaves. The DNJ biosynthetic pathway was preliminarily speculated. It was presumed that DNJ in mulberry leaves is derived from lysine, and lysine is derived from the aspartic acid–DAP pathway. The conversion of lysine to DNJ was predicted to involve the conversion of lysine by LDC to produce cadaverine, followed by the production of a piperidine ring structure by AOC. The expression levels of three key genes encoding LDC and AOC were significantly correlated with DNJ content. Five CYP450 genes and two methyltransferase genes were significantly associated with DNJ content, suggesting that 1-piperidinene may be catalyzed by CYP450 enzymes and methyltransferases to DNJ alkaloid. Cloning experiments and function analysis are currently being conducted to elucidate the role of these candidate genes in DNJ synthesis of *M. alba*.

## Supplemental Information

10.7717/peerj.5443/supp-1 Supplemental Information 1 Fig. S1. Statistics of assembly length for transcripts.

10.7717/peerj.5443/supp-2 Supplemental Information 2 Fig. S2. The number of sequences with functional annotations in each database.

10.7717/peerj.5443/supp-3 Supplemental Information 3 Fig. S3. KOG categories of transcripts.

10.7717/peerj.5443/supp-4 Supplemental Information 4 Table S1. The Gene IDs and FPKM of 20 random transcripts.

10.7717/peerj.5443/supp-5 Supplemental Information 5 Table S2. Primers used in this study.

10.7717/peerj.5443/supp-6 Supplemental Information 6 Table S3. All transcripts assigned to 323 KEGG pathways between the two mulberry libraries.

10.7717/peerj.5443/supp-7 Supplemental Information 7 Table S4. Expression statistics of transcripts between M7 and M11 libraries.

10.7717/peerj.5443/supp-8 Supplemental Information 8 Table S5. Significantly differentially expressed transcripts assigned to 294 KEGG pathways between the two mulberry libraries.

10.7717/peerj.5443/supp-9 Supplemental Information 9 Table S6. The FPKM of transcripts involve in DNJ biosynthesis pathway.

10.7717/peerj.5443/supp-10 Supplemental Information 10 Table S7. CYP450 related gene statistics in *Morus alba* L. leaves transcriptome data.

10.7717/peerj.5443/supp-11 Supplemental Information 11 Table S8. Methyltransferase related gene statistics in *Morus alba* L. transcriptome data.

10.7717/peerj.5443/supp-12 Supplemental Information 12 Table S9. All differentially expressed transcripts involved in DNJ biosynthesis transcripts.

10.7717/peerj.5443/supp-13 Supplemental Information 13 Raw data.

## Additional Information and Declarations

### Data Availability

The following information was supplied regarding data availability: M7 and M11: STUDY: PRJNA427492 (SRP127713) Ma7 and Ma11: STUDY: PRJNA438058 (SRP135584)
