# Supplementary figures and images for: Transcriptome analysis and identification of key genes involved in 1-deoxynojirimycin biosynthesis of mulberry (Morus alba L.)
Source: PeerJ. 2018 Aug 23;6:e5443. doi: 10.7717/peerj.5443 (PMC6109587; doi:10.7717/peerj.5443)

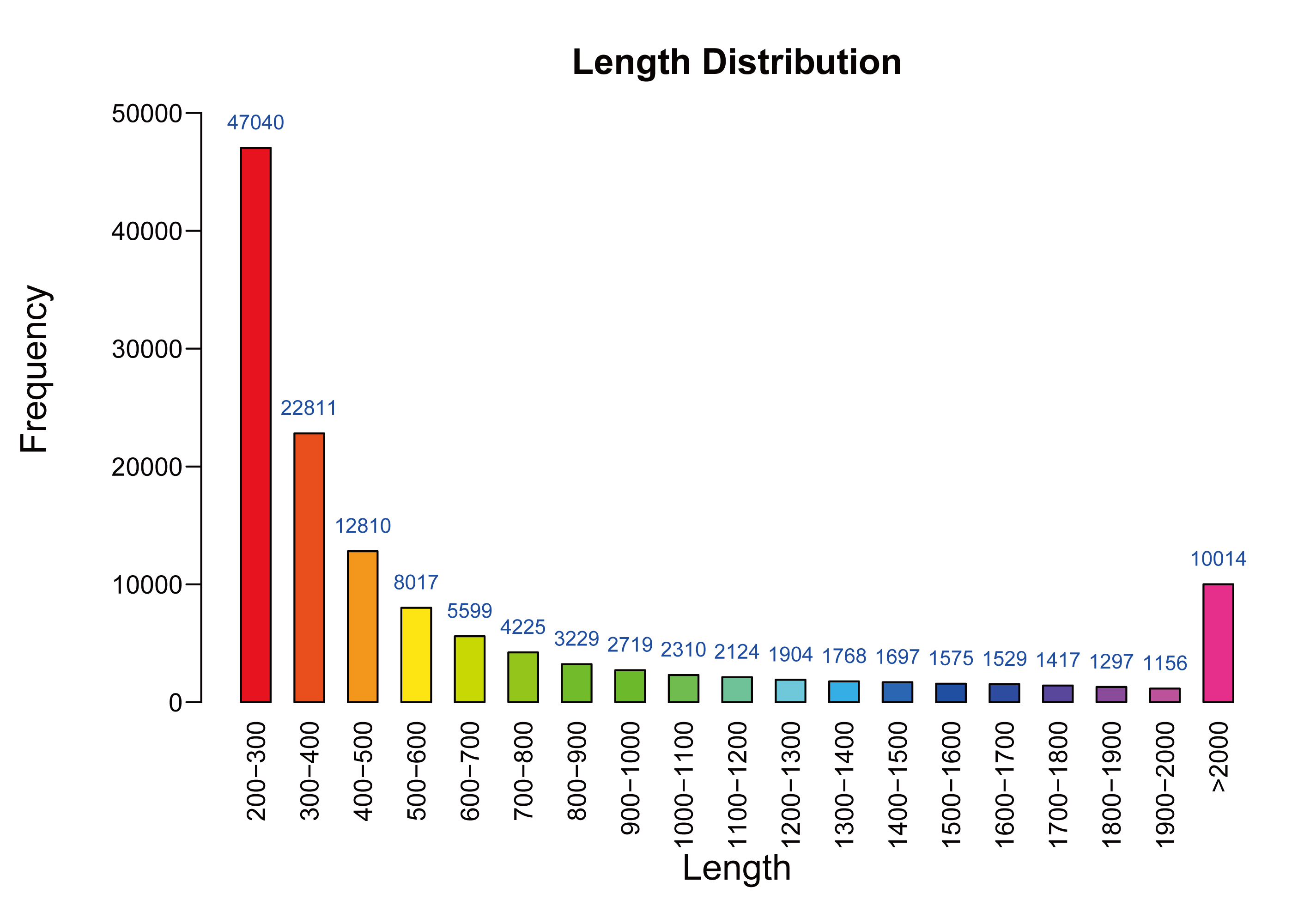

Supplement: Supplemental Information 1 [file peerj-06-5443-s001.png]

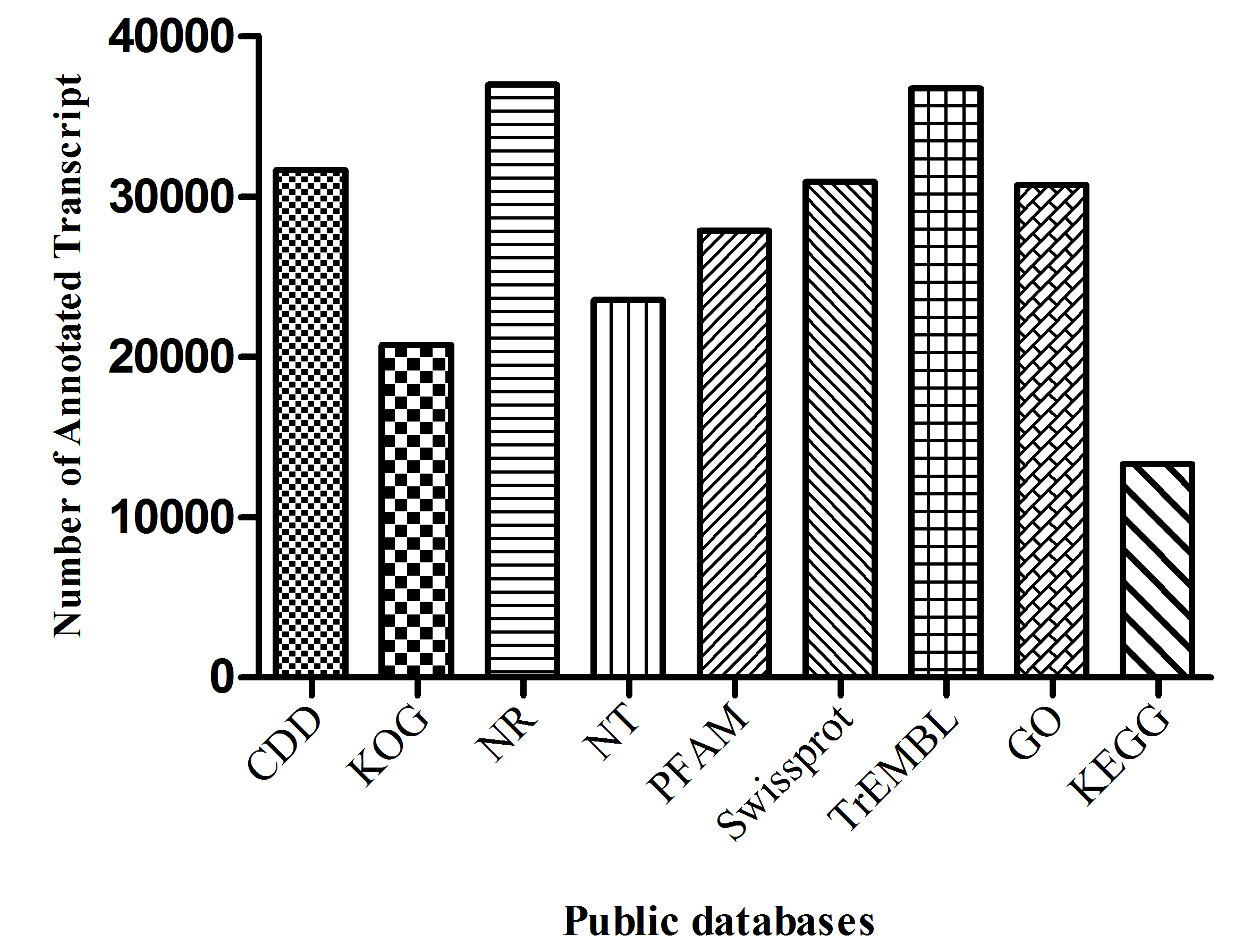

Supplement: Supplemental Information 2 [file peerj-06-5443-s002.png]

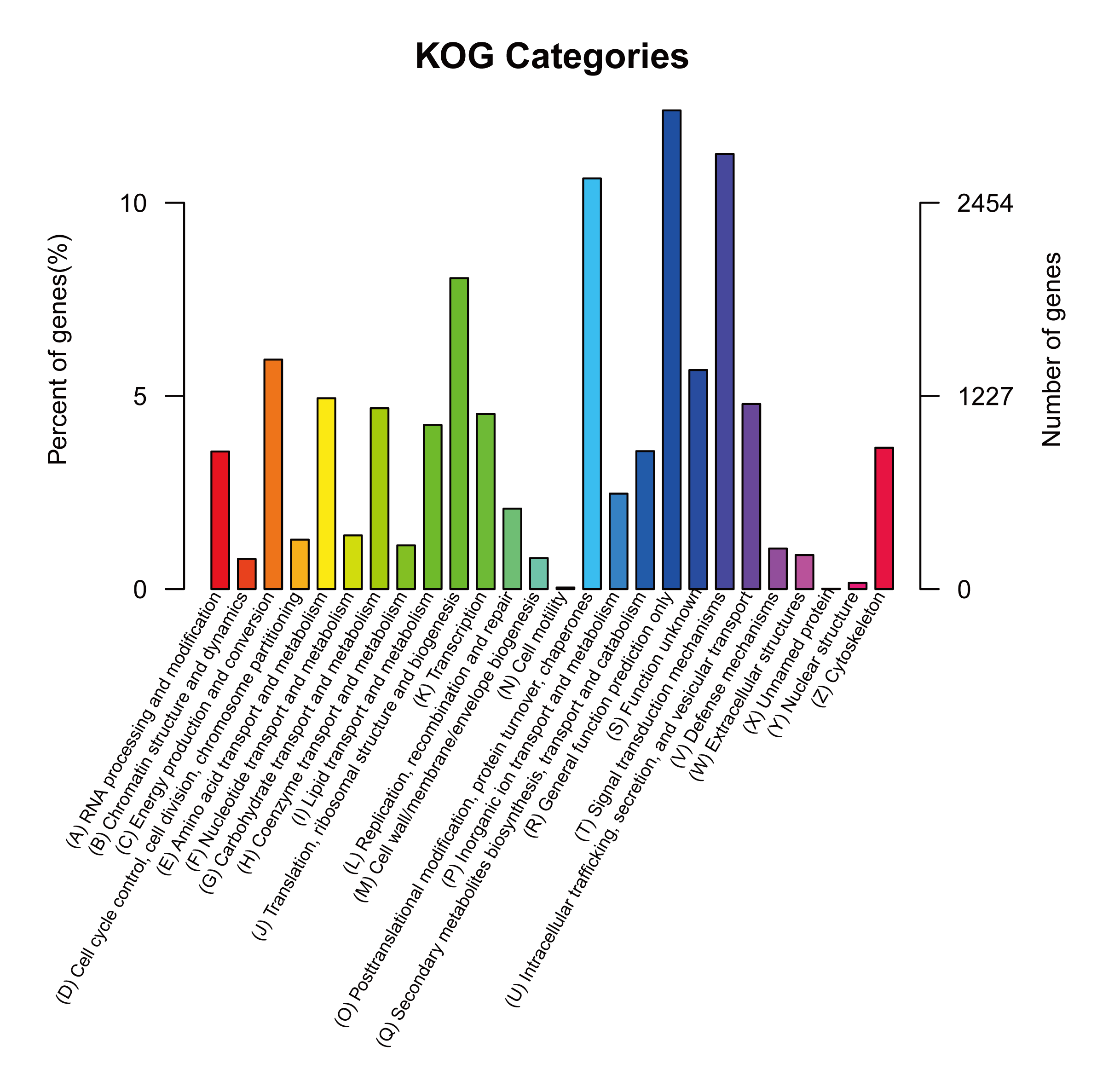

Supplement: Supplemental Information 3 [file peerj-06-5443-s003.png]
