# Supplementary material for: Transcriptome analysis and identification of key genes involved in 1-deoxynojirimycin biosynthesis of mulberry (Morus alba L.)
Source: PeerJ. 2018 Aug 23;6:e5443. doi: 10.7717/peerj.5443 (PMC6109587; doi:10.7717/peerj.5443)
Supplement: Supplemental Information 4 [file peerj-06-5443-s004.doc]

**Table S1 The Gene IDs and FPKM of 20 random transcripts**

| **Transcripts** | **M7 FPKM** | **M11 FPKM** | **P Value** | **Mark** |
| --- | --- | --- | --- | --- |
| c37429_g1 | 63.13 | 27.7 | 1.03892E-51 | Down |
| c34113_g1 | 0.19 | 1.39 | 1.64263E-08 | Up |
| c33508_g1 | 1.07 | 0.28 | 1.56409E-06 | Down |
| c28625_g1 | 0.05 | 0.79 | 9.90097E-13 | Up |
| c41034_g1 | 2.39 | 5.36 | 6.55698E-13 | Up |
| c41300_g1 | 6.97 | 3.41 | 1.49454E-25 | Down |
| c40108_g1 | 6.24 | 13.68 | 1.98974E-12 | Up |
| c38736_g1 | 19.28 | 35.68 | 2.72375E-10 | Up |
| c35044_g1 | 19699.79 | 2019.28 | 0 | Down |
| c35856_g1 | 6714.3 | 24977.38 | 1.81611E-06 | Up |
| c35064_g1 | 5404.96 | 445.92 | 0 | Down |
| c41450_g1 | 847.41 | 6328.79 | 3.10161E-08 | Up |
| c15702_g1 | 597.62 | 17.89 | 0 | Down |
| c42576_g1 | 570.19 | 2650.46 | 1.38046E-07 | Up |
| c22708_g1 | 1318.91 | 254.16 | 0 | Down |
| c17096_g1 | 1498.81 | 400.01 | 0 | Down |
| c37284_g1 | 1057.2 | 271.04 | 0 | Down |
| c33693_g1 | 0.93 | 0.18 | 0.548371588 | Down |
| c34152_g1 | 73.91 | 70.21 | 0 | Down |
| c40176_g1 | 1.54 | 3.96 | 1.85817E-10 | Up |
