# Supplementary material for: Transcriptome analysis and identification of key genes involved in 1-deoxynojirimycin biosynthesis of mulberry (Morus alba L.)
Source: PeerJ. 2018 Aug 23;6:e5443. doi: 10.7717/peerj.5443 (PMC6109587; doi:10.7717/peerj.5443)
Supplement: Supplemental Information 5 [file peerj-06-5443-s005.doc]

**Table S2A** **Primers of random transcripts used in this study**

| **Primer name** | **Forward primer sequence (5′to 3′)** | **Reverse primer sequence (5′to 3′)** |
| --- | --- | --- |
| β-actin | CATTGTCTTGTCTGGTGGTT | TCATCATACTCCGACTTTGC |
| c37429_g1 | AGAAAGCCTGGTGAAGAG | ATTTGGGCATATCCGTAG |
| c34113_g1 | GGTGAAGGCAAGTAAGAC | GTAGCCGCAGTTACAAAT |
| c33508_g1 | AACCTGTTTAGCCTCTGG | TTACGGTGTAGGGTGGTC |
| c28625_g1 | CCAATCATACAGCCCAGCAA | AAGCGGGTTCCATTTCTC |
| c41034_g1 | AGAGGTGTTGCCTTGTCG | CCATTGGGTTGCCAGAGT |
| c41300_g1 | AGAGTCCATTACGCTTCG | GTCTGTGCCTTCTGCTTT |
| c40108_g1 | GTAGACGGCGAAGCAAAT | CGTAACCCAAACATCATAG |
| c38736_g1 | ATGCCTGCCTTGCCTTAG | GCACCTTCAAACCCACCT |
| c35044_g1 | GGGCCACCCAAGTCATCT | ATAGCAAGCCTGCCGTTC |
| c35856_g1 | TGGTGATGTCGTCGGGAG | CGTGGCTGAACAATGTGG |
| c35064_g1 | TTAGTATAATCTATTTCGG | CTTTCTATGTGGACTCTGT |
| c41450_g1 | ACATAGACAGGGCACCAT | ATCCTACCCGACGAAGAC |
| c15702_g1 | GTAATGCGGGTCTCCTAA | TGATGTGGTGATTGTGGG |
| c42576_g1 | AACCCTGTCATCATTCGT | ACAACCTGTTGCCGTAGA |
| c22708_g1 | CTCCCACCACAATCTTTC | GCTCAGTCATCCCAGTCC |
| c17096_g1 | TCCGAAGAAGAAGCCACC | CCAACCTCCCTGCCTACA |
| c37284_g1 | CAAGCAACAAAGGGCACCAT | TATCCGACCCGATCCACAAC |
| c33693_g1 | TGGGATTTAGACATGTTTTC | ACGCTCCATTGTATTGATAG |
| c34152_g1 | ACTTTCTGCTGGTAGGT | GCATCGGTATAGTGTCC |
| c40176_g1 | GCAATGGACACTATACCG | TGATGGAGCACTCGAACT |

**Table S2B Primers of 20 CYP450 differentially expressed transcripts**

| **Primer name** | **Forward primer (5'->3')** | **Reverse primer (5'->3')** |
| --- | --- | --- |
| c52682_g1 | TGGTGATGTTGAGAGGGACC | GAACCCATCTGTCCCTACCC |
| c50289_g1 | TCGATAAACCCATCAAGCGC | CGTCTTCAAGCTCACCATGG |
| c47881_g1 | GTTGTACACGATGTCACCCG | AAGGCCTTCAGTGAGACCAA |
| c52287_g1 | ATGCGTTCTCCACCTTCTGA | ACTCTCGCCATCATCACTGT |
| c44921_g1 | CCAAGAAGAGGTGAGGGGAG | TTGGGTCCCTTTGAATTGCC |
| c41596_g2 | ACCCTTGTAGAAGCCTTCCC | GTAGTGTCTTGCTTAGCCGC |
| c39525_g1 | GTTCCGCAGCTTCTTGTGAA | ATCTCATCTTTTCGGCTCGC |
| c29603_g1 | CTCTGAATTCCTGAACGCGG | ACAACACGCCCAACTTCTTC |
| c47981_g2 | TCTGAGGTGATTCGGGCTTT | ACAAATGCCGACGTGAACAA |
| c46601_g1 | AGAGGACAGATTCGTGGCAA | CAGACGGATTTTGGAGCTGG |
| c33960_g1 | AAAACTTCAAGTGGGGTCGC | GAGATTTCTGACAGCCTGCG |
| c47159_g1 | TCGACCAAGCAAAGGAAAGC | TTGCCAGAGGACTTGGACAT |
| c47901_g1 | GGAACAAGAACCGCGAATGT | ATCAAAGGCCACCAACGTTC |
| c51106_g7 | ACAAGGCCCTGACTTCATGA | TGAGGGTAGCCAGGAACAAG |
| c93311_g1 | AAGCCAAGCCACAGATGTTG | TCTTCCATGATCTCCTCGGC |
| c42019_g1 | CGGTAGGTGGTGGAGAAGAG | TCCGGCTCAATATGGACTCC |
| c39327_g1 | GACCATCCACGTTACTGCAC | TGACAGGAGAAGAGGCTTGG |
| c104225_g1 | GAAAGCTCACCGGTCCAAAG | AAAGAAAGGCAGGGCGATTG |
| c105322_g1 | GCATAGGTGAGGCGGGATAT | TCTCTTCGGTAGGGCCCTAT |
| c47780_g1 | CGAAAACCACTGCTGCTGAT | ATTGGCAAGCTCGACGTATC |

**Table S2C Primers of 17 methyltransferase differentially expressed transcripts**

| **Primer name** | **Forward primer (5'->3')** | **Reverse primer (5'->3')** |
| --- | --- | --- |
| c40930_g1 | ATTGTCTGTCCTTCCACCGT | GATGTCACATCAGCGCACAT |
| c41333_g1 | GGGCCTTCACCATGTTCTTG | GGTTGACGGGAAAATGCCTT |
| c61297_g1 | GTACACAGGACCAATCACGC | AGTCACCTCATCAGCTTGCT |
| c16508_g1 | TGAGCATGGTTTCTGAGGGT | ACCACCGCTCTTACAGTCTT |
| c330_g1 | GCTCCTTTCCTTGGCTCCTA | TGTTCCAAAGCTATTCCGACC |
| c14995_g2 | ACGGCGAAGGAGATCAAGTT | CTCTCTGCAACACCACCAAC |
| c41159_g1 | CCGAGGAGCCAAAGAATGTG | CTGCAACTTGGAAGGACACC |
| c50995_g1 | CTGCTCGCATACAGTTGTCC | AACCACAACCATCACAAGCC |
| c15006_g2 | GCCACCAACCCCTTTCTCTA | GTTAGTAGCCGTGGGTGAGA |
| c9559_g1 | TTGCCAGCATCATTGTCCAC | TGAATGTGGCCCAAACTGTG |
| c49082_g2 | TGCCCTAAACGACATGGTCT | CGTCGTCAAGTGAATCGGAC |
| c51779_g2 | CTTGGTTTTCGTTGGCCTCA | AGCTGTTGTCCCAATACCGA |
| c28557_g1 | AATCGGGAATTGCGGTGATG | CCACAGGTCCAGGTACACAT |
| c45960_g7 | GCAGTTGGATCCGTCATGAC | GCAAGTCAAGGGCCATGATT |
| c45319_g2 | GCCATTGAGCTCGGAGTTTT | ACTATTGCCATCACCCACCA |
| c16410_g1 | CTGACGAAGGGGATGCATTG | CTTCAGGTCCGATGCACATG |
| c43454_g1 | TTCCGCTAGCTTGTCGTAGT | AAGTGGAGAAGACTGGGGTG |
