# Supplementary material for: Transcriptome analysis and identification of key genes involved in 1-deoxynojirimycin biosynthesis of mulberry (Morus alba L.)
Source: PeerJ. 2018 Aug 23;6:e5443. doi: 10.7717/peerj.5443 (PMC6109587; doi:10.7717/peerj.5443)
Supplement: Supplemental Information 6 [file peerj-06-5443-s006.doc]

**Table S3 All transcripts assigned to 323 KEGG pathways between the two mulberry libraries.**

| **Pathway** | **ko ID** | **# of Transcripts** | **Transcripts** |
| --- | --- | --- | --- |
| >1. Metabolism |  |  |  |
| 2-Oxocarboxylic acid metabolism | ko01210 | 177 | c104558_g1,c6029_g1,c25134_g1,c11263_g1,c110883_g1,c91379_g1,c88604_g1,c95034_g1,c80465_g1,c106588_g1,c105979_g1,c49462_g1,c52927_g1,c16964_g1,c71149_g1,c48533_g1,c100006_g1,c40311_g1,c79442_g1,c19206_g1,c95068_g1,c27203_g1,c80695_g1,c37292_g1,c94505_g1,c103393_g1,c40621_g1,c110039_g1,c105038_g1,c52959_g1,c32971_g1,c55416_g1,c51781_g1,c84795_g1,c945_g1,c19834_g1,c110385_g1,c66158_g1,c66611_g1,c80270_g1,c67039_g1,c49098_g3,c67769_g1,c47003_g1,c31507_g1,c49757_g3,c1350_g1,c42785_g1,c41596_g2,c945_g2,c83854_g1,c54745_g1,c39609_g1,c61242_g1,c89066_g1,c99893_g1,c71092_g1,c26218_g1,c97409_g1,c66195_g1,c42133_g1,c65042_g1,c101216_g1,c48574_g1,c100842_g1,c19508_g1,c81098_g1,c51479_g1,c85914_g1,c75960_g1,c53308_g1,c64274_g1,c57611_g1,c97253_g1,c12938_g1,c104555_g1,c4149_g1,c78386_g1,c44303_g1,c72940_g1,c100120_g1,c93338_g1,c71601_g1,c12938_g2,c83062_g1,c9388_g1,c63436_g1,c28212_g1,c1888_g2,c66239_g1,c78544_g1,c44012_g1,c11348_g1,c50389_g1,c16638_g1,c10908_g1,c108108_g1,c74727_g1,c34013_g1,c25225_g1,c81417_g1,c66906_g1,c48894_g1,c32058_g1,c41819_g1,c48887_g1,c79123_g1,c8863_g1,c62096_g1,c51583_g1,c48887_g2,c86658_g1,c18329_g1,c51740_g1,c43327_g1,c84626_g1,c87783_g1,c104622_g1,c9286_g1,c95011_g1,c84138_g1,c67249_g1,c62780_g1,c12358_g1,c102419_g1,c42054_g1,c47479_g1,c104877_g1,c73923_g1,c79133_g1,c35770_g1,c104829_g1,c75192_g1,c66191_g1,c43501_g1,c41818_g1,c104455_g1,c1753_g1,c89336_g1,c12516_g1,c46556_g1,c61619_g1,c54565_g1,c42387_g1,c96844_g1,c16761_g1,c26363_g1,c41580_g1,c14803_g1,c71327_g1,c3742_g1,c71604_g1,c95977_g1,c94269_g1,c70771_g1,c28032_g1,c29000_g1,c65758_g1,c57820_g1,c72642_g1,c94496_g1,c83296_g1,c86327_g1,c95128_g1,c79025_g1,c67666_g1,c48449_g1,c43722_g1,c56269_g1,c64536_g1,c96110_g1,c63265_g1,c9318_g1,c39238_g1,c46331_g1,c47551_g1,c46851_g1 |
| Acridone alkaloid biosynthesis | ko01058 | 1 | c39803_g1 |
| Aflatoxin biosynthesis | ko00254 | 3 | c51829_g1,c84449_g1,c69630_g1 |
| Alanine | ko00250 | 177 | c45383_g1,c64692_g1,c5971_g1,c1696_g1,c51194_g1,c110883_g1,c48472_g2,c50984_g1,c51800_g4,c22478_g1,c72302_g1,c74905_g1,c50809_g1,c17547_g1,c106588_g1,c88947_g1,c103062_g1,c68570_g1,c83165_g1,c63776_g1,c100006_g1,c77060_g1,c19206_g1,c11424_g1,c53306_g1,c12659_g1,c80695_g1,c97022_g1,c104802_g1,c59098_g1,c100364_g1,c44498_g1,c40621_g1,c110441_g1,c42270_g1,c40487_g1,c56123_g1,c100459_g1,c110238_g1,c18163_g1,c86834_g1,c100241_g1,c83834_g1,c95320_g1,c90856_g1,c76378_g1,c24284_g1,c43711_g1,c73821_g1,c80270_g1,c9047_g1,c1350_g1,c82300_g1,c2590_g1,c47300_g1,c107366_g1,c109080_g1,c98706_g1,c96722_g1,c50971_g1,c54435_g1,c102809_g1,c44181_g1,c40487_g2,c45909_g1,c112258_g1,c76641_g1,c71951_g1,c100052_g1,c57611_g1,c72454_g1,c12938_g1,c43714_g1,c42689_g1,c30097_g1,c12938_g2,c72147_g1,c85429_g1,c53274_g1,c105015_g1,c63436_g1,c28212_g1,c85122_g1,c73295_g1,c14476_g1,c11348_g1,c103738_g1,c50389_g1,c96233_g1,c65617_g1,c20347_g1,c104264_g1,c50568_g1,c64671_g1,c96341_g1,c45058_g2,c95064_g1,c95996_g1,c77182_g1,c77954_g1,c84626_g1,c94337_g1,c27368_g1,c104622_g1,c34293_g1,c55062_g1,c9500_g1,c101218_g1,c62850_g1,c45816_g1,c94313_g1,c17461_g1,c73923_g1,c113085_g1,c28468_g1,c97674_g1,c35770_g1,c78342_g1,c34815_g1,c105484_g1,c43501_g1,c3591_g1,c13264_g1,c1753_g1,c76720_g1,c53845_g1,c41308_g1,c88883_g1,c18075_g1,c45058_g1,c67775_g1,c9036_g1,c42698_g1,c84712_g1,c56202_g1,c34353_g1,c22770_g1,c6841_g1,c47589_g1,c102038_g1,c86703_g1,c95381_g1,c83077_g1,c52918_g1,c107010_g1,c84542_g1,c55638_g1,c34582_g1,c51145_g1,c24487_g1,c85369_g1,c94256_g1,c107567_g1,c38754_g1,c100234_g1,c41230_g1,c75332_g1,c13249_g1,c73468_g1,c93179_g1,c67666_g1,c45231_g1,c90617_g1,c58698_g1,c42703_g1,c47617_g1,c98996_g1,c93711_g1,c63265_g1,c61668_g1,c60287_g1,c56245_g1,c99974_g1,c52505_g1,c73877_g1,c49414_g1,c63989_g1 |
| alpha-Linolenic acid metabolism | ko00592 | 94 | c49837_g1,c48391_g4,c86204_g1,c83586_g1,c27642_g1,c99472_g1,c41514_g1,c27856_g1,c9364_g1,c41055_g1,c11144_g1,c48391_g2,c110024_g1,c48391_g1,c31403_g1,c47041_g1,c29052_g1,c42828_g1,c83786_g1,c35762_g1,c105841_g1,c52044_g2,c92391_g1,c104495_g1,c49861_g1,c85859_g1,c54787_g1,c109291_g1,c90317_g1,c104011_g1,c50443_g1,c16508_g1,c102397_g1,c34025_g2,c47261_g2,c50443_g2,c57773_g1,c53626_g1,c44808_g1,c1752_g1,c8771_g1,c108568_g1,c48719_g1,c45615_g1,c42337_g1,c109241_g1,c46912_g1,c6642_g1,c108175_g1,c46156_g1,c89579_g1,c40949_g1,c40146_g1,c6594_g1,c18036_g1,c91334_g1,c48391_g3,c6536_g1,c106336_g1,c88993_g1,c29520_g1,c64873_g1,c98768_g1,c74218_g1,c49082_g2,c48375_g1,c29399_g1,c72313_g1,c94438_g1,c93690_g1,c103892_g1,c96705_g1,c49355_g1,c16880_g1,c71698_g1,c81194_g1,c82737_g1,c107910_g1,c84221_g1,c15219_g1,c47063_g1,c43660_g1,c53628_g1,c78159_g1,c62236_g1,c33198_g1,c49096_g4,c105824_g1,c50051_g2,c25790_g1,c86544_g1,c34025_g1,c51828_g1,c26736_g1 |
| Amino sugar and nucleotide sugar metabolism | ko00520 | 175 | c39220_g1,c74497_g1,c35230_g1,c49729_g1,c50375_g1,c8202_g2,c45215_g1,c51294_g2,c26188_g1,c105174_g1,c67593_g1,c19647_g1,c50375_g2,c41978_g1,c6348_g1,c85940_g1,c94234_g1,c52203_g3,c46210_g1,c33732_g1,c67994_g1,c56675_g1,c97022_g1,c47534_g1,c17039_g1,c53549_g1,c50375_g3,c50835_g7,c17860_g1,c72656_g1,c110086_g1,c41726_g1,c71652_g1,c44699_g2,c56744_g1,c94683_g1,c101376_g1,c90856_g1,c44235_g1,c45394_g1,c71270_g1,c97593_g1,c99880_g1,c46166_g2,c38793_g1,c50004_g1,c25280_g1,c38097_g1,c24869_g1,c36333_g1,c64130_g1,c28160_g1,c9166_g1,c29352_g1,c99377_g1,c50835_g8,c98036_g1,c74356_g1,c72186_g1,c103350_g1,c42663_g1,c49211_g3,c77371_g1,c41342_g1,c74132_g1,c49506_g1,c49823_g1,c82676_g1,c81638_g1,c2020_g1,c83930_g1,c84191_g1,c43413_g1,c46831_g1,c71759_g1,c74204_g1,c4008_g1,c53986_g1,c100584_g1,c14572_g1,c100551_g1,c109158_g1,c46365_g1,c104082_g1,c61900_g1,c111846_g1,c41890_g1,c28265_g1,c42339_g1,c51294_g5,c93503_g1,c41805_g1,c1956_g1,c20019_g1,c55616_g1,c106097_g1,c42366_g1,c57149_g1,c109366_g1,c49781_g1,c53081_g1,c72705_g1,c106844_g1,c42520_g1,c48976_g1,c43212_g1,c62487_g1,c74035_g1,c56604_g1,c40754_g1,c71749_g1,c12768_g2,c46996_g1,c47216_g1,c97674_g1,c47544_g1,c62502_g1,c50236_g1,c57866_g1,c61219_g1,c8202_g1,c51294_g7,c48532_g1,c12481_g1,c44013_g1,c104211_g1,c42698_g1,c48071_g1,c44019_g2,c106393_g1,c46473_g1,c49525_g2,c49151_g1,c45030_g1,c13374_g1,c81861_g1,c13722_g1,c35846_g1,c94249_g1,c26165_g1,c110906_g1,c30673_g1,c55638_g1,c58536_g1,c31032_g1,c72127_g1,c46480_g1,c38396_g1,c50086_g1,c92816_g1,c103688_g1,c26258_g1,c89319_g1,c45825_g1,c73468_g1,c84412_g1,c61443_g1,c6455_g1,c54779_g1,c48185_g1,c13328_g1,c10950_g1,c55503_g1,c6425_g1,c52381_g3,c47095_g1,c52381_g2,c82886_g1,c29256_g2,c104237_g1,c49248_g1,c14439_g1,c71625_g1,c63024_g1,c93332_g1 |
| Aminobenzoate degradation | ko00627 | 45 | c62744_g1,c49461_g1,c44494_g1,c63072_g1,c52112_g1,c57881_g1,c52112_g2,c24471_g1,c54530_g1,c95509_g1,c52112_g3,c110831_g1,c11088_g1,c52941_g1,c54345_g1,c83889_g1,c25007_g2,c99792_g1,c108064_g1,c36408_g1,c46568_g1,c33474_g1,c43675_g2,c50878_g1,c82195_g1,c52985_g1,c41440_g1,c11795_g1,c2765_g1,c46140_g1,c46599_g1,c10651_g1,c25106_g1,c54005_g1,c71527_g1,c28775_g2,c10846_g1,c110641_g1,c37774_g1,c48317_g1,c71682_g1,c27078_g1,c41788_g1,c28775_g1,c82973_g1 |
| Anthocyanin biosynthesis | ko00942 | 3 | c47463_g2,c84151_g1,c36718_g1 |
| Arachidonic acid metabolism | ko00590 | 39 | c53891_g1,c72152_g1,c27228_g1,c41055_g1,c44921_g1,c28142_g1,c54761_g1,c33993_g1,c16419_g1,c103320_g1,c88571_g1,c77041_g1,c8401_g1,c96381_g1,c108341_g1,c19935_g2,c57277_g1,c42999_g1,c107064_g1,c47159_g1,c47901_g1,c5476_g1,c9569_g1,c100363_g1,c16959_g1,c43105_g1,c2706_g1,c40725_g1,c48922_g6,c1102_g1,c59619_g1,c94735_g1,c56694_g1,c21974_g1,c47159_g2,c49345_g2,c44400_g2,c52376_g1,c51828_g1 |
| Arginine and proline metabolism | ko00330 | 204 | c104558_g1,c82031_g1,c89219_g1,c46973_g1,c72088_g1,c110883_g1,c51800_g4,c46531_g1,c105115_g1,c91379_g1,c46714_g1,c72302_g1,c106588_g1,c24757_g1,c49462_g1,c30008_g1,c88947_g1,c47621_g1,c30038_g1,c48536_g2,c61356_g1,c35216_g1,c18319_g1,c101116_g1,c19206_g1,c110089_g1,c47365_g1,c80695_g1,c84932_g1,c86580_g1,c50774_g1,c59098_g1,c44498_g1,c40621_g1,c42270_g1,c48142_g1,c106670_g1,c77010_g1,c51781_g1,c18163_g1,c83834_g1,c95320_g1,c40766_g1,c8564_g1,c24284_g1,c80270_g1,c97539_g1,c75402_g1,c62190_g1,c47244_g1,c73811_g1,c43524_g1,c31507_g1,c9047_g1,c50854_g1,c42796_g1,c37859_g1,c1350_g1,c82300_g1,c48536_g3,c15654_g1,c109080_g1,c93433_g1,c34608_g1,c71607_g1,c101216_g1,c48863_g1,c88691_g1,c26212_g1,c48574_g1,c49843_g1,c102809_g1,c51479_g1,c85914_g1,c90984_g1,c44181_g1,c98558_g1,c45909_g1,c112258_g1,c76641_g1,c41057_g1,c55541_g1,c452_g1,c57611_g1,c97253_g1,c12938_g1,c43714_g1,c50235_g1,c71601_g1,c12938_g2,c87181_g1,c85429_g1,c75055_g1,c61823_g1,c53274_g1,c38395_g1,c28212_g1,c85122_g1,c105469_g1,c1888_g2,c55078_g1,c972_g1,c73295_g1,c50389_g1,c37014_g1,c87855_g1,c108108_g1,c93472_g1,c29553_g1,c77558_g1,c96233_g1,c46568_g1,c81417_g1,c93105_g1,c107580_g1,c50568_g1,c64671_g1,c62096_g1,c14697_g1,c49723_g1,c86658_g1,c105453_g1,c45058_g2,c18329_g1,c95064_g1,c34731_g1,c95996_g1,c52336_g2,c44223_g1,c10998_g1,c58316_g1,c6580_g1,c84626_g1,c104622_g1,c43752_g1,c45085_g1,c55062_g1,c95011_g1,c53312_g1,c77610_g1,c46985_g1,c45816_g1,c47479_g1,c46549_g1,c73923_g1,c25130_g1,c8465_g1,c102364_g1,c101624_g1,c99458_g1,c28468_g1,c71959_g1,c86870_g1,c96313_g1,c34815_g1,c66191_g1,c46599_g1,c43501_g1,c3591_g1,c41818_g1,c1753_g1,c54005_g1,c47012_g1,c26574_g1,c45058_g1,c28775_g2,c41580_g1,c56202_g1,c52635_g3,c22770_g1,c55060_g1,c54352_g1,c83077_g1,c62978_g1,c27216_g1,c51145_g1,c48317_g1,c38754_g1,c100234_g1,c41230_g1,c49460_g2,c76192_g1,c94496_g1,c72925_g1,c1398_g1,c45231_g1,c83915_g1,c89815_g1,c41057_g2,c90369_g1,c47617_g1,c93711_g1,c96110_g1,c28775_g1,c63265_g1,c61668_g1,c69864_g1,c53314_g1,c70358_g1,c72332_g1,c72685_g1,c18208_g1,c56839_g1,c42292_g1 |
| Ascorbate and aldarate metabolism | ko00053 | 90 | c89219_g1,c38643_g1,c44778_g1,c106784_g1,c47621_g1,c41536_g1,c48536_g2,c46582_g1,c35216_g1,c20519_g1,c109139_g1,c110089_g1,c47365_g1,c55094_g1,c82135_g1,c86580_g1,c47081_g1,c64519_g1,c106670_g1,c85892_g1,c40766_g1,c37159_g1,c94637_g1,c97539_g1,c75402_g1,c62190_g1,c47244_g1,c73811_g1,c25280_g1,c24869_g1,c48536_g3,c34608_g1,c38682_g1,c86689_g1,c99377_g1,c88691_g1,c41057_g1,c49211_g3,c55541_g1,c50235_g1,c75055_g1,c61823_g1,c105469_g1,c30928_g1,c45736_g1,c39602_g1,c29553_g1,c42323_g1,c25669_g1,c94688_g1,c111846_g1,c49723_g1,c95114_g1,c52336_g2,c58316_g1,c47484_g1,c45085_g1,c49031_g1,c34663_g1,c43212_g1,c74035_g1,c102364_g1,c46868_g1,c44778_g2,c49088_g1,c44395_g1,c82960_g1,c73183_g1,c37410_g1,c54113_g1,c50400_g2,c62978_g1,c99066_g1,c33518_g1,c35846_g1,c26165_g1,c44060_g1,c50086_g1,c92816_g1,c26258_g1,c76192_g1,c1398_g1,c83915_g1,c89815_g1,c41057_g2,c90369_g1,c45355_g1,c5325_g1,c49031_g3,c50646_g1 |
| Benzoate degradation | ko00362 | 29 | c65424_g1,c48902_g1,c27642_g1,c99472_g1,c15296_g1,c14782_g1,c11144_g1,c77190_g1,c80976_g1,c54787_g1,c75123_g1,c53626_g1,c64933_g1,c42337_g1,c46156_g1,c6594_g1,c91334_g1,c103615_g1,c6536_g1,c57934_g1,c49065_g1,c93690_g1,c103892_g1,c37822_g1,c82737_g1,c72872_g1,c43660_g1,c84599_g1,c31759_g1 |
| beta-Alanine metabolism | ko00410 | 171 | c52678_g2,c8198_g1,c45383_g1,c89219_g1,c94070_g1,c46531_g1,c44302_g2,c35789_g1,c11497_g1,c47621_g1,c83165_g1,c30038_g1,c9421_g1,c13346_g1,c48536_g2,c35216_g1,c52533_g4,c18319_g1,c52798_g4,c62877_g1,c110089_g1,c77947_g1,c726_g1,c17463_g1,c47365_g1,c13175_g1,c37769_g1,c52818_g1,c47185_g1,c86580_g1,c47420_g1,c18801_g1,c69486_g1,c92658_g1,c46822_g1,c48142_g1,c82486_g1,c106670_g1,c77010_g1,c43009_g2,c81982_g1,c85139_g1,c52729_g1,c66882_g1,c40766_g1,c50954_g5,c99712_g1,c112308_g1,c49366_g9,c47384_g1,c89320_g1,c97539_g1,c75402_g1,c62190_g1,c47244_g1,c73811_g1,c50870_g1,c78560_g1,c2590_g1,c48536_g3,c34608_g1,c52061_g2,c4317_g1,c50971_g1,c91515_g1,c39032_g1,c88691_g1,c33831_g1,c103399_g1,c36513_g1,c39997_g1,c1906_g1,c48257_g1,c78330_g1,c56783_g1,c12050_g1,c109689_g1,c41057_g1,c55541_g1,c27943_g1,c27323_g1,c12162_g1,c27619_g1,c30097_g1,c50235_g1,c83324_g1,c50796_g9,c36907_g1,c50954_g2,c75055_g1,c61823_g1,c105469_g1,c50584_g1,c96322_g1,c972_g1,c58116_g1,c48999_g1,c46948_g1,c29553_g1,c43444_g2,c49366_g5,c100270_g1,c65617_g1,c98660_g1,c85135_g1,c106416_g1,c45230_g1,c93748_g1,c49723_g1,c34317_g1,c40850_g1,c34731_g1,c52336_g2,c58316_g1,c72626_g1,c30815_g1,c45085_g1,c77787_g1,c54776_g1,c52434_g1,c102459_g1,c52030_g1,c84199_g1,c460_g1,c94313_g1,c85240_g1,c102364_g1,c27356_g1,c4317_g2,c52858_g6,c102188_g1,c25261_g1,c44681_g1,c56741_g1,c103084_g1,c29591_g1,c36340_g1,c34019_g1,c18177_g1,c39207_g1,c46277_g1,c34353_g1,c111270_g1,c62978_g1,c52918_g1,c47598_g1,c42128_g1,c48105_g1,c48882_g1,c27216_g1,c52271_g2,c47618_g1,c83437_g1,c76192_g1,c72925_g1,c1398_g1,c52466_g1,c49366_g1,c83915_g1,c89815_g1,c41057_g2,c90369_g1,c41124_g1,c110160_g1,c48230_g1,c46775_g1,c51499_g2,c62283_g1,c32423_g1,c51868_g1,c50316_g1 |
| Betalain biosynthesis | ko00965 | 6 | c54456_g1,c95214_g1,c94472_g1,c82743_g1,c68373_g1,c44570_g1 |
| Biosynthesis of amino acids | ko01230 | 636 | c104558_g1,c109984_g1,c76030_g1,c104137_g1,c44170_g1,c82031_g1,c45645_g1,c38356_g1,c41857_g1,c88999_g1,c6029_g1,c57311_g1,c72088_g1,c11263_g1,c110883_g1,c52441_g2,c51800_g4,c61635_g1,c96286_g1,c105115_g1,c91379_g1,c42733_g1,c92695_g1,c74905_g1,c47400_g1,c88604_g1,c95034_g1,c42740_g1,c56073_g1,c80465_g1,c48127_g2,c73106_g1,c46874_g1,c8857_g1,c106588_g1,c105979_g1,c96551_g1,c39222_g1,c49462_g1,c46281_g1,c96407_g1,c104210_g1,c88947_g1,c63776_g1,c52927_g1,c68591_g1,c31636_g1,c16964_g1,c86188_g1,c73942_g1,c101256_g1,c71149_g1,c868_g1,c47044_g1,c48844_g2,c42988_g1,c48533_g1,c100006_g1,c35216_g1,c40311_g1,c59715_g1,c53966_g1,c79442_g1,c45381_g1,c5249_g1,c100050_g1,c105912_g1,c101116_g1,c57794_g1,c19206_g1,c46404_g4,c25123_g2,c60397_g1,c36407_g1,c95068_g1,c59201_g1,c110089_g1,c93494_g1,c27203_g1,c24934_g1,c50882_g1,c98311_g1,c73078_g1,c45680_g1,c25404_g1,c52344_g5,c80695_g1,c80123_g1,c27600_g1,c50179_g2,c73578_g1,c37292_g1,c77184_g1,c48094_g1,c98363_g1,c94505_g1,c96933_g1,c59098_g1,c42784_g1,c103393_g1,c97967_g1,c40621_g1,c98388_g1,c46404_g1,c103747_g1,c90205_g1,c110039_g1,c105038_g1,c19297_g1,c48970_g1,c21303_g1,c42027_g1,c49559_g2,c53135_g1,c52959_g1,c47688_g1,c3557_g1,c32971_g1,c105628_g1,c100459_g1,c61871_g1,c16496_g1,c37799_g1,c17536_g2,c55416_g1,c104105_g1,c51781_g1,c84795_g1,c83241_g1,c18163_g1,c44028_g1,c94230_g1,c945_g1,c76102_g1,c19834_g1,c75603_g1,c95320_g1,c8564_g1,c110385_g1,c107161_g1,c14450_g1,c66158_g1,c38070_g1,c45803_g1,c24284_g1,c29684_g1,c66611_g1,c80270_g1,c64213_g1,c67039_g1,c49098_g3,c72023_g1,c43427_g1,c73282_g1,c47244_g1,c18147_g1,c82202_g1,c97094_g1,c67769_g1,c12390_g1,c76255_g1,c43677_g1,c106435_g1,c96344_g1,c48427_g1,c47003_g1,c72006_g1,c50115_g1,c46151_g1,c31507_g1,c110541_g1,c34733_g1,c93137_g1,c49757_g3,c50854_g1,c1350_g1,c42785_g1,c945_g2,c53873_g1,c51735_g1,c41831_g1,c83854_g1,c109012_g1,c61242_g1,c89066_g1,c100063_g1,c84258_g1,c15654_g1,c104310_g1,c109080_g1,c99893_g1,c93433_g1,c71092_g1,c34281_g1,c26218_g1,c14545_g1,c98299_g1,c70597_g1,c71607_g1,c108530_g1,c98706_g1,c106159_g1,c6653_g2,c51658_g3,c87086_g1,c51128_g1,c97409_g1,c68919_g1,c66195_g1,c12471_g1,c42133_g1,c38169_g1,c65042_g1,c38897_g1,c23993_g1,c76028_g1,c112630_g1,c101216_g1,c93988_g1,c47668_g1,c47044_g2,c87593_g1,c60359_g1,c48863_g1,c48574_g1,c100842_g1,c25123_g1,c20257_g1,c19508_g1,c49599_g1,c81098_g1,c6653_g1,c102809_g1,c51479_g1,c85914_g1,c75960_g1,c29286_g1,c44181_g1,c85117_g1,c48132_g2,c112258_g1,c76641_g1,c64297_g1,c81385_g1,c108398_g1,c50019_g1,c56971_g1,c87608_g1,c44849_g2,c53308_g1,c103573_g1,c100052_g1,c55541_g1,c452_g1,c103120_g1,c47283_g1,c109939_g1,c64274_g1,c57611_g1,c42239_g1,c45885_g1,c97253_g1,c8176_g2,c77583_g1,c12938_g1,c72500_g1,c24536_g1,c35032_g1,c106540_g1,c29316_g1,c104555_g1,c43714_g1,c47900_g1,c4149_g1,c78386_g1,c45119_g1,c72940_g1,c100120_g1,c12056_g1,c82149_g1,c93338_g1,c71601_g1,c105807_g1,c79946_g1,c12938_g2,c84114_g1,c41255_g1,c47074_g1,c83062_g1,c47900_g2,c85429_g1,c109380_g1,c9388_g1,c53274_g1,c9362_g1,c39848_g1,c63436_g1,c27198_g1,c40802_g1,c28212_g1,c85122_g1,c52882_g1,c105469_g1,c53631_g1,c48823_g1,c98474_g1,c14995_g1,c1888_g2,c66239_g1,c48370_g1,c55078_g1,c95141_g1,c50526_g2,c49207_g1,c105051_g1,c78544_g1,c93038_g1,c73295_g1,c14476_g1,c44012_g1,c35188_g1,c91964_g1,c11348_g1,c50389_g1,c16638_g1,c10908_g1,c108108_g1,c74727_g1,c57387_g1,c93472_g1,c97209_g1,c85195_g1,c71900_g1,c34013_g1,c44349_g1,c25225_g1,c104009_g1,c100202_g1,c54743_g1,c81417_g1,c66906_g1,c93181_g1,c93189_g1,c54211_g1,c108018_g1,c15006_g1,c48894_g1,c44734_g1,c82781_g1,c1939_g1,c32058_g1,c54222_g1,c50868_g1,c101915_g1,c41819_g1,c54175_g1,c48887_g1,c93105_g1,c83928_g1,c45500_g1,c58532_g1,c50150_g1,c79123_g1,c104264_g1,c3870_g1,c50568_g1,c101267_g1,c8863_g1,c43644_g1,c66141_g1,c48310_g1,c79843_g1,c64671_g1,c62096_g1,c73269_g1,c51583_g1,c77408_g1,c107446_g1,c48887_g2,c48879_g1,c86658_g1,c34561_g1,c105453_g1,c69093_g1,c65408_g1,c18329_g1,c95064_g1,c95996_g1,c51740_g1,c82523_g1,c43327_g1,c69203_g1,c57349_g1,c84626_g1,c98122_g1,c87783_g1,c104622_g1,c45923_g1,c66337_g1,c39352_g1,c13402_g1,c78598_g1,c55062_g1,c100312_g1,c52622_g3,c9286_g1,c95011_g1,c87345_g1,c35331_g1,c42250_g1,c56114_g1,c88808_g1,c66749_g1,c84138_g1,c48628_g1,c9043_g1,c9285_g2,c82679_g1,c48427_g2,c33875_g1,c38803_g1,c67249_g1,c62780_g1,c26628_g1,c98277_g1,c12358_g1,c102419_g1,c66208_g1,c63367_g1,c99539_g1,c77610_g1,c79871_g1,c18541_g1,c25409_g1,c63275_g1,c45816_g1,c47479_g1,c17461_g1,c104877_g1,c99612_g1,c53687_g1,c73923_g1,c42918_g1,c50526_g3,c87748_g1,c45425_g1,c41338_g1,c99458_g1,c29716_g1,c52622_g2,c12036_g1,c46700_g1,c50699_g1,c28468_g1,c79133_g1,c31470_g1,c17869_g1,c49306_g2,c35770_g1,c61163_g1,c104829_g1,c34116_g1,c71959_g1,c43880_g2,c95283_g1,c34815_g1,c75192_g1,c44377_g1,c66191_g1,c58823_g1,c43501_g1,c3591_g1,c13264_g1,c19449_g1,c78700_g1,c43737_g1,c41818_g1,c4459_g1,c104455_g1,c45327_g2,c54124_g1,c39440_g1,c1753_g1,c9921_g1,c103322_g1,c43115_g1,c89336_g1,c72220_g1,c12516_g1,c101370_g1,c109884_g1,c21011_g1,c46556_g1,c85745_g1,c61619_g1,c54565_g1,c42387_g1,c96844_g1,c44832_g2,c28291_g1,c16761_g1,c26363_g1,c76059_g1,c82144_g1,c77042_g1,c20257_g2,c41580_g1,c9036_g1,c56892_g1,c56202_g1,c67532_g1,c46404_g3,c14803_g1,c3742_g1,c49242_g1,c55154_g1,c45327_g3,c24600_g1,c61298_g1,c48266_g1,c71604_g1,c62156_g1,c55060_g1,c50016_g1,c15887_g1,c97042_g1,c63898_g1,c38946_g1,c95977_g1,c106754_g1,c94269_g1,c107010_g1,c43427_g2,c40769_g1,c70771_g1,c112079_g1,c72032_g1,c61407_g1,c92958_g1,c28032_g1,c101270_g1,c29000_g1,c36681_g1,c43595_g1,c65758_g1,c38423_g1,c49174_g1,c67501_g1,c41230_g1,c55637_g1,c48708_g1,c99146_g1,c27664_g1,c108002_g1,c59882_g1,c43733_g1,c45960_g1,c57820_g1,c72642_g1,c74256_g1,c5988_g1,c94496_g1,c13249_g1,c68678_g1,c83296_g1,c86327_g1,c95128_g1,c58199_g1,c96465_g1,c93179_g1,c37431_g1,c73090_g1,c79025_g1,c45648_g1,c67666_g1,c82324_g1,c31740_g1,c48449_g1,c76091_g1,c58698_g1,c61234_g1,c43722_g1,c41423_g1,c110574_g1,c29198_g1,c44203_g1,c93711_g1,c56269_g1,c26284_g1,c11162_g1,c64536_g1,c45852_g1,c58356_g1,c38735_g1,c96110_g1,c47115_g1,c63265_g1,c51974_g1,c45838_g1,c106552_g1,c40283_g2,c9318_g1,c61668_g1,c58304_g1,c43454_g1,c59464_g1,c69864_g1,c39238_g1,c46404_g2,c94570_g1,c85823_g1,c89702_g1,c60287_g1,c70358_g1,c46331_g1,c74521_g1,c74030_g1,c87107_g1,c72141_g1,c63465_g1,c15105_g1,c49460_g3,c48624_g1,c47551_g1,c35146_g1,c52505_g1,c46851_g1,c72332_g1,c43883_g1,c72685_g1,c48310_g2,c49356_g1,c54230_g1,c112461_g1,c76988_g1,c45578_g2 |
| Biosynthesis of ansamycins | ko01051 | 15 | c68591_g1,c76102_g1,c72023_g1,c6653_g2,c12471_g1,c6653_g1,c93181_g1,c34561_g1,c65408_g1,c35331_g1,c87748_g1,c55637_g1,c99146_g1,c76091_g1,c15105_g1 |
| Biosynthesis of siderophore group nonribosomal peptides | ko01053 | 1 | c51088_g1 |
| Biosynthesis of unsaturated fatty acids | ko01040 | 94 | c51048_g1,c91648_g1,c45903_g1,c85912_g1,c36972_g1,c83586_g1,c27642_g1,c7453_g1,c99472_g1,c62088_g1,c5941_g1,c27856_g1,c42039_g1,c97175_g1,c11144_g1,c43519_g1,c108605_g1,c71720_g1,c20142_g2,c31092_g1,c31403_g1,c105584_g1,c37502_g1,c74426_g1,c5104_g1,c103085_g1,c95324_g1,c19291_g1,c83786_g1,c13150_g1,c44731_g1,c105841_g1,c71973_g1,c61794_g1,c52044_g2,c104182_g1,c42039_g2,c85859_g1,c54787_g1,c104011_g1,c89210_g1,c64939_g1,c43130_g1,c35017_g1,c79285_g1,c57773_g1,c105187_g1,c53626_g1,c90027_g1,c50317_g6,c107391_g1,c42337_g1,c46912_g1,c108175_g1,c46156_g1,c50317_g1,c6594_g1,c91334_g1,c71636_g1,c64756_g1,c61705_g1,c66424_g1,c6536_g1,c47306_g1,c92834_g1,c45693_g1,c44583_g1,c64873_g1,c98768_g1,c8801_g1,c61703_g1,c15679_g1,c29969_g1,c66721_g1,c105645_g1,c103310_g1,c96486_g1,c29399_g1,c31025_g1,c31829_g1,c93690_g1,c103892_g1,c53357_g1,c71698_g1,c82737_g1,c50506_g1,c43660_g1,c12646_g1,c76322_g1,c57537_g1,c63304_g1,c86544_g1,c41393_g1,c26736_g1 |
| Biotin metabolism | ko00780 | 47 | c91648_g1,c9199_g1,c7453_g1,c62088_g1,c97175_g1,c46917_g2,c49563_g1,c105584_g1,c74426_g1,c47661_g1,c5104_g1,c111491_g1,c98327_g1,c62015_g1,c110431_g1,c34254_g1,c42227_g1,c13150_g1,c75476_g1,c61794_g1,c70483_g1,c84896_g1,c43130_g1,c79285_g1,c76513_g1,c49775_g1,c107391_g1,c6706_g1,c16700_g2,c75495_g1,c61705_g1,c37035_g1,c4175_g1,c42121_g1,c15679_g1,c29969_g1,c66721_g1,c11363_g1,c45858_g1,c105645_g1,c53679_g1,c74592_g1,c31829_g1,c2198_g1,c50506_g1,c97941_g1,c97788_g1 |
| Bisphenol degradation | ko00363 | 38 | c62744_g1,c49461_g1,c44494_g1,c63072_g1,c52112_g1,c57881_g1,c52112_g2,c24471_g1,c54530_g1,c95509_g1,c52112_g3,c110831_g1,c11088_g1,c52941_g1,c54345_g1,c83889_g1,c25007_g2,c99792_g1,c108064_g1,c36408_g1,c33474_g1,c43675_g2,c50878_g1,c82195_g1,c52985_g1,c11795_g1,c2765_g1,c46140_g1,c10651_g1,c25106_g1,c71527_g1,c10846_g1,c110641_g1,c37774_g1,c71682_g1,c27078_g1,c41788_g1,c82973_g1 |
| Brassinosteroid biosynthesis | ko00905 | 21 | c53634_g1,c47881_g1,c39749_g1,c4543_g1,c39525_g1,c530_g1,c40867_g1,c47420_g2,c87710_g1,c25481_g1,c51006_g1,c47981_g2,c109597_g1,c105913_g1,c101524_g1,c51106_g7,c4116_g1,c68549_g1,c42019_g1,c94262_g1,c100747_g1 |
| Butanoate metabolism | ko00650 | 71 | c45383_g1,c65424_g1,c18220_g1,c6029_g1,c95034_g1,c68570_g1,c83165_g1,c16964_g1,c48902_g1,c65889_g1,c95068_g1,c15296_g1,c12659_g1,c14782_g1,c105038_g1,c110441_g1,c73821_g1,c92986_g1,c2590_g1,c77190_g1,c61242_g1,c80976_g1,c66195_g1,c50971_g1,c64274_g1,c75123_g1,c104555_g1,c30097_g1,c93338_g1,c38127_g2,c57634_g1,c66239_g1,c74727_g1,c64933_g1,c65617_g1,c14188_g1,c103615_g1,c104126_g1,c87783_g1,c34293_g1,c9286_g1,c79501_g1,c67249_g1,c62850_g1,c94313_g1,c57934_g1,c94166_g1,c49065_g1,c82596_g1,c105484_g1,c10482_g1,c107241_g1,c34353_g1,c86703_g1,c52918_g1,c52901_g1,c34582_g1,c37822_g1,c94256_g1,c107421_g1,c72872_g1,c72642_g1,c86327_g1,c48449_g1,c84599_g1,c98996_g1,c4023_g1,c54704_g1,c38496_g1,c31759_g1,c39018_g1 |
| Butirosin and neomycin biosynthesis | ko00524 | 7 | c6348_g1,c67994_g1,c100584_g1,c50236_g1,c30673_g1,c52381_g3,c52381_g2 |
| C5-Branched dibasic acid metabolism | ko00660 | 31 | c6029_g1,c11263_g1,c95034_g1,c16964_g1,c95068_g1,c94505_g1,c105038_g1,c32971_g1,c67769_g1,c49757_g3,c61242_g1,c66195_g1,c64274_g1,c104555_g1,c93338_g1,c66239_g1,c78544_g1,c74727_g1,c48894_g1,c87783_g1,c9286_g1,c67249_g1,c104877_g1,c3742_g1,c70771_g1,c57820_g1,c72642_g1,c86327_g1,c48449_g1,c64536_g1,c47551_g1 |
| Caffeine metabolism | ko00232 | 17 | c54289_g1,c109324_g1,c33816_g1,c77055_g1,c72989_g1,c106149_g1,c788_g1,c95319_g1,c52493_g1,c53666_g1,c39493_g1,c81878_g1,c63587_g1,c105775_g1,c59078_g1,c47893_g1,c68744_g1 |
| Caprolactam degradation | ko00930 | 7 | c65676_g1,c82728_g1,c109882_g1,c71745_g1,c61957_g1,c107826_g1,c33398_g1 |
| Carbon fixation in photosynthetic organisms | ko00710 | 177 | c109984_g1,c72144_g1,c104137_g1,c12991_g1,c110554_g1,c45645_g1,c18240_g1,c41857_g1,c55505_g1,c110883_g1,c45370_g1,c14873_g1,c46874_g1,c106588_g1,c73851_g1,c102864_g1,c39222_g1,c104210_g1,c68591_g1,c101256_g1,c47044_g1,c17506_g1,c100006_g1,c5249_g1,c57794_g1,c93517_g1,c80695_g1,c48094_g1,c98388_g1,c103747_g1,c48970_g1,c49559_g2,c53135_g1,c47688_g1,c16496_g1,c76146_g1,c44028_g1,c76102_g1,c107161_g1,c80270_g1,c64213_g1,c72023_g1,c43427_g1,c82202_g1,c76255_g1,c40278_g1,c72006_g1,c99582_g1,c1350_g1,c25743_g1,c104310_g1,c70719_g1,c34281_g1,c48397_g1,c6653_g2,c51658_g3,c12471_g1,c40011_g2,c47044_g2,c26_g2,c6653_g1,c74796_g1,c37201_g1,c49196_g1,c41499_g1,c87608_g1,c52142_g1,c109939_g1,c57611_g1,c12938_g1,c82149_g1,c12938_g2,c84114_g1,c85447_g1,c93721_g1,c63436_g1,c27198_g1,c40802_g1,c28212_g1,c52882_g1,c95141_g1,c43166_g1,c11153_g1,c49207_g1,c62375_g1,c75440_g1,c11348_g1,c41865_g1,c107227_g1,c50389_g1,c54743_g1,c93181_g1,c44734_g1,c54222_g1,c54175_g1,c49902_g2,c38853_g1,c43644_g1,c48310_g1,c36720_g1,c42775_g1,c34561_g1,c104067_g1,c69093_g1,c65408_g1,c99736_g1,c54243_g1,c84626_g1,c104622_g1,c29224_g1,c35331_g1,c71923_g1,c33875_g1,c99539_g1,c18541_g1,c25409_g1,c63275_g1,c99612_g1,c57038_g1,c73923_g1,c45902_g2,c61150_g1,c87748_g1,c45578_g1,c41338_g1,c12036_g1,c35770_g1,c64693_g1,c43880_g2,c101332_g1,c43501_g1,c11851_g1,c51142_g1,c49091_g1,c49902_g1,c1753_g1,c50458_g1,c85745_g1,c28291_g1,c26_g1,c24600_g1,c18302_g1,c40011_g1,c63898_g1,c43427_g2,c48655_g1,c61032_g1,c15666_g1,c52142_g2,c74712_g1,c49174_g1,c55637_g1,c99146_g1,c1642_g1,c47889_g1,c78499_g1,c68678_g1,c48827_g1,c37431_g1,c67666_g1,c82324_g1,c76091_g1,c107026_g1,c42807_g1,c99594_g1,c29075_g1,c47115_g1,c63265_g1,c47818_g1,c78175_g1,c94570_g1,c66235_g1,c15105_g1,c79094_g1,c43883_g1,c48310_g2,c13770_g1 |
| Carbon fixation pathways in prokaryotes | ko00720 | 139 | c110554_g1,c78806_g1,c47875_g1,c64170_g1,c109633_g1,c94923_g1,c88604_g1,c105979_g1,c73851_g1,c96407_g1,c9460_g1,c52927_g1,c48902_g1,c63195_g1,c17506_g1,c68302_g1,c98311_g1,c37292_g1,c103393_g1,c65074_g1,c110039_g1,c21303_g1,c52959_g1,c51231_g1,c88843_g1,c99669_g1,c58217_g1,c84795_g1,c86261_g1,c9412_g1,c945_g1,c110385_g1,c31007_g1,c36049_g1,c72878_g1,c9460_g2,c47937_g2,c93137_g1,c67728_g1,c42785_g1,c945_g2,c77190_g1,c66879_g1,c89066_g1,c85100_g1,c92836_g1,c70719_g1,c71092_g1,c48397_g1,c80976_g1,c97409_g1,c42133_g1,c88505_g1,c100842_g1,c20532_g1,c19508_g1,c75960_g1,c108398_g1,c53308_g1,c85718_g1,c75123_g1,c4149_g1,c100120_g1,c581_g2,c99342_g1,c837_g1,c85941_g1,c9388_g1,c53631_g1,c52404_g4,c10908_g1,c64933_g1,c49145_g1,c100202_g1,c66906_g1,c48887_g1,c101267_g1,c51583_g1,c48887_g2,c42775_g1,c103615_g1,c67839_g1,c67810_g1,c82477_g1,c86489_g1,c47635_g1,c24897_g1,c29224_g1,c50138_g2,c84138_g1,c62780_g1,c26628_g1,c102419_g1,c1434_g1,c37085_g1,c93775_g1,c49691_g1,c92573_g1,c46119_g1,c99135_g1,c64693_g1,c104284_g1,c49091_g1,c67546_g1,c89336_g1,c50458_g1,c581_g1,c53386_g1,c54565_g1,c16761_g1,c62374_g1,c78609_g1,c71604_g1,c58779_g1,c96911_g1,c77164_g1,c37822_g1,c28032_g1,c52291_g4,c62466_g1,c65758_g1,c72872_g1,c67246_g1,c111518_g1,c46544_g1,c54443_g1,c59896_g1,c97398_g1,c5988_g1,c46119_g2,c79025_g1,c76864_g1,c43722_g1,c84830_g1,c88093_g1,c83975_g1,c78175_g1,c31759_g1,c104506_g1 |
| Carbon metabolism | ko01200 | 763 | c48241_g1,c109984_g1,c19122_g1,c52678_g2,c72144_g1,c8198_g1,c104137_g1,c9293_g1,c12991_g1,c44170_g1,c110554_g1,c53491_g1,c45645_g1,c38356_g1,c18240_g1,c41857_g1,c29194_g1,c76954_g1,c55505_g1,c88999_g1,c110876_g1,c71672_g1,c110883_g1,c45370_g1,c94070_g1,c57341_g1,c65199_g1,c46994_g1,c74805_g1,c14873_g1,c77515_g1,c47875_g1,c52428_g1,c64170_g1,c109633_g1,c8202_g2,c94923_g1,c88604_g1,c11497_g1,c48127_g2,c51081_g1,c46874_g1,c8857_g1,c106588_g1,c105979_g1,c73851_g1,c96551_g1,c102864_g1,c38078_g1,c39222_g1,c46281_g1,c96407_g1,c104210_g1,c9460_g1,c52927_g1,c68591_g1,c31636_g1,c9421_g1,c101256_g1,c6348_g1,c48902_g1,c85940_g1,c47044_g1,c48844_g2,c42988_g1,c13346_g1,c48533_g1,c17506_g1,c96653_g1,c45979_g1,c100006_g1,c65889_g1,c68302_g1,c59715_g1,c59059_g1,c52533_g4,c5249_g1,c100050_g1,c52798_g4,c62877_g1,c57794_g1,c93517_g1,c11910_g1,c46404_g4,c25123_g2,c59201_g1,c27203_g1,c24934_g1,c77947_g1,c98311_g1,c726_g1,c73078_g1,c65926_g1,c67994_g1,c52344_g5,c37769_g1,c71859_g1,c52818_g1,c80695_g1,c27600_g1,c92790_g1,c76596_g1,c50179_g2,c55308_g1,c56675_g1,c37292_g1,c77184_g1,c48094_g1,c47420_g1,c98363_g1,c17039_g1,c96933_g1,c18801_g1,c103393_g1,c69486_g1,c100948_g1,c95742_g1,c65074_g1,c98388_g1,c66648_g1,c11271_g1,c46404_g1,c103747_g1,c90205_g1,c110039_g1,c19297_g1,c92658_g1,c48970_g1,c21303_g1,c49559_g2,c53135_g1,c68793_g1,c52959_g1,c47688_g1,c42135_g1,c45242_g1,c3557_g1,c51231_g1,c99904_g1,c61871_g1,c16496_g1,c37799_g1,c88843_g1,c76146_g1,c43009_g2,c81982_g1,c85139_g1,c99669_g1,c58217_g1,c84795_g1,c44028_g1,c52729_g1,c94230_g1,c86423_g1,c84428_g1,c95469_g1,c42371_g1,c945_g1,c84047_g1,c76102_g1,c73958_g1,c110385_g1,c50954_g5,c107161_g1,c108204_g1,c55130_g1,c99712_g1,c14450_g1,c49366_g9,c31007_g1,c47384_g1,c71978_g1,c68666_g1,c89741_g1,c36049_g1,c89320_g1,c99880_g1,c29684_g1,c72878_g1,c80270_g1,c64213_g1,c38793_g1,c72023_g1,c43427_g1,c9747_g1,c9460_g2,c18147_g1,c82202_g1,c97094_g1,c76255_g1,c106435_g1,c40278_g1,c96344_g1,c48427_g1,c78140_g1,c72006_g1,c47937_g2,c68426_g1,c43637_g1,c99582_g1,c46151_g1,c976_g1,c110541_g1,c34733_g1,c93137_g1,c67728_g1,c93937_g1,c1350_g1,c42785_g1,c945_g2,c53873_g1,c51735_g1,c41831_g1,c83854_g1,c77190_g1,c109012_g1,c25743_g1,c107366_g1,c66879_g1,c89066_g1,c64130_g1,c85100_g1,c112656_g1,c104310_g1,c92836_g1,c70719_g1,c99893_g1,c71092_g1,c34281_g1,c26218_g1,c47022_g1,c28542_g2,c108530_g1,c48397_g1,c80976_g1,c6653_g2,c51658_g3,c97409_g1,c52061_g2,c68919_g1,c74445_g1,c12471_g1,c42133_g1,c78259_g1,c40011_g2,c38169_g1,c109486_g1,c38897_g1,c10937_g1,c76028_g1,c91515_g1,c67729_g1,c39032_g1,c47668_g1,c47044_g2,c61692_g1,c60359_g1,c103950_g1,c109587_g1,c88505_g1,c100842_g1,c33831_g1,c25123_g1,c81276_g1,c20257_g1,c19508_g1,c67587_g1,c72186_g1,c26_g2,c81098_g1,c6653_g1,c75960_g1,c1906_g1,c74796_g1,c54418_g1,c48257_g1,c85117_g1,c78330_g1,c37201_g1,c48132_g2,c74924_g1,c46898_g1,c82112_g1,c49196_g1,c56845_g1,c81385_g1,c108398_g1,c56971_g1,c41499_g1,c12050_g1,c109689_g1,c87608_g1,c23285_g1,c75473_g1,c53308_g1,c103573_g1,c77371_g1,c103120_g1,c27943_g1,c27323_g1,c83757_g1,c52142_g1,c47283_g1,c109939_g1,c57611_g1,c42239_g1,c45885_g1,c22216_g1,c28352_g1,c8176_g2,c12938_g1,c75123_g1,c106540_g1,c47900_g1,c4149_g1,c78386_g1,c12162_g1,c72940_g1,c100120_g1,c27619_g1,c82149_g1,c79946_g1,c12938_g2,c84114_g1,c83324_g1,c581_g2,c99342_g1,c41255_g1,c85447_g1,c50796_g9,c36907_g1,c837_g1,c47074_g1,c50954_g2,c65414_g1,c83062_g1,c82266_g1,c77688_g1,c47900_g2,c93721_g1,c109380_g1,c85941_g1,c75573_g1,c9388_g1,c38127_g2,c39848_g1,c63436_g1,c27198_g1,c40802_g1,c28212_g1,c74668_g1,c52882_g1,c53631_g1,c48823_g1,c14995_g1,c19122_g2,c108090_g1,c71759_g1,c95141_g1,c43166_g1,c50584_g1,c11153_g1,c49207_g1,c62375_g1,c96322_g1,c87186_g1,c58116_g1,c44012_g1,c65913_g1,c75440_g1,c66953_g1,c10611_g1,c11348_g1,c41865_g1,c48999_g1,c107227_g1,c13859_g1,c50389_g1,c16638_g1,c10908_g1,c46948_g1,c24562_g1,c64933_g1,c57387_g1,c85195_g1,c29152_g1,c87592_g1,c44349_g1,c43444_g2,c100584_g1,c49366_g5,c81440_g1,c25225_g1,c49145_g1,c98660_g1,c78345_g1,c100202_g1,c54743_g1,c94165_g1,c50326_g1,c66906_g1,c93181_g1,c54211_g1,c36510_g1,c92052_g1,c15006_g1,c40100_g1,c21888_g1,c44734_g1,c1939_g1,c85135_g1,c21314_g1,c54222_g1,c55393_g1,c50868_g1,c101352_g1,c41819_g1,c54175_g1,c48887_g1,c45500_g1,c106416_g1,c58532_g1,c49902_g2,c38853_g1,c93748_g1,c101267_g1,c28265_g1,c43644_g1,c108664_g1,c48310_g1,c79843_g1,c36720_g1,c51583_g1,c26866_g1,c48887_g2,c42775_g1,c34561_g1,c34317_g1,c104067_g1,c107473_g1,c69093_g1,c65408_g1,c103615_g1,c84312_g1,c67839_g1,c68624_g1,c107669_g1,c82523_g1,c82477_g1,c86489_g1,c99736_g1,c75511_g1,c54243_g1,c57349_g1,c99079_g1,c47635_g1,c84626_g1,c33782_g2,c46725_g1,c75696_g1,c98122_g1,c104622_g1,c57149_g1,c30815_g1,c39352_g1,c61561_g1,c109895_g1,c29224_g1,c88902_g1,c50138_g2,c77787_g1,c90146_g1,c54776_g1,c52434_g1,c35331_g1,c102459_g1,c52030_g1,c71923_g1,c989_g1,c79501_g1,c74433_g1,c66749_g1,c84138_g1,c84199_g1,c9043_g1,c9285_g2,c82679_g1,c460_g1,c48427_g2,c33875_g1,c19122_g3,c38803_g1,c62780_g1,c26628_g1,c98277_g1,c57042_g1,c12358_g1,c102419_g1,c66208_g1,c63367_g1,c99539_g1,c51888_g3,c53329_g1,c18541_g1,c1434_g1,c25409_g1,c63275_g1,c3998_g1,c14937_g1,c99612_g1,c53687_g1,c57038_g1,c73923_g1,c45902_g2,c56604_g1,c61150_g1,c87748_g1,c93775_g1,c30117_g1,c45578_g1,c45425_g1,c85240_g1,c41338_g1,c27356_g1,c49691_g1,c12036_g1,c92573_g1,c46119_g1,c52858_g6,c102188_g1,c99135_g1,c79133_g1,c25261_g1,c44229_g1,c31470_g1,c44681_g1,c17869_g1,c35770_g1,c62502_g1,c104829_g1,c50236_g1,c48920_g1,c64693_g1,c43880_g2,c101332_g1,c44377_g1,c100365_g1,c29591_g1,c58823_g1,c47203_g2,c43044_g1,c43501_g1,c66467_g1,c36340_g1,c8202_g1,c34019_g1,c63880_g1,c11851_g1,c64429_g1,c45977_g1,c50299_g1,c104284_g1,c51142_g1,c19449_g1,c49091_g1,c72608_g1,c78700_g1,c18177_g1,c67546_g1,c10482_g1,c49902_g1,c73176_g1,c104455_g1,c45327_g2,c54124_g1,c39440_g1,c1753_g1,c9921_g1,c103322_g1,c6656_g1,c89336_g1,c50458_g1,c109884_g1,c21011_g1,c19829_g2,c85745_g1,c581_g1,c53386_g1,c54565_g1,c97646_g1,c57871_g1,c39207_g1,c46277_g1,c28291_g1,c16761_g1,c26363_g1,c76059_g1,c77042_g1,c20257_g2,c62374_g1,c87808_g1,c26_g1,c107752_g1,c46404_g3,c78609_g1,c97772_g1,c49242_g1,c45327_g3,c24600_g1,c61298_g1,c71604_g1,c48865_g1,c62156_g1,c18302_g1,c48964_g1,c58779_g1,c50016_g1,c40011_g1,c97042_g1,c110027_g1,c63898_g1,c13374_g1,c111270_g1,c9747_g2,c96911_g1,c47598_g1,c42128_g1,c55327_g1,c68022_g1,c110906_g1,c43427_g2,c77164_g1,c40769_g1,c48105_g1,c98527_g1,c93500_g1,c94053_g1,c30673_g1,c112079_g1,c72032_g1,c61407_g1,c58536_g1,c72127_g1,c110131_g1,c52271_g2,c48655_g1,c37822_g1,c24487_g1,c61032_g1,c28032_g1,c71603_g1,c15666_g1,c11478_g1,c73456_g1,c101270_g1,c52142_g2,c36681_g1,c62466_g1,c65758_g1,c38423_g1,c74712_g1,c49174_g1,c86582_g1,c72872_g1,c55637_g1,c67246_g1,c111518_g1,c46544_g1,c99146_g1,c57532_g1,c108002_g1,c59882_g1,c43733_g1,c1642_g1,c47889_g1,c45960_g1,c54443_g1,c82314_g1,c94934_g1,c83437_g1,c59896_g1,c97398_g1,c41524_g1,c17106_g1,c74256_g1,c78499_g1,c5988_g1,c46119_g2,c68678_g1,c48827_g1,c78405_g1,c83296_g1,c58199_g1,c73806_g1,c96465_g1,c65245_g1,c37431_g1,c79025_g1,c52466_g1,c67666_g1,c49366_g1,c82324_g1,c31740_g1,c76091_g1,c76864_g1,c85095_g1,c81892_g1,c43722_g1,c10950_g1,c42703_g1,c110160_g1,c29198_g1,c107026_g1,c42807_g1,c47764_g1,c57054_g1,c48230_g1,c99594_g1,c26284_g1,c84830_g1,c52381_g3,c29075_g1,c4023_g1,c45852_g1,c88093_g1,c103790_g1,c58356_g1,c73561_g1,c47115_g1,c40176_g1,c36810_g1,c83975_g1,c63265_g1,c51974_g1,c45838_g1,c47818_g1,c58304_g1,c43454_g1,c59464_g1,c52381_g2,c46775_g1,c78175_g1,c51499_g2,c62283_g1,c57655_g1,c46404_g2,c94570_g1,c89702_g1,c74521_g1,c87107_g1,c66235_g1,c72141_g1,c15105_g1,c79094_g1,c43883_g1,c31759_g1,c48310_g2,c104506_g1,c49356_g1,c83179_g1,c45578_g2,c13770_g1,c87279_g1 |
| Carotenoid biosynthesis | ko00906 | 41 | c52337_g2,c112744_g1,c36617_g1,c47061_g1,c41985_g1,c45663_g4,c83263_g1,c46893_g1,c50101_g1,c45219_g1,c48201_g1,c10574_g1,c30282_g1,c29635_g1,c48389_g1,c45889_g1,c13167_g1,c45051_g1,c108109_g1,c46290_g1,c59387_g1,c96832_g1,c68208_g1,c50244_g1,c84063_g1,c76981_g1,c49213_g1,c49450_g2,c50244_g2,c44798_g1,c43910_g1,c41483_g1,c50961_g1,c26780_g1,c66318_g1,c24229_g1,c45302_g1,c81887_g1,c96141_g1,c51667_g1,c35962_g1 |
| Chloroalkane and chloroalkene degradation | ko00625 | 52 | c48241_g1,c89219_g1,c47621_g1,c48536_g2,c47365_g1,c86580_g1,c68793_g1,c42135_g1,c106670_g1,c84047_g1,c40766_g1,c97539_g1,c75402_g1,c62190_g1,c9747_g1,c73811_g1,c93937_g1,c48536_g3,c34608_g1,c88691_g1,c75473_g1,c41057_g1,c22216_g1,c50235_g1,c75055_g1,c61823_g1,c108090_g1,c16528_g1,c29553_g1,c36418_g1,c92052_g1,c40100_g1,c49723_g1,c107473_g1,c52336_g2,c58316_g1,c33782_g2,c45085_g1,c102364_g1,c45977_g1,c19829_g2,c9747_g2,c62978_g1,c98527_g1,c46819_g1,c76192_g1,c1398_g1,c83915_g1,c89815_g1,c81892_g1,c41057_g2,c90369_g1 |
| Chlorocyclohexane and chlorobenzene degradation | ko00361 | 4 | c63842_g1,c21113_g2,c49975_g1,c57110_g1 |
| Citrate cycle (TCA cycle) | ko00020 | 220 | c53491_g1,c78806_g1,c18240_g1,c76954_g1,c110876_g1,c71672_g1,c45370_g1,c65199_g1,c74805_g1,c14873_g1,c77515_g1,c94923_g1,c88604_g1,c105979_g1,c102864_g1,c96407_g1,c9460_g1,c52927_g1,c63195_g1,c48533_g1,c45979_g1,c93517_g1,c11910_g1,c27203_g1,c98311_g1,c65926_g1,c92790_g1,c76596_g1,c55308_g1,c37292_g1,c103393_g1,c95742_g1,c65074_g1,c66648_g1,c11271_g1,c110039_g1,c21303_g1,c52959_g1,c45242_g1,c88843_g1,c76146_g1,c58217_g1,c84795_g1,c86261_g1,c9412_g1,c945_g1,c73958_g1,c110385_g1,c108204_g1,c68666_g1,c9460_g2,c40278_g1,c78140_g1,c68426_g1,c976_g1,c93137_g1,c42785_g1,c945_g2,c83854_g1,c25743_g1,c89066_g1,c112656_g1,c92836_g1,c99893_g1,c71092_g1,c26218_g1,c47022_g1,c97409_g1,c74445_g1,c42133_g1,c40011_g2,c10937_g1,c67729_g1,c61692_g1,c109587_g1,c88505_g1,c100842_g1,c20532_g1,c19508_g1,c67587_g1,c26_g2,c81098_g1,c75960_g1,c54418_g1,c37201_g1,c82112_g1,c56845_g1,c108398_g1,c23285_g1,c53308_g1,c83757_g1,c52142_g1,c85718_g1,c4149_g1,c78386_g1,c72940_g1,c100120_g1,c83062_g1,c77688_g1,c93721_g1,c75573_g1,c9388_g1,c53631_g1,c44012_g1,c65913_g1,c66953_g1,c10611_g1,c52404_g4,c16638_g1,c10908_g1,c24562_g1,c29152_g1,c87592_g1,c81440_g1,c25225_g1,c49145_g1,c78345_g1,c100202_g1,c94165_g1,c66906_g1,c21888_g1,c21314_g1,c55393_g1,c101352_g1,c41819_g1,c48887_g1,c101267_g1,c108664_g1,c51583_g1,c26866_g1,c48887_g2,c104067_g1,c67810_g1,c86489_g1,c75511_g1,c54243_g1,c24897_g1,c46725_g1,c109895_g1,c88902_g1,c90146_g1,c71923_g1,c74433_g1,c84138_g1,c62780_g1,c26628_g1,c12358_g1,c102419_g1,c51888_g3,c53329_g1,c3998_g1,c37085_g1,c14937_g1,c45902_g2,c30117_g1,c45578_g1,c79133_g1,c44229_g1,c104829_g1,c48920_g1,c101332_g1,c47203_g2,c66467_g1,c64429_g1,c50299_g1,c104284_g1,c73176_g1,c104455_g1,c6656_g1,c89336_g1,c54565_g1,c97646_g1,c16761_g1,c26363_g1,c87808_g1,c26_g1,c107752_g1,c97772_g1,c71604_g1,c48865_g1,c18302_g1,c48964_g1,c40011_g1,c110027_g1,c96911_g1,c68022_g1,c77164_g1,c93500_g1,c94053_g1,c110131_g1,c48655_g1,c28032_g1,c71603_g1,c11478_g1,c52291_g4,c73456_g1,c62466_g1,c65758_g1,c74712_g1,c86582_g1,c97398_g1,c17106_g1,c5988_g1,c83296_g1,c73806_g1,c79025_g1,c76864_g1,c43722_g1,c107026_g1,c99594_g1,c84830_g1,c73561_g1,c36810_g1,c83975_g1,c57655_g1,c66235_g1,c79094_g1,c104506_g1,c83179_g1,c87279_g1 |
| Cutin | ko00073 | 28 | c43681_g1,c94042_g1,c54799_g1,c491_g1,c59316_g1,c48278_g1,c16252_g1,c100671_g1,c50094_g1,c20049_g1,c64261_g1,c49905_g1,c83737_g1,c47699_g2,c102643_g1,c46313_g1,c41023_g1,c30382_g1,c93311_g1,c104225_g1,c66737_g1,c85902_g1,c89692_g1,c109686_g1,c110363_g1,c38144_g1,c62726_g1,c11111_g1 |
| Cyanoamino acid metabolism | ko00460 | 91 | c75871_g1,c26992_g1,c34485_g1,c43433_g1,c105092_g1,c40346_g1,c31636_g1,c4006_g1,c59715_g1,c45733_g1,c45667_g1,c98363_g1,c103806_g1,c63646_g1,c105620_g1,c48921_g1,c83090_g1,c99912_g1,c37640_g1,c34733_g1,c41596_g2,c46304_g2,c112628_g1,c55215_g1,c109012_g1,c46876_g1,c51877_g1,c84333_g1,c9748_g1,c46090_g1,c73306_g1,c35474_g1,c32367_g1,c42239_g1,c28388_g2,c14995_g1,c107170_g1,c85195_g1,c40728_g1,c50326_g1,c15006_g1,c47985_g1,c52483_g1,c1939_g1,c92777_g1,c40522_g1,c103333_g1,c44888_g1,c52507_g1,c26184_g1,c99744_g1,c98122_g1,c105150_g1,c71906_g1,c11662_g1,c35368_g1,c105463_g1,c103744_g1,c32052_g1,c57853_g1,c46907_g1,c106634_g1,c60986_g1,c50637_g1,c54124_g1,c58209_g1,c51615_g1,c103322_g1,c46907_g2,c58915_g1,c62156_g1,c47634_g1,c2063_g1,c48149_g1,c46907_g3,c45960_g1,c48740_g1,c86498_g1,c42151_g1,c107457_g1,c4381_g1,c29198_g1,c66108_g1,c66969_g1,c43454_g1,c33348_g1,c72141_g1,c52365_g1,c42276_g1,c11015_g1,c783_g1 |
| Cysteine and methionine metabolism | ko00270 | 198 | c18240_g1,c46973_g1,c110883_g1,c45370_g1,c55402_g1,c14873_g1,c96286_g1,c92695_g1,c47400_g1,c106885_g1,c94941_g1,c73106_g1,c106588_g1,c58880_g1,c30038_g1,c86188_g1,c73942_g1,c40311_g1,c53966_g1,c18319_g1,c105912_g1,c93517_g1,c19206_g1,c93666_g1,c36407_g1,c93494_g1,c47072_g1,c50882_g1,c73078_g1,c25404_g1,c80695_g1,c27298_g1,c42784_g1,c66286_g1,c40621_g1,c90205_g1,c74790_g1,c48669_g1,c3557_g1,c76146_g1,c77010_g1,c105550_g1,c83836_g1,c77109_g1,c94230_g1,c33708_g1,c19834_g1,c41099_g1,c85612_g1,c80270_g1,c99408_g1,c1035_g1,c1350_g1,c45866_g1,c100063_g1,c84258_g1,c39507_g1,c14545_g1,c106159_g1,c87086_g1,c40011_g2,c83027_g1,c98178_g1,c112630_g1,c35141_g1,c93988_g1,c26212_g1,c104246_g1,c31236_g1,c109512_g1,c103665_g1,c37201_g1,c62793_g1,c67135_g1,c50019_g1,c52142_g1,c57611_g1,c49564_g1,c8176_g2,c77583_g1,c12938_g1,c72500_g1,c30253_g2,c47900_g1,c12056_g1,c12938_g2,c47900_g2,c93721_g1,c91563_g1,c28212_g1,c98474_g1,c50526_g2,c105051_g1,c518_g1,c972_g1,c52472_g1,c110967_g1,c35544_g2,c50389_g1,c87855_g1,c97209_g1,c102800_g1,c45857_g1,c72683_g1,c104009_g1,c108018_g1,c45500_g1,c58532_g1,c33233_g1,c79123_g1,c50159_g1,c8863_g1,c48669_g2,c66141_g1,c27392_g1,c102754_g1,c73269_g1,c74720_g1,c104067_g1,c75607_g1,c34731_g1,c52205_g2,c44223_g1,c69203_g1,c54243_g1,c84626_g1,c104622_g1,c50000_g1,c11864_g1,c9503_g1,c78598_g1,c53312_g1,c56114_g1,c88808_g1,c82679_g1,c85544_g1,c73923_g1,c45902_g2,c50526_g3,c45578_g1,c45425_g1,c44637_g1,c59032_g1,c34116_g1,c95283_g1,c101332_g1,c44377_g1,c43501_g1,c45251_g1,c50787_g1,c78700_g1,c58822_g1,c43737_g1,c45327_g2,c1753_g1,c68383_g1,c103322_g1,c101370_g1,c46556_g1,c56892_g1,c43791_g1,c14803_g1,c113292_g1,c45327_g3,c48266_g1,c18302_g1,c40011_g1,c38946_g1,c85639_g1,c29146_g1,c61407_g1,c48655_g1,c74712_g1,c81302_g1,c11220_g1,c72925_g1,c95128_g1,c73090_g1,c31740_g1,c61234_g1,c41423_g1,c44203_g1,c107026_g1,c63958_g1,c16410_g1,c63265_g1,c51974_g1,c61153_g1,c39238_g1,c66235_g1,c72141_g1,c63465_g1,c49460_g3,c79094_g1,c18208_g1,c103549_g1,c112461_g1,c101156_g1 |
| Degradation of aromatic compounds | ko01220 | 34 | c48241_g1,c65676_g1,c68793_g1,c42135_g1,c82728_g1,c84047_g1,c9747_g1,c109882_g1,c93937_g1,c51428_g3,c75473_g1,c22216_g1,c71745_g1,c108090_g1,c61957_g1,c16528_g1,c107826_g1,c36418_g1,c92052_g1,c40100_g1,c107473_g1,c103522_g1,c33782_g2,c346_g1,c14751_g1,c45977_g1,c33398_g1,c19829_g2,c35949_g1,c9747_g2,c98527_g1,c46819_g1,c81892_g1,c17108_g1 |
| D-Glutamine and D-glutamate metabolism | ko00471 | 9 | c44498_g1,c42270_g1,c83834_g1,c9047_g1,c45909_g1,c45058_g2,c45058_g1,c22770_g1,c83077_g1 |
| Diterpenoid biosynthesis | ko00904 | 33 | c22766_g1,c10005_g1,c52682_g1,c43338_g1,c3074_g1,c71902_g1,c46628_g1,c21359_g1,c82835_g1,c68672_g1,c39601_g1,c83812_g1,c53705_g1,c51287_g1,c26819_g2,c17385_g1,c49999_g1,c15778_g1,c37864_g1,c11516_g1,c68464_g1,c17800_g1,c47472_g1,c31157_g1,c45036_g1,c57520_g1,c26819_g1,c87840_g1,c63947_g1,c45036_g2,c86336_g1,c101519_g1,c83547_g1 |
| Drug metabolism - cytochrome P450 | ko00982 | 64 | c48241_g1,c96098_g1,c104014_g1,c40882_g1,c71557_g1,c39643_g1,c11873_g1,c43955_g1,c68793_g1,c42135_g1,c93083_g1,c84047_g1,c38812_g1,c39643_g2,c9747_g1,c93937_g1,c45101_g1,c98338_g1,c9319_g1,c42729_g1,c83021_g1,c60051_g1,c75473_g1,c45697_g1,c22216_g1,c43652_g1,c106019_g1,c108090_g1,c42912_g1,c16528_g1,c39602_g1,c36418_g1,c87967_g1,c92052_g1,c40100_g1,c28793_g1,c49385_g1,c107473_g1,c9390_g1,c78636_g1,c47211_g3,c33782_g2,c84629_g1,c40886_g1,c17446_g1,c61392_g1,c40_g1,c45977_g1,c51598_g1,c37410_g1,c19829_g2,c47211_g2,c37299_g1,c41401_g1,c71559_g1,c9747_g2,c99066_g1,c98527_g1,c76728_g1,c46819_g1,c81892_g1,c40395_g1,c75009_g1,c43824_g1 |
| Drug metabolism - other enzymes | ko00983 | 61 | c54289_g1,c98613_g1,c33871_g1,c109324_g1,c64555_g1,c33816_g1,c83746_g1,c59919_g1,c17463_g1,c13175_g1,c41264_g1,c100658_g1,c215_g1,c46822_g1,c82486_g1,c98762_g1,c66882_g1,c77055_g1,c78560_g1,c72989_g1,c47798_g1,c44585_g1,c89508_g1,c106149_g1,c4317_g1,c57931_g1,c78249_g1,c62719_g1,c57572_g1,c99024_g1,c63836_g1,c88998_g1,c52493_g1,c39602_g1,c100270_g1,c50510_g1,c53666_g1,c49336_g1,c80248_g1,c39493_g1,c109730_g1,c81878_g1,c55233_g1,c105775_g1,c4317_g2,c45272_g1,c56741_g1,c67738_g1,c110788_g1,c37410_g1,c59078_g1,c42545_g1,c99066_g1,c102568_g1,c65388_g1,c60409_g1,c83336_g1,c37462_g1,c68744_g1,c84642_g1,c50316_g1 |
| Ether lipid metabolism | ko00565 | 26 | c51039_g2,c3700_g1,c50203_g2,c29797_g1,c63785_g1,c1591_g1,c51133_g1,c41055_g1,c47578_g1,c40729_g1,c45951_g1,c52888_g1,c50203_g1,c3957_g1,c30134_g1,c48602_g1,c45795_g1,c50837_g1,c24808_g1,c27249_g2,c45281_g1,c104274_g1,c44745_g1,c849_g1,c51828_g1,c51512_g4 |
| Fatty acid biosynthesis | ko00061 | 74 | c51048_g1,c91648_g1,c64170_g1,c51829_g1,c50246_g1,c9199_g1,c7453_g1,c62088_g1,c71634_g1,c84449_g1,c62277_g1,c97175_g1,c46917_g2,c49563_g1,c105584_g1,c31007_g1,c74426_g1,c5104_g1,c111491_g1,c98327_g1,c47937_g2,c110431_g1,c19291_g1,c42227_g1,c46982_g2,c13150_g1,c85100_g1,c44731_g1,c71973_g1,c61794_g1,c70483_g1,c84896_g1,c43130_g1,c73073_g1,c79285_g1,c76513_g1,c837_g1,c85941_g1,c80929_g1,c100324_g1,c107391_g1,c46166_g1,c6706_g1,c16700_g2,c75495_g1,c67839_g1,c71636_g1,c82477_g1,c61705_g1,c37035_g1,c52719_g8,c50138_g2,c46119_g1,c99135_g1,c42121_g1,c15679_g1,c29969_g1,c66721_g1,c11363_g1,c105645_g1,c62374_g1,c58779_g1,c31829_g1,c2198_g1,c93225_g1,c111518_g1,c46544_g1,c50506_g1,c54443_g1,c69630_g1,c59896_g1,c46119_g2,c15346_g1,c97941_g1 |
| Fatty acid degradation | ko00071 | 134 | c48241_g1,c48391_g4,c89219_g1,c47621_g1,c86204_g1,c48902_g1,c48536_g2,c35216_g1,c83586_g1,c27642_g1,c110089_g1,c99472_g1,c90263_g1,c47365_g1,c86580_g1,c27856_g1,c68793_g1,c42135_g1,c13064_g1,c106670_g1,c11144_g1,c48391_g2,c47670_g1,c84047_g1,c40766_g1,c48391_g1,c31403_g1,c52467_g2,c97539_g1,c75402_g1,c41724_g1,c62190_g1,c9747_g1,c47244_g1,c73811_g1,c93937_g1,c83786_g1,c77190_g1,c64000_g1,c48536_g3,c105841_g1,c34608_g1,c93852_g1,c52044_g2,c80976_g1,c47650_g1,c104495_g1,c91515_g1,c85859_g1,c54787_g1,c88691_g1,c90317_g1,c104011_g1,c46926_g4,c75473_g1,c41057_g1,c55541_g1,c22216_g1,c75123_g1,c44740_g1,c57773_g1,c50235_g1,c75055_g1,c61823_g1,c105469_g1,c53626_g1,c108090_g1,c58198_g1,c16528_g1,c103655_g1,c64933_g1,c29553_g1,c36418_g1,c77643_g1,c92052_g1,c45615_g1,c40100_g1,c42337_g1,c46912_g1,c108175_g1,c46156_g1,c52393_g3,c6594_g1,c49723_g1,c107473_g1,c91334_g1,c103615_g1,c52336_g2,c58316_g1,c33782_g2,c48391_g3,c45085_g1,c6536_g1,c106336_g1,c73577_g1,c88993_g1,c64873_g1,c98768_g1,c102364_g1,c51066_g1,c45977_g1,c9237_g1,c19829_g2,c29399_g1,c9747_g2,c62978_g1,c93690_g1,c103892_g1,c98527_g1,c37822_g1,c16880_g1,c71698_g1,c82737_g1,c107910_g1,c72872_g1,c46819_g1,c76192_g1,c87138_g1,c43660_g1,c51162_g1,c1398_g1,c83915_g1,c89815_g1,c81892_g1,c41057_g2,c90369_g1,c55937_g1,c110160_g1,c105824_g1,c93069_g1,c51499_g2,c86544_g1,c31759_g1,c26736_g1 |
| Fatty acid elongation | ko00062 | 45 | c45903_g1,c49877_g1,c1316_g1,c85912_g1,c36972_g1,c66184_g1,c39182_g1,c10620_g1,c42039_g1,c57186_g1,c43519_g1,c20142_g2,c55893_g1,c95324_g1,c89597_g1,c104182_g1,c42039_g2,c49845_g1,c64939_g1,c49346_g3,c9258_g1,c37616_g1,c35017_g1,c73054_g1,c64756_g1,c103318_g1,c38131_g3,c92834_g1,c45693_g1,c32242_g1,c53064_g1,c45748_g1,c103310_g1,c16248_g1,c96486_g1,c31025_g1,c11921_g1,c37841_g1,c49877_g2,c63304_g1,c110034_g1,c34278_g1,c41393_g1,c50103_g1,c45484_g1 |
| Fatty acid metabolism | ko01212 | 191 | c48391_g4,c51048_g1,c91648_g1,c64170_g1,c51829_g1,c45903_g1,c50246_g1,c9199_g1,c86204_g1,c48902_g1,c83586_g1,c27642_g1,c7453_g1,c99472_g1,c90263_g1,c62088_g1,c5941_g1,c71634_g1,c27856_g1,c84449_g1,c13064_g1,c42039_g1,c62277_g1,c97175_g1,c57186_g1,c11144_g1,c48391_g2,c47670_g1,c43519_g1,c108605_g1,c71720_g1,c20142_g2,c31092_g1,c46917_g2,c49563_g1,c48391_g1,c31403_g1,c105584_g1,c31007_g1,c37502_g1,c74426_g1,c52467_g2,c5104_g1,c111491_g1,c41724_g1,c98327_g1,c103085_g1,c47937_g2,c95324_g1,c110431_g1,c19291_g1,c83786_g1,c42227_g1,c77190_g1,c64000_g1,c46982_g2,c13150_g1,c85100_g1,c44731_g1,c105841_g1,c71973_g1,c93852_g1,c61794_g1,c52044_g2,c70483_g1,c80976_g1,c104182_g1,c47650_g1,c104495_g1,c42039_g2,c91515_g1,c85859_g1,c49845_g1,c54787_g1,c90317_g1,c104011_g1,c89210_g1,c64939_g1,c46926_g4,c84896_g1,c43130_g1,c35017_g1,c73073_g1,c75123_g1,c44740_g1,c79285_g1,c76513_g1,c57773_g1,c837_g1,c85941_g1,c105187_g1,c53626_g1,c90027_g1,c50317_g6,c80929_g1,c103655_g1,c100324_g1,c107391_g1,c64933_g1,c46166_g1,c77643_g1,c6706_g1,c45615_g1,c42337_g1,c16700_g2,c46912_g1,c75495_g1,c108175_g1,c46156_g1,c50317_g1,c52393_g3,c6594_g1,c91334_g1,c103615_g1,c67839_g1,c71636_g1,c64756_g1,c82477_g1,c61705_g1,c66424_g1,c48391_g3,c37035_g1,c50138_g2,c6536_g1,c106336_g1,c47306_g1,c73577_g1,c88993_g1,c92834_g1,c45693_g1,c44583_g1,c64873_g1,c98768_g1,c8801_g1,c61703_g1,c46119_g1,c99135_g1,c42121_g1,c15679_g1,c51066_g1,c29969_g1,c66721_g1,c11363_g1,c53064_g1,c105645_g1,c45748_g1,c103310_g1,c16248_g1,c9237_g1,c62374_g1,c58779_g1,c29399_g1,c31025_g1,c31829_g1,c2198_g1,c93690_g1,c103892_g1,c53357_g1,c37822_g1,c16880_g1,c93225_g1,c71698_g1,c82737_g1,c107910_g1,c72872_g1,c111518_g1,c46544_g1,c50506_g1,c54443_g1,c69630_g1,c59896_g1,c87138_g1,c46119_g2,c43660_g1,c12646_g1,c51162_g1,c76322_g1,c57537_g1,c55937_g1,c110160_g1,c63304_g1,c15346_g1,c97941_g1,c105824_g1,c93069_g1,c51499_g2,c34278_g1,c86544_g1,c41393_g1,c31759_g1,c26736_g1 |
| Flavone and flavonol biosynthesis | ko00944 | 12 | c12837_g1,c53453_g1,c82561_g1,c15754_g1,c49048_g7,c29230_g1,c29230_g2,c87677_g1,c70634_g1,c35342_g1,c62714_g1,c103862_g1 |
| Flavonoid biosynthesis | ko00941 | 58 | c12837_g1,c89588_g1,c1193_g1,c46370_g1,c53453_g1,c15063_g1,c82561_g1,c87450_g1,c37687_g1,c26805_g1,c15754_g1,c31395_g1,c51428_g3,c83030_g1,c46041_g1,c29230_g1,c93747_g1,c84218_g1,c29230_g2,c31168_g1,c87677_g1,c43720_g1,c36742_g1,c49138_g1,c103522_g1,c37277_g1,c83163_g1,c72777_g1,c346_g1,c52122_g1,c51160_g4,c35342_g1,c64830_g1,c11407_g1,c14751_g1,c89612_g1,c24369_g1,c62714_g1,c38231_g1,c35949_g1,c28083_g1,c40341_g1,c74253_g1,c40956_g1,c83161_g1,c103862_g1,c46108_g1,c13468_g1,c100652_g1,c62392_g1,c17108_g1,c4089_g1,c62366_g1,c47926_g1,c51227_g1,c9453_g1,c46529_g1,c4094_g1 |
| Fluorobenzoate degradation | ko00364 | 4 | c63842_g1,c21113_g2,c49975_g1,c57110_g1 |
| Folate biosynthesis | ko00790 | 28 | c7094_g1,c106981_g1,c218_g2,c44748_g1,c47939_g1,c87129_g1,c82656_g1,c83758_g1,c86348_g1,c49756_g2,c105468_g1,c35488_g1,c49004_g6,c8397_g1,c44052_g1,c30245_g1,c46930_g1,c49949_g6,c96535_g1,c42011_g3,c7773_g1,c52018_g1,c52482_g5,c88852_g1,c67819_g1,c96542_g1,c40411_g1,c83642_g1 |
| Fructose and mannose metabolism | ko00051 | 116 | c1378_g1,c12991_g1,c41857_g1,c35230_g1,c33335_g1,c73320_g1,c48673_g1,c39222_g1,c67593_g1,c61354_g1,c41978_g1,c6348_g1,c47044_g1,c102313_g1,c98388_g1,c48970_g1,c53135_g1,c47688_g1,c16496_g1,c72656_g1,c65053_g1,c107161_g1,c97593_g1,c61975_g1,c82202_g1,c76255_g1,c72006_g1,c99582_g1,c85320_g1,c36333_g1,c107554_g1,c34281_g1,c33934_g1,c38169_g1,c47044_g2,c103350_g1,c71507_g1,c41342_g1,c74132_g1,c45885_g1,c43795_g1,c106108_g1,c49506_g1,c69976_g1,c2020_g1,c21811_g2,c39848_g1,c27198_g1,c40802_g1,c67694_g1,c52882_g1,c43166_g1,c95382_g1,c4008_g1,c53986_g1,c75440_g1,c102169_g1,c100584_g1,c92847_g1,c54743_g1,c104082_g1,c44734_g1,c54222_g1,c54175_g1,c82664_g1,c65460_g1,c48310_g1,c42339_g1,c36720_g1,c91922_g1,c20019_g1,c62983_g1,c49781_g1,c53081_g1,c106844_g1,c53413_g1,c45764_g1,c25409_g1,c99612_g1,c57038_g1,c41338_g1,c31470_g1,c50236_g1,c19285_g1,c39440_g1,c109884_g1,c49242_g1,c85425_g1,c98060_g1,c13722_g1,c40769_g1,c30673_g1,c61032_g1,c43733_g1,c68678_g1,c48827_g1,c14647_g1,c51423_g2,c37431_g1,c82324_g1,c55503_g1,c79055_g1,c52381_g3,c47095_g1,c52381_g2,c107431_g1,c31549_g1,c16785_g1,c71874_g1,c21811_g1,c104237_g1,c28386_g1,c49248_g1,c48310_g2,c33832_g1,c13770_g1 |
| Galactose metabolism | ko00052 | 99 | c48345_g1,c104176_g1,c30143_g1,c25499_g1,c29259_g1,c104216_g1,c6348_g1,c49964_g2,c86706_g1,c40559_g1,c63753_g1,c67994_g1,c47534_g1,c72568_g1,c94683_g1,c44235_g1,c50574_g1,c71270_g1,c61975_g1,c46476_g2,c78558_g1,c54133_g1,c28160_g1,c79990_g1,c93993_g1,c33934_g1,c38169_g1,c61203_g1,c74356_g1,c45885_g1,c106108_g1,c82676_g1,c81638_g1,c56842_g1,c83930_g1,c21811_g2,c39848_g1,c74204_g1,c30953_g1,c40912_g1,c102169_g1,c100584_g1,c111150_g1,c48458_g2,c27149_g1,c46365_g1,c45581_g1,c50882_g2,c82664_g1,c65460_g1,c55616_g1,c68640_g1,c42366_g1,c62983_g1,c35654_g1,c56290_g1,c34189_g1,c43584_g1,c47317_g1,c41639_g1,c6624_g1,c73787_g1,c45764_g1,c62487_g1,c31470_g1,c50236_g1,c18342_g1,c85363_g1,c39440_g1,c109884_g1,c108220_g1,c96091_g1,c44019_g2,c49242_g1,c85425_g1,c46597_g1,c81861_g1,c98060_g1,c40769_g1,c30673_g1,c46480_g1,c103688_g1,c43733_g1,c41577_g1,c13328_g1,c35092_g1,c52381_g3,c89199_g1,c52381_g2,c31549_g1,c16785_g1,c29287_g1,c71874_g1,c21811_g1,c82886_g1,c63024_g1,c48647_g1,c50003_g1,c98280_g1 |
| Geraniol degradation | ko00281 | 5 | c92986_g1,c82596_g1,c107241_g1,c54704_g1,c39018_g1 |
| Glucosinolate biosynthesis | ko00966 | 7 | c25134_g1,c41596_g2,c54745_g1,c39609_g1,c44303_g1,c42054_g1,c71327_g1 |
| Glutathione metabolism | ko00480 | 151 | c38643_g1,c46994_g1,c52428_g1,c88604_g1,c103519_g1,c96098_g1,c105979_g1,c38078_g1,c41536_g1,c30038_g1,c104014_g1,c40882_g1,c96653_g1,c61356_g1,c46582_g1,c71557_g1,c20519_g1,c59059_g1,c71593_g1,c18319_g1,c103464_g1,c27228_g1,c39643_g1,c11873_g1,c82135_g1,c84932_g1,c110039_g1,c52959_g1,c93083_g1,c47717_g1,c99904_g1,c77010_g1,c84428_g1,c42371_g1,c112666_g1,c38812_g1,c39643_g2,c5260_g1,c85090_g1,c89741_g1,c39556_g1,c43637_g1,c3922_g1,c45101_g1,c54761_g1,c38682_g1,c33993_g1,c16419_g1,c42133_g1,c113262_g1,c98338_g1,c103950_g1,c9319_g1,c81276_g1,c42729_g1,c75960_g1,c90984_g1,c25720_g1,c60051_g1,c88571_g1,c45697_g1,c77041_g1,c48444_g1,c43652_g1,c106019_g1,c14781_g1,c113143_g1,c87181_g1,c96381_g1,c9388_g1,c74668_g1,c57602_g1,c42912_g1,c972_g1,c30928_g1,c37014_g1,c87967_g1,c86069_g1,c25669_g1,c48887_g1,c107580_g1,c28793_g1,c57277_g1,c49385_g1,c98703_g1,c48887_g2,c49666_g1,c84312_g1,c34731_g1,c107669_g1,c9390_g1,c78636_g1,c47211_g3,c42999_g1,c10998_g1,c47484_g1,c99079_g1,c59511_g1,c84629_g1,c71515_g1,c106600_g1,c43752_g1,c88767_g1,c44580_g1,c62780_g1,c17446_g1,c5476_g1,c88094_g1,c9569_g1,c62106_g1,c72620_g1,c44609_g1,c43105_g1,c40_g1,c86870_g1,c54295_g1,c100365_g1,c78106_g1,c75248_g1,c72608_g1,c84888_g1,c48922_g6,c51598_g1,c57871_g1,c16761_g1,c47211_g2,c1102_g1,c37299_g1,c41401_g1,c71604_g1,c54352_g1,c71559_g1,c74038_g1,c512_g1,c96310_g1,c61214_g2,c82314_g1,c94934_g1,c72925_g1,c43722_g1,c21974_g1,c48997_g2,c50625_g1,c40395_g1,c45355_g1,c26248_g1,c40176_g1,c5325_g1,c97161_g1,c43824_g1,c3988_g1 |
| Glycerolipid metabolism | ko00561 | 125 | c51448_g1,c89219_g1,c57341_g1,c52513_g1,c25499_g1,c47621_g1,c49964_g2,c48536_g2,c35216_g1,c110089_g1,c63753_g1,c48966_g1,c11103_g1,c47365_g1,c86580_g1,c65676_g1,c43341_g1,c19227_g1,c106670_g1,c51298_g1,c82728_g1,c40766_g1,c2626_g1,c48612_g2,c38627_g1,c112776_g1,c65707_g1,c61975_g1,c106420_g1,c97539_g1,c75402_g1,c62190_g1,c47244_g1,c73811_g1,c103543_g1,c77769_g1,c62222_g1,c109882_g1,c36845_g1,c40729_g1,c48536_g3,c40834_g1,c28521_g1,c34608_g1,c33934_g1,c50528_g1,c88691_g1,c82115_g1,c41057_g1,c55541_g1,c106108_g1,c48136_g1,c50235_g1,c21811_g2,c75055_g1,c61823_g1,c13327_g1,c71745_g1,c41564_g1,c105469_g1,c52159_g2,c61957_g1,c107826_g1,c101845_g1,c102169_g1,c29553_g1,c48458_g2,c42470_g1,c11157_g1,c47592_g1,c82664_g1,c65460_g1,c49723_g1,c52336_g2,c58316_g1,c62983_g1,c45085_g1,c34861_g1,c56463_g1,c989_g1,c4451_g1,c47317_g1,c49406_g3,c45764_g1,c76677_g1,c45356_g1,c36483_g1,c72822_g1,c102364_g1,c47366_g1,c46996_g1,c62506_g1,c65627_g1,c33398_g1,c51822_g1,c39999_g1,c85425_g1,c98060_g1,c62978_g1,c97172_g1,c50980_g1,c44889_g1,c65082_g1,c47600_g1,c76192_g1,c49205_g1,c1398_g1,c41577_g1,c83915_g1,c89815_g1,c41057_g2,c90369_g1,c57054_g1,c32245_g1,c29108_g1,c50621_g1,c49406_g2,c31549_g1,c16785_g1,c71874_g1,c21811_g1,c57040_g1,c76545_g1,c39212_g1,c51828_g1 |
| Glycerophospholipid metabolism | ko00564 | 129 | c53930_g1,c51039_g2,c40273_g1,c52513_g1,c3700_g1,c40930_g1,c48677_g1,c42917_g1,c61108_g1,c50203_g2,c29797_g1,c72184_g1,c82218_g1,c63785_g1,c49596_g2,c20984_g1,c1591_g1,c51133_g1,c67584_g1,c41055_g1,c36266_g1,c47578_g1,c84609_g1,c14182_g1,c43782_g1,c570_g1,c59147_g1,c2626_g1,c38627_g1,c65707_g1,c106420_g1,c39230_g1,c103543_g1,c77769_g1,c72939_g1,c36845_g1,c40729_g1,c38644_g1,c45951_g1,c40834_g1,c44817_g1,c52888_g1,c107892_g1,c38024_g1,c94842_g1,c94906_g1,c63689_g1,c74694_g1,c16404_g1,c54517_g1,c28154_g1,c82115_g1,c108597_g1,c13327_g1,c38212_g1,c50203_g1,c5515_g1,c101845_g1,c65830_g1,c44249_g2,c50515_g1,c105007_g1,c94625_g1,c58293_g1,c43990_g1,c44249_g1,c76704_g1,c34861_g1,c18264_g1,c3957_g1,c25713_g1,c30134_g1,c49406_g3,c76677_g1,c36483_g1,c44723_g1,c47366_g1,c10676_g1,c62234_g1,c48602_g1,c62506_g1,c45795_g1,c50837_g1,c97495_g1,c54339_g1,c63349_g1,c24808_g1,c95708_g1,c15672_g1,c82763_g1,c65627_g1,c68060_g1,c50944_g2,c86222_g1,c47553_g1,c27249_g2,c73892_g1,c88977_g1,c46665_g1,c51822_g1,c45281_g1,c52549_g2,c47270_g1,c54167_g1,c96024_g1,c97172_g1,c50980_g1,c36220_g1,c44889_g1,c46627_g1,c65082_g1,c47600_g1,c104274_g1,c44745_g1,c27091_g1,c49205_g1,c92025_g1,c68713_g1,c32245_g1,c93218_g1,c50621_g1,c49406_g2,c849_g1,c104971_g1,c57040_g1,c110728_g1,c75534_g1,c51828_g1,c51512_g4 |
| Glycine | ko00260 | 175 | c53491_g1,c51081_g1,c53592_g1,c8857_g1,c30874_g1,c46281_g1,c31636_g1,c42988_g1,c83979_g1,c35216_g1,c40311_g1,c59715_g1,c25123_g2,c59201_g1,c110089_g1,c24934_g1,c11424_g1,c47185_g1,c50179_g2,c98363_g1,c100948_g1,c42027_g1,c19866_g1,c40487_g1,c105628_g1,c83241_g1,c86423_g1,c95469_g1,c19834_g1,c108204_g1,c55130_g1,c43711_g1,c29684_g1,c12311_g1,c47244_g1,c97094_g1,c43677_g1,c96344_g1,c48427_g1,c110541_g1,c50870_g1,c34733_g1,c90400_g1,c82786_g1,c53873_g1,c41831_g1,c109012_g1,c107366_g1,c64814_g1,c104908_g1,c109950_g1,c14545_g1,c26471_g1,c38897_g1,c23993_g1,c54435_g1,c25123_g1,c20257_g1,c39505_g1,c36513_g1,c39997_g1,c40487_g2,c74924_g1,c46898_g1,c56783_g1,c64297_g1,c50019_g1,c55541_g1,c42239_g1,c77583_g1,c41975_g3,c106540_g1,c45688_g1,c7643_g1,c105807_g1,c82266_g1,c63436_g1,c105469_g1,c14995_g1,c58119_g1,c87186_g1,c93038_g1,c11348_g1,c59612_g1,c97209_g1,c85195_g1,c44349_g1,c7088_g1,c15006_g1,c84615_g1,c1939_g1,c50868_g1,c44958_g2,c79123_g1,c8863_g1,c40887_g1,c40850_g1,c68624_g1,c69203_g1,c57349_g1,c72626_g1,c46725_g1,c98122_g1,c9483_g1,c109895_g1,c9285_g2,c48427_g2,c38803_g1,c98277_g1,c63367_g1,c53687_g1,c42918_g1,c30117_g1,c46700_g1,c26617_g1,c44229_g1,c83605_g1,c50272_g1,c95283_g1,c103084_g1,c63880_g1,c64429_g1,c19449_g1,c54124_g1,c9921_g1,c6656_g1,c72220_g1,c29832_g1,c2941_g1,c21011_g1,c46556_g1,c76059_g1,c20257_g2,c39999_g1,c37732_g2,c14803_g1,c47589_g1,c61298_g1,c62156_g1,c97042_g1,c106754_g1,c55327_g1,c112079_g1,c72032_g1,c48882_g1,c92958_g1,c24487_g1,c101270_g1,c85369_g1,c48708_g1,c47618_g1,c59882_g1,c45960_g1,c74256_g1,c78405_g1,c95128_g1,c75861_g1,c33979_g1,c90617_g1,c62415_g1,c42703_g1,c29198_g1,c45852_g1,c58356_g1,c73561_g1,c45838_g1,c43454_g1,c59464_g1,c39238_g1,c74521_g1,c87107_g1,c32423_g1,c51868_g1,c44958_g1,c54230_g1 |
| Glycolysis / Gluconeogenesis | ko00010 | 311 | c48241_g1,c104137_g1,c12991_g1,c53491_g1,c45645_g1,c38356_g1,c41857_g1,c89219_g1,c29194_g1,c30143_g1,c8202_g2,c48127_g2,c46874_g1,c8857_g1,c96551_g1,c102864_g1,c39222_g1,c104210_g1,c47621_g1,c101256_g1,c6348_g1,c85940_g1,c47044_g1,c48844_g2,c42988_g1,c48536_g2,c45979_g1,c35216_g1,c68302_g1,c5249_g1,c100050_g1,c57794_g1,c46404_g4,c25123_g2,c110089_g1,c24934_g1,c67994_g1,c52344_g5,c47365_g1,c27600_g1,c92790_g1,c67788_g1,c86580_g1,c56675_g1,c47534_g1,c48094_g1,c65676_g1,c17039_g1,c96933_g1,c98388_g1,c66648_g1,c46404_g1,c19297_g1,c48970_g1,c49559_g2,c53135_g1,c68793_g1,c47688_g1,c42135_g1,c45242_g1,c61871_g1,c16496_g1,c37799_g1,c106670_g1,c99669_g1,c82728_g1,c44028_g1,c84047_g1,c40766_g1,c107161_g1,c108204_g1,c36049_g1,c99880_g1,c97539_g1,c38793_g1,c75402_g1,c62190_g1,c43427_g1,c9747_g1,c47244_g1,c18147_g1,c82202_g1,c73811_g1,c76255_g1,c106435_g1,c40278_g1,c78140_g1,c72006_g1,c99582_g1,c46151_g1,c110541_g1,c99408_g1,c109882_g1,c93937_g1,c51735_g1,c12004_g1,c45866_g1,c25743_g1,c48536_g3,c64130_g1,c104310_g1,c34608_g1,c34281_g1,c47022_g1,c68919_g1,c29097_g1,c38169_g1,c38897_g1,c76028_g1,c67729_g1,c47668_g1,c47044_g2,c60359_g1,c88691_g1,c25123_g1,c20257_g1,c72186_g1,c26_g2,c85117_g1,c48132_g2,c82112_g1,c56845_g1,c56971_g1,c87608_g1,c23285_g1,c75473_g1,c41057_g1,c103573_g1,c55541_g1,c77371_g1,c83757_g1,c45885_g1,c22216_g1,c81638_g1,c82149_g1,c50235_g1,c79946_g1,c84114_g1,c581_g2,c99342_g1,c47074_g1,c109380_g1,c75055_g1,c61823_g1,c39848_g1,c71745_g1,c27198_g1,c40802_g1,c52882_g1,c105469_g1,c48823_g1,c108090_g1,c82456_g1,c71759_g1,c74204_g1,c95141_g1,c43166_g1,c49207_g1,c61957_g1,c16528_g1,c75440_g1,c66953_g1,c107826_g1,c24562_g1,c57387_g1,c102800_g1,c29553_g1,c100584_g1,c36418_g1,c52939_g1,c54743_g1,c54211_g1,c92052_g1,c40100_g1,c21888_g1,c44734_g1,c21314_g1,c54222_g1,c48262_g1,c54175_g1,c28265_g1,c43644_g1,c48310_g1,c79843_g1,c36720_g1,c49723_g1,c107473_g1,c69093_g1,c82523_g1,c55616_g1,c52336_g2,c58316_g1,c33782_g2,c46725_g1,c57149_g1,c39352_g1,c109895_g1,c35654_g1,c45085_g1,c90146_g1,c30135_g1,c71923_g1,c74433_g1,c9043_g1,c33875_g1,c38803_g1,c66208_g1,c99539_g1,c18541_g1,c25409_g1,c99612_g1,c57038_g1,c56604_g1,c93775_g1,c30117_g1,c41338_g1,c102364_g1,c44637_g1,c92573_g1,c44229_g1,c31470_g1,c62502_g1,c50236_g1,c58823_g1,c47203_g2,c85363_g1,c8202_g1,c64429_g1,c45977_g1,c50299_g1,c19449_g1,c33398_g1,c67546_g1,c39440_g1,c9921_g1,c6656_g1,c109884_g1,c19829_g2,c85745_g1,c581_g1,c53386_g1,c28291_g1,c77042_g1,c20257_g2,c50633_g1,c26_g1,c46404_g3,c49242_g1,c24600_g1,c61298_g1,c48865_g1,c48964_g1,c50016_g1,c97042_g1,c110027_g1,c63898_g1,c13374_g1,c61891_g1,c9747_g2,c62978_g1,c110906_g1,c43427_g2,c40769_g1,c98527_g1,c93500_g1,c30673_g1,c112079_g1,c58536_g1,c72127_g1,c61032_g1,c71603_g1,c46480_g1,c101270_g1,c38423_g1,c49174_g1,c46819_g1,c43733_g1,c76192_g1,c535_g1,c68678_g1,c48827_g1,c58199_g1,c96465_g1,c1398_g1,c37431_g1,c82324_g1,c83915_g1,c89815_g1,c81892_g1,c41057_g2,c90369_g1,c10950_g1,c63958_g1,c47764_g1,c99594_g1,c26284_g1,c52381_g3,c45852_g1,c73561_g1,c58304_g1,c52381_g2,c37200_g1,c46404_g2,c94570_g1,c89702_g1,c29287_g1,c48310_g2,c49356_g1,c103549_g1,c63024_g1,c13770_g1 |
| Glycosaminoglycan biosynthesis - heparan sulfate / heparin | ko00534 | 1 | c43035_g1 |
| Glycosaminoglycan degradation | ko00531 | 34 | c74497_g1,c48345_g1,c35102_g1,c63671_g1,c105174_g1,c29259_g1,c47273_g1,c107087_g1,c48316_g1,c46476_g2,c50004_g1,c48701_g1,c54133_g1,c66612_g1,c41850_g1,c101668_g1,c100289_g1,c87332_g1,c84191_g1,c64700_g1,c38152_g1,c61900_g1,c51321_g1,c33696_g1,c68640_g1,c50714_g1,c73787_g1,c72702_g1,c57866_g1,c106393_g1,c49151_g1,c68029_g1,c29256_g2,c71625_g1 |
| Glycosphingolipid biosynthesis - ganglio series | ko00604 | 16 | c74497_g1,c48345_g1,c105174_g1,c29259_g1,c46476_g2,c50004_g1,c54133_g1,c84191_g1,c61900_g1,c68640_g1,c73787_g1,c57866_g1,c106393_g1,c49151_g1,c29256_g2,c71625_g1 |
| Glycosphingolipid biosynthesis - globo series | ko00603 | 19 | c74497_g1,c105174_g1,c25499_g1,c49964_g2,c63753_g1,c50004_g1,c84191_g1,c49638_g1,c48458_g2,c61900_g1,c45255_g1,c47317_g1,c57866_g1,c36245_g1,c106393_g1,c49151_g1,c41577_g1,c29256_g2,c71625_g1 |
| Glycosylphosphatidylinositol(GPI)-anchor biosynthesis | ko00563 | 24 | c45486_g1,c51827_g1,c48769_g1,c43128_g1,c48615_g1,c48363_g1,c46179_g2,c28237_g1,c40569_g1,c51723_g3,c100015_g1,c29493_g1,c48936_g1,c49950_g1,c62973_g1,c25506_g1,c51872_g1,c47494_g1,c49721_g1,c53863_g1,c41228_g1,c45131_g1,c50529_g1,c38981_g1 |
| Glyoxylate and dicarboxylate metabolism | ko00630 | 156 | c19122_g1,c18240_g1,c78634_g1,c45370_g1,c14873_g1,c52843_g1,c54766_g1,c52927_g1,c31636_g1,c48902_g1,c62442_g1,c48533_g1,c57092_g1,c59715_g1,c93517_g1,c93426_g1,c71859_g1,c37292_g1,c44030_g1,c98363_g1,c59098_g1,c103393_g1,c93591_g1,c19866_g1,c76146_g1,c84795_g1,c112512_g1,c945_g1,c95320_g1,c110385_g1,c40431_g1,c72878_g1,c34733_g1,c90400_g1,c42785_g1,c82786_g1,c945_g2,c83854_g1,c77190_g1,c109012_g1,c107366_g1,c89066_g1,c71092_g1,c26218_g1,c28542_g2,c80976_g1,c97409_g1,c26471_g1,c78259_g1,c40011_g2,c44774_g1,c100842_g1,c19508_g1,c102809_g1,c44181_g1,c40403_g1,c37201_g1,c112258_g1,c53308_g1,c52142_g1,c42239_g1,c28352_g1,c41975_g3,c75123_g1,c48492_g1,c43714_g1,c45688_g1,c4149_g1,c78386_g1,c51480_g1,c72940_g1,c100120_g1,c55749_g1,c85429_g1,c93721_g1,c63436_g1,c14995_g1,c19122_g2,c11153_g1,c62375_g1,c58119_g1,c73295_g1,c11348_g1,c13859_g1,c10908_g1,c64933_g1,c85195_g1,c25225_g1,c50326_g1,c66906_g1,c15006_g1,c1939_g1,c62384_g1,c64671_g1,c51583_g1,c104067_g1,c103615_g1,c95064_g1,c54243_g1,c98122_g1,c55062_g1,c84138_g1,c19122_g3,c12358_g1,c102419_g1,c45816_g1,c45902_g2,c45578_g1,c79133_g1,c26617_g1,c104829_g1,c101332_g1,c100039_g1,c3591_g1,c51142_g1,c73343_g1,c54124_g1,c89336_g1,c54565_g1,c63784_g1,c42418_g1,c39999_g1,c37732_g2,c62156_g1,c18302_g1,c29206_g1,c40011_g1,c54520_g1,c48655_g1,c37822_g1,c24487_g1,c28032_g1,c93760_g1,c65758_g1,c74712_g1,c72872_g1,c57532_g1,c1642_g1,c45960_g1,c41524_g1,c83296_g1,c93516_g1,c65245_g1,c79025_g1,c33979_g1,c62415_g1,c42703_g1,c29198_g1,c107026_g1,c93711_g1,c61668_g1,c13249_g2,c43454_g1,c66235_g1,c79094_g1,c31759_g1 |
| Histidine metabolism | ko00340 | 56 | c89219_g1,c57311_g1,c42740_g1,c47621_g1,c48536_g2,c35216_g1,c45381_g1,c110089_g1,c47365_g1,c44873_g1,c86580_g1,c77300_g1,c106670_g1,c40766_g1,c97539_g1,c75402_g1,c62190_g1,c47244_g1,c73811_g1,c48536_g3,c34608_g1,c98299_g1,c51128_g1,c37416_g1,c88691_g1,c41057_g1,c55541_g1,c50235_g1,c75055_g1,c61823_g1,c105469_g1,c91964_g1,c29553_g1,c82781_g1,c101915_g1,c50150_g1,c49723_g1,c52336_g2,c58316_g1,c66337_g1,c13402_g1,c45085_g1,c102364_g1,c50699_g1,c55154_g1,c62978_g1,c48658_g1,c76192_g1,c1398_g1,c83915_g1,c89815_g1,c41057_g2,c90369_g1,c11162_g1,c41369_g1,c85823_g1 |
| Indole alkaloid biosynthesis | ko00901 | 4 | c72812_g1,c33111_g1,c85625_g1,c105896_g1 |
| Inositol phosphate metabolism | ko00562 | 82 | c111201_g1,c44778_g1,c106784_g1,c78834_g1,c19074_g1,c109139_g1,c47420_g1,c69486_g1,c98388_g1,c92658_g1,c53135_g1,c47688_g1,c79588_g1,c47578_g1,c16496_g1,c84609_g1,c85892_g1,c41491_g1,c45576_g1,c107161_g1,c108570_g1,c47384_g1,c37159_g1,c7923_g1,c38764_g1,c76255_g1,c25403_g1,c103865_g1,c47840_g1,c51930_g1,c45951_g1,c45182_g1,c38471_g1,c17647_g1,c16404_g1,c27943_g1,c50987_g1,c28513_g1,c48454_g1,c83324_g1,c27198_g1,c96322_g1,c13281_g1,c48999_g1,c45736_g1,c47405_g1,c46990_g2,c50324_g1,c55469_g1,c54222_g1,c45836_g1,c40171_g1,c91460_g1,c28439_g1,c101725_g1,c72745_g1,c32939_g1,c64363_g1,c99612_g1,c41338_g1,c3572_g1,c44681_g1,c45795_g1,c50837_g1,c46868_g1,c44778_g2,c46800_g1,c18872_g1,c34019_g1,c50791_g1,c44060_g1,c107467_g1,c64601_g1,c76881_g1,c48724_g1,c52092_g1,c52617_g1,c29296_g1,c43923_g1,c46775_g1,c22860_g1,c64976_g1 |
| Isoflavonoid biosynthesis | ko00943 | 1 | c109247_g1 |
| Isoquinoline alkaloid biosynthesis | ko00950 | 64 | c110883_g1,c61635_g1,c47400_g1,c73106_g1,c106588_g1,c82363_g1,c46702_g1,c86188_g1,c29251_g1,c35259_g1,c19206_g1,c36407_g1,c80695_g1,c47185_g1,c40621_g1,c13239_g1,c36419_g1,c80270_g1,c50870_g1,c100784_g1,c1350_g1,c44795_g1,c106159_g1,c47713_g1,c52843_g3,c36513_g1,c39997_g1,c56783_g1,c57611_g1,c12938_g1,c31736_g1,c12938_g2,c68148_g1,c28212_g1,c50389_g1,c109475_g1,c40850_g1,c24713_g1,c84626_g1,c72626_g1,c104622_g1,c78598_g1,c83145_g1,c73923_g1,c15049_g1,c54662_g1,c34116_g1,c103084_g1,c43501_g1,c51578_g1,c1753_g1,c88333_g1,c38946_g1,c48882_g1,c47618_g1,c109944_g1,c48686_g1,c41423_g1,c110574_g1,c63265_g1,c32423_g1,c51868_g1,c48624_g1,c45458_g1 |
| Limonene and pinene degradation | ko00903 | 69 | c62744_g1,c89219_g1,c47621_g1,c48536_g2,c49461_g1,c47365_g1,c86580_g1,c44494_g1,c106670_g1,c63072_g1,c52112_g1,c57881_g1,c52112_g2,c40766_g1,c24471_g1,c54530_g1,c97539_g1,c95509_g1,c75402_g1,c62190_g1,c73811_g1,c48536_g3,c52112_g3,c34608_g1,c110831_g1,c11088_g1,c52941_g1,c54345_g1,c83889_g1,c88691_g1,c41057_g1,c25007_g2,c50235_g1,c99792_g1,c75055_g1,c61823_g1,c108064_g1,c36408_g1,c29553_g1,c33474_g1,c43675_g2,c49723_g1,c52336_g2,c58316_g1,c50878_g1,c45085_g1,c82195_g1,c52985_g1,c11795_g1,c102364_g1,c2765_g1,c46140_g1,c10651_g1,c25106_g1,c71527_g1,c10846_g1,c62978_g1,c110641_g1,c37774_g1,c76192_g1,c71682_g1,c27078_g1,c1398_g1,c83915_g1,c89815_g1,c41057_g2,c90369_g1,c41788_g1,c82973_g1 |
| Linoleic acid metabolism | ko00591 | 36 | c14844_g1,c47880_g1,c71538_g1,c41055_g1,c44921_g1,c28142_g1,c47041_g1,c29052_g1,c42828_g1,c67014_g1,c49861_g1,c44136_g1,c52913_g1,c6973_g1,c48719_g1,c97404_g1,c18036_g1,c107064_g1,c94204_g1,c47159_g1,c47901_g1,c42787_g1,c93523_g1,c100363_g1,c27222_g1,c48375_g1,c72198_g1,c59619_g1,c103803_g1,c94735_g1,c56694_g1,c47159_g2,c49345_g2,c52376_g1,c51828_g1,c61344_g1 |
| Lipoic acid metabolism | ko00785 | 13 | c66137_g1,c49621_g1,c54025_g1,c40484_g1,c66002_g1,c51718_g3,c48927_g1,c51421_g1,c34433_g1,c51718_g8,c99353_g1,c38051_g2,c50089_g1 |
| Lipopolysaccharide biosynthesis | ko00540 | 16 | c109286_g1,c77565_g1,c56630_g1,c52995_g1,c31080_g1,c26822_g1,c84815_g1,c53355_g1,c62358_g1,c95080_g1,c49121_g1,c52096_g1,c31698_g1,c65993_g1,c71562_g1,c48008_g1 |
| Lysine biosynthesis | ko00300 | 30 | c868_g1,c35216_g1,c40311_g1,c110089_g1,c19834_g1,c73282_g1,c47244_g1,c50115_g1,c14545_g1,c50019_g1,c44849_g2,c55541_g1,c29316_g1,c45119_g1,c105469_g1,c97209_g1,c83928_g1,c79123_g1,c8863_g1,c107446_g1,c69203_g1,c87345_g1,c79871_g1,c46556_g1,c14803_g1,c43595_g1,c95128_g1,c106552_g1,c39238_g1,c35146_g1 |
| Lysine degradation | ko00310 | 85 | c89219_g1,c71672_g1,c65199_g1,c74805_g1,c53592_g1,c30874_g1,c47621_g1,c48902_g1,c48536_g2,c35216_g1,c110089_g1,c47365_g1,c86580_g1,c50367_g1,c95742_g1,c11271_g1,c106670_g1,c73958_g1,c40766_g1,c52752_g1,c73821_g1,c97539_g1,c75402_g1,c62190_g1,c47244_g1,c73811_g1,c67720_g1,c976_g1,c67913_g1,c48154_g1,c77190_g1,c48536_g3,c112656_g1,c34608_g1,c80976_g1,c88691_g1,c54418_g1,c41057_g1,c55541_g1,c75123_g1,c50235_g1,c75055_g1,c61823_g1,c105469_g1,c65913_g1,c64933_g1,c29553_g1,c81440_g1,c78345_g1,c52752_g2,c44958_g2,c108664_g1,c26866_g1,c49723_g1,c103615_g1,c52336_g2,c58316_g1,c45085_g1,c88902_g1,c51888_g3,c14937_g1,c9559_g1,c102364_g1,c51779_g2,c52338_g1,c62978_g1,c68022_g1,c110131_g1,c37822_g1,c72872_g1,c78472_g1,c76192_g1,c17106_g1,c46335_g1,c85410_g1,c1398_g1,c83915_g1,c89815_g1,c41057_g2,c90369_g1,c36604_g2,c57655_g1,c44958_g1,c31759_g1,c83179_g1 |
| Metabolism of xenobiotics by cytochrome P450 | ko00980 | 76 | c48241_g1,c96098_g1,c104014_g1,c40882_g1,c71557_g1,c72152_g1,c39643_g1,c11873_g1,c68793_g1,c84190_g1,c42135_g1,c52287_g1,c93083_g1,c84047_g1,c38812_g1,c39643_g2,c9747_g1,c93937_g1,c63904_g1,c45101_g1,c32774_g1,c98338_g1,c9319_g1,c42729_g1,c60051_g1,c75473_g1,c45697_g1,c22216_g1,c8401_g1,c43652_g1,c106019_g1,c108090_g1,c42912_g1,c16528_g1,c21934_g1,c29603_g1,c49343_g1,c39602_g1,c36418_g1,c19935_g2,c87967_g1,c92052_g1,c40100_g1,c61878_g1,c28793_g1,c49385_g1,c52376_g2,c107473_g1,c9390_g1,c78636_g1,c47211_g3,c33782_g2,c84629_g1,c17446_g1,c40_g1,c83529_g1,c45977_g1,c47780_g1,c51598_g1,c37410_g1,c19829_g2,c98867_g1,c47211_g2,c37299_g1,c41401_g1,c71559_g1,c9747_g2,c99066_g1,c98527_g1,c46819_g1,c72293_g1,c28343_g1,c81892_g1,c53269_g1,c40395_g1,c43824_g1 |
| Methane metabolism | ko00680 | 178 | c48241_g1,c9293_g1,c12991_g1,c110554_g1,c38356_g1,c41857_g1,c57341_g1,c109633_g1,c8857_g1,c73851_g1,c96551_g1,c39222_g1,c46281_g1,c31636_g1,c47044_g1,c42988_g1,c17506_g1,c68302_g1,c59715_g1,c100050_g1,c46404_g4,c25123_g2,c59201_g1,c24934_g1,c50179_g2,c98363_g1,c96933_g1,c46404_g1,c48970_g1,c68793_g1,c42135_g1,c99669_g1,c84047_g1,c71978_g1,c36049_g1,c29684_g1,c9747_g1,c18147_g1,c82202_g1,c97094_g1,c106435_g1,c96344_g1,c48427_g1,c72006_g1,c99582_g1,c110541_g1,c34733_g1,c93937_g1,c53873_g1,c109012_g1,c107366_g1,c66879_g1,c70719_g1,c34281_g1,c28542_g2,c48397_g1,c68919_g1,c78259_g1,c38169_g1,c109486_g1,c38897_g1,c76028_g1,c47044_g2,c60359_g1,c25123_g1,c20257_g1,c85117_g1,c56971_g1,c75473_g1,c42239_g1,c45885_g1,c22216_g1,c106540_g1,c79946_g1,c581_g2,c99342_g1,c109380_g1,c39848_g1,c40802_g1,c52882_g1,c48823_g1,c14995_g1,c108090_g1,c43166_g1,c75440_g1,c85195_g1,c44349_g1,c54743_g1,c54211_g1,c92052_g1,c15006_g1,c40100_g1,c44734_g1,c1939_g1,c50868_g1,c54175_g1,c48310_g1,c36720_g1,c42775_g1,c107473_g1,c57349_g1,c33782_g2,c75696_g1,c98122_g1,c39352_g1,c29224_g1,c989_g1,c9043_g1,c9285_g2,c48427_g2,c38803_g1,c57042_g1,c66208_g1,c63367_g1,c1434_g1,c25409_g1,c53687_g1,c57038_g1,c93775_g1,c92573_g1,c31470_g1,c58823_g1,c45977_g1,c19449_g1,c67546_g1,c54124_g1,c39440_g1,c9921_g1,c50458_g1,c109884_g1,c21011_g1,c19829_g2,c581_g1,c53386_g1,c77042_g1,c20257_g2,c46404_g3,c78609_g1,c49242_g1,c61298_g1,c62156_g1,c97042_g1,c9747_g2,c40769_g1,c98527_g1,c112079_g1,c72032_g1,c24487_g1,c61032_g1,c101270_g1,c67246_g1,c57532_g1,c59882_g1,c43733_g1,c45960_g1,c74256_g1,c68678_g1,c48827_g1,c96465_g1,c37431_g1,c82324_g1,c81892_g1,c42703_g1,c29198_g1,c57054_g1,c45852_g1,c88093_g1,c103790_g1,c58356_g1,c45838_g1,c43454_g1,c59464_g1,c78175_g1,c46404_g2,c89702_g1,c48310_g2,c49356_g1,c13770_g1 |
| Monoterpenoid biosynthesis | ko00902 | 8 | c69000_g1,c37480_g1,c6250_g1,c102410_g1,c19935_g1,c42038_g1,c65003_g1,c51723_g1 |
| Naphthalene degradation | ko00626 | 21 | c48241_g1,c68793_g1,c42135_g1,c84047_g1,c9747_g1,c93937_g1,c75473_g1,c22216_g1,c108090_g1,c16528_g1,c36418_g1,c92052_g1,c40100_g1,c107473_g1,c33782_g2,c45977_g1,c19829_g2,c9747_g2,c98527_g1,c46819_g1,c81892_g1 |
| N-Glycan biosynthesis | ko00510 | 82 | c42367_g1,c11324_g1,c65217_g1,c42055_g1,c47359_g1,c55605_g1,c87339_g1,c40679_g1,c29861_g1,c34532_g1,c297_g2,c49565_g1,c109206_g1,c107452_g1,c53467_g1,c38107_g1,c64583_g1,c53758_g1,c46139_g1,c56228_g1,c51478_g3,c88543_g1,c106225_g1,c94704_g1,c83103_g1,c55531_g1,c97777_g1,c105727_g1,c36347_g1,c93276_g1,c45932_g2,c46454_g1,c47138_g1,c40569_g1,c54919_g1,c11693_g1,c64187_g1,c67129_g1,c48108_g1,c40647_g1,c7078_g1,c46757_g1,c17986_g1,c52598_g1,c38194_g1,c62673_g1,c86564_g1,c34776_g1,c48199_g1,c48418_g1,c80944_g1,c45570_g1,c28880_g1,c82328_g1,c62491_g1,c51347_g1,c53980_g1,c51673_g1,c43326_g2,c89814_g1,c33707_g1,c37708_g2,c101193_g1,c66322_g1,c46588_g1,c75449_g1,c69249_g1,c94457_g1,c8895_g1,c55979_g1,c62421_g1,c91146_g1,c104206_g1,c54350_g1,c67459_g1,c74644_g1,c7901_g1,c42338_g1,c29002_g1,c51606_g1,c44940_g1,c42628_g1 |
| Nicotinate and nicotinamide metabolism | ko00760 | 43 | c5971_g1,c43840_g2,c100979_g1,c98225_g1,c85652_g1,c11242_g1,c95336_g1,c15791_g1,c41619_g1,c50219_g1,c8759_g1,c64486_g1,c51660_g1,c103762_g1,c48248_g1,c98925_g1,c47514_g1,c85371_g1,c950_g1,c52203_g1,c96420_g1,c15769_g1,c43645_g1,c56096_g1,c104508_g1,c11275_g1,c43840_g1,c81115_g1,c41308_g1,c43534_g1,c15637_g1,c36967_g1,c100405_g1,c93394_g1,c33975_g1,c50655_g1,c50500_g1,c950_g2,c34006_g1,c48237_g1,c43309_g2,c50131_g1,c67616_g1 |
| Nitrogen metabolism | ko00910 | 79 | c9479_g1,c41254_g1,c44716_g1,c44281_g1,c74905_g1,c52843_g1,c83019_g1,c92861_g1,c63776_g1,c57092_g1,c106878_g1,c59098_g1,c44498_g1,c42270_g1,c100459_g1,c112512_g1,c103866_g1,c83834_g1,c30625_g1,c95320_g1,c45692_g1,c9047_g1,c82300_g1,c96707_g1,c98706_g1,c102809_g1,c44181_g1,c45909_g1,c19247_g1,c112258_g1,c12319_g1,c100052_g1,c43714_g1,c54756_g1,c62622_g1,c58934_g1,c85429_g1,c109025_g1,c73295_g1,c14476_g1,c96233_g1,c50326_g1,c104264_g1,c64671_g1,c45058_g2,c99368_g1,c95064_g1,c44281_g2,c55062_g1,c50091_g1,c45816_g1,c17461_g1,c73101_g1,c3591_g1,c13264_g1,c45058_g1,c44700_g1,c9036_g1,c110638_g1,c22770_g1,c52226_g1,c83077_g1,c27278_g1,c34210_g1,c107010_g1,c17535_g1,c51145_g1,c38754_g1,c59692_g1,c50141_g1,c13249_g1,c93179_g1,c45231_g1,c58698_g1,c93711_g1,c61668_g1,c13249_g2,c60287_g1,c52505_g1 |
| Novobiocin biosynthesis | ko00401 | 11 | c47400_g1,c42740_g1,c73106_g1,c86188_g1,c36407_g1,c106159_g1,c91964_g1,c78598_g1,c34116_g1,c38946_g1,c41423_g1 |
| One carbon pool by folate | ko00670 | 49 | c41583_g1,c47875_g1,c92695_g1,c31636_g1,c59715_g1,c106981_g1,c98363_g1,c50722_g4,c100948_g1,c51231_g1,c55130_g1,c77717_g1,c34733_g1,c67728_g1,c16830_g1,c47939_g1,c109012_g1,c87086_g1,c44774_g1,c46898_g1,c42239_g1,c82266_g1,c14995_g1,c85195_g1,c86348_g1,c12491_g2,c15006_g1,c1939_g1,c76955_g1,c48545_g3,c68624_g1,c47635_g1,c63056_g1,c98122_g1,c86751_g1,c56114_g1,c26477_g1,c49004_g6,c49691_g1,c35754_g1,c47140_g1,c54124_g1,c65936_g1,c62156_g1,c52229_g3,c45960_g1,c29198_g1,c43454_g1,c48545_g2 |
| Other glycan degradation | ko00511 | 51 | c35829_g1,c97575_g1,c74497_g1,c48345_g1,c105174_g1,c29259_g1,c46421_g1,c86706_g1,c51051_g1,c48143_g1,c110570_g1,c67745_g1,c5465_g1,c50533_g1,c46476_g2,c50004_g1,c54133_g1,c49665_g1,c79990_g1,c65195_g1,c50018_g1,c73912_g1,c56842_g1,c51871_g1,c84191_g1,c43471_g1,c75981_g1,c49326_g3,c111150_g1,c61900_g1,c47903_g1,c61482_g1,c68640_g1,c34189_g1,c76512_g1,c43980_g1,c30455_g1,c6624_g1,c73787_g1,c13944_g1,c57866_g1,c95067_g1,c108220_g1,c96091_g1,c106393_g1,c49151_g1,c95809_g1,c89199_g1,c90510_g1,c29256_g2,c71625_g1 |
| Other types of O-glycan biosynthesis | ko00514 | 9 | c63062_g1,c105095_g1,c51648_g1,c51648_g2,c36070_g1,c16489_g1,c31476_g1,c37139_g1,c51190_g1 |
| Oxidative phosphorylation | ko00190 | 427 | c34735_g1,c90042_g1,c104220_g1,c95194_g1,c76954_g1,c10168_g1,c9517_g1,c17211_g1,c6604_g1,c84193_g1,c17113_g1,c60032_g1,c23083_g1,c75973_g1,c61064_g1,c3152_g1,c88181_g1,c105427_g1,c86494_g1,c81902_g1,c62196_g1,c94748_g1,c16948_g1,c7023_g1,c5241_g1,c80939_g1,c9214_g1,c96152_g1,c37459_g1,c104875_g1,c28591_g1,c51453_g8,c2841_g1,c40470_g1,c79303_g1,c97900_g1,c86894_g1,c84323_g1,c62555_g1,c28409_g1,c99822_g1,c83301_g1,c11910_g1,c18509_g1,c97317_g1,c53704_g1,c61602_g1,c52218_g2,c48499_g8,c65926_g1,c36632_g1,c98483_g1,c50720_g2,c105682_g1,c55308_g1,c48753_g1,c28894_g1,c13073_g1,c30615_g1,c106979_g1,c83916_g1,c88807_g1,c75542_g1,c103930_g1,c53271_g1,c92603_g1,c44289_g1,c48494_g1,c64920_g1,c73651_g1,c56181_g1,c106656_g1,c11792_g1,c15538_g1,c46405_g1,c31137_g1,c27333_g1,c48499_g6,c88393_g1,c2296_g1,c62789_g1,c40541_g1,c84731_g1,c68666_g1,c44822_g1,c11135_g1,c11911_g1,c27357_g1,c1628_g1,c53271_g2,c48922_g2,c39137_g1,c6086_g1,c61157_g1,c49376_g1,c68426_g1,c28261_g1,c57137_g1,c18647_g2,c29611_g1,c65581_g1,c18990_g2,c73076_g1,c98352_g1,c7622_g1,c78090_g1,c62118_g1,c69677_g1,c77072_g1,c4249_g1,c17915_g1,c41632_g1,c50749_g1,c110615_g1,c24691_g1,c17949_g1,c41066_g1,c14090_g1,c93047_g1,c53194_g1,c101000_g1,c50260_g1,c64608_g1,c78300_g1,c44937_g1,c5725_g1,c97305_g1,c9292_g1,c63324_g1,c83401_g1,c39227_g1,c30788_g1,c3915_g1,c51278_g2,c36970_g1,c24765_g1,c56831_g1,c52382_g6,c51476_g1,c61151_g1,c48918_g1,c74445_g1,c46051_g1,c71754_g1,c5271_g1,c40240_g1,c10937_g1,c97011_g1,c31127_g1,c56922_g1,c106417_g1,c87061_g1,c12071_g2,c109587_g1,c39385_g2,c52382_g1,c67587_g1,c72555_g1,c53040_g1,c90597_g1,c41754_g1,c46069_g1,c14180_g1,c108755_g1,c46944_g1,c60627_g1,c28919_g1,c23902_g1,c61909_g1,c53377_g1,c28562_g1,c61990_g1,c94865_g1,c46847_g1,c60632_g1,c29366_g1,c47794_g1,c88839_g1,c42167_g1,c78612_g1,c72669_g1,c92175_g1,c92805_g1,c73772_g1,c41812_g1,c51608_g2,c99750_g1,c112880_g1,c75573_g1,c52218_g3,c96215_g1,c105318_g1,c13993_g1,c32334_g1,c27328_g1,c79468_g1,c72305_g1,c72552_g1,c33826_g1,c103490_g1,c24691_g2,c15687_g1,c10611_g1,c35073_g1,c80156_g1,c17178_g1,c12725_g1,c31564_g1,c221_g1,c43157_g1,c5914_g1,c29152_g1,c87592_g1,c83478_g1,c17130_g1,c40254_g1,c20780_g1,c86867_g1,c82933_g1,c46446_g1,c50682_g1,c27013_g1,c98456_g1,c29538_g1,c102717_g1,c90632_g1,c24947_g1,c58824_g1,c55393_g1,c92961_g1,c68268_g1,c61622_g1,c28591_g2,c107114_g1,c27564_g1,c61368_g1,c72125_g1,c52917_g1,c106256_g1,c75828_g1,c70950_g1,c98162_g1,c84291_g1,c12872_g1,c17210_g1,c106212_g1,c28822_g1,c41028_g2,c52317_g5,c18990_g1,c41565_g1,c66264_g1,c30917_g1,c54708_g1,c56354_g1,c92807_g1,c29868_g1,c62266_g1,c112501_g1,c26063_g1,c49338_g2,c92951_g1,c74003_g1,c101824_g1,c62022_g1,c58011_g1,c86260_g1,c89605_g1,c16695_g1,c51278_g3,c107519_g1,c39227_g2,c53932_g1,c101459_g1,c10855_g1,c9519_g1,c31199_g1,c52382_g5,c110399_g1,c95203_g1,c48922_g5,c108293_g1,c37192_g1,c102383_g1,c94434_g1,c42052_g1,c105411_g1,c10633_g1,c44602_g1,c106377_g1,c3998_g1,c97915_g1,c86442_g1,c17294_g1,c89657_g1,c2421_g1,c75790_g1,c71718_g1,c46178_g1,c15584_g1,c57022_g1,c73464_g1,c71787_g1,c36255_g1,c41886_g1,c33801_g1,c103267_g1,c46723_g1,c1141_g1,c17283_g1,c39769_g1,c27303_g1,c48920_g1,c57755_g1,c43095_g1,c75929_g1,c98114_g1,c59129_g1,c79539_g1,c18443_g1,c19567_g1,c66467_g1,c52382_g3,c73196_g1,c27283_g1,c38274_g1,c47346_g1,c61560_g1,c52908_g1,c85012_g1,c36710_g1,c88846_g1,c97646_g1,c71873_g1,c75767_g1,c71632_g1,c78097_g1,c28543_g1,c74972_g1,c67598_g1,c107752_g1,c97772_g1,c83089_g1,c104037_g1,c2494_g1,c82420_g1,c15019_g1,c73802_g1,c16199_g1,c104392_g1,c67688_g1,c77154_g1,c45750_g1,c4548_g1,c76343_g1,c42308_g1,c22231_g1,c12814_g1,c71294_g1,c39823_g1,c46465_g1,c32556_g1,c95676_g1,c94053_g1,c6502_g1,c64420_g1,c34225_g1,c51932_g6,c108546_g1,c104382_g1,c11478_g1,c52015_g1,c81999_g1,c86582_g1,c9150_g1,c39644_g1,c50012_g2,c62331_g1,c29694_g1,c1164_g1,c95449_g1,c103443_g1,c46403_g1,c61300_g1,c43979_g1,c56674_g1,c76908_g1,c56271_g1,c73806_g1,c71628_g1,c48352_g1,c99838_g1,c93037_g1,c52510_g3,c78711_g1,c39385_g1,c9525_g1,c72030_g1,c93723_g1,c82205_g1,c68443_g1,c51453_g3,c39644_g2,c46242_g1,c18647_g1,c73323_g1,c106734_g1,c82717_g1,c29505_g1,c108219_g1,c80887_g1,c44924_g1,c88306_g1,c57606_g1,c73672_g1,c108352_g1,c82188_g1,c110997_g1,c4027_g1,c34164_g1,c28432_g1,c100009_g1,c16903_g1,c30814_g1,c15911_g1,c68770_g1,c45920_g1 |
| Pantothenate and CoA biosynthesis | ko00770 | 70 | c6029_g1,c44302_g2,c95034_g1,c35789_g1,c16964_g1,c71149_g1,c79442_g1,c95068_g1,c17463_g1,c13175_g1,c105038_g1,c46822_g1,c71763_g1,c82486_g1,c55416_g1,c84946_g1,c43045_g1,c45766_g2,c66882_g1,c112308_g1,c66158_g1,c67039_g1,c49098_g3,c109043_g1,c47003_g1,c78560_g1,c61242_g1,c39358_g1,c66195_g1,c4317_g1,c65042_g1,c39637_g1,c64274_g1,c104555_g1,c93338_g1,c66239_g1,c86070_g1,c47514_g1,c74727_g1,c85371_g1,c34013_g1,c100270_g1,c71641_g1,c32058_g1,c43327_g1,c87783_g1,c44344_g1,c9286_g1,c67249_g1,c4317_g2,c56741_g1,c75192_g1,c28685_g1,c50383_g1,c61619_g1,c42387_g1,c95977_g1,c94269_g1,c29000_g1,c28557_g1,c72642_g1,c86327_g1,c48449_g1,c41124_g1,c85083_g1,c9318_g1,c46331_g1,c46851_g1,c106827_g1,c50316_g1 |
| Pentose and glucuronate interconversions | ko00040 | 138 | c109984_g1,c10715_g1,c89219_g1,c48673_g1,c4233_g1,c40778_g1,c47621_g1,c48536_g2,c47365_g1,c102313_g1,c86580_g1,c82509_g1,c5959_g1,c103747_g1,c24859_g1,c28618_g1,c106670_g1,c72765_g1,c94683_g1,c40766_g1,c24020_g1,c71270_g1,c47139_g1,c61975_g1,c64213_g1,c66185_g1,c97539_g1,c75402_g1,c62190_g1,c73811_g1,c28808_g1,c25280_g1,c24869_g1,c112708_g1,c48536_g3,c34608_g1,c41774_g1,c33934_g1,c99377_g1,c16621_g1,c25044_g1,c88691_g1,c74356_g1,c38151_g1,c25044_g2,c41057_g1,c24454_g1,c106108_g1,c39522_g1,c46517_g2,c50235_g1,c55255_g1,c83930_g1,c21811_g2,c4067_g1,c75055_g1,c61823_g1,c39033_g1,c95382_g1,c15366_g1,c113241_g1,c102169_g1,c24630_g1,c39602_g1,c29553_g1,c7310_g1,c92847_g1,c82548_g1,c46365_g1,c44736_g1,c111846_g1,c40831_g1,c82664_g1,c65460_g1,c49723_g1,c49231_g1,c47370_g1,c52336_g2,c46306_g1,c40831_g2,c54597_g1,c58316_g1,c42366_g1,c62983_g1,c45085_g1,c49007_g1,c96453_g1,c45764_g1,c63275_g1,c43212_g1,c74035_g1,c35055_g1,c31452_g1,c102364_g1,c12036_g1,c49171_g1,c26299_g1,c43880_g2,c9971_g1,c74312_g1,c34753_g1,c37410_g1,c85425_g1,c57956_g1,c81861_g1,c98060_g1,c62978_g1,c99066_g1,c35846_g1,c26165_g1,c74875_g1,c51775_g1,c103742_g1,c47590_g1,c50086_g1,c92816_g1,c26258_g1,c60708_g1,c46517_g1,c76192_g1,c1398_g1,c83915_g1,c89815_g1,c41057_g2,c90369_g1,c24632_g1,c34529_g2,c86421_g1,c7528_g1,c45860_g1,c25367_g3,c46006_g1,c31549_g1,c44126_g1,c16785_g1,c71874_g1,c21811_g1,c31739_g1 |
| Pentose phosphate pathway | ko00030 | 135 | c109984_g1,c12991_g1,c44170_g1,c41857_g1,c29194_g1,c88999_g1,c46994_g1,c52428_g1,c8202_g2,c38078_g1,c39222_g1,c68591_g1,c85940_g1,c47044_g1,c96653_g1,c59059_g1,c56675_g1,c77184_g1,c47534_g1,c17039_g1,c103747_g1,c48970_g1,c99904_g1,c84428_g1,c42371_g1,c76102_g1,c14450_g1,c89741_g1,c99880_g1,c64213_g1,c38793_g1,c72023_g1,c82202_g1,c72006_g1,c43637_g1,c99582_g1,c64130_g1,c34281_g1,c108530_g1,c6653_g2,c51658_g3,c61834_g1,c12471_g1,c38169_g1,c47044_g2,c103950_g1,c81276_g1,c72186_g1,c6653_g1,c81385_g1,c77371_g1,c103120_g1,c47283_g1,c109939_g1,c45885_g1,c81638_g1,c41255_g1,c65414_g1,c39848_g1,c40802_g1,c74668_g1,c52882_g1,c71759_g1,c74204_g1,c43166_g1,c75440_g1,c68586_g1,c54743_g1,c93181_g1,c36510_g1,c44734_g1,c54175_g1,c28265_g1,c48310_g1,c36720_g1,c34561_g1,c65408_g1,c84312_g1,c107669_g1,c55616_g1,c99079_g1,c57149_g1,c61561_g1,c50386_g1,c35331_g1,c66749_g1,c25409_g1,c63275_g1,c57038_g1,c56604_g1,c87748_g1,c12036_g1,c31470_g1,c17869_g1,c62502_g1,c43880_g2,c100365_g1,c43044_g1,c8202_g1,c72608_g1,c39440_g1,c109884_g1,c57871_g1,c49242_g1,c13374_g1,c110906_g1,c40769_g1,c58536_g1,c72127_g1,c61032_g1,c46480_g1,c36681_g1,c55637_g1,c99146_g1,c108002_g1,c43733_g1,c82314_g1,c94934_g1,c68678_g1,c48827_g1,c37431_g1,c82324_g1,c76091_g1,c85095_g1,c10950_g1,c47764_g1,c47115_g1,c40176_g1,c62957_g1,c15105_g1,c43883_g1,c48310_g2,c63024_g1,c45578_g2,c13770_g1 |
| Peptidoglycan biosynthesis | ko00550 | 4 | c82532_g1,c95069_g1,c48792_g1,c36703_g1 |
| Phenylalanine | ko00400 | 90 | c76030_g1,c110883_g1,c52441_g2,c61635_g1,c42733_g1,c47400_g1,c42740_g1,c56073_g1,c73106_g1,c106588_g1,c86188_g1,c19206_g1,c60397_g1,c36407_g1,c45680_g1,c80695_g1,c80123_g1,c73578_g1,c97967_g1,c40621_g1,c17536_g2,c104105_g1,c83241_g1,c75603_g1,c38070_g1,c45803_g1,c80270_g1,c12390_g1,c1350_g1,c70597_g1,c106159_g1,c87593_g1,c49599_g1,c29286_g1,c57611_g1,c12938_g1,c24536_g1,c35032_g1,c105807_g1,c12938_g2,c9362_g1,c28212_g1,c48370_g1,c35188_g1,c91964_g1,c50389_g1,c71900_g1,c93189_g1,c3870_g1,c77408_g1,c48879_g1,c84626_g1,c104622_g1,c45923_g1,c78598_g1,c100312_g1,c52622_g3,c42250_g1,c48628_g1,c73923_g1,c42918_g1,c29716_g1,c52622_g2,c46700_g1,c49306_g2,c61163_g1,c34116_g1,c43501_g1,c4459_g1,c1753_g1,c43115_g1,c72220_g1,c44832_g2,c82144_g1,c67532_g1,c15887_g1,c38946_g1,c106754_g1,c67501_g1,c48708_g1,c27664_g1,c45648_g1,c41423_g1,c110574_g1,c38735_g1,c63265_g1,c40283_g2,c74030_g1,c48624_g1,c76988_g1 |
| Phenylalanine metabolism | ko00360 | 160 | c65424_g1,c110883_g1,c39949_g1,c61635_g1,c9632_g1,c73876_g1,c47400_g1,c79994_g1,c42740_g1,c56073_g1,c73106_g1,c106588_g1,c72052_g1,c38304_g1,c86188_g1,c19206_g1,c60397_g1,c36407_g1,c104156_g1,c52903_g1,c66186_g1,c15296_g1,c80695_g1,c47185_g1,c73578_g1,c63280_g1,c40621_g1,c14782_g1,c26845_g1,c95199_g1,c69393_g1,c44511_g1,c19209_g1,c57994_g1,c82179_g1,c19431_g1,c14092_g1,c35123_g1,c35895_g1,c45291_g1,c40617_g1,c80270_g1,c50870_g1,c103501_g1,c109967_g1,c91920_g1,c63048_g1,c1350_g1,c57644_g1,c43182_g1,c51616_g1,c46486_g1,c96072_g1,c15202_g1,c106159_g1,c29794_g1,c51428_g3,c30728_g1,c36513_g1,c39997_g1,c28892_g1,c49643_g1,c56783_g1,c8170_g1,c108213_g1,c57611_g1,c12938_g1,c18743_g1,c24536_g1,c105678_g1,c12938_g2,c49078_g2,c57417_g1,c37258_g1,c91685_g1,c28212_g1,c9103_g1,c91964_g1,c4521_g1,c53527_g1,c50389_g1,c77399_g1,c46568_g1,c47995_g2,c93189_g1,c94620_g1,c46272_g1,c104217_g1,c40850_g1,c103522_g1,c84626_g1,c72626_g1,c104622_g1,c2594_g1,c44892_g1,c78598_g1,c56723_g1,c58660_g1,c77962_g1,c346_g1,c54835_g1,c57934_g1,c73923_g1,c109680_g1,c36592_g1,c49065_g1,c94900_g1,c3992_g1,c34116_g1,c103084_g1,c46599_g1,c78151_g1,c43501_g1,c14751_g1,c44877_g1,c1753_g1,c27191_g1,c33138_g1,c54005_g1,c28775_g2,c84168_g1,c28404_g1,c38946_g1,c35949_g1,c49078_g1,c9351_g1,c48882_g1,c37487_g1,c48317_g1,c47618_g1,c41451_g1,c38056_g1,c47831_g1,c103441_g1,c93451_g1,c108375_g1,c47761_g2,c13468_g1,c36651_g1,c11012_g1,c84599_g1,c17108_g1,c41423_g1,c40770_g1,c110574_g1,c42253_g1,c34446_g1,c28775_g1,c63265_g1,c43127_g1,c62961_g1,c74030_g1,c38177_g1,c32423_g1,c51868_g1,c48624_g1,c110530_g1,c29176_g1,c50461_g1,c49223_g1 |
| Phenylpropanoid biosynthesis | ko00940 | 227 | c67847_g1,c75871_g1,c39949_g1,c26992_g1,c1193_g1,c34485_g1,c43433_g1,c9632_g1,c73876_g1,c67084_g1,c79994_g1,c105092_g1,c40346_g1,c72052_g1,c38304_g1,c4006_g1,c4232_g1,c51832_g2,c29660_g1,c74027_g1,c104156_g1,c52903_g1,c45733_g1,c66186_g1,c63121_g1,c50289_g1,c45667_g1,c26046_g1,c63280_g1,c103806_g1,c26845_g1,c55564_g1,c87450_g1,c69393_g1,c44511_g1,c63646_g1,c39743_g2,c53703_g1,c48733_g1,c57994_g1,c105620_g1,c82179_g1,c19431_g1,c76713_g1,c26805_g1,c14092_g1,c35123_g1,c36431_g1,c83090_g1,c35895_g1,c97104_g1,c40617_g1,c99912_g1,c37640_g1,c103501_g1,c6312_g1,c109967_g1,c91920_g1,c63048_g1,c91657_g1,c57644_g1,c43182_g1,c112628_g1,c60593_g1,c55215_g1,c46876_g1,c51877_g1,c84333_g1,c9748_g1,c51616_g1,c46090_g1,c46486_g1,c96072_g1,c15202_g1,c43801_g1,c73306_g1,c105966_g1,c29794_g1,c36634_g1,c51428_g3,c83030_g1,c30728_g1,c91431_g1,c28892_g1,c49643_g1,c45319_g1,c35474_g1,c8170_g1,c32367_g1,c108213_g1,c47326_g2,c18743_g1,c93747_g1,c53682_g1,c105678_g1,c28388_g2,c43201_g1,c67684_g1,c49078_g2,c57417_g1,c84218_g1,c37258_g1,c330_g1,c91685_g1,c9103_g1,c39667_g1,c48733_g4,c107170_g1,c81102_g1,c77399_g1,c31168_g1,c72397_g1,c40728_g1,c47995_g2,c94620_g1,c52483_g1,c92777_g1,c40522_g1,c46272_g1,c103333_g1,c51832_g1,c44888_g1,c104217_g1,c52507_g1,c103522_g1,c26184_g1,c99744_g1,c35243_g1,c2594_g1,c105150_g1,c83163_g1,c44892_g1,c71906_g1,c72777_g1,c44966_g1,c56723_g1,c58660_g1,c105463_g1,c77962_g1,c346_g1,c54835_g1,c109680_g1,c36592_g1,c103744_g1,c32052_g1,c94900_g1,c46907_g1,c3992_g1,c11407_g1,c106634_g1,c60986_g1,c76267_g1,c87733_g1,c78151_g1,c50637_g1,c14751_g1,c113150_g1,c44887_g1,c45598_g1,c44877_g1,c24369_g1,c27191_g1,c51615_g1,c33138_g1,c46907_g2,c84168_g1,c28404_g1,c58915_g1,c47634_g1,c35949_g1,c2063_g1,c58065_g1,c49078_g1,c28083_g1,c48149_g1,c54568_g1,c99550_g1,c46907_g3,c37487_g1,c74253_g1,c83161_g1,c41451_g1,c38056_g1,c47831_g1,c46108_g1,c103441_g1,c9174_g1,c93451_g1,c108375_g1,c47761_g2,c109191_g1,c13468_g1,c36651_g1,c48740_g1,c100652_g1,c11012_g1,c47326_g1,c86498_g1,c62392_g1,c17108_g1,c73114_g1,c40770_g1,c45319_g2,c107457_g1,c4381_g1,c42253_g1,c34446_g1,c66108_g1,c51227_g1,c66969_g1,c92463_g1,c43127_g1,c33348_g1,c62961_g1,c65760_g1,c17965_g1,c30577_g1,c52365_g1,c38177_g1,c46529_g1,c110530_g1,c11015_g1,c783_g1,c29176_g1,c50461_g1,c49223_g1,c4094_g1 |
| Phosphonate and phosphinate metabolism | ko00440 | 9 | c67584_g1,c36266_g1,c38024_g1,c74694_g1,c54517_g1,c50515_g1,c86222_g1,c45281_g1,c36220_g1 |
| Photosynthesis | ko00195 | 51 | c5241_g1,c40470_g1,c42603_g2,c57680_g1,c4539_g1,c28778_g1,c104567_g1,c51273_g5,c36940_g1,c37040_g1,c50520_g1,c44289_g1,c15538_g1,c54120_g1,c84731_g1,c19507_g1,c30057_g1,c50749_g1,c49460_g1,c36516_g1,c103373_g1,c61160_g1,c15678_g1,c39250_g1,c10839_g1,c93468_g1,c31111_g1,c41278_g1,c27564_g1,c61368_g1,c61569_g1,c41565_g1,c36468_g1,c43560_g1,c94434_g1,c95724_g1,c29236_g1,c40106_g1,c39210_g1,c51273_g7,c18289_g1,c36710_g1,c16199_g1,c43897_g1,c48082_g2,c48886_g1,c47636_g1,c38300_g1,c52964_g1,c52902_g1,c17118_g1 |
| Photosynthesis - antenna proteins | ko00196 | 15 | c48747_g1,c40955_g1,c40554_g1,c52879_g1,c31473_g1,c33603_g1,c9134_g1,c43342_g1,c47742_g1,c44903_g1,c92809_g1,c26239_g1,c43608_g2,c48508_g1,c43126_g1 |
| Polycyclic aromatic hydrocarbon degradation | ko00624 | 42 | c62744_g1,c49461_g1,c44873_g1,c77300_g1,c44494_g1,c63072_g1,c52112_g1,c57881_g1,c52112_g2,c24471_g1,c54530_g1,c95509_g1,c52112_g3,c110831_g1,c11088_g1,c52941_g1,c54345_g1,c83889_g1,c37416_g1,c25007_g2,c99792_g1,c108064_g1,c36408_g1,c33474_g1,c43675_g2,c50878_g1,c82195_g1,c52985_g1,c11795_g1,c2765_g1,c46140_g1,c10651_g1,c25106_g1,c71527_g1,c10846_g1,c110641_g1,c37774_g1,c48658_g1,c71682_g1,c27078_g1,c41788_g1,c82973_g1 |
| Porphyrin and chlorophyll metabolism | ko00860 | 90 | c26331_g1,c43598_g1,c48896_g1,c46154_g1,c89235_g1,c76943_g1,c54055_g1,c30509_g1,c44414_g1,c46362_g1,c44757_g1,c88430_g1,c43755_g1,c50720_g2,c46703_g1,c48753_g1,c81058_g1,c45206_g1,c93242_g1,c99370_g1,c76870_g1,c49826_g2,c32296_g1,c45757_g1,c47968_g2,c38697_g1,c38160_g1,c43313_g1,c97011_g1,c51937_g1,c45307_g1,c49204_g1,c62514_g1,c36048_g1,c42477_g1,c100308_g1,c45066_g1,c46369_g1,c31358_g1,c690_g1,c39602_g1,c47385_g1,c46446_g1,c77919_g1,c1413_g1,c77899_g1,c43943_g1,c43358_g1,c54071_g1,c51057_g1,c44103_g2,c8898_g1,c86044_g1,c66053_g1,c49856_g1,c78749_g1,c40447_g1,c27000_g1,c107099_g1,c97057_g1,c82568_g1,c45714_g1,c85118_g1,c61258_g1,c37410_g1,c88846_g1,c40833_g1,c50163_g1,c76097_g1,c78121_g1,c62856_g1,c99066_g1,c107230_g1,c75956_g1,c58511_g1,c29096_g1,c76614_g1,c95928_g1,c60477_g1,c71582_g1,c50646_g2,c52924_g1,c45722_g1,c46605_g1,c29395_g1,c48715_g1,c84765_g1,c76699_g1,c100009_g1,c99584_g1 |
| Propanoate metabolism | ko00640 | 194 | c52678_g2,c8198_g1,c89219_g1,c110876_g1,c94070_g1,c77515_g1,c64170_g1,c51829_g1,c11497_g1,c47621_g1,c83165_g1,c9421_g1,c48902_g1,c13346_g1,c48536_g2,c35216_g1,c68302_g1,c52533_g4,c52798_g4,c62877_g1,c110089_g1,c77947_g1,c726_g1,c47365_g1,c37769_g1,c52818_g1,c76596_g1,c86580_g1,c47420_g1,c18801_g1,c69486_g1,c92658_g1,c84449_g1,c106670_g1,c43009_g2,c81982_g1,c85139_g1,c99669_g1,c52729_g1,c40766_g1,c50954_g5,c99712_g1,c49366_g9,c31007_g1,c47384_g1,c36049_g1,c89320_g1,c72878_g1,c97539_g1,c75402_g1,c62190_g1,c47244_g1,c73811_g1,c47937_g2,c99408_g1,c45866_g1,c77190_g1,c48536_g3,c85100_g1,c34608_g1,c80976_g1,c52061_g2,c91515_g1,c39032_g1,c61692_g1,c88691_g1,c33831_g1,c103399_g1,c1906_g1,c48257_g1,c78330_g1,c12050_g1,c109689_g1,c41057_g1,c53308_g1,c55541_g1,c27943_g1,c27323_g1,c75123_g1,c12162_g1,c27619_g1,c50235_g1,c83324_g1,c581_g2,c99342_g1,c50796_g9,c36907_g1,c837_g1,c50954_g2,c77688_g1,c85941_g1,c75055_g1,c61823_g1,c105469_g1,c57634_g1,c50584_g1,c96322_g1,c58116_g1,c48999_g1,c46948_g1,c64933_g1,c102800_g1,c29553_g1,c43444_g2,c49366_g5,c98660_g1,c94165_g1,c66906_g1,c85135_g1,c101352_g1,c106416_g1,c45230_g1,c93748_g1,c14188_g1,c49723_g1,c34317_g1,c103615_g1,c67839_g1,c104126_g1,c82477_g1,c75511_g1,c52336_g2,c58316_g1,c30815_g1,c45085_g1,c50138_g2,c77787_g1,c54776_g1,c52434_g1,c102459_g1,c52030_g1,c84199_g1,c460_g1,c102419_g1,c53329_g1,c93775_g1,c85240_g1,c102364_g1,c27356_g1,c44637_g1,c92573_g1,c46119_g1,c52858_g6,c102188_g1,c99135_g1,c25261_g1,c44681_g1,c29591_g1,c36340_g1,c34019_g1,c18177_g1,c67546_g1,c73176_g1,c581_g1,c53386_g1,c39207_g1,c46277_g1,c62374_g1,c87808_g1,c58779_g1,c111270_g1,c62978_g1,c47598_g1,c42128_g1,c48105_g1,c52271_g2,c37822_g1,c73456_g1,c72872_g1,c111518_g1,c46544_g1,c54443_g1,c69630_g1,c83437_g1,c59896_g1,c76192_g1,c46119_g2,c1398_g1,c52466_g1,c49366_g1,c83915_g1,c89815_g1,c41057_g2,c90369_g1,c110160_g1,c63958_g1,c48230_g1,c36810_g1,c46775_g1,c51499_g2,c62283_g1,c31759_g1,c103549_g1,c87279_g1 |
| Purine metabolism | ko00230 | 321 | c95746_g1,c97051_g1,c54289_g1,c44170_g1,c89549_g1,c100401_g1,c88999_g1,c95531_g1,c95720_g1,c40943_g1,c100811_g1,c43840_g2,c109760_g1,c47393_g1,c48127_g2,c103519_g1,c17547_g1,c56099_g1,c3854_g1,c49041_g1,c1954_g1,c49880_g1,c50327_g6,c48844_g2,c109324_g1,c64555_g1,c33816_g1,c83746_g1,c94579_g1,c85703_g1,c43221_g1,c52039_g1,c52005_g3,c55119_g1,c73448_g1,c53306_g1,c52344_g5,c103538_g1,c53275_g1,c27600_g1,c51337_g1,c77184_g1,c47534_g1,c215_g1,c51153_g3,c100364_g1,c50722_g4,c60649_g1,c19297_g1,c35641_g1,c77288_g1,c110353_g1,c78234_g1,c11071_g1,c97623_g1,c61871_g1,c37799_g1,c110238_g1,c10225_g1,c49636_g1,c100241_g1,c98539_g1,c104888_g1,c60347_g1,c14450_g1,c76378_g1,c90649_g1,c78797_g1,c38201_g1,c77717_g1,c33339_g1,c92076_g1,c46151_g1,c57347_g1,c73495_g1,c29331_g1,c83142_g1,c77055_g1,c101923_g1,c72989_g1,c47213_g1,c39195_g1,c41161_g1,c51735_g1,c62495_g1,c76071_g1,c92486_g1,c42746_g1,c89508_g1,c50327_g5,c3949_g1,c88530_g1,c106149_g1,c96722_g1,c74934_g1,c57931_g1,c788_g1,c47668_g1,c72361_g1,c26133_g1,c99129_g1,c51153_g2,c48132_g2,c95100_g1,c37733_g1,c24358_g1,c62792_g1,c35699_g1,c95319_g1,c88666_g1,c36570_g1,c103573_g1,c103120_g1,c47283_g1,c109268_g1,c39597_g1,c92670_g1,c104730_g1,c42689_g1,c51660_g1,c62719_g1,c81638_g1,c48444_g1,c29742_g1,c47074_g1,c82185_g1,c14781_g1,c72147_g1,c40339_g1,c52388_g3,c48395_g1,c92307_g1,c41922_g1,c51932_g5,c57602_g1,c74204_g1,c99024_g1,c37207_g1,c40967_g1,c47111_g1,c63836_g1,c88998_g1,c11100_g2,c83003_g1,c103738_g1,c52074_g1,c59436_g1,c43066_g1,c47514_g1,c52493_g1,c57387_g1,c85371_g1,c97068_g1,c35517_g1,c61778_g1,c52486_g1,c50825_g2,c63986_g1,c51107_g1,c65126_g1,c20347_g1,c11100_g1,c50510_g1,c53666_g1,c49336_g1,c98175_g1,c41898_g1,c65825_g1,c79843_g1,c70337_g1,c76955_g1,c87273_g1,c92888_g1,c45557_g1,c26379_g1,c109184_g1,c80248_g1,c25712_g1,c10784_g1,c82523_g1,c48988_g2,c55616_g1,c91150_g1,c46164_g2,c39493_g1,c59511_g1,c57919_g1,c6580_g1,c86751_g1,c109730_g1,c89662_g1,c38372_g1,c9500_g1,c81878_g1,c46200_g1,c53147_g1,c26021_g1,c49983_g2,c107316_g1,c110063_g1,c63587_g1,c66749_g1,c66170_g1,c109205_g1,c82965_g1,c82103_g1,c26477_g1,c64849_g1,c108921_g1,c40560_g1,c9491_g1,c40460_g1,c36262_g1,c51475_g1,c39117_g2,c94632_g1,c62106_g1,c41013_g1,c50841_g1,c25130_g1,c8465_g1,c91366_g1,c105775_g1,c38259_g1,c34493_g1,c56096_g1,c46293_g2,c43970_g1,c109955_g1,c46159_g1,c83369_g1,c44636_g1,c43840_g1,c35754_g1,c67738_g1,c56336_g1,c75248_g1,c110788_g1,c14789_g1,c845_g1,c60503_g1,c56860_g1,c34158_g1,c91919_g1,c64743_g1,c88883_g1,c16610_g1,c40775_g1,c59078_g1,c94764_g1,c6841_g1,c57770_g1,c83832_g1,c47270_g1,c65936_g1,c9078_g1,c102038_g1,c52229_g3,c30653_g1,c67906_g1,c50016_g1,c4230_g1,c109908_g1,c51064_g1,c77864_g1,c105046_g1,c37556_g1,c29303_g1,c46480_g1,c36681_g1,c52173_g1,c38423_g1,c24721_g1,c40852_g1,c24829_g1,c47893_g1,c61214_g2,c105690_g1,c44698_g1,c49231_g2,c98995_g1,c33975_g1,c34020_g1,c58199_g1,c44573_g1,c49341_g3,c60409_g1,c112465_g1,c29148_g1,c69226_g1,c104871_g1,c65713_g1,c78552_g1,c83336_g1,c96667_g1,c97658_g1,c50625_g1,c37462_g1,c68744_g1,c13104_g1,c51562_g1,c26284_g1,c104305_g1,c40838_g1,c50500_g1,c58304_g1,c27214_g1,c9078_g2,c66320_g1,c97161_g1,c73623_g1,c87176_g1,c56245_g1,c31515_g1,c34006_g1,c108828_g1,c43309_g2,c50131_g1,c63024_g1,c49427_g1,c49414_g1 |
| Pyrimidine metabolism | ko00240 | 230 | c95746_g1,c97051_g1,c64692_g1,c1696_g1,c51194_g1,c89549_g1,c95531_g1,c48472_g2,c50984_g1,c95720_g1,c43840_g2,c103929_g1,c103519_g1,c40953_g1,c56099_g1,c98613_g1,c341_g1,c33871_g1,c86963_g1,c49041_g1,c103062_g1,c1954_g1,c49880_g1,c50327_g6,c104788_g1,c42172_g1,c94579_g1,c85703_g1,c43221_g1,c52039_g1,c59919_g1,c55119_g1,c17463_g1,c73448_g1,c44252_g1,c13175_g1,c103538_g1,c107780_g1,c37009_g1,c51337_g1,c41264_g1,c106981_g1,c100658_g1,c20444_g1,c46822_g1,c35641_g1,c44668_g1,c11071_g1,c97623_g1,c66289_g1,c82486_g1,c10225_g1,c98762_g1,c49636_g1,c86834_g1,c66882_g1,c38201_g1,c84733_g1,c33339_g1,c75982_g1,c73495_g1,c78560_g1,c39195_g1,c47798_g1,c62495_g1,c47939_g1,c45482_g1,c44585_g1,c15791_g1,c92486_g1,c42746_g1,c50327_g5,c4317_g1,c74934_g1,c112464_g1,c65677_g1,c37733_g1,c24358_g1,c41515_g1,c35699_g1,c88666_g1,c97752_g1,c36570_g1,c50369_g1,c109268_g1,c78249_g1,c39597_g1,c92670_g1,c104730_g1,c41350_g1,c51660_g1,c48444_g1,c14781_g1,c40339_g1,c52388_g3,c105015_g1,c103762_g1,c57572_g1,c48395_g1,c92307_g1,c41922_g1,c57602_g1,c47111_g1,c52074_g1,c88754_g1,c96732_g1,c86348_g1,c100270_g1,c61778_g1,c52486_g1,c63986_g1,c51107_g1,c65126_g1,c41898_g1,c70337_g1,c92888_g1,c26379_g1,c64696_g1,c45202_g1,c96341_g1,c25712_g1,c48988_g2,c77182_g1,c46164_g2,c59511_g1,c57919_g1,c77954_g1,c94337_g1,c89662_g1,c38372_g1,c46200_g1,c53147_g1,c26021_g1,c49983_g2,c42792_g1,c110063_g1,c109205_g1,c82103_g1,c64849_g1,c108921_g1,c9491_g1,c36262_g1,c55233_g1,c62106_g1,c50841_g1,c91366_g1,c45870_g1,c113085_g1,c4317_g2,c49004_g6,c38259_g1,c34493_g1,c56096_g1,c45272_g1,c52432_g1,c78342_g1,c46159_g1,c56741_g1,c43840_g1,c56336_g1,c75248_g1,c845_g1,c81115_g1,c60503_g1,c56860_g1,c91919_g1,c81172_g1,c64743_g1,c53845_g1,c16610_g1,c18075_g1,c37856_g1,c67775_g1,c84712_g1,c57770_g1,c83832_g1,c42545_g1,c102568_g1,c4230_g1,c109908_g1,c51064_g1,c84542_g1,c77864_g1,c105046_g1,c65388_g1,c37556_g1,c98365_g1,c29303_g1,c31753_g1,c36967_g1,c107567_g1,c40819_g1,c52173_g1,c89447_g1,c73187_g1,c40852_g1,c61214_g2,c49231_g2,c74196_g1,c98995_g1,c33975_g1,c34020_g1,c49341_g3,c112465_g1,c65713_g1,c78552_g1,c96667_g1,c108519_g1,c50625_g1,c13104_g1,c51562_g1,c107150_g1,c104305_g1,c40838_g1,c50500_g1,c27214_g1,c97161_g1,c84642_g1,c73623_g1,c31515_g1,c99974_g1,c34006_g1,c108828_g1,c73877_g1,c43309_g2,c38689_g1,c50131_g1,c49427_g1,c50316_g1,c109968_g1 |
| Pyruvate metabolism | ko00620 | 272 | c19122_g1,c72144_g1,c110554_g1,c53491_g1,c18240_g1,c89219_g1,c55505_g1,c45370_g1,c14873_g1,c77859_g1,c64170_g1,c109633_g1,c51829_g1,c94923_g1,c48127_g2,c73851_g1,c102864_g1,c96407_g1,c9460_g1,c47621_g1,c48902_g1,c48844_g2,c48536_g2,c17506_g1,c45979_g1,c35216_g1,c68302_g1,c93517_g1,c110089_g1,c98311_g1,c52344_g5,c47365_g1,c27600_g1,c92790_g1,c86580_g1,c73707_g1,c30759_g1,c65074_g1,c66648_g1,c19297_g1,c84449_g1,c21303_g1,c45242_g1,c61871_g1,c37799_g1,c88843_g1,c106670_g1,c76146_g1,c99669_g1,c58217_g1,c40766_g1,c108204_g1,c31007_g1,c36049_g1,c61975_g1,c97539_g1,c75402_g1,c62190_g1,c9460_g2,c47244_g1,c73811_g1,c40278_g1,c78140_g1,c47937_g2,c46151_g1,c93137_g1,c99408_g1,c86727_g1,c53627_g1,c51735_g1,c45866_g1,c77190_g1,c25743_g1,c66879_g1,c82216_g1,c48536_g3,c106025_g1,c85100_g1,c92836_g1,c70719_g1,c34608_g1,c47022_g1,c48397_g1,c80976_g1,c33934_g1,c40011_g2,c67729_g1,c47668_g1,c88691_g1,c88505_g1,c26_g2,c74796_g1,c37201_g1,c48132_g2,c82112_g1,c56845_g1,c108398_g1,c23285_g1,c41057_g1,c103573_g1,c55541_g1,c83757_g1,c52142_g1,c106108_g1,c75123_g1,c50235_g1,c581_g2,c99342_g1,c85447_g1,c837_g1,c47074_g1,c21811_g2,c93721_g1,c85941_g1,c75055_g1,c61823_g1,c105469_g1,c57634_g1,c53631_g1,c19122_g2,c4238_g1,c66953_g1,c41865_g1,c107227_g1,c13859_g1,c102169_g1,c24562_g1,c64933_g1,c57387_g1,c102800_g1,c29553_g1,c49145_g1,c100202_g1,c21888_g1,c51219_g2,c21314_g1,c49902_g2,c101267_g1,c14188_g1,c82664_g1,c65460_g1,c79843_g1,c49723_g1,c42775_g1,c104067_g1,c103615_g1,c67839_g1,c104126_g1,c51740_g1,c82523_g1,c82477_g1,c86489_g1,c99736_g1,c52336_g2,c54243_g1,c58316_g1,c49156_g2,c46725_g1,c62983_g1,c109895_g1,c29224_g1,c45085_g1,c50138_g2,c90146_g1,c38282_g1,c71923_g1,c74433_g1,c19122_g3,c26628_g1,c45764_g1,c1434_g1,c41440_g1,c45902_g2,c61150_g1,c93775_g1,c30117_g1,c45578_g1,c102364_g1,c44637_g1,c92573_g1,c46119_g1,c99135_g1,c44229_g1,c27469_g1,c47200_g1,c64693_g1,c101332_g1,c47203_g2,c11851_g1,c64429_g1,c50299_g1,c104284_g1,c49091_g1,c57426_g1,c67546_g1,c49902_g1,c6656_g1,c12516_g1,c50458_g1,c581_g1,c53386_g1,c62374_g1,c26_g1,c78609_g1,c85425_g1,c48865_g1,c18302_g1,c48964_g1,c58779_g1,c50016_g1,c40011_g1,c109007_g1,c110027_g1,c96911_g1,c98060_g1,c62978_g1,c77164_g1,c93500_g1,c48655_g1,c37822_g1,c71603_g1,c52142_g2,c62466_g1,c38423_g1,c74712_g1,c72872_g1,c67246_g1,c111518_g1,c46544_g1,c47889_g1,c54443_g1,c69630_g1,c59896_g1,c97398_g1,c76192_g1,c41524_g1,c78499_g1,c5988_g1,c46119_g2,c58199_g1,c1398_g1,c83915_g1,c89815_g1,c59451_g1,c76864_g1,c41057_g2,c90369_g1,c107026_g1,c42807_g1,c63958_g1,c99594_g1,c56269_g1,c26284_g1,c84830_g1,c29075_g1,c88093_g1,c73561_g1,c83975_g1,c58304_g1,c78175_g1,c95205_g1,c31549_g1,c66235_g1,c16785_g1,c79094_g1,c71874_g1,c21811_g1,c102417_g1,c31759_g1,c104506_g1,c103549_g1,c87715_g1 |
| Retinol metabolism | ko00830 | 50 | c48241_g1,c35900_g1,c95562_g1,c73550_g1,c68793_g1,c84190_g1,c42135_g1,c52287_g1,c51298_g1,c84047_g1,c84139_g1,c9747_g1,c93937_g1,c63904_g1,c72851_g1,c32774_g1,c75473_g1,c22216_g1,c41564_g1,c108090_g1,c16528_g1,c21934_g1,c29603_g1,c49343_g1,c39602_g1,c36418_g1,c93096_g1,c92052_g1,c40100_g1,c61878_g1,c83751_g1,c105523_g1,c52376_g2,c107473_g1,c33782_g2,c39327_g1,c45977_g1,c47780_g1,c61548_g1,c37410_g1,c19829_g2,c98867_g1,c9747_g2,c99066_g1,c98527_g1,c46819_g1,c72293_g1,c28343_g1,c78663_g1,c81892_g1 |
| Riboflavin metabolism | ko00740 | 21 | c83807_g1,c50492_g1,c16646_g1,c36406_g1,c50715_g1,c80468_g1,c88220_g1,c40300_g1,c16646_g2,c35819_g1,c47514_g1,c85371_g1,c27428_g1,c99596_g1,c56495_g1,c43685_g1,c31737_g1,c47149_g1,c26117_g1,c42623_g1,c62369_g1 |
| Selenocompound metabolism | ko00450 | 50 | c72663_g1,c96286_g1,c92695_g1,c73942_g1,c42172_g1,c93494_g1,c84964_g1,c61269_g1,c101923_g1,c47213_g1,c100063_g1,c84258_g1,c39507_g1,c65644_g1,c3949_g1,c87086_g1,c112630_g1,c103665_g1,c97752_g1,c12056_g1,c98474_g1,c50526_g2,c105051_g1,c73597_g1,c108018_g1,c40950_g1,c66141_g1,c73269_g1,c109184_g1,c67800_g1,c56114_g1,c88808_g1,c26945_g1,c61290_g1,c87256_g1,c50526_g3,c108878_g1,c86247_g1,c43737_g1,c79395_g1,c101370_g1,c87656_g1,c63202_g1,c26211_g1,c31753_g1,c49301_g1,c94729_g1,c52226_g2,c63465_g1,c47928_g1 |
| Sesquiterpenoid and triterpenoid biosynthesis | ko00909 | 28 | c63619_g1,c101797_g1,c41520_g1,c48597_g1,c21445_g1,c49759_g1,c43339_g1,c48909_g1,c51514_g1,c47582_g1,c101626_g1,c52024_g1,c45996_g1,c57060_g1,c45674_g1,c1522_g1,c2514_g1,c87635_g1,c48909_g2,c61921_g1,c19786_g1,c50138_g1,c5916_g1,c2172_g1,c28506_g1,c46257_g1,c95625_g1,c41540_g1 |
| Sphingolipid metabolism | ko00600 | 65 | c48345_g1,c9216_g1,c25499_g1,c29259_g1,c99028_g1,c49964_g2,c46421_g1,c86706_g1,c111661_g1,c63753_g1,c51051_g1,c49377_g1,c45908_g1,c46476_g2,c42409_g1,c76122_g1,c54133_g1,c28450_g1,c44683_g1,c79990_g1,c64484_g1,c49876_g1,c71865_g1,c90992_g1,c56842_g1,c4650_g1,c94822_g1,c61725_g1,c75981_g1,c47968_g3,c111150_g1,c48458_g2,c83236_g1,c94182_g1,c42403_g2,c47903_g1,c51328_g1,c84934_g1,c25368_g1,c68640_g1,c35824_g1,c34189_g1,c47317_g1,c51641_g1,c6624_g1,c73787_g1,c105497_g1,c102204_g1,c71834_g1,c9542_g1,c49784_g1,c73667_g1,c30318_g1,c108220_g1,c96091_g1,c77768_g1,c38431_g1,c82061_g1,c52128_g1,c83770_g1,c34090_g1,c41577_g1,c71981_g1,c89199_g1,c547_g1 |
| Starch and sucrose metabolism | ko00500 | 318 | c4013_g1,c75871_g1,c39220_g1,c10715_g1,c31604_g1,c79060_g1,c75022_g1,c49729_g1,c104176_g1,c26992_g1,c34485_g1,c43433_g1,c39752_g1,c8202_g2,c84181_g1,c42588_g1,c51294_g2,c52437_g1,c26188_g1,c105092_g1,c40346_g1,c83271_g1,c40778_g1,c104216_g1,c50382_g1,c87820_g1,c41978_g1,c6348_g1,c48471_g1,c4006_g1,c85940_g1,c51037_g1,c43881_g1,c30877_g1,c33732_g1,c75364_g1,c34945_g1,c101041_g1,c45733_g1,c44033_g2,c67994_g1,c44806_g1,c82509_g1,c56675_g1,c47534_g1,c45667_g1,c17039_g1,c69139_g1,c103806_g1,c67708_g1,c50835_g7,c24859_g1,c28618_g1,c63646_g1,c72765_g1,c103438_g1,c72568_g1,c105620_g1,c44699_g2,c94683_g1,c15358_g1,c75151_g1,c45524_g2,c50532_g1,c52258_g2,c50574_g1,c24020_g1,c83090_g1,c45394_g1,c71270_g1,c94893_g1,c99880_g1,c47139_g1,c66185_g1,c38793_g1,c99912_g1,c106949_g1,c78558_g1,c56460_g1,c49578_g1,c37640_g1,c28808_g1,c25280_g1,c88495_g1,c38097_g1,c24869_g1,c112628_g1,c55215_g1,c46876_g1,c112708_g1,c47223_g3,c51877_g1,c84333_g1,c64130_g1,c9748_g1,c46090_g1,c93993_g1,c29352_g1,c73306_g1,c4084_g1,c41774_g1,c32138_g1,c14057_g1,c99377_g1,c50835_g8,c52728_g1,c16621_g1,c25044_g1,c74356_g1,c72186_g1,c87047_g1,c38151_g1,c25044_g2,c35474_g1,c32367_g1,c77371_g1,c24454_g1,c48933_g1,c73347_g1,c49823_g1,c39522_g1,c67217_g1,c81638_g1,c26403_g1,c55255_g1,c28388_g2,c76615_g1,c49997_g1,c44578_g1,c67461_g1,c83930_g1,c39033_g1,c43413_g1,c46831_g1,c44624_g1,c71759_g1,c74204_g1,c15366_g1,c40818_g1,c107170_g1,c49654_g1,c47514_g1,c33766_g1,c85371_g1,c24630_g1,c39602_g1,c7310_g1,c100584_g1,c40728_g1,c14572_g1,c27149_g1,c48923_g2,c109158_g1,c52483_g1,c46365_g1,c66680_g1,c44736_g1,c45581_g1,c65008_g1,c92777_g1,c50882_g2,c40522_g1,c111846_g1,c40831_g1,c49654_g2,c28265_g1,c103333_g1,c42339_g1,c44888_g1,c50381_g1,c49231_g1,c93503_g1,c47370_g1,c55616_g1,c52507_g1,c40831_g2,c54597_g1,c26184_g1,c99744_g1,c42366_g1,c53150_g1,c57149_g1,c41900_g1,c105150_g1,c50261_g1,c71906_g1,c26444_g1,c56290_g1,c32001_g1,c72705_g1,c57623_g1,c100057_g1,c49007_g1,c42520_g1,c41639_g1,c105463_g1,c96453_g1,c48976_g1,c43212_g1,c74035_g1,c56604_g1,c103744_g1,c32052_g1,c31452_g1,c12768_g2,c49171_g1,c46907_g1,c47216_g1,c62502_g1,c6396_g1,c50236_g1,c26299_g1,c18342_g1,c104504_g1,c54955_g1,c106634_g1,c67624_g1,c61219_g1,c60986_g1,c7086_g1,c71804_g1,c50637_g1,c8202_g1,c9971_g1,c14268_g1,c41683_g1,c48532_g1,c53702_g1,c74312_g1,c4000_g1,c12481_g1,c51615_g1,c44013_g1,c37410_g1,c104211_g1,c46907_g2,c64949_g1,c51058_g1,c58915_g1,c48071_g1,c39993_g1,c99259_g1,c46473_g1,c49525_g2,c85948_g1,c57956_g1,c47634_g1,c45030_g1,c13374_g1,c81861_g1,c99066_g1,c2063_g1,c35846_g1,c26165_g1,c110906_g1,c48149_g1,c74875_g1,c51775_g1,c30673_g1,c30774_g1,c58536_g1,c49391_g1,c47590_g1,c72127_g1,c46907_g3,c29715_g1,c46480_g1,c73869_g1,c62881_g1,c43261_g1,c26258_g1,c60708_g1,c51093_g1,c47223_g1,c65633_g1,c50781_g1,c39511_g2,c46875_g1,c48234_g1,c48740_g1,c61443_g1,c6455_g1,c76110_g1,c70281_g1,c86498_g1,c54779_g1,c51251_g2,c24632_g1,c10950_g1,c86421_g1,c107457_g1,c4381_g1,c47223_g2,c7528_g1,c52381_g3,c87605_g1,c66108_g1,c66969_g1,c45860_g1,c25367_g3,c47095_g1,c24540_g1,c33348_g1,c52381_g2,c43058_g1,c60648_g1,c15010_g1,c44126_g1,c52365_g1,c49957_g1,c11015_g1,c47089_g1,c783_g1,c63024_g1,c31739_g1,c50003_g1,c109854_g1,c78933_g1 |
| Steroid biosynthesis | ko00100 | 52 | c73099_g1,c41333_g1,c12870_g1,c40416_g1,c48597_g1,c40165_g1,c49759_g1,c24949_g1,c43951_g1,c86365_g1,c56904_g1,c74206_g1,c4186_g1,c15131_g1,c37270_g1,c43147_g1,c94008_g1,c75438_g1,c93650_g1,c29212_g1,c51919_g2,c46987_g1,c45996_g1,c51452_g3,c71780_g1,c69117_g1,c82081_g1,c54922_g1,c47051_g1,c12243_g1,c48958_g1,c68074_g1,c40719_g2,c44655_g1,c29788_g1,c49341_g1,c26220_g1,c49116_g1,c16186_g1,c27329_g1,c50138_g1,c17456_g1,c56419_g1,c44963_g1,c47922_g1,c78216_g1,c54699_g1,c30894_g1,c816_g1,c38742_g1,c51828_g1,c86104_g1 |
| Steroid hormone biosynthesis | ko00140 | 28 | c51154_g3,c84190_g1,c52287_g1,c43519_g1,c95324_g1,c63904_g1,c32774_g1,c50040_g1,c64939_g1,c21934_g1,c29603_g1,c49343_g1,c39602_g1,c37278_g2,c61878_g1,c52376_g2,c33960_g1,c64756_g1,c105322_g1,c47780_g1,c37410_g1,c98867_g1,c82743_g1,c99066_g1,c72293_g1,c28343_g1,c63304_g1,c41393_g1 |
| Stilbenoid | ko00945 | 65 | c62744_g1,c1193_g1,c49461_g1,c87450_g1,c44494_g1,c63072_g1,c52112_g1,c57881_g1,c26805_g1,c52112_g2,c24471_g1,c54530_g1,c95509_g1,c52112_g3,c110831_g1,c11088_g1,c52941_g1,c54345_g1,c83889_g1,c51428_g3,c83030_g1,c93747_g1,c25007_g2,c99792_g1,c84218_g1,c108064_g1,c36408_g1,c31168_g1,c33474_g1,c43675_g2,c103522_g1,c50878_g1,c83163_g1,c72777_g1,c82195_g1,c346_g1,c52985_g1,c11795_g1,c2765_g1,c46140_g1,c11407_g1,c10651_g1,c25106_g1,c14751_g1,c24369_g1,c71527_g1,c10846_g1,c35949_g1,c28083_g1,c110641_g1,c37774_g1,c74253_g1,c83161_g1,c46108_g1,c71682_g1,c27078_g1,c13468_g1,c100652_g1,c62392_g1,c17108_g1,c41788_g1,c51227_g1,c46529_g1,c82973_g1,c4094_g1 |
| Streptomycin biosynthesis | ko00521 | 20 | c111201_g1,c6348_g1,c67994_g1,c47534_g1,c103865_g1,c81638_g1,c74204_g1,c100584_g1,c50324_g1,c55616_g1,c45836_g1,c101725_g1,c72745_g1,c50236_g1,c30673_g1,c46480_g1,c107467_g1,c52381_g3,c52381_g2,c63024_g1 |
| Styrene degradation | ko00643 | 23 | c27881_g1,c43118_g1,c110051_g1,c71938_g1,c36433_g1,c43482_g1,c46568_g1,c29069_g1,c36724_g1,c74538_g1,c62433_g1,c18926_g1,c46599_g1,c54005_g1,c28775_g2,c84081_g1,c15696_g1,c48317_g1,c73168_g1,c88744_g1,c1444_g1,c28775_g1,c84734_g1 |
| Sulfur metabolism | ko00920 | 79 | c93666_g1,c200_g2,c108958_g1,c73078_g1,c58424_g1,c77822_g1,c90205_g1,c84943_g1,c105449_g1,c3557_g1,c94230_g1,c5430_g1,c66105_g1,c81472_g1,c101923_g1,c47213_g1,c93921_g1,c100063_g1,c53606_g1,c50667_g1,c3949_g1,c11155_g1,c98178_g1,c104246_g1,c62793_g1,c1530_g2,c6075_g1,c8176_g2,c47900_g1,c43294_g1,c47900_g2,c40967_g1,c105051_g1,c71045_g1,c31587_g1,c37875_g1,c26838_g1,c73353_g1,c45500_g1,c58532_g1,c100327_g1,c1530_g1,c109184_g1,c91111_g1,c3692_g1,c68127_g1,c82679_g1,c85544_g1,c45425_g1,c49854_g1,c20434_g1,c63456_g1,c44377_g1,c50787_g1,c52411_g3,c78700_g1,c110207_g1,c16834_g1,c43737_g1,c45327_g2,c103322_g1,c55559_g1,c45327_g3,c67906_g1,c85234_g1,c40926_g1,c62420_g1,c61407_g1,c11160_g1,c65544_g1,c99367_g1,c49887_g1,c49854_g2,c44573_g1,c31740_g1,c51974_g1,c55737_g1,c72141_g1,c96545_g1 |
| Synthesis and degradation of ketone bodies | ko00072 | 18 | c18220_g1,c48902_g1,c92986_g1,c77190_g1,c80976_g1,c75123_g1,c64933_g1,c103615_g1,c94166_g1,c82596_g1,c107241_g1,c52901_g1,c37822_g1,c72872_g1,c54704_g1,c38496_g1,c31759_g1,c39018_g1 |
| Taurine and hypotaurine metabolism | ko00430 | 14 | c45383_g1,c954_g1,c2590_g1,c83027_g1,c50971_g1,c30097_g1,c45690_g1,c65617_g1,c94313_g1,c13566_g1,c34353_g1,c44310_g1,c52918_g1,c59463_g1 |
| Terpenoid backbone biosynthesis | ko00900 | 90 | c85141_g1,c49478_g1,c34423_g1,c18220_g1,c63619_g1,c44752_g2,c48902_g1,c57233_g1,c99965_g1,c40205_g1,c65861_g1,c45150_g1,c38736_g2,c51322_g1,c13121_g1,c51793_g1,c52352_g2,c83322_g1,c98049_g1,c45559_g1,c26433_g1,c103507_g1,c50442_g1,c10653_g1,c48406_g1,c47582_g1,c77190_g1,c49826_g2,c45756_g1,c103565_g1,c80976_g1,c30757_g1,c38160_g1,c107306_g1,c28452_g1,c75123_g1,c46382_g1,c51069_g1,c50142_g1,c9761_g1,c74208_g1,c64933_g1,c33344_g1,c27846_g2,c77518_g1,c50995_g1,c71711_g1,c59768_g1,c40969_g1,c9546_g1,c46398_g2,c71573_g1,c14988_g1,c50442_g2,c103615_g1,c68167_g1,c51069_g2,c43723_g1,c42496_g1,c58204_g1,c50790_g1,c50362_g1,c2048_g1,c93381_g1,c100226_g1,c94166_g1,c29539_g1,c66831_g1,c46623_g1,c50373_g1,c48192_g1,c46475_g1,c37819_g1,c52901_g1,c58428_g1,c37822_g1,c84740_g1,c93872_g1,c72872_g1,c13675_g1,c41525_g1,c93281_g1,c35801_g1,c73371_g1,c51442_g2,c10650_g2,c38496_g1,c64034_g1,c31759_g1,c12917_g1 |
| Tetracycline biosynthesis | ko00253 | 18 | c64170_g1,c31007_g1,c47937_g2,c85100_g1,c837_g1,c85941_g1,c67839_g1,c82477_g1,c50138_g2,c46119_g1,c99135_g1,c62374_g1,c58779_g1,c111518_g1,c46544_g1,c54443_g1,c59896_g1,c46119_g2 |
| Thiamine metabolism | ko00730 | 30 | c36521_g1,c49478_g1,c50010_g1,c97135_g1,c10653_g1,c112796_g1,c40304_g1,c34967_g1,c67976_g1,c9761_g1,c68836_g1,c40969_g1,c110026_g1,c43723_g1,c50790_g1,c93381_g1,c61290_g1,c109030_g1,c2233_g1,c87256_g1,c47554_g1,c86247_g1,c4070_g1,c48482_g1,c49301_g1,c73983_g1,c93778_g1,c94705_g1,c60585_g1,c64190_g1 |
| Toluene degradation | ko00623 | 4 | c63842_g1,c21113_g2,c49975_g1,c57110_g1 |
| Tropane | ko00960 | 58 | c110883_g1,c94421_g1,c61635_g1,c47400_g1,c42740_g1,c73106_g1,c106588_g1,c86188_g1,c45334_g1,c19206_g1,c36407_g1,c80695_g1,c47185_g1,c70219_g1,c40621_g1,c80270_g1,c50870_g1,c1350_g1,c94629_g1,c93455_g1,c106159_g1,c36513_g1,c39997_g1,c56783_g1,c6116_g1,c51907_g4,c57611_g1,c12938_g1,c35009_g1,c12938_g2,c28212_g1,c91964_g1,c50389_g1,c32605_g1,c34313_g1,c48242_g1,c40850_g1,c84626_g1,c72626_g1,c104622_g1,c78598_g1,c73923_g1,c34116_g1,c103084_g1,c43501_g1,c41537_g1,c1753_g1,c38946_g1,c34007_g1,c48882_g1,c47618_g1,c41423_g1,c110574_g1,c107837_g1,c63265_g1,c32423_g1,c51868_g1,c48624_g1 |
| Tryptophan metabolism | ko00380 | 115 | c94211_g1,c78634_g1,c89219_g1,c25134_g1,c71672_g1,c65199_g1,c74805_g1,c54766_g1,c47621_g1,c48902_g1,c48536_g2,c35216_g1,c93426_g1,c110089_g1,c64803_g1,c47365_g1,c86580_g1,c44030_g1,c95742_g1,c11271_g1,c106358_g1,c93591_g1,c84190_g1,c52287_g1,c106670_g1,c40766_g1,c98829_g1,c44312_g1,c97539_g1,c75402_g1,c62190_g1,c47244_g1,c73811_g1,c63904_g1,c39609_g1,c77190_g1,c48536_g3,c112656_g1,c32774_g1,c16415_g1,c34608_g1,c80976_g1,c43134_g1,c9475_g1,c88691_g1,c54418_g1,c41057_g1,c55541_g1,c75123_g1,c44303_g1,c50235_g1,c75055_g1,c61823_g1,c105469_g1,c21934_g1,c29603_g1,c65913_g1,c73597_g1,c51291_g2,c49343_g1,c64933_g1,c29553_g1,c46568_g1,c78345_g1,c61878_g1,c53992_g1,c62384_g1,c49575_g1,c52376_g2,c26866_g1,c49723_g1,c51291_g5,c103615_g1,c52336_g2,c58316_g1,c45529_g1,c45085_g1,c88902_g1,c53305_g1,c42054_g1,c14937_g1,c102364_g1,c46599_g1,c100039_g1,c47780_g1,c73343_g1,c54005_g1,c63784_g1,c98867_g1,c28775_g2,c55064_g1,c87656_g1,c54520_g1,c62978_g1,c68022_g1,c26211_g1,c37822_g1,c48317_g1,c93760_g1,c3978_g1,c72872_g1,c51291_g4,c76192_g1,c17106_g1,c72293_g1,c28343_g1,c1398_g1,c83915_g1,c89815_g1,c41057_g2,c90369_g1,c94729_g1,c28775_g1,c57655_g1,c31759_g1 |
| Tyrosine metabolism | ko00350 | 117 | c48241_g1,c27881_g1,c110883_g1,c61635_g1,c47400_g1,c42740_g1,c73106_g1,c106588_g1,c82363_g1,c46702_g1,c86188_g1,c43118_g1,c29251_g1,c35259_g1,c19206_g1,c36407_g1,c110051_g1,c80695_g1,c44873_g1,c47185_g1,c77300_g1,c40621_g1,c95199_g1,c68793_g1,c42135_g1,c13239_g1,c19209_g1,c36419_g1,c84047_g1,c71938_g1,c73821_g1,c45291_g1,c80270_g1,c9747_g1,c50870_g1,c36433_g1,c93937_g1,c100784_g1,c1350_g1,c44795_g1,c106159_g1,c47713_g1,c37416_g1,c52843_g3,c36513_g1,c39997_g1,c56783_g1,c75473_g1,c57611_g1,c22216_g1,c12938_g1,c43482_g1,c31736_g1,c12938_g2,c68148_g1,c28212_g1,c108090_g1,c16528_g1,c91964_g1,c4521_g1,c53527_g1,c50389_g1,c109475_g1,c36418_g1,c92052_g1,c40100_g1,c29069_g1,c109806_g1,c36724_g1,c40850_g1,c107473_g1,c24713_g1,c74538_g1,c84626_g1,c33782_g2,c72626_g1,c104622_g1,c78598_g1,c83145_g1,c62433_g1,c73923_g1,c15049_g1,c54662_g1,c34116_g1,c18926_g1,c103084_g1,c43501_g1,c45977_g1,c51578_g1,c1753_g1,c88333_g1,c19829_g2,c82743_g1,c38946_g1,c9747_g2,c9351_g1,c98527_g1,c35384_g1,c84081_g1,c48882_g1,c15696_g1,c48658_g1,c47618_g1,c46819_g1,c109944_g1,c73168_g1,c88744_g1,c81892_g1,c48686_g1,c41423_g1,c110574_g1,c1444_g1,c63265_g1,c84734_g1,c32423_g1,c51868_g1,c48624_g1 |
| Ubiquinone and other terpenoid-quinone biosynthesis | ko00130 | 77 | c90054_g1,c51862_g2,c95583_g1,c51684_g1,c39949_g1,c73876_g1,c47400_g1,c73106_g1,c92511_g1,c52381_g4,c86188_g1,c67763_g1,c54742_g1,c44553_g1,c36407_g1,c104156_g1,c52903_g1,c109910_g1,c84553_g1,c61297_g1,c82179_g1,c35123_g1,c110757_g1,c16684_g1,c91920_g1,c43182_g1,c98726_g1,c106159_g1,c52550_g2,c57250_g1,c50048_g1,c49643_g1,c8170_g1,c108213_g1,c105678_g1,c25145_g1,c8444_g1,c52423_g1,c49078_g2,c57417_g1,c71872_g1,c41159_g1,c53527_g1,c98274_g1,c77399_g1,c34358_g1,c52550_g3,c104217_g1,c2100_g1,c51217_g2,c78598_g1,c41260_g1,c52550_g1,c72572_g1,c36592_g1,c94900_g1,c110536_g1,c34116_g1,c52550_g4,c27191_g1,c84168_g1,c51088_g1,c47683_g1,c38946_g1,c49078_g1,c91339_g1,c46296_g2,c41451_g1,c38056_g1,c108375_g1,c41423_g1,c83673_g1,c50983_g1,c43127_g1,c29512_g1,c50461_g1,c44990_g1 |
| Valine | ko00280 | 227 | c64043_g1,c52678_g2,c8198_g1,c53491_g1,c18220_g1,c89219_g1,c94070_g1,c39740_g1,c11497_g1,c44391_g1,c47621_g1,c83165_g1,c95498_g1,c9421_g1,c48902_g1,c13346_g1,c48536_g2,c65924_g1,c35216_g1,c79442_g1,c52533_g4,c65899_g1,c52798_g4,c62877_g1,c27642_g1,c110089_g1,c99472_g1,c77947_g1,c726_g1,c47365_g1,c37769_g1,c52818_g1,c86580_g1,c47420_g1,c18801_g1,c99073_g1,c52317_g6,c106211_g1,c69486_g1,c92658_g1,c103508_g1,c106670_g1,c43009_g2,c11144_g1,c81982_g1,c85139_g1,c61770_g1,c52729_g1,c55995_g1,c40766_g1,c50954_g5,c108204_g1,c99712_g1,c49366_g9,c66158_g1,c47384_g1,c30465_g1,c89320_g1,c92986_g1,c72878_g1,c97539_g1,c75402_g1,c49098_g3,c62190_g1,c47244_g1,c73811_g1,c54475_g1,c47003_g1,c77190_g1,c48536_g3,c104923_g1,c34608_g1,c80976_g1,c52061_g2,c65042_g1,c91515_g1,c39032_g1,c54787_g1,c88691_g1,c33831_g1,c90229_g1,c1906_g1,c48257_g1,c78330_g1,c49677_g2,c12050_g1,c109689_g1,c41057_g1,c55541_g1,c27943_g1,c27323_g1,c75123_g1,c17036_g1,c44818_g1,c12162_g1,c47268_g1,c97481_g1,c27619_g1,c50235_g1,c83324_g1,c50796_g9,c36907_g1,c50954_g2,c18052_g1,c75055_g1,c61823_g1,c53832_g1,c95201_g1,c19334_g1,c105469_g1,c53626_g1,c50584_g1,c96322_g1,c58116_g1,c48999_g1,c103114_g1,c46948_g1,c43745_g1,c64933_g1,c29553_g1,c43444_g2,c49366_g5,c98660_g1,c85135_g1,c42337_g1,c106416_g1,c46156_g1,c93748_g1,c49677_g1,c6594_g1,c49723_g1,c59037_g1,c34317_g1,c91334_g1,c103615_g1,c28308_g1,c52336_g2,c104063_g1,c58316_g1,c9457_g1,c46725_g1,c30815_g1,c109895_g1,c45085_g1,c77787_g1,c54776_g1,c52434_g1,c6536_g1,c102459_g1,c52030_g1,c98371_g1,c84199_g1,c460_g1,c94166_g1,c30117_g1,c54384_g1,c85240_g1,c102364_g1,c27356_g1,c52858_g6,c102188_g1,c25261_g1,c44229_g1,c44681_g1,c83507_g1,c82596_g1,c29591_g1,c36340_g1,c34019_g1,c53374_g1,c64429_g1,c67667_g1,c18177_g1,c6656_g1,c81538_g1,c39207_g1,c46277_g1,c48735_g1,c107241_g1,c66210_g1,c53105_g1,c105905_g1,c111270_g1,c95977_g1,c62978_g1,c47598_g1,c42128_g1,c94269_g1,c93690_g1,c103892_g1,c52901_g1,c48105_g1,c112132_g1,c52271_g2,c37822_g1,c29000_g1,c82737_g1,c78760_g1,c72872_g1,c78203_g1,c83437_g1,c76192_g1,c43660_g1,c1398_g1,c52466_g1,c49366_g1,c83915_g1,c89815_g1,c93857_g1,c10844_g2,c41057_g2,c90369_g1,c110160_g1,c48230_g1,c46123_g2,c73561_g1,c54704_g1,c9318_g1,c46775_g1,c51499_g2,c62283_g1,c46331_g1,c38496_g1,c110093_g1,c31759_g1,c404_g1,c39018_g1 |
| Valine | ko00290 | 62 | c6029_g1,c11263_g1,c95034_g1,c80465_g1,c16964_g1,c71149_g1,c79442_g1,c95068_g1,c94505_g1,c105038_g1,c32971_g1,c55416_g1,c66158_g1,c66611_g1,c67039_g1,c49098_g3,c67769_g1,c47003_g1,c49757_g3,c41831_g1,c61242_g1,c66195_g1,c65042_g1,c64274_g1,c104555_g1,c93338_g1,c66239_g1,c78544_g1,c74727_g1,c34013_g1,c48894_g1,c32058_g1,c51740_g1,c43327_g1,c87783_g1,c9286_g1,c67249_g1,c98277_g1,c104877_g1,c75192_g1,c12516_g1,c61619_g1,c42387_g1,c96844_g1,c76059_g1,c3742_g1,c95977_g1,c94269_g1,c70771_g1,c29000_g1,c57820_g1,c72642_g1,c86327_g1,c48449_g1,c56269_g1,c64536_g1,c9318_g1,c46331_g1,c74521_g1,c87107_g1,c47551_g1,c46851_g1 |
| Various types of N-glycan biosynthesis | ko00513 | 57 | c65217_g1,c42055_g1,c47359_g1,c55605_g1,c87339_g1,c40679_g1,c29861_g1,c34532_g1,c45075_g1,c107452_g1,c38107_g1,c64583_g1,c53758_g1,c46139_g1,c49863_g1,c88543_g1,c106225_g1,c94704_g1,c83103_g1,c93276_g1,c46454_g1,c47138_g1,c54919_g1,c11693_g1,c64187_g1,c67129_g1,c48108_g1,c44156_g1,c47638_g1,c7078_g1,c46757_g1,c38194_g1,c86564_g1,c48418_g1,c80944_g1,c45570_g1,c82328_g1,c62491_g1,c51673_g1,c89814_g1,c37708_g2,c101193_g1,c66322_g1,c46588_g1,c69249_g1,c94457_g1,c8895_g1,c55979_g1,c62421_g1,c91146_g1,c104206_g1,c54350_g1,c67459_g1,c7901_g1,c42338_g1,c51606_g1,c44940_g1 |
| Vitamin B6 metabolism | ko00750 | 30 | c107871_g1,c46719_g1,c59201_g1,c107660_g1,c50179_g2,c85653_g1,c42027_g1,c40375_g1,c97094_g1,c23181_g1,c18439_g1,c45113_g1,c106540_g1,c49953_g1,c87161_g1,c93038_g1,c44349_g1,c27301_g1,c78529_g1,c57349_g1,c63367_g1,c43398_g1,c19212_g1,c92958_g1,c29654_g1,c34167_g1,c8689_g1,c4522_g1,c54230_g1,c39527_g1 |
| Zeatin biosynthesis | ko00908 | 27 | c83202_g1,c38786_g1,c57237_g1,c48229_g1,c32987_g1,c15788_g1,c61481_g1,c36729_g1,c9564_g1,c73548_g1,c48961_g1,c46601_g1,c36657_g1,c85697_g1,c52260_g4,c49107_g2,c51211_g1,c61154_g1,c96554_g1,c9924_g1,c68044_g1,c110510_g1,c19928_g1,c49107_g1,c95445_g1,c79937_g1,c19275_g1 |
| >2. Genetic Information Processing |  |  |  |
| Aminoacyl-tRNA biosynthesis | ko00970 | 164 | c72663_g1,c43598_g1,c61401_g1,c100577_g1,c49930_g1,c61355_g1,c11106_g1,c54055_g1,c39538_g1,c99346_g1,c84590_g1,c52273_g2,c50549_g2,c51084_g1,c97108_g1,c36846_g1,c54236_g1,c100225_g1,c94919_g1,c78197_g1,c50072_g1,c50167_g2,c73443_g1,c30005_g1,c13301_g1,c98702_g1,c86430_g1,c32301_g1,c24917_g1,c50082_g1,c48586_g2,c49855_g1,c69506_g1,c75459_g1,c97629_g1,c30529_g1,c61873_g1,c41475_g1,c78038_g1,c113157_g1,c61269_g1,c16830_g1,c106313_g1,c6027_g1,c79317_g1,c80736_g1,c98500_g1,c65644_g1,c60803_g1,c111510_g1,c96374_g1,c51937_g1,c41910_g1,c86512_g1,c74717_g1,c71990_g1,c77057_g1,c72860_g1,c17098_g1,c96338_g1,c100775_g1,c49991_g1,c13445_g1,c92934_g1,c84347_g1,c87139_g1,c54589_g1,c19264_g1,c67067_g1,c50931_g1,c42557_g1,c47620_g1,c96742_g1,c50979_g1,c12491_g2,c76536_g1,c108526_g1,c70866_g1,c61722_g1,c62371_g1,c77919_g1,c61983_g1,c81552_g1,c1413_g1,c84023_g1,c47708_g1,c40950_g1,c104367_g1,c90060_g1,c106449_g1,c95180_g1,c84571_g1,c48545_g3,c107191_g1,c76965_g1,c109873_g1,c48472_g1,c67800_g1,c99486_g1,c97538_g1,c97631_g1,c111095_g1,c107721_g1,c50033_g1,c61301_g1,c26945_g1,c38136_g1,c108545_g1,c96056_g1,c105708_g1,c74153_g1,c74214_g1,c82568_g1,c43716_g1,c86083_g1,c47443_g2,c15616_g1,c29113_g1,c94671_g1,c49428_g1,c61641_g1,c112831_g1,c73599_g1,c47708_g2,c98851_g1,c79395_g1,c48411_g2,c109841_g1,c105410_g1,c76097_g1,c50247_g1,c351_g1,c73592_g1,c14770_g2,c7768_g1,c63202_g1,c107230_g1,c86199_g1,c109570_g1,c16896_g2,c85719_g1,c45159_g1,c12190_g1,c101228_g1,c708_g1,c43975_g1,c105827_g1,c82395_g1,c99482_g1,c38411_g1,c56090_g1,c74022_g1,c103922_g1,c107900_g1,c40698_g1,c82278_g1,c84765_g1,c74303_g1,c93043_g1,c48545_g2,c53090_g1,c36578_g1,c61806_g1,c47928_g1 |
| Basal transcription factors | ko03022 | 47 | c47667_g1,c56832_g1,c32329_g1,c31541_g1,c45652_g1,c47984_g1,c51644_g1,c50788_g1,c27289_g1,c86211_g1,c100104_g1,c94103_g1,c51299_g1,c41576_g1,c50281_g2,c101381_g1,c48215_g1,c2913_g1,c39127_g1,c51643_g2,c47980_g1,c76231_g1,c45501_g1,c38755_g1,c105296_g1,c18356_g1,c38931_g1,c71059_g1,c50704_g2,c93569_g1,c49884_g1,c41237_g1,c29085_g1,c41098_g1,c49211_g2,c34592_g1,c47120_g1,c25526_g1,c29016_g1,c34587_g1,c62625_g1,c40636_g1,c11161_g1,c14374_g1,c49696_g1,c40022_g1,c50149_g2 |
| Base excision repair | ko03410 | 69 | c43726_g1,c58136_g1,c85253_g1,c40118_g1,c104301_g1,c42874_g1,c52039_g1,c19372_g1,c97771_g1,c51337_g1,c63859_g1,c35641_g1,c42827_g1,c101974_g1,c39282_g1,c82246_g1,c63101_g1,c44576_g1,c40177_g1,c44699_g1,c51273_g6,c109005_g1,c46228_g2,c92670_g1,c73461_g1,c68514_g1,c24719_g1,c52486_g1,c63986_g1,c97441_g1,c72533_g1,c46860_g1,c41365_g1,c48988_g2,c47399_g1,c94535_g1,c48908_g1,c4024_g1,c103673_g1,c36262_g1,c34493_g1,c61946_g1,c61973_g1,c38068_g1,c56860_g1,c51238_g1,c57770_g1,c104417_g1,c68887_g1,c100618_g1,c62404_g1,c40852_g1,c47817_g2,c63377_g1,c73833_g1,c51108_g1,c49341_g3,c65713_g1,c47817_g1,c104180_g1,c92988_g1,c13104_g1,c52009_g1,c49089_g1,c73623_g1,c52531_g1,c43495_g1,c41567_g1,c49427_g1 |
| DNA replication | ko03030 | 79 | c95746_g1,c48844_g1,c93317_g1,c53216_g1,c71843_g1,c36575_g1,c11707_g1,c49041_g1,c52749_g2,c87775_g1,c104301_g1,c45250_g1,c99863_g1,c52039_g1,c97771_g1,c88057_g1,c51337_g1,c84028_g1,c63859_g1,c35641_g1,c42827_g1,c101974_g1,c82246_g1,c63101_g1,c107035_g1,c106343_g1,c49889_g1,c44307_g1,c99166_g1,c35284_g1,c45397_g1,c39597_g1,c92670_g1,c73461_g1,c41456_g1,c71958_g1,c52486_g1,c63986_g1,c72533_g1,c25712_g1,c42895_g1,c48988_g2,c98062_g1,c5612_g1,c96589_g1,c63285_g1,c36262_g1,c34493_g1,c17238_g1,c45922_g1,c82910_g1,c93229_g1,c43600_g1,c56860_g1,c52200_g1,c46575_g1,c57770_g1,c100618_g1,c42506_g1,c4916_g1,c52173_g1,c40852_g1,c28631_g1,c108410_g1,c51108_g1,c49341_g3,c112465_g1,c65713_g1,c104180_g1,c51369_g1,c92988_g1,c13104_g1,c41885_g1,c73623_g1,c52531_g1,c44599_g1,c33371_g1,c98855_g1,c49427_g1 |
| Fanconi anemia pathway | ko03460 | 68 | c48844_g1,c403_g1,c52106_g1,c61233_g1,c52749_g2,c13668_g1,c47647_g1,c40216_g1,c31238_g1,c52667_g2,c50517_g1,c21634_g1,c95505_g1,c110983_g1,c51415_g1,c36179_g1,c108982_g1,c103282_g1,c48104_g1,c40040_g1,c49405_g1,c52615_g1,c44352_g1,c44307_g1,c52258_g1,c99166_g1,c52582_g1,c52153_g1,c13038_g1,c45397_g1,c95177_g1,c50273_g2,c52597_g1,c71958_g1,c52807_g1,c36481_g1,c46307_g1,c51411_g1,c51899_g1,c45156_g1,c50050_g1,c108889_g1,c79863_g1,c50399_g1,c62666_g1,c5612_g1,c96589_g1,c52571_g1,c52797_g1,c17238_g1,c93229_g1,c46575_g1,c49310_g1,c57672_g1,c10264_g1,c52719_g7,c52549_g1,c50594_g1,c47632_g2,c4916_g1,c28396_g1,c108410_g1,c19158_g1,c109692_g1,c48381_g1,c52062_g1,c24373_g1,c33371_g1 |
| Homologous recombination | ko03440 | 61 | c48844_g1,c52749_g2,c45250_g1,c31238_g1,c51337_g1,c50517_g1,c108982_g1,c103282_g1,c48104_g1,c83281_g1,c52694_g1,c51068_g1,c49330_g1,c44307_g1,c46442_g1,c99166_g1,c45397_g1,c96595_g1,c18430_g1,c72254_g1,c52597_g1,c112181_g1,c44047_g1,c71958_g1,c2150_g1,c52486_g1,c63986_g1,c79973_g1,c24377_g1,c106426_g1,c48988_g2,c45388_g1,c50399_g1,c75432_g1,c5612_g1,c96589_g1,c36262_g1,c49348_g1,c34493_g1,c17238_g1,c50926_g1,c93229_g1,c56860_g1,c48932_g1,c46575_g1,c57770_g1,c52549_g1,c50594_g1,c4916_g1,c28396_g1,c48860_g1,c108410_g1,c19158_g1,c48236_g1,c49341_g3,c65713_g1,c13104_g1,c73623_g1,c107546_g1,c33371_g1,c49427_g1 |
| Mismatch repair | ko03430 | 65 | c48844_g1,c10141_g1,c52711_g1,c42013_g1,c38318_g1,c52749_g2,c104301_g1,c45250_g1,c97771_g1,c74282_g1,c63859_g1,c101974_g1,c82246_g1,c63101_g1,c52104_g1,c107035_g1,c49889_g1,c44307_g1,c51923_g1,c99166_g1,c51473_g2,c35284_g1,c49984_g1,c51468_g1,c45397_g1,c44645_g1,c50273_g2,c73461_g1,c36583_g1,c71958_g1,c52486_g1,c72533_g1,c45156_g1,c108889_g1,c48988_g2,c5612_g1,c96589_g1,c36262_g1,c34493_g1,c17238_g1,c93229_g1,c56860_g1,c52200_g1,c46575_g1,c57770_g1,c100618_g1,c42506_g1,c4916_g1,c94736_g1,c106919_g1,c28631_g1,c108410_g1,c61341_g1,c51108_g1,c49341_g3,c65713_g1,c104180_g1,c92988_g1,c13104_g1,c41885_g1,c73623_g1,c52531_g1,c33371_g1,c98855_g1,c49427_g1 |
| mRNA surveillance pathway | ko03015 | 234 | c47635_g2,c28835_g1,c68732_g1,c51781_g3,c48996_g1,c26910_g1,c52510_g1,c6097_g1,c31021_g1,c42550_g1,c21184_g1,c90691_g1,c105973_g1,c34494_g1,c46305_g1,c49637_g1,c30900_g1,c109525_g1,c62815_g1,c82872_g1,c46651_g1,c92940_g1,c82150_g1,c9202_g1,c53983_g1,c51984_g1,c40511_g1,c14051_g1,c58723_g1,c48729_g1,c27434_g1,c29687_g1,c81287_g1,c74862_g1,c92153_g1,c101090_g1,c14649_g1,c47222_g1,c21700_g1,c52284_g1,c26033_g1,c79325_g1,c18431_g1,c54500_g1,c45415_g1,c37435_g1,c62816_g1,c71535_g1,c3955_g1,c104747_g1,c47222_g2,c96913_g1,c21694_g1,c17127_g1,c92930_g1,c48566_g1,c96128_g1,c46462_g1,c56690_g1,c26107_g1,c48127_g3,c92800_g1,c108910_g1,c26657_g1,c50398_g3,c9107_g1,c103268_g1,c59234_g1,c101438_g1,c35496_g1,c100082_g1,c46109_g3,c74420_g1,c48359_g1,c1854_g1,c103876_g1,c1106_g1,c49485_g1,c33253_g1,c66991_g1,c15924_g1,c100829_g1,c48091_g1,c50398_g4,c10665_g1,c43419_g1,c62848_g1,c26129_g1,c38896_g1,c43513_g1,c50078_g1,c50276_g3,c25384_g1,c69554_g1,c95771_g1,c9106_g1,c111711_g1,c50398_g2,c46651_g3,c94681_g1,c97170_g1,c75725_g1,c109421_g1,c46466_g1,c31483_g1,c93724_g1,c50153_g1,c49744_g1,c91850_g1,c28863_g1,c66689_g1,c35601_g1,c86031_g1,c17004_g1,c31685_g1,c70407_g1,c62528_g1,c41123_g1,c49744_g2,c109598_g1,c29612_g2,c50564_g1,c107280_g1,c29664_g1,c105080_g1,c99356_g1,c52020_g1,c104681_g1,c51881_g2,c96785_g1,c56408_g1,c49917_g2,c96169_g1,c48899_g1,c86595_g1,c100137_g1,c35907_g2,c18120_g1,c51392_g2,c17777_g1,c45523_g1,c16392_g1,c60706_g1,c28632_g1,c26504_g1,c86663_g1,c93284_g1,c53847_g1,c84260_g1,c54783_g1,c107250_g1,c17123_g1,c106661_g1,c11124_g1,c100570_g1,c42615_g1,c50619_g1,c84203_g1,c42979_g1,c48330_g1,c25972_g2,c94283_g1,c88753_g1,c103704_g1,c72172_g1,c83962_g1,c55121_g1,c41115_g1,c77516_g1,c97989_g1,c46805_g1,c103419_g1,c25469_g1,c94013_g1,c19849_g1,c42363_g1,c103481_g1,c31760_g1,c46824_g1,c45941_g3,c97340_g1,c52523_g1,c94968_g1,c78951_g1,c34711_g1,c9260_g1,c14073_g1,c9158_g1,c35044_g1,c50321_g3,c111069_g1,c52794_g1,c105218_g1,c59388_g1,c29612_g1,c72513_g1,c78289_g1,c38893_g1,c58440_g1,c33481_g1,c82984_g1,c83451_g1,c86485_g1,c7026_g1,c45509_g1,c42289_g1,c17140_g1,c51146_g1,c51817_g1,c62057_g1,c46297_g1,c88795_g1,c6222_g1,c47743_g2,c30384_g1,c51926_g1,c15902_g1,c42680_g1,c56506_g1,c50173_g1,c109172_g1,c53249_g1,c52135_g1,c18207_g1,c71671_g1,c99364_g1,c34831_g2,c20232_g1,c38085_g1,c48456_g1,c46174_g1,c102618_g1,c64955_g1,c93659_g1 |
| Non-homologous end-joining | ko03450 | 9 | c42827_g1,c49330_g1,c72254_g1,c36138_g1,c36643_g1,c72756_g1,c49655_g1,c52290_g1,c49089_g1 |
| Nucleotide excision repair | ko03420 | 98 | c50982_g1,c48844_g1,c47667_g1,c56832_g1,c5675_g1,c70580_g1,c29237_g1,c82051_g1,c10141_g1,c52749_g2,c31541_g1,c82053_g2,c104301_g1,c43178_g1,c52039_g1,c97771_g1,c51337_g1,c96041_g1,c74282_g1,c63859_g1,c35641_g1,c101974_g1,c45652_g1,c82246_g1,c50297_g1,c63101_g1,c46244_g1,c107035_g1,c10987_g1,c44352_g1,c49889_g1,c44307_g1,c86879_g1,c99166_g1,c89167_g1,c35284_g1,c50281_g2,c45397_g1,c92670_g1,c62662_g1,c58749_g1,c73461_g1,c71958_g1,c51643_g2,c52486_g1,c63986_g1,c36481_g1,c53875_g1,c72533_g1,c48988_g2,c40194_g1,c38755_g1,c5612_g1,c96589_g1,c36262_g1,c61853_g1,c60256_g1,c34493_g1,c17238_g1,c93229_g1,c41237_g1,c56860_g1,c52200_g1,c46575_g1,c57770_g1,c51820_g1,c29085_g1,c100618_g1,c42506_g1,c47120_g1,c56658_g1,c4916_g1,c106919_g1,c40852_g1,c107335_g1,c28631_g1,c47602_g2,c108410_g1,c109692_g1,c51108_g1,c47301_g2,c49341_g3,c65713_g1,c77461_g1,c50038_g1,c104180_g1,c92988_g1,c13104_g1,c49696_g1,c41885_g1,c18649_g1,c15614_g1,c73623_g1,c52531_g1,c33371_g1,c98855_g1,c49427_g1,c64403_g1 |
| Proteasome | ko03050 | 162 | c43658_g1,c25536_g1,c103271_g1,c92900_g1,c89555_g1,c88699_g1,c55830_g1,c1063_g1,c53760_g1,c77748_g1,c44590_g1,c62235_g1,c41307_g1,c61530_g1,c27195_g1,c95175_g1,c73454_g1,c63074_g1,c96199_g1,c89288_g1,c65948_g1,c2892_g1,c64441_g1,c36821_g1,c37563_g1,c66470_g1,c82424_g1,c79969_g1,c90082_g1,c98263_g1,c39082_g3,c41253_g1,c95007_g1,c39120_g1,c43392_g1,c65498_g1,c82056_g1,c45932_g1,c80928_g1,c39196_g1,c31109_g1,c54134_g1,c96636_g1,c36474_g1,c28552_g1,c96992_g1,c78123_g1,c8277_g1,c55321_g1,c34285_g1,c3493_g1,c75650_g1,c72160_g1,c17251_g1,c28328_g1,c42036_g1,c84078_g1,c1136_g1,c15630_g1,c76268_g1,c47075_g1,c37391_g2,c66705_g1,c61786_g1,c35475_g1,c28127_g1,c59110_g1,c87525_g1,c18430_g1,c90488_g1,c7284_g1,c86457_g1,c37972_g1,c97819_g1,c5564_g1,c39082_g2,c85854_g1,c56162_g1,c89737_g1,c89524_g1,c74074_g1,c61168_g1,c59843_g1,c25325_g1,c42926_g1,c106131_g1,c103646_g1,c37708_g1,c41658_g1,c57971_g1,c59099_g1,c28351_g1,c106986_g1,c16887_g1,c34285_g2,c57275_g1,c73528_g1,c38039_g1,c4030_g1,c92979_g1,c91936_g1,c24337_g1,c11849_g1,c17713_g1,c10477_g1,c86986_g1,c82162_g1,c81470_g1,c26475_g1,c73048_g1,c10096_g1,c65079_g1,c78186_g1,c6136_g1,c41433_g1,c66674_g1,c91598_g1,c35240_g1,c110885_g1,c73057_g1,c16188_g1,c24911_g1,c66530_g1,c69270_g1,c50161_g1,c41280_g1,c101900_g1,c27453_g1,c80710_g1,c97671_g1,c80587_g1,c62985_g1,c14745_g1,c37713_g1,c53898_g1,c54513_g1,c28519_g1,c103981_g1,c20949_g1,c24406_g1,c76850_g1,c41152_g1,c95622_g1,c54548_g1,c111706_g1,c41590_g1,c61519_g1,c65618_g1,c108467_g1,c79959_g1,c43998_g1,c83600_g1,c43160_g1,c71554_g1,c41377_g1,c87226_g1,c98409_g1,c41364_g1,c38595_g1,c24769_g1,c77498_g1,c82293_g1 |
| Protein export | ko03060 | 91 | c42241_g1,c40672_g1,c97556_g1,c17158_g1,c30393_g1,c62590_g1,c17451_g1,c107314_g1,c84465_g1,c30418_g1,c44463_g1,c45083_g1,c62393_g1,c76398_g1,c77978_g1,c39157_g1,c105487_g1,c96312_g1,c98712_g1,c47905_g1,c60569_g1,c4911_g1,c43524_g2,c1904_g1,c1443_g1,c84054_g1,c30770_g1,c72742_g1,c44481_g1,c41022_g2,c55144_g1,c109997_g1,c45432_g1,c48699_g4,c46668_g1,c66917_g1,c64653_g1,c49230_g2,c53515_g1,c84044_g1,c51719_g1,c62799_g1,c57725_g1,c71581_g1,c30184_g1,c17391_g2,c48056_g1,c9986_g1,c26875_g1,c106096_g1,c35201_g1,c28233_g1,c41022_g3,c110534_g1,c39253_g1,c38150_g1,c27168_g1,c25794_g1,c99517_g1,c53395_g1,c61215_g1,c66942_g1,c50792_g1,c41022_g1,c36303_g1,c70446_g1,c18548_g1,c110797_g1,c67888_g1,c80850_g1,c41643_g1,c70131_g1,c82145_g1,c37346_g1,c72267_g1,c87045_g1,c43409_g1,c84006_g1,c107807_g1,c43661_g1,c47471_g1,c104755_g1,c62530_g1,c94120_g1,c7864_g1,c35528_g1,c16093_g1,c31732_g1,c92833_g1,c64712_g1,c4535_g1 |
| Protein processing in endoplasmic reticulum | ko04141 | 486 | c52710_g2,c55831_g1,c65434_g1,c97556_g1,c5675_g1,c43567_g2,c103765_g1,c17158_g1,c38980_g1,c8275_g1,c91578_g1,c41134_g1,c11324_g1,c83942_g1,c79265_g1,c81371_g1,c108492_g1,c95850_g1,c29237_g1,c82051_g1,c45895_g1,c79104_g1,c65741_g1,c81465_g1,c103488_g1,c31425_g1,c48439_g1,c35059_g1,c65217_g1,c42055_g1,c34801_g1,c35449_g1,c45611_g2,c37756_g1,c88761_g1,c64341_g1,c10972_g1,c60514_g1,c48436_g2,c47359_g1,c86739_g1,c31274_g1,c84465_g1,c87471_g1,c107274_g1,c51881_g1,c73263_g1,c55605_g1,c87339_g1,c82434_g1,c68830_g1,c84503_g1,c40971_g1,c98696_g1,c30744_g1,c50702_g1,c52710_g3,c65806_g1,c29861_g1,c68079_g1,c76236_g1,c94479_g1,c34532_g1,c46828_g2,c21454_g1,c297_g2,c62393_g1,c17033_g1,c49352_g3,c95765_g1,c41951_g1,c37409_g1,c35896_g1,c46682_g1,c85837_g1,c76398_g1,c36992_g1,c77978_g1,c98089_g1,c43180_g1,c39157_g1,c5979_g1,c9008_g1,c72929_g1,c61802_g1,c96409_g1,c84871_g1,c99424_g1,c58704_g1,c90199_g1,c25388_g1,c89738_g1,c96066_g1,c28859_g1,c34549_g1,c72530_g1,c24433_g1,c85426_g1,c37866_g2,c103495_g1,c4911_g1,c49027_g1,c108097_g1,c63084_g1,c43571_g1,c92924_g1,c37962_g1,c43524_g2,c42048_g1,c59352_g1,c1904_g1,c46244_g1,c46828_g3,c73749_g1,c95536_g1,c60132_g1,c30224_g1,c105941_g1,c49269_g3,c88688_g1,c47248_g1,c82762_g1,c76196_g1,c97805_g1,c13933_g1,c73322_g1,c46157_g1,c75604_g1,c107452_g1,c97513_g1,c51595_g1,c37837_g1,c72031_g1,c53758_g1,c48114_g1,c58757_g1,c104689_g1,c45475_g1,c46139_g1,c82844_g1,c77877_g1,c19117_g1,c104764_g1,c44374_g1,c56228_g1,c83187_g1,c52377_g3,c16911_g1,c40208_g1,c86879_g1,c84054_g1,c88543_g1,c55047_g1,c61897_g1,c62682_g1,c47316_g1,c69034_g1,c27003_g1,c30486_g2,c18645_g1,c48214_g1,c44481_g1,c98426_g1,c30486_g3,c22358_g1,c41022_g2,c55144_g1,c70725_g1,c94868_g1,c49828_g1,c43036_g1,c2340_g1,c109997_g1,c75980_g1,c7804_g1,c103545_g1,c54318_g1,c40095_g1,c83466_g1,c83541_g1,c53783_g1,c53046_g1,c71422_g1,c36117_g1,c48699_g4,c83103_g1,c59360_g1,c57404_g1,c703_g1,c9035_g1,c18330_g1,c47186_g1,c47459_g1,c97427_g1,c43298_g1,c59458_g1,c41912_g1,c69319_g1,c55531_g1,c107131_g1,c39066_g1,c65384_g1,c52522_g1,c97777_g1,c64653_g1,c110650_g1,c49230_g2,c97101_g1,c50843_g1,c53622_g1,c30107_g1,c46148_g1,c84044_g1,c56887_g1,c99321_g1,c101207_g1,c55960_g1,c84476_g1,c75045_g1,c24816_g1,c83184_g1,c47138_g1,c61491_g1,c54919_g1,c11693_g1,c67129_g1,c53821_g1,c65797_g1,c62662_g1,c57725_g1,c58749_g1,c30184_g1,c42491_g1,c17391_g2,c27942_g1,c92790_g2,c48108_g1,c48306_g1,c95433_g1,c26875_g1,c26912_g1,c8448_g1,c37300_g1,c106096_g1,c47187_g1,c20191_g1,c67855_g1,c53443_g1,c87756_g1,c82688_g1,c32310_g2,c42033_g1,c92599_g1,c51938_g1,c35201_g1,c30486_g1,c54734_g1,c31830_g1,c70327_g1,c52022_g1,c39157_g2,c28233_g1,c100725_g1,c65057_g1,c41022_g3,c7457_g1,c26221_g1,c77560_g1,c31410_g1,c53340_g1,c108127_g1,c86026_g1,c52598_g1,c42639_g1,c38150_g1,c98969_g1,c73801_g1,c47763_g1,c107655_g1,c82641_g1,c13183_g1,c103460_g1,c54833_g1,c89618_g1,c62673_g1,c71807_g1,c34581_g1,c38552_g2,c87325_g1,c49633_g1,c23873_g1,c48877_g1,c96576_g1,c63003_g1,c2722_g1,c51511_g2,c50875_g1,c4631_g1,c62084_g1,c94623_g1,c10943_g1,c40194_g1,c18552_g1,c104751_g1,c54723_g1,c30518_g1,c74411_g1,c63919_g1,c39145_g2,c104110_g1,c103608_g1,c43067_g2,c88796_g1,c47975_g1,c104810_g1,c56715_g1,c54926_g1,c49817_g1,c79194_g1,c36740_g1,c5215_g1,c107217_g1,c47810_g2,c186_g1,c48418_g1,c46688_g2,c103497_g1,c45570_g1,c102073_g1,c47616_g1,c43070_g1,c18935_g1,c105070_g1,c16266_g1,c82328_g1,c36058_g1,c41022_g1,c62491_g1,c61853_g1,c62353_g1,c44318_g1,c60256_g1,c50218_g2,c44852_g1,c46828_g1,c3197_g1,c44286_g1,c15285_g1,c71912_g1,c68010_g1,c74058_g1,c80850_g1,c41643_g1,c65129_g1,c53980_g1,c73509_g1,c87049_g1,c82327_g1,c45436_g1,c65754_g1,c72267_g1,c45236_g1,c3963_g1,c45167_g2,c106274_g1,c66713_g1,c75222_g1,c94352_g1,c71954_g1,c62342_g1,c97219_g1,c77529_g1,c45027_g1,c34941_g2,c43308_g1,c84850_g1,c54690_g1,c58221_g1,c29673_g1,c59077_g1,c37708_g2,c106851_g1,c19485_g1,c40383_g1,c101193_g1,c66322_g1,c4813_g1,c27179_g2,c16213_g1,c99231_g1,c57085_g1,c26951_g1,c17193_g1,c48111_g1,c104744_g1,c32310_g1,c13172_g1,c40516_g1,c19536_g1,c50702_g2,c45297_g1,c82349_g1,c109031_g1,c75449_g1,c64428_g1,c49243_g1,c42165_g1,c47043_g1,c105795_g1,c49687_g1,c75365_g1,c34812_g1,c28968_g1,c46514_g1,c4455_g1,c56377_g1,c8895_g1,c56658_g1,c55979_g1,c31158_g1,c62421_g1,c50576_g1,c91146_g1,c34941_g1,c19107_g1,c104206_g1,c55706_g1,c57958_g1,c86000_g1,c50617_g1,c16093_g1,c50298_g1,c48200_g1,c96864_g1,c49610_g1,c54350_g1,c43036_g2,c96185_g1,c67459_g1,c46736_g1,c49161_g1,c73431_g1,c688_g1,c13411_g1,c56533_g1,c28421_g1,c112008_g1,c4665_g1,c90122_g1,c57174_g1,c50445_g1,c110498_g1,c47666_g1,c107618_g1,c45552_g1,c25487_g1,c64712_g1,c9281_g1,c74430_g1,c39145_g1,c56943_g1,c34348_g1,c74644_g1,c94169_g1,c19301_g1,c7901_g1,c47361_g1,c42608_g1,c102593_g1,c46696_g1,c18181_g1,c15614_g1,c4535_g1,c60836_g1,c71833_g1,c46852_g1,c104947_g1,c105116_g1,c29335_g1,c95392_g1,c45611_g1,c110452_g1,c61718_g1,c36762_g1,c64403_g1,c16677_g1 |
| Ribosome | ko03010 | 1010 | c31326_g1,c73763_g1,c38829_g1,c82376_g1,c95497_g1,c53822_g1,c55018_g1,c26698_g1,c7107_g1,c53834_g1,c110106_g1,c12370_g1,c111947_g1,c111148_g1,c27273_g1,c42864_g1,c48716_g1,c72926_g1,c84074_g1,c25187_g1,c96861_g1,c48983_g1,c45791_g2,c24542_g1,c104015_g1,c106662_g1,c24468_g1,c76411_g1,c38889_g1,c67588_g1,c41682_g2,c95572_g1,c38625_g1,c38819_g1,c84666_g1,c13376_g1,c23459_g1,c88642_g1,c38339_g1,c3943_g1,c61749_g1,c40364_g1,c32647_g1,c83647_g1,c93414_g1,c49614_g1,c78587_g1,c70022_g1,c95125_g1,c64228_g1,c54827_g1,c72605_g1,c89059_g1,c65568_g1,c34628_g1,c4091_g1,c61062_g1,c31398_g1,c30647_g1,c92196_g1,c90882_g1,c63929_g1,c84048_g1,c104694_g1,c99990_g1,c22922_g1,c54509_g1,c83774_g1,c106330_g1,c10099_g1,c2055_g1,c50738_g2,c30904_g2,c48969_g2,c58455_g1,c53669_g1,c11884_g1,c25581_g1,c13841_g1,c45822_g1,c93436_g1,c98650_g1,c53302_g1,c55688_g1,c28427_g3,c74938_g1,c97334_g1,c44625_g1,c34917_g1,c22858_g1,c24987_g1,c19343_g1,c37384_g1,c36053_g1,c108707_g1,c29260_g1,c65504_g1,c17775_g1,c78187_g1,c28881_g1,c32271_g1,c96470_g1,c92814_g1,c43865_g1,c26588_g1,c26520_g1,c25801_g1,c95271_g1,c92855_g1,c42743_g1,c98034_g1,c13704_g1,c102625_g1,c88279_g1,c29099_g1,c94838_g1,c103029_g1,c38440_g1,c57632_g1,c35275_g2,c19429_g1,c44282_g1,c58195_g1,c35688_g1,c58034_g1,c77777_g1,c28673_g2,c29205_g1,c9218_g1,c38126_g1,c78890_g1,c95739_g1,c103285_g1,c107684_g1,c78898_g1,c54038_g1,c26049_g1,c26295_g1,c1967_g1,c77369_g1,c110113_g1,c36759_g2,c35798_g1,c16877_g1,c113223_g1,c85483_g1,c4628_g1,c83619_g1,c100209_g1,c100283_g1,c89557_g1,c38650_g2,c54654_g1,c87033_g1,c29641_g1,c72703_g1,c80938_g1,c42315_g2,c13855_g1,c2158_g1,c103353_g1,c107383_g1,c12774_g1,c10717_g1,c84001_g1,c73726_g1,c37650_g1,c40356_g1,c77373_g1,c84459_g1,c41709_g1,c72950_g1,c19288_g1,c74147_g1,c109704_g1,c104756_g1,c78879_g1,c36012_g1,c94442_g1,c94121_g1,c85170_g1,c29436_g1,c87630_g1,c46488_g2,c26730_g1,c35963_g1,c6672_g1,c85488_g1,c104791_g1,c41455_g1,c110072_g1,c95032_g1,c113243_g1,c83688_g1,c26472_g1,c94171_g1,c52317_g3,c58558_g1,c104672_g1,c48821_g1,c64806_g1,c105673_g1,c52268_g2,c20008_g1,c20167_g1,c34287_g3,c83793_g1,c46494_g1,c59662_g1,c22909_g1,c111374_g1,c3485_g1,c85838_g1,c98666_g1,c110061_g1,c67469_g1,c43504_g1,c53481_g1,c95574_g1,c8671_g1,c54954_g1,c38096_g1,c45792_g1,c72798_g1,c65680_g1,c61224_g1,c66231_g1,c88926_g1,c110509_g1,c99035_g1,c13703_g1,c28845_g1,c103349_g1,c6914_g1,c28427_g2,c53360_g1,c15633_g1,c95577_g1,c54893_g1,c108145_g1,c80650_g1,c54633_g1,c25250_g1,c22103_g1,c18318_g1,c34095_g1,c98541_g1,c25108_g1,c41893_g1,c54373_g1,c22681_g1,c104054_g1,c11709_g1,c5670_g1,c94241_g1,c43715_g1,c66785_g1,c52317_g4,c49224_g1,c109938_g1,c111354_g1,c27032_g1,c63346_g1,c68177_g1,c65483_g1,c42277_g1,c93103_g1,c99947_g1,c106037_g1,c34321_g1,c95623_g1,c74222_g1,c6034_g1,c18316_g1,c35219_g1,c29046_g1,c35275_g1,c34287_g2,c7487_g1,c39233_g1,c17518_g1,c60842_g1,c87680_g1,c81246_g1,c47920_g1,c41538_g1,c24341_g1,c41742_g1,c38597_g1,c72135_g1,c29532_g1,c102616_g1,c99549_g1,c44393_g1,c35366_g1,c42743_g2,c28259_g1,c41572_g1,c49352_g1,c111829_g1,c99654_g1,c109253_g1,c41502_g1,c1310_g1,c112213_g1,c82171_g1,c100540_g1,c38_g1,c38198_g1,c93687_g1,c18441_g1,c41682_g1,c50313_g2,c41211_g2,c77933_g1,c73874_g1,c54762_g1,c88373_g1,c35325_g1,c93637_g1,c68174_g1,c17741_g1,c9065_g1,c37215_g1,c31178_g1,c30081_g1,c61966_g1,c38745_g2,c20366_g1,c31453_g1,c111452_g1,c9181_g1,c31324_g1,c82370_g1,c53907_g1,c93463_g1,c100986_g1,c111959_g1,c2593_g1,c105643_g1,c4001_g1,c65160_g1,c42001_g1,c19392_g1,c103132_g1,c76968_g1,c39235_g1,c25197_g1,c52332_g4,c5061_g1,c103480_g1,c9131_g1,c82471_g1,c87917_g1,c70786_g1,c86612_g1,c39935_g2,c91205_g1,c82360_g1,c96172_g1,c58248_g1,c17537_g1,c72402_g1,c111159_g1,c102307_g1,c77053_g1,c56301_g1,c54438_g1,c18046_g1,c76460_g1,c75993_g1,c38271_g1,c72758_g1,c43449_g1,c81933_g1,c7328_g1,c97157_g1,c90659_g1,c102116_g1,c65102_g1,c102637_g1,c13347_g1,c19954_g1,c13834_g1,c71173_g1,c41410_g1,c22989_g1,c84467_g1,c95759_g1,c9088_g1,c78233_g1,c53913_g1,c65519_g1,c26811_g1,c28498_g1,c40982_g1,c53486_g1,c107686_g1,c95260_g1,c95598_g1,c21250_g2,c67094_g1,c39832_g1,c37608_g1,c109888_g1,c35157_g1,c110817_g1,c67796_g1,c55295_g1,c105659_g1,c104691_g1,c66004_g1,c95983_g1,c64038_g1,c21400_g1,c99201_g1,c17134_g1,c35920_g1,c110682_g1,c46440_g1,c72068_g1,c3721_g1,c60152_g1,c31906_g1,c74041_g1,c80374_g1,c32965_g1,c109036_g1,c90163_g1,c67187_g1,c45196_g1,c101566_g1,c103636_g1,c67268_g1,c99681_g1,c15662_g1,c105397_g1,c100062_g1,c109430_g1,c37316_g1,c44087_g1,c33901_g1,c66199_g1,c37503_g1,c99699_g1,c44619_g1,c93587_g1,c30353_g1,c76675_g1,c108271_g1,c30738_g1,c21674_g1,c102762_g1,c101916_g1,c36759_g1,c40220_g1,c76379_g1,c57363_g1,c107652_g1,c84270_g1,c44132_g1,c62541_g1,c95941_g1,c72671_g1,c59124_g1,c87432_g1,c35733_g1,c75872_g1,c53995_g1,c54655_g1,c103068_g1,c34729_g1,c102323_g1,c83858_g1,c9341_g1,c100582_g1,c23459_g2,c537_g1,c84884_g1,c59832_g1,c104619_g1,c83806_g1,c12320_g1,c93172_g1,c57936_g1,c29093_g1,c23679_g1,c18134_g1,c63127_g1,c10881_g1,c62475_g1,c16972_g1,c109466_g1,c65719_g1,c83615_g1,c45428_g3,c30069_g1,c79815_g1,c77173_g1,c25214_g1,c42840_g1,c33647_g1,c63018_g1,c93867_g1,c36282_g1,c30736_g1,c18297_g1,c3008_g1,c23854_g1,c30258_g1,c10592_g1,c107275_g1,c2097_g1,c94293_g1,c40142_g2,c41917_g1,c109790_g1,c38195_g1,c93986_g1,c29091_g1,c79905_g1,c68187_g1,c47379_g1,c30780_g1,c93980_g1,c46840_g1,c108015_g1,c22407_g1,c76399_g1,c103913_g1,c34498_g1,c30978_g1,c83415_g1,c99700_g1,c38228_g1,c24514_g1,c29492_g1,c59027_g1,c57996_g1,c86448_g1,c78267_g1,c80854_g1,c81180_g1,c91325_g1,c104046_g1,c90693_g1,c81984_g1,c77078_g1,c66296_g1,c9189_g1,c80101_g1,c58606_g1,c95693_g1,c13453_g1,c85821_g1,c87263_g1,c76238_g1,c17135_g1,c24943_g1,c98652_g1,c69489_g1,c93067_g1,c3937_g1,c109757_g1,c94587_g1,c103454_g1,c27299_g1,c32972_g1,c62097_g1,c53976_g1,c24948_g1,c7813_g1,c44819_g1,c84075_g1,c94695_g1,c18285_g1,c25679_g1,c1004_g1,c95988_g1,c22768_g1,c99726_g1,c6393_g1,c34930_g1,c41546_g1,c107664_g1,c87694_g1,c37437_g1,c78886_g1,c41211_g1,c4122_g1,c43188_g1,c96971_g1,c74013_g1,c78109_g1,c38939_g1,c38998_g1,c46504_g1,c54177_g1,c103575_g1,c76249_g1,c36197_g1,c95493_g1,c21250_g1,c34740_g1,c10924_g1,c34924_g1,c8684_g1,c36442_g1,c85820_g1,c62419_g1,c30014_g1,c24570_g1,c72058_g1,c9067_g1,c104024_g1,c53183_g1,c2856_g1,c42369_g1,c1296_g1,c68364_g1,c6560_g1,c94307_g1,c4307_g1,c27140_g1,c43225_g1,c19568_g2,c36271_g1,c34704_g1,c88863_g1,c15125_g1,c68111_g1,c30985_g2,c89961_g1,c24642_g1,c4159_g1,c73040_g1,c42161_g3,c59861_g1,c109592_g1,c68038_g1,c82485_g1,c85152_g1,c63020_g1,c84550_g1,c16821_g1,c63549_g1,c10996_g1,c35099_g1,c53242_g1,c53082_g1,c16910_g1,c87460_g1,c88742_g1,c112296_g1,c96132_g1,c91771_g1,c19090_g1,c241_g1,c16874_g1,c66927_g1,c44036_g1,c93963_g1,c17940_g1,c14652_g1,c110320_g1,c94982_g1,c83447_g1,c59999_g1,c41109_g1,c34983_g1,c59378_g1,c58591_g1,c96702_g1,c33947_g1,c35953_g1,c37766_g1,c31493_g2,c84673_g1,c28940_g1,c21072_g1,c74394_g1,c23992_g1,c106597_g1,c40820_g1,c32745_g1,c26064_g1,c57874_g1,c28617_g1,c9233_g1,c81432_g1,c104895_g1,c22850_g1,c42940_g1,c78377_g1,c25703_g1,c35539_g1,c40976_g1,c33331_g1,c62808_g1,c26496_g1,c8199_g1,c91822_g1,c16241_g1,c55446_g1,c28427_g1,c86883_g1,c12031_g1,c88063_g1,c9563_g1,c78219_g1,c71914_g1,c17838_g1,c98404_g1,c29105_g1,c35056_g1,c108166_g1,c39046_g1,c105018_g1,c26933_g1,c82474_g1,c73334_g1,c53282_g1,c107706_g1,c109018_g1,c1055_g1,c64243_g1,c40457_g1,c19873_g1,c10714_g1,c77497_g1,c76691_g1,c74412_g1,c2814_g1,c42174_g4,c98794_g1,c57390_g1,c82166_g1,c28297_g1,c102777_g1,c67940_g1,c61145_g2,c39598_g1,c62783_g1,c94152_g1,c44930_g1,c68266_g1,c69378_g1,c8940_g1,c54855_g1,c11364_g1,c6178_g1,c42589_g1,c86735_g1,c68286_g1,c71766_g1,c111187_g1,c55703_g1,c45701_g2,c100583_g1,c24786_g1,c44664_g2,c27136_g1,c55225_g1,c97086_g1,c62043_g1,c46270_g1,c83820_g1,c73807_g1,c24201_g2,c94248_g1,c30904_g1,c45542_g1,c27614_g1,c54358_g1,c72341_g1,c25143_g1,c46355_g1,c31184_g1,c52930_g1,c89110_g1,c90989_g1,c55790_g1,c104174_g1,c26996_g1,c57907_g1,c26261_g1,c22768_g2,c76605_g1,c35583_g2,c8951_g1,c33901_g2,c75016_g1,c61314_g1,c26270_g1,c84867_g1,c83743_g1,c22065_g1,c11862_g1,c100786_g1,c109391_g1,c93079_g1,c77146_g1,c43504_g2,c301_g1,c70943_g1,c12514_g1,c12123_g1,c27199_g1,c59948_g1,c54781_g1,c17007_g1,c34287_g1,c27239_g1,c19568_g1,c86440_g1,c109114_g1,c30348_g1,c29714_g1,c30985_g1,c41193_g1,c57691_g1,c56875_g1,c43949_g1,c31327_g1,c59010_g1,c73603_g1,c82063_g1,c74101_g1,c31310_g1,c83825_g1,c54867_g1,c57842_g1,c44058_g1,c40103_g2,c78727_g1,c103512_g1,c47725_g1,c35868_g1,c59216_g1,c73123_g1,c27528_g1,c109385_g1,c87393_g1,c16510_g1,c72421_g1,c108261_g1,c16681_g1,c85726_g1,c64922_g1,c18501_g1,c87311_g1,c39961_g1,c30312_g1,c33914_g1,c54975_g1,c76159_g1,c34939_g1,c75612_g1,c76900_g1,c79147_g1,c37780_g1,c24201_g1,c43605_g1,c66353_g1,c5221_g1,c79338_g1,c18266_g1,c82189_g1,c73715_g1,c29380_g1,c38719_g1,c106262_g1,c96108_g1,c44202_g1,c41854_g1,c16978_g1,c13055_g1,c37743_g1,c92841_g1,c100181_g1,c100944_g1,c84732_g1,c93219_g1,c56427_g1,c58185_g1,c35616_g1,c30477_g1,c18781_g1,c12348_g1,c20495_g1,c62790_g1,c68765_g1,c51689_g2,c71645_g1,c19288_g2,c94190_g1,c5069_g1,c93399_g1,c94428_g1,c47692_g1,c28476_g1,c61223_g1,c11166_g1,c40150_g1,c103939_g1,c39935_g1,c18133_g1,c94898_g1,c38650_g1,c97443_g1,c36515_g1,c79915_g1,c67179_g1,c41024_g1,c40211_g1,c49279_g1,c66281_g1,c35796_g1,c26695_g1,c28350_g1,c28673_g1,c109409_g1,c111912_g1,c19860_g1,c104347_g1,c75709_g1,c76202_g1,c93827_g1,c65402_g1,c110271_g1,c97232_g1,c45701_g1,c76026_g1,c66123_g1,c90682_g1,c39085_g1,c79234_g1,c40142_g1,c108693_g1,c19249_g1,c68257_g1,c39503_g1,c27772_g1,c109507_g1,c85375_g1,c21784_g1,c26455_g1,c26898_g1,c11165_g1,c68078_g1,c47959_g1,c93174_g1,c93239_g1,c6211_g1,c54442_g1,c34016_g1,c25421_g1,c9025_g1,c41326_g1,c41532_g1,c75104_g1,c17857_g1,c10752_g1,c40498_g1,c1285_g1,c34065_g1,c16106_g1,c12059_g1,c70714_g1,c72009_g1,c77847_g1,c26839_g1,c86107_g1,c56878_g1,c37023_g1,c95741_g1,c34774_g1,c39073_g1,c21803_g1,c104752_g1,c10687_g1,c56572_g1,c61711_g1,c24542_g2,c94604_g1,c79257_g1,c79823_g1,c93630_g1,c24232_g1,c30211_g1,c19435_g1,c9795_g1,c91033_g1,c112324_g1,c31493_g1,c105715_g1,c43922_g1,c99524_g1,c48600_g1,c67255_g1,c94444_g1,c33848_g1,c71677_g1,c44124_g1,c41876_g1,c95301_g1,c75836_g1,c70727_g1,c71758_g1,c103276_g1 |
| Ribosome biogenesis in eukaryotes | ko03008 | 122 | c76828_g1,c75285_g1,c51182_g1,c79117_g1,c34282_g1,c75900_g1,c50516_g2,c46435_g1,c36043_g1,c55141_g1,c99899_g1,c38003_g1,c86184_g1,c27351_g1,c98724_g1,c87773_g1,c26800_g1,c38079_g1,c38947_g1,c35534_g1,c51649_g1,c4235_g1,c92788_g1,c47258_g1,c43306_g1,c54429_g1,c56496_g1,c74711_g1,c41474_g1,c109731_g1,c111102_g1,c50913_g1,c46386_g1,c100645_g1,c77015_g1,c109107_g1,c3455_g1,c67873_g1,c18139_g1,c51589_g2,c47110_g1,c72321_g1,c9389_g1,c109499_g1,c50344_g1,c52508_g12,c40152_g1,c49899_g1,c9930_g1,c51512_g1,c103972_g1,c33924_g1,c37738_g1,c40398_g1,c47751_g1,c28865_g1,c52977_g1,c35898_g1,c106085_g1,c67885_g1,c43323_g1,c38408_g1,c49874_g1,c33795_g1,c15936_g1,c17095_g1,c50847_g1,c10748_g1,c60808_g1,c40472_g1,c107458_g1,c35119_g1,c35915_g1,c46664_g1,c95538_g1,c64081_g1,c50495_g2,c51722_g1,c49766_g1,c80007_g1,c52276_g1,c17233_g1,c52889_g1,c41238_g1,c88069_g1,c82597_g1,c84522_g1,c51554_g1,c104758_g1,c50770_g1,c7965_g1,c11351_g1,c62976_g1,c38527_g1,c45126_g1,c72071_g1,c62154_g1,c24646_g1,c56627_g1,c100338_g1,c102619_g1,c98707_g1,c49658_g1,c59536_g1,c47849_g1,c49956_g1,c98091_g1,c46064_g1,c95600_g1,c52052_g1,c55886_g1,c107967_g1,c51512_g5,c41813_g1,c106578_g1,c76754_g1,c95993_g1,c57678_g1,c43448_g1,c65776_g1,c50516_g1,c49000_g1 |
| RNA degradation | ko03018 | 183 | c44423_g1,c38356_g1,c51182_g1,c31667_g1,c31021_g1,c42550_g1,c53212_g1,c73735_g1,c110633_g1,c32142_g1,c96551_g1,c50352_g1,c41791_g1,c48709_g1,c50698_g1,c100050_g1,c46404_g4,c97107_g1,c36924_g1,c94546_g1,c38776_g1,c96933_g1,c105045_g1,c101090_g1,c46404_g1,c61949_g1,c38760_g1,c51840_g8,c43582_g1,c25101_g1,c13889_g1,c39676_g1,c61047_g1,c50274_g1,c40092_g1,c52057_g2,c73840_g1,c7395_g1,c3955_g1,c82241_g2,c104026_g1,c91638_g1,c54185_g1,c71570_g1,c1291_g1,c111368_g1,c18147_g1,c106435_g1,c54076_g1,c39664_g1,c51257_g1,c108910_g1,c84924_g1,c28040_g1,c67603_g1,c103268_g1,c46619_g1,c51489_g1,c61423_g1,c56831_g1,c50653_g2,c63385_g1,c49485_g1,c68919_g1,c74934_g1,c2545_g1,c76028_g1,c60359_g1,c100829_g1,c88748_g1,c85117_g1,c62844_g1,c52017_g5,c28034_g1,c56971_g1,c98092_g1,c49659_g4,c44872_g1,c52228_g1,c96174_g1,c40413_g1,c69639_g1,c51512_g1,c79946_g1,c37475_g1,c52162_g3,c51425_g1,c52416_g2,c109380_g1,c50149_g1,c9106_g1,c48823_g1,c50149_g3,c46466_g1,c93724_g1,c49744_g1,c28863_g1,c4972_g1,c50653_g1,c40396_g1,c65964_g1,c51184_g1,c49744_g2,c45934_g1,c54211_g1,c43169_g3,c51107_g1,c38260_g1,c29136_g1,c55357_g1,c29664_g1,c104681_g1,c13865_g1,c38966_g2,c42750_g1,c70337_g1,c49758_g1,c86595_g1,c72525_g1,c83146_g1,c17777_g1,c101079_g1,c47238_g1,c48470_g1,c61213_g1,c90874_g1,c60706_g1,c39352_g1,c74442_g1,c49983_g2,c54783_g1,c107250_g1,c9043_g1,c84763_g1,c66208_g1,c100570_g1,c25972_g2,c46124_g2,c41115_g1,c49864_g1,c50797_g1,c40413_g2,c14975_g1,c58823_g1,c31760_g1,c41069_g1,c18260_g1,c79072_g1,c65922_g1,c77042_g1,c64196_g1,c46404_g3,c9158_g1,c40993_g1,c105354_g1,c48048_g1,c109908_g1,c83666_g1,c42117_g1,c13202_g1,c50024_g1,c65521_g1,c7026_g1,c104287_g1,c5358_g1,c98995_g1,c87776_g1,c96465_g1,c99838_g1,c49607_g2,c51512_g5,c51712_g1,c26649_g1,c50024_g2,c85883_g1,c71671_g1,c46404_g2,c89702_g1,c73846_g1,c34654_g1,c65776_g1,c49356_g1,c71568_g2 |
| RNA polymerase | ko03020 | 69 | c89549_g1,c95531_g1,c95720_g1,c56099_g1,c49880_g1,c50327_g6,c94579_g1,c85703_g1,c43221_g1,c55119_g1,c11071_g1,c97623_g1,c10225_g1,c49636_g1,c38201_g1,c33339_g1,c39195_g1,c62495_g1,c50327_g5,c37733_g1,c24358_g1,c35699_g1,c88666_g1,c36570_g1,c109268_g1,c104730_g1,c40339_g1,c52388_g3,c48395_g1,c47111_g1,c52074_g1,c61778_g1,c65126_g1,c92888_g1,c26379_g1,c46164_g2,c57919_g1,c38372_g1,c46200_g1,c53147_g1,c26021_g1,c110063_g1,c109205_g1,c82103_g1,c64849_g1,c108921_g1,c9491_g1,c91366_g1,c38259_g1,c46159_g1,c56336_g1,c845_g1,c60503_g1,c91919_g1,c16610_g1,c83832_g1,c4230_g1,c51064_g1,c77864_g1,c105046_g1,c37556_g1,c49231_g2,c96667_g1,c51562_g1,c104305_g1,c40838_g1,c27214_g1,c31515_g1,c108828_g1 |
| RNA transport | ko03013 | 338 | c76994_g1,c47635_g2,c28835_g1,c51781_g3,c28695_g1,c4360_g1,c51342_g1,c30046_g1,c3826_g1,c31021_g1,c42550_g1,c90691_g1,c51341_g3,c50114_g2,c4287_g1,c28858_g1,c40162_g1,c62815_g1,c5381_g1,c6040_g1,c65751_g1,c82872_g1,c50516_g2,c73194_g1,c43103_g1,c82150_g1,c56873_g1,c91743_g1,c110930_g1,c40511_g1,c31799_g1,c82163_g1,c41943_g1,c91734_g1,c73403_g1,c58723_g1,c52544_g1,c52421_g1,c61685_g1,c55141_g1,c84503_g1,c107134_g1,c50861_g2,c38622_g1,c64334_g1,c58208_g1,c29979_g1,c63521_g1,c40363_g1,c79316_g1,c81287_g1,c84759_g1,c46950_g1,c10588_g1,c42333_g1,c52446_g1,c101090_g1,c9619_g1,c38947_g1,c51982_g3,c19306_g1,c107638_g1,c82404_g1,c26033_g1,c79325_g1,c62522_g1,c38283_g1,c24356_g1,c70685_g1,c3955_g1,c104747_g1,c104832_g1,c93995_g1,c92924_g1,c17127_g1,c15664_g1,c46195_g1,c89298_g1,c28587_g1,c87327_g1,c45881_g1,c56690_g1,c78956_g1,c87458_g1,c62237_g1,c92800_g1,c107522_g1,c50462_g1,c47310_g1,c43484_g1,c108910_g1,c24588_g1,c20194_g2,c78926_g1,c50678_g1,c51595_g1,c46782_g1,c59248_g1,c103268_g1,c44343_g1,c60125_g1,c53442_g1,c101438_g1,c50986_g3,c100082_g1,c91989_g1,c74420_g1,c48359_g1,c47745_g1,c106447_g1,c38103_g1,c49485_g1,c18320_g1,c72334_g1,c94555_g1,c50635_g1,c44883_g1,c30539_g1,c49307_g3,c18187_g1,c100829_g1,c75972_g1,c48091_g1,c47754_g1,c73030_g1,c58517_g1,c78640_g1,c80552_g1,c109499_g1,c43441_g1,c19234_g1,c62848_g1,c46095_g1,c52508_g12,c50268_g1,c98157_g1,c75045_g1,c15959_g1,c43513_g1,c16727_g1,c98736_g1,c47007_g1,c48911_g1,c80750_g1,c40398_g1,c84328_g1,c95771_g1,c9106_g1,c55300_g1,c79179_g1,c97170_g1,c63184_g1,c75725_g1,c33954_g1,c103370_g1,c46466_g1,c31483_g1,c93724_g1,c50153_g1,c49744_g1,c28863_g1,c49726_g1,c68178_g1,c65958_g1,c86031_g1,c31685_g1,c41123_g1,c67885_g1,c49744_g2,c110020_g1,c17511_g1,c33795_g1,c53812_g1,c54001_g1,c17095_g1,c50564_g1,c77067_g1,c29664_g1,c50847_g1,c41055_g3,c10748_g1,c104681_g1,c50493_g1,c49043_g1,c51881_g2,c9273_g1,c71685_g1,c100589_g1,c45881_g2,c6972_g1,c89618_g1,c103899_g1,c56717_g1,c47382_g1,c38910_g2,c86595_g1,c87034_g1,c100137_g1,c18120_g1,c107458_g1,c37667_g1,c17061_g1,c14355_g1,c46095_g2,c51392_g2,c17777_g1,c82890_g1,c45523_g1,c40570_g1,c60706_g1,c95538_g1,c86663_g1,c39145_g2,c62060_g1,c48322_g1,c52058_g1,c51631_g1,c70527_g1,c84260_g1,c54783_g1,c29199_g1,c107250_g1,c106661_g1,c68869_g1,c80007_g1,c105835_g1,c100570_g1,c100764_g1,c50970_g1,c43717_g1,c15525_g1,c25972_g2,c78027_g1,c67653_g1,c55121_g1,c41115_g1,c101532_g1,c80058_g1,c16490_g1,c52323_g1,c49325_g1,c46878_g1,c88069_g1,c50541_g1,c78352_g1,c99689_g1,c61821_g1,c42378_g1,c50764_g1,c65714_g1,c49838_g1,c85026_g1,c112117_g1,c34831_g1,c31760_g1,c51760_g1,c39727_g1,c38910_g1,c63746_g1,c94968_g1,c46095_g3,c112428_g1,c109313_g1,c37107_g1,c112229_g1,c11351_g1,c93695_g1,c72071_g1,c14073_g1,c63610_g1,c52387_g1,c9158_g1,c33571_g1,c104744_g1,c52647_g1,c48344_g1,c112607_g1,c105218_g1,c37302_g1,c92929_g1,c16721_g1,c52734_g2,c9080_g1,c18568_g1,c12427_g1,c76643_g1,c47068_g1,c73760_g1,c74747_g1,c20306_g1,c33481_g1,c57096_g1,c40436_g1,c103453_g1,c52136_g1,c95849_g1,c103754_g1,c31674_g1,c83451_g1,c65315_g1,c7026_g1,c45846_g1,c63426_g1,c73311_g1,c61405_g1,c11837_g1,c46001_g1,c47286_g1,c23544_g1,c43531_g1,c48123_g1,c2268_g1,c107967_g1,c44193_g1,c47666_g1,c110614_g1,c47377_g1,c65325_g1,c11645_g1,c39145_g1,c17982_g1,c52651_g1,c8333_g1,c51873_g1,c97748_g1,c52135_g1,c110549_g1,c16838_g1,c52975_g1,c71671_g1,c33571_g2,c34831_g2,c3826_g2,c73428_g1,c102618_g1,c64955_g1,c50516_g1,c93659_g1 |
| SNARE interactions in vesicular transport | ko04130 | 37 | c13238_g1,c40861_g1,c44948_g1,c12854_g1,c103426_g1,c43279_g1,c39200_g1,c68501_g1,c10768_g1,c14608_g1,c25111_g1,c25445_g1,c33971_g2,c47859_g2,c48633_g1,c36573_g1,c72001_g1,c19035_g1,c19139_g1,c9054_g1,c43065_g1,c35711_g1,c45252_g1,c43673_g1,c103760_g1,c95834_g1,c39451_g1,c52843_g2,c93984_g1,c41083_g2,c45438_g1,c28286_g1,c37158_g1,c39546_g1,c36808_g1,c672_g1,c16955_g1 |
| Spliceosome | ko03040 | 442 | c47635_g2,c75285_g1,c71988_g1,c86220_g1,c63141_g1,c105024_g1,c39735_g2,c79117_g1,c50341_g1,c91578_g1,c90691_g1,c83800_g1,c57048_g1,c35793_g1,c38582_g1,c81465_g1,c45465_g1,c92694_g1,c30490_g1,c62815_g1,c87214_g1,c646_g1,c98973_g1,c82872_g1,c8820_g1,c81710_g1,c20356_g1,c34611_g1,c64390_g1,c82150_g1,c178_g1,c46413_g5,c96610_g1,c87471_g1,c52568_g1,c58723_g1,c55501_g1,c90609_g1,c68830_g1,c40971_g1,c61992_g1,c65806_g1,c45624_g1,c77526_g1,c81287_g1,c46828_g2,c94742_g1,c95765_g1,c77639_g1,c20274_g1,c76791_g1,c61949_g1,c38760_g1,c3999_g1,c79325_g1,c5979_g1,c13889_g1,c93554_g1,c49430_g2,c100328_g1,c37374_g1,c84871_g1,c56430_g1,c99424_g1,c113238_g1,c90199_g1,c96066_g1,c104747_g1,c110289_g1,c40275_g1,c71836_g1,c49003_g1,c77131_g1,c37866_g2,c63084_g1,c103012_g1,c90712_g1,c104643_g1,c13949_g1,c6129_g1,c46828_g3,c56496_g1,c60132_g1,c30224_g1,c43024_g1,c88688_g1,c47248_g1,c112784_g1,c56690_g1,c76196_g1,c6193_g1,c97805_g1,c73322_g1,c51883_g1,c99697_g1,c75604_g1,c86004_g1,c50678_g1,c53327_g1,c38845_g1,c77549_g1,c104689_g1,c24649_g1,c31049_g1,c19117_g1,c34507_g1,c78950_g1,c101438_g1,c97507_g1,c52067_g1,c655_g1,c46746_g1,c34130_g1,c100082_g1,c94367_g1,c31782_g1,c48359_g1,c109771_g1,c79728_g1,c9379_g1,c27003_g1,c47338_g1,c98426_g1,c84926_g1,c43036_g1,c89016_g1,c75980_g1,c58297_g1,c99815_g1,c94897_g1,c98348_g1,c52404_g2,c44243_g1,c23379_g1,c48091_g1,c19895_g1,c71422_g1,c36117_g1,c33206_g1,c39265_g1,c28271_g1,c97452_g1,c41808_g1,c36405_g1,c48461_g1,c48905_g2,c59458_g1,c31026_g1,c12966_g1,c103641_g1,c85401_g1,c49165_g1,c90611_g1,c97101_g1,c62848_g1,c73055_g1,c79564_g1,c30640_g1,c99503_g1,c176_g1,c99321_g1,c45070_g1,c50268_g1,c43513_g1,c86593_g1,c49684_g1,c65492_g1,c80289_g1,c48660_g1,c65797_g1,c36750_g1,c33146_g1,c72318_g1,c97170_g1,c75725_g1,c71038_g1,c48306_g1,c45742_g1,c27452_g1,c31483_g1,c34275_g1,c20191_g1,c39455_g1,c71068_g1,c45516_g1,c111551_g1,c79323_g1,c40004_g1,c583_g1,c45596_g1,c32310_g2,c44250_g1,c94225_g1,c63359_g1,c42033_g1,c92599_g1,c60570_g1,c40571_g1,c97117_g1,c18166_g1,c31685_g1,c65964_g1,c34190_g1,c100497_g1,c82591_g1,c19371_g1,c103693_g1,c94480_g1,c41123_g1,c72239_g1,c61571_g1,c39157_g2,c64893_g1,c38463_g1,c99954_g1,c8028_g1,c65057_g1,c15936_g1,c44824_g1,c7457_g1,c35619_g1,c50564_g1,c20021_g1,c74700_g1,c47353_g2,c51881_g2,c72783_g1,c43651_g1,c72187_g1,c61688_g1,c109075_g1,c30944_g1,c52600_g3,c71685_g1,c96405_g1,c103460_g1,c54833_g1,c40713_g1,c34581_g1,c46420_g1,c42750_g1,c34017_g1,c87832_g1,c45369_g1,c23873_g1,c1272_g1,c3990_g1,c96576_g1,c63003_g1,c87034_g1,c97697_g1,c52021_g1,c52645_g1,c48219_g2,c50355_g1,c52643_g1,c38276_g1,c96800_g1,c69492_g1,c10943_g1,c102705_g1,c74020_g1,c98920_g1,c24994_g1,c103502_g1,c101079_g1,c52028_g3,c17185_g1,c61213_g1,c63919_g1,c52629_g2,c86663_g1,c53544_g1,c88796_g1,c104810_g1,c38037_g1,c61158_g1,c84260_g1,c52692_g2,c41094_g1,c51824_g1,c85348_g1,c106661_g1,c31976_g1,c103497_g1,c102073_g1,c105722_g1,c46667_g1,c51033_g3,c18935_g1,c54389_g1,c105070_g1,c82173_g1,c51637_g1,c81985_g1,c42264_g1,c36058_g1,c107120_g1,c72951_g1,c98174_g1,c62353_g1,c49092_g1,c48752_g2,c46828_g1,c27625_g1,c108286_g1,c48905_g1,c47664_g1,c16547_g1,c55457_g1,c55121_g1,c84031_g1,c97510_g1,c10753_g1,c18137_g1,c71725_g1,c44293_g1,c57858_g1,c99452_g1,c87049_g1,c65754_g1,c40603_g1,c51490_g1,c82730_g1,c41069_g1,c62342_g1,c34941_g2,c53890_g1,c43732_g2,c101192_g1,c94968_g1,c18260_g1,c88163_g1,c51841_g1,c16380_g1,c44486_g3,c61421_g1,c44055_g1,c46861_g1,c14073_g1,c48203_g1,c99231_g1,c108203_g1,c17193_g1,c21691_g1,c32310_g1,c109754_g1,c13172_g1,c40516_g1,c31234_g1,c57209_g1,c105218_g1,c109031_g1,c102619_g1,c53290_g1,c52210_g2,c89304_g1,c86474_g1,c48203_g2,c83666_g1,c45637_g1,c54342_g1,c78427_g1,c4455_g1,c24327_g1,c28000_g1,c17175_g2,c47736_g1,c1714_g1,c54157_g1,c103683_g1,c77077_g1,c43916_g1,c51975_g6,c34941_g1,c47849_g1,c47289_g2,c97809_g1,c108508_g1,c59050_g1,c55706_g1,c47281_g1,c65908_g1,c65521_g1,c44741_g1,c94639_g1,c50929_g1,c27182_g1,c51313_g2,c105520_g1,c78526_g1,c11457_g1,c81275_g1,c50312_g1,c47122_g3,c87776_g1,c43036_g2,c59855_g1,c51195_g1,c91706_g1,c1279_g1,c72092_g1,c48752_g1,c25385_g1,c47169_g1,c92201_g1,c13411_g1,c56533_g1,c19310_g1,c57174_g1,c8800_g1,c59396_g1,c39870_g1,c110498_g1,c107618_g1,c72628_g1,c65325_g1,c354_g1,c74430_g1,c43611_g1,c15144_g1,c25355_g1,c19301_g1,c85792_g1,c89348_g1,c52135_g1,c82236_g1,c57678_g1,c102593_g1,c34831_g2,c47434_g1,c92769_g1,c53788_g1,c73846_g1,c56624_g1,c73428_g1,c34876_g1,c93118_g1,c102618_g1,c93659_g1,c78160_g1,c47807_g1 |
| Sulfur relay system | ko04122 | 29 | c86131_g1,c93666_g1,c44748_g1,c98178_g1,c34900_g1,c104246_g1,c43749_g2,c87129_g1,c62793_g1,c67976_g1,c50111_g1,c68836_g1,c110026_g1,c109030_g1,c85544_g1,c29589_g1,c95992_g1,c8397_g1,c44052_g1,c50787_g1,c46930_g1,c7773_g1,c48482_g1,c52482_g5,c67819_g1,c73983_g1,c93778_g1,c94705_g1,c60585_g1 |
| Ubiquitin mediated proteolysis | ko04120 | 203 | c65434_g1,c112711_g1,c51810_g2,c82289_g1,c70580_g1,c68581_g1,c44149_g1,c83800_g1,c51588_g2,c79265_g1,c81371_g1,c29237_g1,c105321_g1,c35059_g1,c48410_g1,c82053_g2,c64390_g1,c11969_g1,c42460_g1,c86739_g1,c49295_g1,c47647_g1,c73263_g1,c46213_g2,c45590_g1,c96041_g1,c72904_g1,c67361_g1,c48586_g1,c49866_g1,c38747_g1,c36992_g1,c50331_g1,c98089_g1,c34649_g1,c85196_g1,c104731_g1,c50297_g1,c25388_g1,c106371_g1,c34549_g1,c104832_g1,c72530_g1,c24433_g1,c61369_g1,c48742_g1,c67814_g1,c39926_g1,c104500_g1,c15664_g1,c33763_g3,c28295_g1,c49423_g2,c31109_g2,c51941_g2,c10987_g1,c82345_g1,c78985_g1,c61656_g1,c52266_g1,c42109_g1,c48843_g1,c44491_g1,c48871_g2,c44374_g1,c50278_g1,c86879_g1,c96635_g1,c30287_g1,c51053_g1,c38609_g1,c42174_g3,c9379_g1,c89167_g1,c106447_g1,c30750_g1,c24997_g1,c107724_g1,c45232_g1,c27608_g1,c103545_g1,c34256_g1,c76170_g1,c41676_g1,c53046_g1,c57404_g1,c67054_g1,c9035_g1,c69319_g1,c53622_g1,c99503_g1,c55889_g1,c24816_g1,c26946_g1,c39822_g2,c877_g1,c82203_g1,c84357_g1,c79179_g1,c27942_g1,c92790_g2,c87367_g1,c26912_g1,c31061_g1,c93171_g1,c104068_g1,c54734_g1,c24285_g1,c24624_g1,c45515_g1,c19042_g1,c98969_g1,c107655_g1,c13183_g1,c61993_g1,c46616_g1,c37330_g2,c244_g1,c53875_g1,c95365_g1,c87325_g1,c92607_g1,c51941_g1,c4776_g1,c18261_g2,c19520_g1,c50875_g1,c4631_g1,c62084_g1,c54701_g1,c68783_g1,c103608_g1,c39630_g1,c46373_g1,c96051_g1,c32969_g1,c51824_g1,c72316_g1,c10566_g1,c52005_g5,c47810_g2,c47125_g2,c43859_g1,c26062_g1,c60256_g1,c93119_g1,c3197_g1,c76089_g1,c49394_g1,c53545_g1,c100075_g1,c71912_g1,c93128_g1,c87513_g1,c43322_g1,c109559_g1,c96563_g1,c86401_g1,c89998_g1,c29673_g1,c19485_g1,c27179_g2,c63610_g1,c86414_g1,c50804_g1,c19536_g1,c18368_g1,c93349_g1,c38166_g1,c93615_g1,c52419_g1,c56658_g1,c51032_g1,c54795_g1,c1334_g1,c50617_g1,c28240_g1,c69009_g1,c12727_g1,c47082_g1,c41807_g1,c42461_g1,c96185_g1,c47301_g2,c50504_g1,c33763_g2,c37915_g1,c77461_g1,c50038_g1,c45291_g2,c43531_g1,c105780_g1,c45552_g1,c34348_g1,c46067_g1,c11034_g1,c66838_g1,c56061_g1,c95392_g1,c44104_g1,c14380_g1,c64403_g1,c105182_g1 |
| >3. Environmental Information Processing |  |  |  |
| ABC transporters | ko02010 | 121 | c30967_g1,c51676_g1,c66237_g1,c45117_g1,c36648_g1,c82155_g1,c50441_g1,c84657_g1,c83628_g1,c23574_g1,c108460_g1,c50437_g2,c13454_g1,c200_g2,c78809_g1,c58424_g1,c37057_g1,c83052_g1,c84943_g1,c78378_g1,c50437_g1,c63694_g1,c80570_g1,c5430_g1,c66105_g1,c27421_g1,c99150_g1,c81472_g1,c103721_g1,c45444_g1,c64589_g1,c48539_g1,c107405_g1,c53606_g1,c97706_g1,c94523_g1,c11155_g1,c66949_g1,c62577_g1,c47427_g1,c84185_g1,c71536_g1,c51704_g1,c79542_g1,c82623_g1,c46513_g1,c65387_g1,c52783_g1,c84121_g1,c95683_g1,c83170_g1,c64142_g1,c71516_g1,c50820_g1,c25215_g1,c46967_g1,c45785_g1,c57171_g1,c99619_g1,c109396_g1,c111702_g1,c51124_g1,c46144_g1,c109095_g1,c5719_g2,c108277_g1,c107734_g1,c86881_g1,c62921_g1,c82892_g1,c105420_g1,c58762_g1,c103046_g1,c92812_g1,c30857_g1,c83460_g1,c40527_g1,c44657_g1,c91111_g1,c58739_g1,c36294_g1,c63217_g1,c3692_g1,c95427_g1,c83539_g1,c107454_g1,c1331_g1,c50342_g1,c60935_g1,c63456_g1,c30952_g1,c105731_g1,c54126_g1,c88326_g1,c80963_g1,c110207_g1,c13931_g1,c7563_g1,c63744_g1,c64617_g1,c47001_g1,c85234_g1,c51124_g2,c62420_g1,c12800_g1,c65544_g1,c69734_g1,c51118_g1,c99367_g1,c96792_g1,c64272_g1,c54957_g1,c29338_g1,c55737_g1,c52103_g1,c105054_g1,c25557_g1,c10652_g1,c92839_g1,c59736_g1,c52260_g5 |
| Bacterial secretion system | ko03070 | 26 | c40672_g1,c107314_g1,c44463_g1,c105487_g1,c47905_g1,c60569_g1,c1443_g1,c72742_g1,c45432_g1,c51719_g1,c9986_g1,c39253_g1,c27168_g1,c25794_g1,c99517_g1,c61215_g1,c50792_g1,c70446_g1,c110797_g1,c70131_g1,c82145_g1,c87045_g1,c84006_g1,c107807_g1,c104755_g1,c94120_g1 |
| Calcium signaling pathway | ko04020 | 101 | c76621_g1,c37132_g1,c26595_g1,c27141_g1,c51771_g2,c3181_g1,c46744_g1,c42776_g1,c19074_g1,c80845_g1,c50319_g1,c33484_g1,c5974_g1,c84587_g1,c53776_g1,c12089_g1,c3185_g1,c2113_g1,c46921_g2,c44119_g1,c91611_g1,c17569_g1,c6498_g1,c21357_g1,c7923_g1,c38764_g1,c37692_g1,c16385_g1,c45910_g1,c49876_g1,c61358_g1,c43584_g2,c29860_g1,c46040_g1,c67391_g1,c25760_g1,c84537_g1,c97090_g1,c50987_g1,c28513_g1,c31260_g1,c82692_g1,c73069_g1,c34284_g1,c103279_g1,c41052_g1,c94822_g1,c40326_g1,c67359_g1,c44244_g1,c18232_g1,c45851_g1,c109560_g1,c75246_g1,c18014_g1,c25114_g1,c66315_g1,c56646_g1,c94320_g1,c1091_g1,c13394_g1,c24007_g1,c46921_g1,c86007_g1,c34788_g1,c6980_g1,c43877_g1,c37949_g1,c47867_g1,c65337_g1,c45151_g1,c41047_g1,c31785_g1,c57248_g1,c44669_g1,c47509_g1,c6637_g1,c97532_g1,c63909_g1,c86481_g1,c83293_g1,c92831_g1,c60888_g1,c82782_g1,c94398_g1,c44434_g1,c29145_g1,c18232_g2,c72709_g1,c64069_g1,c91829_g1,c64601_g1,c105274_g1,c44899_g1,c54846_g1,c28657_g2,c51960_g1,c68809_g1,c31195_g1,c48463_g1,c68374_g1 |
| cGMP - PKG signaling pathway | ko04022 | 134 | c76621_g1,c37132_g1,c26595_g1,c27141_g1,c86608_g1,c51771_g2,c3181_g1,c24428_g1,c109525_g1,c46744_g1,c42776_g1,c53983_g1,c80845_g1,c50319_g1,c33484_g1,c5974_g1,c84587_g1,c82683_g1,c53776_g1,c54500_g1,c12089_g1,c3185_g1,c50180_g1,c2113_g1,c75818_g1,c46921_g2,c44119_g1,c91611_g1,c17569_g1,c6498_g1,c21357_g1,c37692_g1,c75097_g1,c16385_g1,c9165_g1,c45910_g1,c61358_g1,c43584_g2,c29860_g1,c28252_g1,c46040_g1,c91916_g1,c67391_g1,c45587_g1,c51940_g1,c25760_g1,c84537_g1,c97090_g1,c31260_g1,c27041_g1,c82692_g1,c50276_g3,c73069_g1,c34284_g1,c49332_g1,c103279_g1,c41052_g1,c25384_g1,c40326_g1,c67359_g1,c44244_g1,c18232_g1,c45851_g1,c109560_g1,c75246_g1,c18014_g1,c25114_g1,c66315_g1,c85582_g1,c97068_g1,c56646_g1,c94320_g1,c29612_g2,c1091_g1,c13394_g1,c24007_g1,c46921_g1,c96785_g1,c49917_g2,c96169_g1,c44412_g1,c86007_g1,c34788_g1,c6980_g1,c43877_g1,c37949_g1,c43821_g1,c47867_g1,c65337_g1,c48330_g1,c65565_g1,c5975_g1,c45151_g1,c41047_g1,c40051_g1,c46805_g1,c103419_g1,c31785_g1,c57248_g1,c44669_g1,c47509_g1,c94013_g1,c84986_g1,c6637_g1,c97532_g1,c63909_g1,c9260_g1,c86481_g1,c83293_g1,c92831_g1,c60888_g1,c59388_g1,c29612_g1,c82782_g1,c94398_g1,c44434_g1,c29145_g1,c18232_g2,c72709_g1,c45509_g1,c64069_g1,c91829_g1,c105274_g1,c49401_g2,c82119_g1,c85931_g1,c54846_g1,c28657_g2,c51960_g1,c68809_g1,c18207_g1,c31195_g1,c48463_g1,c68374_g1 |
| ErbB signaling pathway | ko04012 | 34 | c70915_g1,c31614_g1,c110547_g1,c49070_g1,c81516_g1,c73569_g1,c96884_g1,c96691_g1,c75818_g1,c104688_g1,c75097_g1,c45587_g1,c88836_g1,c42666_g1,c56501_g1,c72535_g1,c61876_g1,c102213_g1,c50859_g1,c41349_g1,c67900_g1,c18161_g1,c74373_g1,c100612_g1,c90282_g1,c65565_g1,c40051_g1,c111499_g1,c76480_g1,c48692_g2,c46989_g1,c67782_g1,c44899_g1,c27176_g1 |
| FoxO signaling pathway | ko04068 | 134 | c61071_g1,c1323_g1,c48547_g1,c57420_g1,c78634_g1,c42570_g1,c803_g1,c107397_g1,c37511_g1,c54766_g1,c111162_g1,c103945_g1,c52836_g1,c70944_g1,c71757_g1,c36164_g1,c93426_g1,c47785_g1,c33552_g1,c2859_g1,c44030_g1,c52736_g1,c84030_g1,c93591_g1,c48377_g1,c74441_g1,c29941_g1,c103771_g1,c82712_g1,c75818_g1,c47524_g2,c27245_g1,c52836_g3,c49550_g1,c30839_g1,c19559_g1,c52628_g2,c35834_g1,c7955_g1,c62599_g1,c75097_g1,c49819_g4,c50953_g1,c38471_g1,c14311_g1,c52316_g2,c40424_g1,c45587_g1,c17647_g1,c35198_g1,c84521_g1,c23121_g1,c26999_g1,c48170_g1,c93738_g1,c53373_g1,c35383_g1,c5131_g1,c40631_g1,c51687_g3,c111847_g1,c111208_g1,c39542_g2,c50458_g2,c72535_g1,c84326_g1,c44565_g1,c44174_g1,c47524_g3,c83302_g1,c38500_g1,c40669_g1,c37894_g1,c62384_g1,c39912_g1,c75676_g1,c14104_g1,c40688_g1,c83740_g1,c39309_g1,c61340_g1,c83486_g1,c43462_g1,c53981_g1,c64363_g1,c52628_g1,c48865_g3,c83387_g1,c65565_g1,c100075_g1,c38467_g1,c16206_g1,c40051_g1,c100039_g1,c52199_g2,c97640_g1,c24876_g1,c73343_g1,c63784_g1,c99976_g1,c54520_g1,c30268_g1,c32760_g1,c51687_g2,c32229_g1,c52316_g3,c46989_g1,c93760_g1,c53926_g1,c65911_g1,c1334_g1,c33613_g1,c42701_g1,c54622_g1,c72040_g1,c99310_g1,c63562_g1,c73286_g1,c41835_g1,c94065_g1,c47524_g1,c111047_g1,c45291_g2,c48724_g1,c110288_g1,c53973_g1,c76613_g1,c16625_g1,c41153_g1,c52310_g2,c28756_g1,c64976_g1,c88514_g1,c53757_g1 |
| Hedgehog signaling pathway | ko04340 | 27 | c31614_g1,c97730_g1,c25317_g1,c71757_g1,c48377_g1,c96691_g1,c47587_g1,c5131_g1,c40631_g1,c39543_g1,c34230_g1,c56501_g1,c48794_g1,c27259_g1,c38776_g3,c102213_g1,c41349_g1,c50269_g3,c18161_g1,c39309_g1,c53981_g1,c32354_g1,c52086_g1,c46561_g1,c86035_g1,c27176_g1,c55367_g1 |
| HIF-1 signaling pathway | ko04066 | 127 | c70915_g1,c1378_g1,c104137_g1,c45645_g1,c38356_g1,c33335_g1,c110547_g1,c29237_g1,c96551_g1,c101256_g1,c6348_g1,c49070_g1,c45979_g1,c81516_g1,c73569_g1,c100050_g1,c25801_g1,c57794_g1,c46404_g4,c38622_g1,c40363_g1,c92790_g1,c48094_g1,c96933_g1,c66648_g1,c46404_g1,c9619_g1,c96884_g1,c75818_g1,c87327_g1,c18147_g1,c106435_g1,c78140_g1,c104688_g1,c27032_g1,c75097_g1,c104310_g1,c86879_g1,c47022_g1,c68919_g1,c76028_g1,c67729_g1,c60359_g1,c45587_g1,c85117_g1,c58517_g1,c82112_g1,c56845_g1,c56971_g1,c88836_g1,c87608_g1,c23285_g1,c69976_g1,c42666_g1,c35383_g1,c82149_g1,c79946_g1,c84114_g1,c26946_g1,c109380_g1,c51687_g3,c46440_g1,c48823_g1,c49207_g1,c66953_g1,c24562_g1,c100584_g1,c61876_g1,c54211_g1,c21314_g1,c50859_g1,c19042_g1,c43644_g1,c67900_g1,c69093_g1,c44819_g1,c74373_g1,c100612_g1,c39352_g1,c90146_g1,c74433_g1,c9043_g1,c33875_g1,c66208_g1,c99539_g1,c18541_g1,c90282_g1,c60256_g1,c65565_g1,c8199_g1,c40051_g1,c50236_g1,c58823_g1,c111499_g1,c85745_g1,c37107_g1,c28291_g1,c26261_g1,c77042_g1,c76480_g1,c46404_g3,c11862_g1,c24600_g1,c48865_g1,c54781_g1,c48692_g2,c110027_g1,c63898_g1,c51687_g2,c30673_g1,c71603_g1,c56658_g1,c49174_g1,c67782_g1,c99310_g1,c62790_g1,c14647_g1,c96465_g1,c44899_g1,c79055_g1,c52381_g3,c52381_g2,c46404_g2,c94570_g1,c89702_g1,c49356_g1,c64403_g1 |
| Hippo signaling pathway | ko04390 | 86 | c68732_g1,c6097_g1,c28372_g1,c31614_g1,c30900_g1,c109525_g1,c92940_g1,c53983_g1,c51984_g1,c71757_g1,c55928_g1,c106501_g1,c14649_g1,c47222_g1,c48377_g1,c54500_g1,c96691_g1,c71535_g1,c47222_g2,c96913_g1,c36334_g1,c28530_g1,c106498_g1,c26107_g1,c973_g1,c53264_g1,c35496_g1,c103876_g1,c65507_g1,c3969_g1,c109664_g1,c33990_g1,c15924_g1,c19545_g1,c35623_g1,c86149_g1,c11164_g2,c5131_g1,c40631_g1,c50276_g3,c25384_g1,c69554_g1,c56501_g1,c60531_g1,c66689_g1,c70407_g1,c26955_g1,c4652_g1,c29612_g2,c4201_g1,c102213_g1,c96785_g1,c49917_g2,c96169_g1,c41349_g1,c39520_g1,c18161_g1,c37003_g1,c16392_g1,c53847_g1,c39309_g1,c87317_g1,c50872_g1,c42615_g1,c94737_g1,c53981_g1,c48330_g1,c46805_g1,c103419_g1,c94013_g1,c103481_g1,c9260_g1,c15250_g1,c59388_g1,c104235_g1,c29612_g1,c33990_g2,c31781_g1,c45509_g1,c47743_g2,c27176_g1,c41755_g1,c56506_g1,c18207_g1,c48456_g1,c98046_g1 |
| Hippo signaling pathway -fly | ko04391 | 70 | c68732_g1,c6097_g1,c20837_g1,c28372_g1,c30900_g1,c92940_g1,c51984_g1,c71757_g1,c45600_g1,c55928_g1,c106501_g1,c14649_g1,c47222_g1,c48377_g1,c71535_g1,c47222_g2,c96913_g1,c36334_g1,c28530_g1,c106498_g1,c26107_g1,c973_g1,c53264_g1,c35496_g1,c103876_g1,c40182_g1,c65507_g1,c3969_g1,c109664_g1,c33990_g1,c15924_g1,c106527_g1,c19545_g1,c35623_g1,c86149_g1,c11164_g2,c5131_g1,c40631_g1,c69554_g1,c60531_g1,c66689_g1,c70407_g1,c26955_g1,c4652_g1,c47791_g2,c4201_g1,c39520_g1,c37003_g1,c16392_g1,c14594_g1,c53847_g1,c39309_g1,c87317_g1,c50872_g1,c42615_g1,c94737_g1,c53981_g1,c19397_g1,c47791_g1,c103481_g1,c15250_g1,c104235_g1,c33990_g2,c31781_g1,c39909_g1,c47743_g2,c41755_g1,c56506_g1,c48456_g1,c98046_g1 |
| Jak-STAT signaling pathway | ko04630 | 5 | c35383_g1,c51687_g3,c49394_g1,c51687_g2,c99310_g1 |
| MAPK signaling pathway | ko04010 | 138 | c48560_g1,c26595_g1,c91578_g1,c51771_g2,c81465_g1,c4329_g1,c82152_g1,c87471_g1,c68830_g1,c40971_g1,c65806_g1,c48247_g2,c46828_g2,c95765_g1,c85476_g1,c54485_g1,c51603_g3,c5979_g1,c84871_g1,c99424_g1,c90199_g1,c96066_g1,c37866_g2,c63084_g1,c75818_g1,c46921_g2,c61171_g1,c46828_g3,c60132_g1,c30224_g1,c88688_g1,c47248_g1,c76196_g1,c97805_g1,c73322_g1,c62391_g1,c75604_g1,c93546_g1,c104689_g1,c19117_g1,c75097_g1,c27003_g1,c98426_g1,c43036_g1,c75980_g1,c41202_g1,c45587_g1,c71422_g1,c36117_g1,c7076_g1,c59458_g1,c93365_g1,c55924_g1,c27920_g1,c97101_g1,c99321_g1,c71662_g1,c82692_g1,c65797_g1,c46031_g1,c48306_g1,c45851_g1,c20191_g1,c30868_g1,c72535_g1,c32310_g2,c42033_g1,c92599_g1,c77336_g1,c39157_g2,c65057_g1,c7457_g1,c51603_g1,c13394_g1,c38253_g1,c46921_g1,c103460_g1,c54833_g1,c34581_g1,c50176_g1,c23873_g1,c72326_g1,c96576_g1,c63003_g1,c10943_g1,c86007_g1,c63919_g1,c88796_g1,c37689_g1,c104810_g1,c43877_g1,c103497_g1,c102073_g1,c18935_g1,c105070_g1,c36058_g1,c47867_g1,c62353_g1,c46828_g1,c65565_g1,c34368_g1,c40051_g1,c87049_g1,c54073_g1,c65754_g1,c62342_g1,c107614_g1,c34941_g2,c99231_g1,c17193_g1,c40704_g2,c32310_g1,c13172_g1,c40516_g1,c37697_g1,c109031_g1,c78655_g1,c4455_g1,c94398_g1,c95446_g1,c83855_g1,c46989_g1,c34941_g1,c72709_g1,c55706_g1,c43036_g2,c28281_g1,c13411_g1,c56533_g1,c57174_g1,c103367_g1,c110498_g1,c107618_g1,c74430_g1,c19301_g1,c26895_g1,c102593_g1,c37646_g1 |
| MAPK signaling pathway - fly | ko04013 | 5 | c75818_g1,c75097_g1,c45587_g1,c65565_g1,c40051_g1 |
| MAPK signaling pathway - yeast | ko04011 | 9 | c73707_g1,c30759_g1,c43484_g1,c86727_g1,c106025_g1,c4238_g1,c49156_g2,c38282_g1,c27469_g1 |
| mTOR signaling pathway | ko04150 | 81 | c70915_g1,c61071_g1,c42570_g1,c47050_g1,c110547_g1,c35418_g1,c94952_g1,c49070_g1,c81516_g1,c49016_g1,c73569_g1,c25801_g1,c38622_g1,c40363_g1,c9619_g1,c74441_g1,c76639_g1,c96884_g1,c103771_g1,c11342_g1,c47524_g2,c87327_g1,c49550_g1,c7955_g1,c104688_g1,c27032_g1,c75097_g1,c50953_g1,c38471_g1,c17647_g1,c58517_g1,c88836_g1,c93738_g1,c53373_g1,c42666_g1,c46440_g1,c72535_g1,c44565_g1,c6288_g1,c63176_g1,c44174_g1,c61876_g1,c47524_g3,c40669_g1,c53653_g1,c50859_g1,c39912_g1,c67900_g1,c44819_g1,c74373_g1,c100612_g1,c49357_g2,c83486_g1,c90282_g1,c64363_g1,c8199_g1,c40051_g1,c97640_g1,c111499_g1,c39681_g1,c37107_g1,c26261_g1,c99976_g1,c76480_g1,c11862_g1,c54781_g1,c48692_g2,c30268_g1,c32760_g1,c32229_g1,c46989_g1,c65911_g1,c108416_g1,c67782_g1,c62790_g1,c47524_g1,c48724_g1,c41153_g1,c28721_g1,c64976_g1,c53757_g1 |
| Neuroactive ligand-receptor interaction | ko04080 | 11 | c56768_g1,c110467_g1,c52722_g1,c92864_g1,c70986_g1,c40081_g1,c71964_g1,c106298_g1,c93427_g1,c71735_g1,c91830_g1 |
| NF-kappa B signaling pathway | ko04064 | 311 | c28479_g1,c104104_g1,c82662_g1,c8503_g1,c35897_g1,c42058_g1,c42455_g1,c20428_g1,c31551_g1,c31284_g1,c48654_g1,c63236_g1,c49133_g1,c90349_g1,c52047_g3,c75900_g1,c19656_g1,c67256_g1,c85350_g1,c64798_g1,c36093_g1,c9338_g1,c50509_g1,c51362_g1,c46431_g1,c68166_g1,c51393_g2,c109082_g1,c41136_g1,c50433_g1,c52836_g1,c76045_g1,c41452_g1,c77468_g1,c48850_g1,c68992_g1,c99939_g1,c67306_g1,c20958_g1,c59703_g1,c70554_g1,c25682_g1,c51493_g1,c98724_g1,c28106_g1,c50497_g1,c87121_g1,c30825_g1,c4653_g1,c55823_g1,c24811_g1,c44772_g1,c29500_g1,c59145_g1,c51579_g1,c21101_g1,c43041_g1,c19257_g1,c52736_g1,c93320_g1,c72649_g1,c29983_g2,c43961_g1,c52309_g1,c48726_g1,c51393_g1,c73826_g1,c20517_g1,c106847_g1,c20072_g1,c37055_g1,c77647_g1,c52385_g2,c104832_g1,c49940_g3,c43215_g1,c99845_g1,c15664_g1,c89573_g1,c16333_g1,c103884_g1,c64857_g1,c24563_g1,c52836_g3,c52687_g3,c27364_g1,c49555_g1,c97243_g1,c60036_g1,c44871_g1,c28878_g1,c43552_g1,c42662_g1,c111102_g1,c12545_g1,c19670_g1,c46019_g1,c99189_g1,c69108_g1,c50706_g2,c49045_g1,c106613_g1,c51250_g1,c85938_g1,c48076_g1,c85515_g1,c50542_g1,c106447_g1,c27417_g1,c105342_g1,c46894_g1,c74335_g1,c30663_g1,c5967_g1,c89522_g1,c12183_g1,c27559_g1,c45585_g1,c31723_g1,c50354_g1,c36550_g1,c51087_g1,c52810_g1,c44213_g2,c63994_g1,c102241_g1,c56011_g1,c52221_g2,c35128_g1,c25032_g1,c31283_g1,c41811_g1,c93658_g1,c38149_g1,c63402_g1,c74219_g1,c31287_g1,c73710_g1,c49457_g4,c33637_g1,c25651_g1,c28865_g1,c2927_g1,c79179_g1,c51933_g1,c45078_g1,c11780_g1,c50217_g1,c43622_g1,c52977_g1,c48112_g2,c99332_g1,c35585_g2,c37345_g1,c39201_g1,c48245_g1,c52206_g3,c38021_g1,c34104_g1,c36833_g1,c26902_g1,c49999_g4,c52084_g5,c45277_g1,c48988_g3,c11267_g1,c48713_g2,c47048_g2,c46096_g1,c40803_g1,c38428_g2,c108116_g1,c35585_g1,c97045_g1,c25369_g1,c13250_g1,c44805_g1,c52646_g1,c42711_g1,c16958_g1,c40688_g1,c65482_g1,c77087_g1,c45556_g1,c84103_g1,c55120_g1,c51243_g7,c48972_g1,c20000_g1,c48288_g1,c61555_g1,c57458_g1,c58309_g1,c45137_g1,c38091_g2,c41208_g1,c77837_g1,c106258_g1,c35140_g1,c20246_g1,c39725_g1,c25026_g1,c51314_g5,c48833_g1,c106429_g1,c34931_g1,c35002_g1,c40640_g1,c77655_g1,c39243_g1,c51009_g1,c23872_g1,c46394_g2,c26562_g1,c97184_g1,c12015_g1,c45152_g1,c17204_g1,c1970_g1,c95560_g1,c109295_g1,c48337_g1,c45705_g1,c40297_g1,c50480_g1,c71256_g1,c17979_g1,c17204_g2,c79424_g1,c43106_g1,c39874_g1,c16587_g1,c45705_g2,c52199_g2,c29193_g1,c75439_g1,c71808_g1,c48425_g1,c41500_g1,c38710_g1,c96255_g1,c53988_g1,c44980_g1,c49276_g4,c92119_g1,c62976_g1,c46059_g2,c47535_g2,c52687_g2,c63601_g1,c32428_g1,c63610_g1,c74294_g1,c63478_g1,c2959_g1,c36767_g1,c56627_g1,c110032_g1,c51379_g1,c31527_g1,c52047_g1,c84912_g1,c14738_g1,c48723_g1,c50422_g6,c70565_g1,c85278_g1,c27075_g1,c50171_g1,c5896_g1,c88625_g1,c41879_g1,c72114_g1,c12186_g1,c33668_g1,c44897_g1,c22716_g1,c15748_g1,c32004_g1,c27875_g1,c108348_g1,c61603_g1,c34370_g1,c107634_g1,c52773_g1,c29143_g1,c37602_g1,c48569_g1,c43531_g1,c5603_g1,c52449_g2,c52687_g4,c54042_g1,c41813_g1,c10126_g1,c62984_g1,c49158_g1,c44871_g2,c95993_g1,c47048_g1,c41963_g1,c31673_g1,c38805_g1,c48713_g1,c34410_g1,c43448_g1,c33820_g1,c59828_g1,c45364_g1,c607_g1,c86378_g1 |
| Notch signaling pathway | ko04330 | 22 | c52413_g4,c19284_g1,c24966_g1,c73214_g1,c68322_g1,c108494_g1,c35383_g1,c51687_g3,c52241_g8,c44250_g1,c63359_g1,c31251_g1,c65050_g1,c58019_g1,c52413_g3,c11520_g1,c45550_g1,c40087_g2,c51687_g2,c94639_g1,c99310_g1,c4402_g1 |
| Phosphatidylinositol signaling system | ko04070 | 87 | c76621_g1,c106784_g1,c3181_g1,c78834_g1,c19074_g1,c61108_g1,c80845_g1,c50319_g1,c33484_g1,c5974_g1,c79588_g1,c53776_g1,c84609_g1,c41491_g1,c570_g1,c3185_g1,c59147_g1,c45576_g1,c108570_g1,c7923_g1,c38764_g1,c25403_g1,c103543_g1,c47840_g1,c51930_g1,c37692_g1,c45182_g1,c38471_g1,c43584_g2,c94906_g1,c29860_g1,c67391_g1,c17647_g1,c16404_g1,c25760_g1,c84537_g1,c97090_g1,c50987_g1,c28513_g1,c31260_g1,c48454_g1,c34284_g1,c103279_g1,c41052_g1,c44244_g1,c13281_g1,c47405_g1,c46990_g2,c50324_g1,c55469_g1,c24007_g1,c40171_g1,c91460_g1,c34788_g1,c28439_g1,c6980_g1,c49406_g3,c32939_g1,c65337_g1,c64363_g1,c3572_g1,c41047_g1,c62506_g1,c31785_g1,c6637_g1,c63909_g1,c86481_g1,c92831_g1,c52549_g2,c82782_g1,c50791_g1,c44060_g1,c64601_g1,c49205_g1,c76881_g1,c48724_g1,c52092_g1,c28657_g2,c52617_g1,c29296_g1,c43923_g1,c68809_g1,c49406_g2,c22860_g1,c31195_g1,c48463_g1,c64976_g1 |
| PI3K-Akt signaling pathway | ko04151 | 252 | c70915_g1,c61071_g1,c68732_g1,c24927_g1,c42570_g1,c6097_g1,c803_g1,c28372_g1,c8275_g1,c31614_g1,c110547_g1,c46305_g1,c30900_g1,c4329_g1,c35418_g1,c47513_g1,c92940_g1,c64341_g1,c111162_g1,c60514_g1,c51984_g1,c49070_g1,c70944_g1,c81516_g1,c73569_g1,c25801_g1,c27434_g1,c38622_g1,c30744_g1,c55928_g1,c106501_g1,c40363_g1,c68079_g1,c76236_g1,c63438_g1,c48247_g2,c21454_g1,c14649_g1,c9619_g1,c85476_g1,c54485_g1,c84030_g1,c35896_g1,c47222_g1,c74441_g1,c18431_g1,c84183_g1,c76639_g1,c96884_g1,c89738_g1,c96691_g1,c71535_g1,c47222_g2,c96913_g1,c21694_g1,c85426_g1,c36334_g1,c103771_g1,c75818_g1,c28530_g1,c59352_g1,c47524_g2,c106498_g1,c61171_g1,c96128_g1,c87327_g1,c49550_g1,c26107_g1,c13933_g1,c93546_g1,c973_g1,c50398_g3,c7955_g1,c48114_g1,c104688_g1,c53264_g1,c62599_g1,c27032_g1,c45475_g1,c82844_g1,c9107_g1,c104764_g1,c35496_g1,c75097_g1,c11484_g1,c50953_g1,c38471_g1,c30486_g2,c14311_g1,c103876_g1,c1106_g1,c33253_g1,c30486_g3,c65507_g1,c49828_g1,c40424_g1,c3969_g1,c97112_g1,c7804_g1,c109664_g1,c33990_g1,c15924_g1,c99632_g1,c50398_g4,c45587_g1,c17647_g1,c59360_g1,c703_g1,c58517_g1,c18330_g1,c7076_g1,c43298_g1,c19545_g1,c93365_g1,c55924_g1,c27920_g1,c88836_g1,c110650_g1,c86149_g1,c26129_g1,c56887_g1,c93738_g1,c53373_g1,c101207_g1,c42666_g1,c83184_g1,c71662_g1,c69554_g1,c111711_g1,c56501_g1,c46440_g1,c50398_g2,c8448_g1,c60531_g1,c30868_g1,c84326_g1,c44565_g1,c66689_g1,c82688_g1,c6288_g1,c77336_g1,c63116_g1,c44174_g1,c70407_g1,c30486_g1,c26955_g1,c31830_g1,c70327_g1,c61876_g1,c4652_g1,c47524_g3,c77560_g1,c40669_g1,c4201_g1,c102213_g1,c86026_g1,c38253_g1,c50859_g1,c56408_g1,c39912_g1,c41349_g1,c39520_g1,c72326_g1,c67900_g1,c105077_g1,c44819_g1,c75676_g1,c18161_g1,c37003_g1,c54723_g1,c16392_g1,c30518_g1,c74373_g1,c100612_g1,c37689_g1,c53847_g1,c54926_g1,c79194_g1,c83486_g1,c87317_g1,c42615_g1,c94737_g1,c90282_g1,c64363_g1,c65565_g1,c8199_g1,c34368_g1,c40051_g1,c25469_g1,c103481_g1,c97640_g1,c94352_g1,c97219_g1,c97340_g1,c111499_g1,c37107_g1,c15250_g1,c26261_g1,c99976_g1,c76480_g1,c57085_g1,c11862_g1,c111069_g1,c54781_g1,c48692_g2,c82349_g1,c37697_g1,c30268_g1,c32760_g1,c39689_g1,c104235_g1,c32229_g1,c105795_g1,c78655_g1,c33990_g2,c31781_g1,c95446_g1,c83855_g1,c50576_g1,c65911_g1,c108416_g1,c86000_g1,c72040_g1,c67782_g1,c63562_g1,c96864_g1,c73286_g1,c62790_g1,c49610_g1,c94065_g1,c62057_g1,c47524_g1,c28281_g1,c47743_g2,c48724_g1,c15902_g1,c112008_g1,c27176_g1,c103367_g1,c42680_g1,c56506_g1,c10282_g1,c41153_g1,c109172_g1,c26895_g1,c28721_g1,c48456_g1,c98046_g1,c64976_g1,c51114_g1,c88514_g1,c53757_g1 |
| Plant hormone signal transduction | ko04075 | 253 | c10658_g1,c38842_g1,c35028_g1,c29239_g1,c108273_g1,c43866_g1,c29153_g1,c40226_g1,c47440_g1,c85536_g1,c40267_g1,c48355_g2,c37248_g1,c104199_g2,c51592_g4,c44552_g1,c26859_g1,c33720_g1,c43868_g1,c52121_g3,c47020_g2,c1916_g1,c47557_g1,c94309_g1,c51857_g1,c48965_g1,c92827_g1,c41182_g1,c4178_g1,c4229_g1,c28684_g1,c37470_g1,c37492_g1,c13000_g1,c39924_g2,c5161_g1,c39011_g1,c48821_g2,c9295_g1,c50366_g1,c96049_g1,c82680_g1,c48422_g1,c16613_g1,c18472_g1,c17138_g1,c44279_g3,c11768_g1,c84794_g1,c38991_g1,c28620_g1,c49890_g3,c107221_g1,c43605_g2,c9183_g1,c40723_g1,c73376_g1,c63731_g1,c42065_g1,c35582_g1,c17243_g1,c27208_g1,c30144_g1,c33521_g1,c28670_g1,c52895_g1,c42171_g1,c45162_g1,c97683_g1,c34645_g1,c48614_g1,c27153_g1,c111140_g1,c41223_g1,c46214_g2,c103375_g1,c42882_g1,c50695_g2,c47608_g2,c9245_g1,c93763_g1,c16925_g1,c36082_g1,c44975_g1,c41105_g1,c25363_g1,c29227_g1,c40123_g1,c15221_g1,c47571_g2,c40879_g1,c36096_g1,c84948_g1,c45232_g2,c43284_g1,c41830_g1,c48743_g1,c8696_g1,c34858_g1,c2677_g1,c47847_g1,c95529_g1,c42843_g1,c19076_g1,c100094_g1,c1964_g1,c48162_g1,c17905_g1,c30850_g1,c103861_g1,c58629_g1,c63144_g1,c32217_g1,c32193_g1,c34055_g1,c41470_g1,c36359_g1,c107414_g1,c83206_g1,c41424_g1,c73394_g1,c31312_g1,c40492_g1,c35349_g1,c48072_g1,c49099_g1,c44603_g1,c41080_g1,c50240_g1,c39242_g1,c44264_g1,c39193_g1,c49519_g1,c29865_g1,c12918_g1,c62807_g1,c93011_g1,c18180_g1,c47097_g1,c13209_g1,c20345_g1,c85770_g1,c37546_g1,c28613_g1,c43547_g1,c46814_g1,c31308_g1,c37418_g1,c37173_g1,c53050_g1,c61969_g1,c48518_g1,c51652_g3,c33978_g1,c45493_g1,c96927_g1,c41529_g1,c26859_g2,c25416_g1,c10696_g1,c42795_g1,c43719_g1,c45823_g1,c81319_g1,c37822_g2,c37377_g1,c83528_g1,c47020_g1,c50095_g2,c82725_g1,c50824_g1,c43112_g1,c48151_g1,c50438_g1,c45434_g1,c40526_g1,c45028_g1,c62907_g1,c39924_g3,c50080_g2,c65342_g1,c43945_g1,c43258_g1,c59392_g1,c19913_g1,c51652_g4,c23068_g1,c35018_g1,c52121_g2,c37460_g1,c37765_g1,c26939_g1,c30751_g1,c50193_g1,c44765_g1,c40280_g1,c17200_g1,c40790_g1,c45778_g1,c27009_g1,c9570_g1,c24577_g1,c52590_g2,c75429_g1,c44906_g1,c45043_g1,c37244_g1,c83546_g1,c50508_g2,c44623_g1,c15703_g1,c38253_g2,c33865_g1,c35981_g1,c29073_g1,c18410_g1,c103984_g1,c59251_g1,c44484_g1,c5578_g1,c35018_g2,c71674_g1,c4670_g1,c31591_g1,c31279_g1,c9130_g1,c46065_g2,c48167_g2,c50264_g1,c20010_g1,c94499_g1,c24093_g1,c29852_g1,c103696_g1,c9219_g1,c39927_g1,c44064_g1,c48848_g1,c35296_g1,c44537_g1,c36773_g1,c30581_g1,c28241_g1,c3153_g1,c27512_g1,c3947_g1,c48372_g1,c209_g1,c43337_g1,c41620_g1,c49159_g1,c48355_g1,c103921_g1 |
| Rap1 signaling pathway | ko04015 | 72 | c76621_g1,c3181_g1,c4329_g1,c80845_g1,c50319_g1,c33484_g1,c48247_g2,c5974_g1,c85476_g1,c54485_g1,c53776_g1,c3185_g1,c75818_g1,c61171_g1,c93546_g1,c37692_g1,c75097_g1,c43584_g2,c29860_g1,c67391_g1,c45587_g1,c7076_g1,c93365_g1,c55924_g1,c27920_g1,c25760_g1,c84537_g1,c97090_g1,c31260_g1,c71662_g1,c34284_g1,c103279_g1,c41052_g1,c44244_g1,c30868_g1,c72535_g1,c77336_g1,c24007_g1,c38253_g1,c72326_g1,c34788_g1,c37689_g1,c28799_g1,c6980_g1,c65337_g1,c65565_g1,c41047_g1,c34368_g1,c40051_g1,c31785_g1,c6637_g1,c63909_g1,c86481_g1,c92831_g1,c37697_g1,c26247_g1,c82782_g1,c78655_g1,c25443_g1,c95446_g1,c83855_g1,c46989_g1,c28281_g1,c103367_g1,c105845_g1,c28605_g1,c28657_g2,c68809_g1,c26895_g1,c105414_g1,c31195_g1,c48463_g1 |
| Ras signaling pathway | ko04014 | 98 | c76621_g1,c24927_g1,c51039_g2,c94994_g1,c3181_g1,c4329_g1,c44450_g1,c80845_g1,c50203_g2,c50319_g1,c29797_g1,c33484_g1,c63785_g1,c48247_g2,c5974_g1,c1591_g1,c85476_g1,c54485_g1,c41055_g1,c53776_g1,c84183_g1,c3185_g1,c75818_g1,c61171_g1,c93546_g1,c37692_g1,c75097_g1,c11484_g1,c46213_g3,c52888_g1,c43584_g2,c97112_g1,c29860_g1,c99632_g1,c67391_g1,c45587_g1,c7076_g1,c93365_g1,c55924_g1,c27920_g1,c25760_g1,c84537_g1,c97090_g1,c34843_g1,c31260_g1,c71662_g1,c34284_g1,c28119_g1,c103279_g1,c41052_g1,c44244_g1,c50203_g1,c26986_g1,c30868_g1,c77336_g1,c63116_g1,c24007_g1,c38253_g1,c72326_g1,c105077_g1,c60324_g1,c34788_g1,c37689_g1,c6980_g1,c3957_g1,c30134_g1,c65337_g1,c65565_g1,c48602_g1,c41047_g1,c34368_g1,c40051_g1,c31785_g1,c24808_g1,c6637_g1,c63909_g1,c86481_g1,c92831_g1,c30837_g1,c37697_g1,c82782_g1,c78655_g1,c95446_g1,c83855_g1,c4502_g1,c18447_g1,c44745_g1,c28281_g1,c103367_g1,c10282_g1,c28657_g2,c68809_g1,c26895_g1,c51463_g1,c74487_g1,c31195_g1,c51512_g4,c48463_g1 |
| TGF-beta signaling pathway | ko04350 | 62 | c70915_g1,c68732_g1,c6097_g1,c110547_g1,c81371_g1,c29237_g1,c30900_g1,c92940_g1,c73569_g1,c14649_g1,c96884_g1,c25388_g1,c71535_g1,c96913_g1,c72530_g1,c26107_g1,c104688_g1,c44374_g1,c35496_g1,c75097_g1,c86879_g1,c103876_g1,c35127_g1,c15924_g1,c42368_g1,c88836_g1,c42666_g1,c35383_g1,c24816_g1,c51687_g3,c26912_g1,c66689_g1,c70407_g1,c61876_g1,c50859_g1,c67900_g1,c50875_g1,c4631_g1,c16392_g1,c74373_g1,c100612_g1,c53847_g1,c47810_g2,c42615_g1,c90282_g1,c48999_g2,c60256_g1,c40051_g1,c103481_g1,c111499_g1,c27179_g2,c76480_g1,c48692_g2,c51687_g2,c56658_g1,c67782_g1,c99310_g1,c47743_g2,c56506_g1,c48456_g1,c95392_g1,c64403_g1 |
| TNF signaling pathway | ko04668 | 12 | c30442_g1,c87546_g1,c75818_g1,c73663_g1,c75097_g1,c45587_g1,c67793_g1,c65565_g1,c40051_g1,c49037_g1,c97586_g1,c49586_g1 |
| Two-component system | ko02020 | 61 | c34735_g1,c60032_g1,c48902_g1,c108958_g1,c48753_g1,c59098_g1,c53271_g1,c92603_g1,c105449_g1,c71720_g1,c95320_g1,c53271_g2,c61157_g1,c77190_g1,c24765_g1,c80976_g1,c102809_g1,c44181_g1,c112258_g1,c6075_g1,c75123_g1,c43714_g1,c85429_g1,c73295_g1,c71045_g1,c31587_g1,c64933_g1,c61622_g1,c107114_g1,c64671_g1,c12872_g1,c103615_g1,c95064_g1,c55062_g1,c110399_g1,c102383_g1,c10633_g1,c45816_g1,c71718_g1,c71787_g1,c3591_g1,c16834_g1,c55559_g1,c74972_g1,c32556_g1,c95676_g1,c11160_g1,c34225_g1,c37822_g1,c52015_g1,c72872_g1,c9150_g1,c1164_g1,c93711_g1,c73323_g1,c106734_g1,c57606_g1,c61668_g1,c100009_g1,c31759_g1,c96545_g1 |
| VEGF signaling pathway | ko04370 | 43 | c26595_g1,c51771_g2,c4329_g1,c48247_g2,c85476_g1,c54485_g1,c75818_g1,c46921_g2,c61171_g1,c93546_g1,c75097_g1,c49876_g1,c45587_g1,c7076_g1,c93365_g1,c55924_g1,c27920_g1,c71662_g1,c82692_g1,c94822_g1,c45851_g1,c30868_g1,c77336_g1,c13394_g1,c38253_g1,c46921_g1,c72326_g1,c86007_g1,c37689_g1,c43877_g1,c47867_g1,c65565_g1,c34368_g1,c40051_g1,c37697_g1,c78655_g1,c94398_g1,c95446_g1,c83855_g1,c72709_g1,c28281_g1,c103367_g1,c26895_g1 |
| Wnt signaling pathway | ko04310 | 98 | c26595_g1,c31614_g1,c44149_g1,c81371_g1,c29237_g1,c51771_g2,c75900_g1,c4329_g1,c71757_g1,c98724_g1,c48247_g2,c49866_g1,c85476_g1,c54485_g1,c48377_g1,c25388_g1,c24966_g1,c96691_g1,c72530_g1,c46921_g2,c86786_g1,c61171_g1,c31109_g2,c93546_g1,c111102_g1,c68322_g1,c44374_g1,c86879_g1,c38609_g1,c7076_g1,c93365_g1,c55924_g1,c27920_g1,c45715_g1,c35383_g1,c5131_g1,c40631_g1,c24816_g1,c103888_g1,c71662_g1,c82692_g1,c28865_g1,c51687_g3,c56501_g1,c26912_g1,c45851_g1,c30868_g1,c52977_g1,c77336_g1,c106599_g1,c102213_g1,c13394_g1,c38253_g1,c46921_g1,c46616_g1,c41349_g1,c72326_g1,c50875_g1,c4631_g1,c18161_g1,c86007_g1,c11520_g1,c37689_g1,c43877_g1,c39309_g1,c47810_g2,c47867_g1,c53981_g1,c26062_g1,c60256_g1,c34368_g1,c45550_g1,c62976_g1,c27179_g2,c56627_g1,c37697_g1,c44532_g1,c51687_g2,c43368_g1,c78655_g1,c94398_g1,c56658_g1,c95446_g1,c83855_g1,c72709_g1,c99310_g1,c28281_g1,c44899_g1,c27176_g1,c103367_g1,c41813_g1,c95993_g1,c26895_g1,c11034_g1,c43448_g1,c72048_g1,c95392_g1,c64403_g1 |
| >4. Cellular Processes |  |  |  |
| Adherens junction | ko04520 | 42 | c75900_g1,c4329_g1,c98724_g1,c48247_g2,c85476_g1,c54485_g1,c29118_g1,c61171_g1,c93546_g1,c111102_g1,c75097_g1,c7076_g1,c93365_g1,c55924_g1,c27920_g1,c35383_g1,c71662_g1,c28865_g1,c51687_g3,c30868_g1,c52977_g1,c77336_g1,c38253_g1,c72326_g1,c37689_g1,c34368_g1,c40051_g1,c62976_g1,c56627_g1,c37697_g1,c51687_g2,c78655_g1,c95446_g1,c83855_g1,c99310_g1,c45108_g2,c28281_g1,c103367_g1,c41813_g1,c95993_g1,c26895_g1,c43448_g1 |
| Apoptosis | ko04210 | 318 | c28479_g1,c104104_g1,c82662_g1,c8503_g1,c35897_g1,c42058_g1,c42455_g1,c26595_g1,c20428_g1,c31551_g1,c31284_g1,c48654_g1,c63236_g1,c49133_g1,c90349_g1,c52047_g3,c51771_g2,c19656_g1,c67256_g1,c85350_g1,c64798_g1,c36093_g1,c9338_g1,c50509_g1,c51362_g1,c46431_g1,c68166_g1,c51393_g2,c109082_g1,c41136_g1,c50433_g1,c52836_g1,c76045_g1,c41452_g1,c77468_g1,c48850_g1,c68992_g1,c99939_g1,c67306_g1,c20958_g1,c59703_g1,c108958_g1,c70554_g1,c25682_g1,c51493_g1,c28106_g1,c50497_g1,c87121_g1,c30825_g1,c4653_g1,c55823_g1,c24811_g1,c44772_g1,c29500_g1,c59145_g1,c51579_g1,c21101_g1,c43041_g1,c19257_g1,c52736_g1,c93320_g1,c72649_g1,c105449_g1,c29983_g2,c43961_g1,c52309_g1,c48726_g1,c51393_g1,c73826_g1,c20517_g1,c106847_g1,c20072_g1,c37055_g1,c77647_g1,c52385_g2,c49940_g3,c43215_g1,c99845_g1,c46921_g2,c89573_g1,c16333_g1,c103884_g1,c64857_g1,c24563_g1,c52836_g3,c52687_g3,c27364_g1,c49555_g1,c97243_g1,c60036_g1,c44871_g1,c28878_g1,c43552_g1,c42662_g1,c12545_g1,c19670_g1,c46019_g1,c99189_g1,c69108_g1,c50706_g2,c49045_g1,c106613_g1,c51250_g1,c85938_g1,c48076_g1,c85515_g1,c50542_g1,c27417_g1,c105342_g1,c46894_g1,c74335_g1,c30663_g1,c5967_g1,c89522_g1,c12183_g1,c27559_g1,c45585_g1,c31723_g1,c50354_g1,c36550_g1,c51087_g1,c52810_g1,c44213_g2,c63994_g1,c6075_g1,c102241_g1,c56011_g1,c52221_g2,c35128_g1,c25032_g1,c31283_g1,c41811_g1,c93658_g1,c38149_g1,c63402_g1,c74219_g1,c31287_g1,c73710_g1,c82692_g1,c49457_g4,c33637_g1,c25651_g1,c2927_g1,c45851_g1,c51933_g1,c45078_g1,c11780_g1,c50217_g1,c71045_g1,c43622_g1,c31587_g1,c48112_g2,c99332_g1,c35585_g2,c37345_g1,c39201_g1,c48245_g1,c52206_g3,c38021_g1,c34104_g1,c36833_g1,c26902_g1,c49999_g4,c52084_g5,c45277_g1,c48988_g3,c13394_g1,c11267_g1,c48713_g2,c47048_g2,c46921_g1,c46096_g1,c40803_g1,c38428_g2,c108116_g1,c35585_g1,c97045_g1,c25369_g1,c13250_g1,c44805_g1,c52646_g1,c42711_g1,c16958_g1,c40688_g1,c86007_g1,c65482_g1,c77087_g1,c45556_g1,c84103_g1,c55120_g1,c51243_g7,c48972_g1,c20000_g1,c48288_g1,c61555_g1,c57458_g1,c58309_g1,c45137_g1,c38091_g2,c41208_g1,c77837_g1,c43877_g1,c106258_g1,c35140_g1,c20246_g1,c39725_g1,c25026_g1,c51314_g5,c48833_g1,c106429_g1,c34931_g1,c35002_g1,c40640_g1,c77655_g1,c39243_g1,c51009_g1,c47867_g1,c23872_g1,c46394_g2,c26562_g1,c97184_g1,c12015_g1,c45152_g1,c17204_g1,c1970_g1,c95560_g1,c109295_g1,c48337_g1,c45705_g1,c40297_g1,c50480_g1,c71256_g1,c17979_g1,c17204_g2,c79424_g1,c43106_g1,c39874_g1,c16587_g1,c45705_g2,c92791_g1,c52199_g2,c29193_g1,c75439_g1,c71808_g1,c48425_g1,c41500_g1,c38710_g1,c16834_g1,c96255_g1,c53988_g1,c44980_g1,c49276_g4,c92119_g1,c55559_g1,c46059_g2,c47535_g2,c52687_g2,c63601_g1,c32428_g1,c74294_g1,c63478_g1,c2959_g1,c36767_g1,c110032_g1,c51379_g1,c31527_g1,c52047_g1,c84912_g1,c11160_g1,c14738_g1,c94398_g1,c48723_g1,c50422_g6,c70565_g1,c85278_g1,c27075_g1,c72709_g1,c50171_g1,c5896_g1,c88625_g1,c41879_g1,c72114_g1,c12186_g1,c33668_g1,c44897_g1,c22716_g1,c15748_g1,c32004_g1,c27875_g1,c108348_g1,c61603_g1,c34370_g1,c107634_g1,c52773_g1,c29143_g1,c100803_g1,c37602_g1,c48569_g1,c5603_g1,c52449_g2,c52687_g4,c54042_g1,c10126_g1,c62984_g1,c49158_g1,c44871_g2,c47048_g1,c41963_g1,c31673_g1,c38805_g1,c48713_g1,c34410_g1,c33820_g1,c59828_g1,c45364_g1,c607_g1,c86378_g1,c96545_g1 |
| Cell cycle | ko04110 | 187 | c48547_g1,c47667_g1,c36207_g1,c112711_g1,c93317_g1,c53216_g1,c803_g1,c28372_g1,c58914_g1,c31614_g1,c39918_g1,c49382_g2,c36575_g1,c81371_g1,c29237_g1,c52413_g4,c48410_g1,c111162_g1,c87775_g1,c38077_g1,c49295_g1,c99863_g1,c52836_g1,c51318_g2,c19284_g1,c50212_g1,c89511_g1,c46254_g1,c88057_g1,c55928_g1,c106501_g1,c45590_g1,c72904_g1,c84028_g1,c63859_g1,c52736_g1,c45329_g1,c84030_g1,c34649_g1,c82246_g1,c19496_g1,c25388_g1,c96691_g1,c63101_g1,c72530_g1,c31460_g1,c36334_g1,c48742_g1,c28530_g1,c28295_g1,c106498_g1,c52836_g3,c43971_g1,c73214_g1,c106343_g1,c35834_g1,c973_g1,c42109_g1,c53264_g1,c62599_g1,c76554_g1,c48871_g2,c44374_g1,c108494_g1,c86879_g1,c14311_g1,c51323_g1,c65507_g1,c30341_g1,c49382_g1,c35127_g1,c40424_g1,c3969_g1,c27608_g1,c109664_g1,c33990_g1,c42368_g1,c110478_g1,c19545_g1,c86149_g1,c48170_g1,c49147_g1,c51720_g1,c35383_g1,c55889_g1,c24816_g1,c17678_g1,c51687_g3,c56501_g1,c40006_g1,c26912_g1,c60531_g1,c104068_g1,c84326_g1,c38807_g1,c26955_g1,c76903_g1,c31251_g1,c4652_g1,c65050_g1,c52168_g1,c34562_g1,c65860_g1,c4201_g1,c102213_g1,c55358_g1,c49292_g1,c41349_g1,c39520_g1,c49853_g1,c18261_g2,c7519_g1,c50875_g1,c75676_g1,c42895_g1,c4631_g1,c18161_g1,c37003_g1,c58019_g1,c40688_g1,c50206_g1,c52413_g3,c19565_g1,c50420_g2,c32969_g1,c98062_g1,c52005_g5,c47810_g2,c87317_g1,c63285_g1,c94737_g1,c43462_g1,c53323_g1,c48999_g2,c60256_g1,c48653_g1,c100075_g1,c45922_g1,c52199_g2,c43600_g1,c24876_g1,c96563_g1,c89998_g1,c15250_g1,c27179_g2,c52719_g7,c100618_g1,c104235_g1,c51687_g2,c48099_g1,c50305_g1,c33990_g2,c47120_g1,c27311_g1,c31781_g1,c56658_g1,c51032_g1,c51294_g1,c1334_g1,c72040_g1,c99310_g1,c46780_g1,c37402_g1,c63562_g1,c73286_g1,c42461_g1,c94065_g1,c91439_g1,c50504_g1,c79435_g1,c45291_g2,c47529_g1,c15161_g1,c27176_g1,c52796_g1,c92988_g1,c105780_g1,c45253_g1,c94111_g1,c52168_g2,c52310_g2,c44599_g1,c98046_g1,c95392_g1,c44104_g1,c88514_g1,c64403_g1 |
| Cell cycle - Caulobacter | ko04112 | 26 | c81886_g1,c44673_g1,c44138_g1,c18555_g1,c67515_g1,c46569_g1,c37355_g2,c57089_g1,c107853_g1,c41784_g1,c86134_g1,c71760_g1,c49047_g1,c95069_g1,c9280_g1,c39829_g1,c54398_g1,c82098_g1,c48792_g1,c45651_g1,c46022_g1,c45670_g2,c76004_g1,c96615_g1,c41171_g1,c56795_g1 |
| Cell cycle - yeast | ko04111 | 125 | c51552_g1,c68732_g1,c112711_g1,c93317_g1,c53216_g1,c6097_g1,c58914_g1,c39918_g1,c49382_g2,c36575_g1,c81371_g1,c29237_g1,c30900_g1,c37584_g1,c48410_g1,c92940_g1,c87775_g1,c45915_g1,c51984_g1,c49295_g1,c99863_g1,c51318_g2,c50212_g1,c46254_g1,c88057_g1,c45590_g1,c72904_g1,c84028_g1,c14649_g1,c45329_g1,c47222_g1,c34649_g1,c51270_g1,c25388_g1,c71535_g1,c47222_g2,c96913_g1,c72530_g1,c31460_g1,c48742_g1,c94369_g1,c28295_g1,c43971_g1,c26107_g1,c106343_g1,c46782_g1,c48871_g2,c44374_g1,c35496_g1,c86879_g1,c103876_g1,c51323_g1,c49893_g1,c30341_g1,c49382_g1,c27608_g1,c15924_g1,c110478_g1,c35623_g1,c11164_g2,c51720_g1,c55889_g1,c24816_g1,c69554_g1,c17678_g1,c26912_g1,c104068_g1,c44611_g1,c66689_g1,c70407_g1,c65860_g1,c55358_g1,c49292_g1,c49853_g1,c18261_g2,c7519_g1,c50875_g1,c42895_g1,c4631_g1,c16392_g1,c50206_g1,c19565_g1,c50420_g2,c32969_g1,c53847_g1,c98062_g1,c52005_g5,c47810_g2,c50872_g1,c63285_g1,c42615_g1,c60256_g1,c48653_g1,c45922_g1,c103481_g1,c43600_g1,c96563_g1,c89998_g1,c10693_g1,c31045_g1,c27179_g2,c48099_g1,c50305_g1,c26398_g1,c27311_g1,c56658_g1,c51032_g1,c51294_g1,c37402_g1,c42461_g1,c50504_g1,c47743_g2,c47529_g1,c15161_g1,c41755_g1,c52796_g1,c56506_g1,c105780_g1,c94111_g1,c48456_g1,c74121_g1,c44599_g1,c95392_g1,c44104_g1,c64403_g1 |
| Endocytosis | ko04144 | 237 | c42436_g1,c94563_g1,c53951_g1,c28235_g1,c51039_g2,c91578_g1,c51588_g2,c81465_g1,c52021_g2,c28486_g1,c35724_g1,c87471_g1,c50203_g2,c29797_g1,c68830_g1,c40971_g1,c108649_g1,c44054_g1,c63785_g1,c65806_g1,c46828_g2,c95765_g1,c1591_g1,c51397_g1,c71762_g1,c82533_g1,c5979_g1,c80861_g1,c84871_g1,c99424_g1,c43135_g1,c82263_g1,c41491_g1,c90199_g1,c76584_g1,c96066_g1,c39541_g1,c43256_g2,c37866_g2,c63084_g1,c49048_g6,c46828_g3,c42266_g1,c60132_g1,c30224_g1,c52241_g4,c88688_g1,c47248_g1,c76196_g1,c97805_g1,c73322_g1,c25568_g1,c75604_g1,c47840_g1,c92811_g1,c104689_g1,c47436_g1,c19117_g1,c55792_g1,c28466_g1,c19566_g1,c27003_g1,c17053_g1,c98426_g1,c56601_g1,c52888_g1,c43036_g1,c75980_g1,c48770_g2,c83842_g1,c51431_g1,c71422_g1,c36117_g1,c60983_g1,c40746_g1,c49155_g1,c39707_g1,c59458_g1,c61765_g1,c63743_g1,c97101_g1,c99321_g1,c34843_g1,c28119_g1,c65797_g1,c97846_g1,c50203_g1,c12922_g1,c48306_g1,c17873_g1,c26986_g1,c20191_g1,c82733_g1,c30817_g1,c32310_g2,c31376_g1,c93474_g1,c106749_g1,c42033_g1,c92599_g1,c82689_g1,c67087_g1,c39157_g2,c17051_g1,c65057_g1,c46990_g2,c7457_g1,c37720_g1,c63490_g1,c40847_g1,c56780_g1,c27186_g2,c62600_g1,c103460_g1,c54833_g1,c39133_g1,c34581_g1,c23873_g1,c96576_g1,c47446_g1,c63003_g1,c96012_g1,c49914_g1,c10943_g1,c60324_g1,c95347_g1,c109918_g1,c40171_g1,c91460_g1,c63919_g1,c27752_g1,c28439_g1,c88796_g1,c104810_g1,c40913_g2,c43750_g1,c3957_g1,c305_g1,c103497_g1,c42037_g1,c102073_g1,c30134_g1,c56900_g1,c78773_g1,c18935_g1,c105070_g1,c43256_g1,c36058_g1,c32939_g1,c62353_g1,c46828_g1,c17180_g1,c93731_g1,c48602_g1,c49846_g1,c113251_g1,c49741_g1,c82348_g1,c76687_g1,c24808_g1,c87049_g1,c65754_g1,c43933_g1,c98715_g1,c71777_g1,c62342_g1,c12821_g1,c34941_g2,c48218_g1,c16712_g1,c63703_g1,c49198_g1,c72109_g1,c42328_g1,c44901_g1,c71543_g1,c99231_g1,c17193_g1,c32310_g1,c13172_g1,c40516_g1,c30837_g1,c28419_g1,c50232_g1,c109031_g1,c109398_g1,c100654_g1,c49955_g1,c50791_g1,c4455_g1,c75744_g1,c52614_g2,c42436_g2,c95926_g1,c34941_g1,c78836_g1,c55706_g1,c50503_g1,c4502_g1,c102790_g1,c33797_g1,c18447_g1,c51908_g1,c47267_g3,c43036_g2,c29121_g1,c61239_g1,c44745_g1,c41037_g1,c38523_g1,c13411_g1,c56533_g1,c46070_g1,c49004_g10,c19409_g1,c57174_g1,c110498_g1,c104431_g1,c107618_g1,c66511_g1,c74430_g1,c43109_g1,c18483_g1,c34052_g1,c19301_g1,c106030_g1,c43923_g1,c13273_g1,c102593_g1,c42469_g1,c110075_g1,c74487_g1,c49901_g1,c20601_g1,c103303_g1,c51512_g4,c94974_g1 |
| Focal adhesion | ko04510 | 64 | c31614_g1,c109525_g1,c4329_g1,c53983_g1,c48247_g2,c85476_g1,c54485_g1,c54500_g1,c96691_g1,c75818_g1,c61171_g1,c93546_g1,c75097_g1,c38471_g1,c45587_g1,c17647_g1,c7076_g1,c93365_g1,c55924_g1,c27920_g1,c53373_g1,c71662_g1,c50276_g3,c25384_g1,c56501_g1,c30868_g1,c72535_g1,c77336_g1,c29612_g2,c40669_g1,c102213_g1,c38253_g1,c96785_g1,c49917_g2,c96169_g1,c41349_g1,c72326_g1,c18161_g1,c37689_g1,c64363_g1,c48330_g1,c65565_g1,c34368_g1,c40051_g1,c46805_g1,c103419_g1,c94013_g1,c9260_g1,c37697_g1,c59388_g1,c29612_g1,c78655_g1,c95446_g1,c83855_g1,c46989_g1,c65911_g1,c45509_g1,c28281_g1,c48724_g1,c27176_g1,c103367_g1,c18207_g1,c26895_g1,c64976_g1 |
| Gap junction | ko04540 | 74 | c49789_g3,c73933_g1,c31798_g1,c27491_g1,c71974_g1,c110717_g1,c50778_g1,c82683_g1,c43208_g1,c61362_g1,c23517_g2,c89389_g1,c78193_g1,c75818_g1,c4467_g1,c22729_g1,c98214_g1,c37426_g1,c17874_g1,c33691_g2,c112121_g1,c43718_g1,c75097_g1,c11193_g1,c18485_g1,c108021_g1,c97609_g1,c45587_g1,c94760_g1,c98667_g1,c43574_g1,c102600_g1,c42374_g1,c7716_g1,c27820_g1,c40269_g1,c6234_g1,c97068_g1,c79310_g1,c43403_g1,c39151_g1,c35091_g1,c81767_g1,c103873_g1,c59200_g1,c43754_g2,c23367_g1,c39968_g1,c65565_g1,c99303_g1,c40051_g1,c84986_g1,c78021_g1,c74201_g1,c40724_g1,c75936_g1,c83171_g1,c13025_g1,c82318_g1,c109250_g1,c39484_g1,c43718_g2,c90700_g1,c29109_g1,c588_g1,c87945_g1,c98002_g1,c93072_g1,c103686_g1,c43754_g1,c70598_g1,c57541_g1,c23517_g1,c33691_g1 |
| Lysosome | ko04142 | 203 | c79351_g1,c51782_g1,c90042_g1,c2520_g1,c74497_g1,c48345_g1,c17211_g1,c6604_g1,c63671_g1,c105174_g1,c31630_g1,c29259_g1,c96152_g1,c104875_g1,c52664_g1,c107087_g1,c28486_g1,c48316_g1,c4312_g2,c80400_g1,c74040_g1,c108649_g1,c27184_g1,c51416_g1,c85895_g1,c31761_g1,c65870_g1,c37335_g1,c93000_g1,c71762_g1,c29049_g1,c106656_g1,c110625_g1,c11315_g1,c37224_g2,c67745_g1,c29598_g3,c41693_g1,c46075_g1,c42826_g1,c74206_g1,c46476_g2,c48563_g1,c47953_g1,c50004_g1,c99150_g1,c32580_g1,c48701_g1,c83114_g1,c41632_g1,c54133_g1,c66612_g1,c29598_g2,c84247_g1,c105751_g1,c101668_g1,c55792_g1,c93650_g1,c48539_g1,c9292_g1,c83401_g1,c61151_g1,c55170_g1,c100289_g1,c29212_g1,c108150_g1,c85469_g1,c84111_g1,c28919_g1,c63743_g1,c44018_g1,c67578_g1,c48515_g1,c47794_g1,c87332_g1,c39982_g1,c84618_g1,c84191_g1,c13993_g1,c83724_g1,c37581_g1,c110120_g1,c64700_g1,c100216_g1,c37523_g1,c49326_g3,c79824_g1,c69117_g1,c93474_g1,c46406_g1,c13427_g1,c221_g1,c17130_g1,c50682_g1,c51292_g1,c38152_g1,c102717_g1,c56433_g1,c90632_g1,c47774_g1,c10980_g1,c50525_g1,c61900_g1,c72443_g1,c112536_g1,c72480_g1,c53452_g1,c33696_g1,c8957_g1,c103425_g1,c41028_g2,c54107_g1,c12243_g1,c48958_g1,c56354_g1,c95347_g1,c68640_g1,c26063_g1,c107525_g1,c51731_g1,c50714_g1,c76512_g1,c9519_g1,c15085_g1,c40719_g2,c100078_g1,c78773_g1,c73787_g1,c94590_g1,c72702_g1,c103526_g1,c44655_g1,c49679_g1,c75790_g1,c85764_g1,c42493_g1,c36255_g1,c93731_g1,c75202_g1,c39769_g1,c97495_g1,c27303_g1,c76687_g1,c53064_g1,c43095_g1,c57866_g1,c98114_g1,c45748_g1,c12821_g1,c16248_g1,c47553_g1,c16186_g1,c49785_g1,c31546_g1,c299_g1,c16712_g1,c40485_g1,c106393_g1,c17735_g1,c7360_g1,c28419_g1,c49151_g1,c42308_g1,c17456_g1,c71294_g1,c57663_g1,c83357_g1,c105443_g1,c68029_g1,c75326_g1,c95454_g1,c75744_g1,c72458_g1,c17883_g1,c83892_g1,c25380_g1,c51908_g1,c59642_g1,c61239_g1,c61211_g1,c83009_g1,c41037_g1,c47922_g1,c78216_g1,c42174_g1,c9525_g1,c19624_g1,c63584_g1,c93723_g1,c45792_g2,c95809_g1,c82184_g1,c13273_g1,c96211_g1,c38742_g1,c19423_g1,c34278_g1,c12475_g1,c29256_g2,c85450_g1,c71625_g1,c86104_g1,c94974_g1 |
| Meiosis - yeast | ko04113 | 124 | c68732_g1,c112711_g1,c93317_g1,c53216_g1,c6097_g1,c47883_g1,c49382_g2,c36575_g1,c46305_g1,c30900_g1,c109525_g1,c51191_g1,c48410_g1,c92940_g1,c87775_g1,c53983_g1,c49295_g1,c99863_g1,c27434_g1,c50212_g1,c46254_g1,c88057_g1,c20039_g1,c45590_g1,c72904_g1,c84028_g1,c14649_g1,c45329_g1,c34649_g1,c18431_g1,c54500_g1,c71535_g1,c96913_g1,c48742_g1,c28295_g1,c96128_g1,c42114_g1,c26107_g1,c106343_g1,c50398_g3,c49191_g1,c9107_g1,c48871_g2,c35496_g1,c103876_g1,c1106_g1,c33253_g1,c30341_g1,c49382_g1,c27608_g1,c45799_g1,c15924_g1,c50398_g4,c110478_g1,c26129_g1,c51720_g1,c55889_g1,c50276_g3,c25384_g1,c17678_g1,c111711_g1,c50398_g2,c104068_g1,c44611_g1,c66689_g1,c70407_g1,c29184_g1,c31639_g1,c29612_g2,c96785_g1,c56408_g1,c49917_g2,c49292_g1,c96169_g1,c49853_g1,c18261_g2,c7519_g1,c42895_g1,c16392_g1,c50206_g1,c19565_g1,c50420_g2,c32969_g1,c53847_g1,c98062_g1,c63285_g1,c42615_g1,c48330_g1,c46805_g1,c103419_g1,c45922_g1,c94013_g1,c103481_g1,c43600_g1,c97340_g1,c96563_g1,c89998_g1,c20802_g1,c9260_g1,c31045_g1,c111069_g1,c59388_g1,c29612_g1,c11169_g1,c50305_g1,c27311_g1,c51032_g1,c51294_g1,c45509_g1,c37402_g1,c62057_g1,c50504_g1,c47743_g2,c15902_g1,c47529_g1,c52796_g1,c56506_g1,c105780_g1,c109172_g1,c18207_g1,c8765_g1,c48456_g1,c44599_g1,c44104_g1 |
| Oocyte meiosis | ko04114 | 196 | c76621_g1,c68732_g1,c112711_g1,c6097_g1,c803_g1,c26595_g1,c28372_g1,c81371_g1,c46305_g1,c29237_g1,c30900_g1,c51771_g2,c3181_g1,c109525_g1,c48410_g1,c92940_g1,c111162_g1,c53983_g1,c49295_g1,c80845_g1,c50319_g1,c27434_g1,c46254_g1,c33484_g1,c55928_g1,c106501_g1,c45590_g1,c72904_g1,c5974_g1,c14649_g1,c45329_g1,c84030_g1,c34649_g1,c53776_g1,c18431_g1,c54500_g1,c25388_g1,c71535_g1,c96913_g1,c72530_g1,c3185_g1,c36334_g1,c48742_g1,c75818_g1,c28530_g1,c46921_g2,c28295_g1,c106498_g1,c96128_g1,c26107_g1,c973_g1,c50398_g3,c42109_g1,c53264_g1,c62599_g1,c9107_g1,c37692_g1,c48871_g2,c44374_g1,c35496_g1,c75097_g1,c86879_g1,c14311_g1,c103876_g1,c1106_g1,c33253_g1,c65507_g1,c43584_g2,c40424_g1,c3969_g1,c27608_g1,c109664_g1,c33990_g1,c15924_g1,c29860_g1,c67391_g1,c50398_g4,c45587_g1,c110478_g1,c19545_g1,c25760_g1,c84537_g1,c86149_g1,c26129_g1,c97090_g1,c31260_g1,c55889_g1,c24816_g1,c82692_g1,c50276_g3,c34284_g1,c103279_g1,c41052_g1,c25384_g1,c44244_g1,c111711_g1,c50398_g2,c26912_g1,c45851_g1,c60531_g1,c104068_g1,c84326_g1,c66689_g1,c70407_g1,c26955_g1,c4652_g1,c34562_g1,c29612_g2,c4201_g1,c13394_g1,c24007_g1,c46921_g1,c96785_g1,c56408_g1,c49917_g2,c96169_g1,c39520_g1,c49853_g1,c18261_g2,c7519_g1,c50875_g1,c75676_g1,c4631_g1,c37003_g1,c86007_g1,c16392_g1,c19565_g1,c34788_g1,c32969_g1,c6980_g1,c53847_g1,c43877_g1,c47810_g2,c87317_g1,c42615_g1,c94737_g1,c47867_g1,c65337_g1,c60256_g1,c48330_g1,c65565_g1,c41047_g1,c40051_g1,c46805_g1,c103419_g1,c31785_g1,c94013_g1,c103481_g1,c97340_g1,c6637_g1,c96563_g1,c89998_g1,c63909_g1,c9260_g1,c15250_g1,c86481_g1,c27179_g2,c92831_g1,c111069_g1,c59388_g1,c104235_g1,c29612_g1,c82782_g1,c33990_g2,c27311_g1,c31781_g1,c94398_g1,c56658_g1,c51032_g1,c72709_g1,c45509_g1,c72040_g1,c63562_g1,c73286_g1,c94065_g1,c62057_g1,c91439_g1,c50504_g1,c44899_g1,c47743_g2,c15902_g1,c52796_g1,c56506_g1,c105780_g1,c28657_g2,c109172_g1,c68809_g1,c18207_g1,c48456_g1,c98046_g1,c31195_g1,c95392_g1,c48463_g1,c44104_g1,c88514_g1,c64403_g1 |
| p53 signaling pathway | ko04115 | 58 | c48547_g1,c803_g1,c44149_g1,c82053_g2,c111162_g1,c42460_g1,c52836_g1,c108958_g1,c49866_g1,c52736_g1,c84030_g1,c105449_g1,c61369_g1,c39926_g1,c31109_g2,c52836_g3,c35834_g1,c62599_g1,c44491_g1,c30287_g1,c38471_g1,c38609_g1,c14311_g1,c40424_g1,c17647_g1,c6075_g1,c48170_g1,c48444_g1,c57602_g1,c84326_g1,c71045_g1,c31587_g1,c46616_g1,c75676_g1,c40688_g1,c43462_g1,c26062_g1,c64363_g1,c52199_g2,c16834_g1,c24876_g1,c55559_g1,c47335_g3,c52719_g7,c11160_g1,c72040_g1,c61214_g2,c63562_g1,c47082_g1,c73286_g1,c94065_g1,c48724_g1,c52310_g2,c11034_g1,c64976_g1,c88514_g1,c14380_g1,c96545_g1 |
| Peroxisome | ko04146 | 183 | c57420_g1,c54289_g1,c78634_g1,c104858_g1,c88604_g1,c53592_g1,c105979_g1,c23384_g1,c94042_g1,c54766_g1,c30874_g1,c6161_g1,c95562_g1,c62442_g1,c109324_g1,c33816_g1,c54799_g1,c93426_g1,c83586_g1,c27642_g1,c45734_g1,c99472_g1,c90263_g1,c51640_g2,c94179_g1,c47785_g1,c44030_g1,c27856_g1,c110039_g1,c93591_g1,c52959_g1,c13064_g1,c11144_g1,c47670_g1,c9197_g1,c29953_g1,c82712_g1,c31403_g1,c27245_g1,c40431_g1,c12549_g1,c52467_g2,c92986_g1,c41724_g1,c77055_g1,c52595_g1,c72989_g1,c83786_g1,c104952_g1,c64000_g1,c107366_g1,c72851_g1,c24850_g1,c78471_g1,c105841_g1,c50689_g1,c93852_g1,c49370_g2,c52044_g2,c106149_g1,c47650_g1,c42133_g1,c85859_g1,c54787_g1,c103399_g1,c75960_g1,c40403_g1,c104011_g1,c94686_g1,c84521_g1,c94922_g1,c55758_g1,c46926_g4,c40070_g1,c26999_g1,c43253_g1,c48492_g1,c99311_g1,c51480_g1,c57773_g1,c55749_g1,c9388_g1,c105364_g1,c92997_g1,c53626_g1,c47673_g1,c38653_g1,c103655_g1,c52493_g1,c27846_g2,c77643_g1,c93096_g1,c31415_g1,c42337_g1,c108277_g1,c46912_g1,c48887_g1,c53666_g1,c102643_g1,c44958_g2,c62921_g1,c62384_g1,c45230_g1,c108175_g1,c46156_g1,c52393_g3,c105523_g1,c6594_g1,c48887_g2,c42122_g1,c44890_g1,c91334_g1,c27256_g1,c39493_g1,c81878_g1,c37339_g1,c6536_g1,c61340_g1,c43645_g1,c42257_g1,c73577_g1,c62780_g1,c51551_g1,c64873_g1,c48358_g1,c83387_g1,c105775_g1,c98768_g1,c46584_g1,c49071_g1,c51066_g1,c82596_g1,c72204_g1,c100039_g1,c45540_g1,c36879_g1,c73343_g1,c61548_g1,c28048_g1,c9237_g1,c50711_g1,c63784_g1,c16761_g1,c42418_g1,c107241_g1,c59078_g1,c82857_g1,c71604_g1,c29206_g1,c29399_g1,c25133_g1,c54520_g1,c93690_g1,c103892_g1,c58428_g1,c24487_g1,c71698_g1,c790_g1,c38455_g1,c93760_g1,c82737_g1,c54622_g1,c87138_g1,c47394_g1,c43660_g1,c93516_g1,c51162_g1,c47942_g1,c50071_g1,c47033_g1,c43722_g1,c55937_g1,c42703_g1,c68744_g1,c54704_g1,c93069_g1,c86544_g1,c62726_g1,c45704_g1,c44958_g1,c28756_g1,c26736_g1,c39018_g1 |
| Phagosome | ko04145 | 213 | c13238_g1,c49789_g3,c90042_g1,c17158_g1,c9517_g1,c17211_g1,c6604_g1,c4329_g1,c96152_g1,c104875_g1,c79303_g1,c73933_g1,c31798_g1,c84465_g1,c27491_g1,c71974_g1,c18509_g1,c110717_g1,c51416_g1,c48247_g2,c13073_g1,c85476_g1,c50778_g1,c54485_g1,c77978_g1,c73651_g1,c106656_g1,c43208_g1,c61362_g1,c23517_g2,c89389_g1,c78193_g1,c4911_g1,c45576_g1,c49048_g6,c4467_g1,c22729_g1,c108570_g1,c98214_g1,c61171_g1,c37426_g1,c17874_g1,c33971_g2,c33691_g2,c48563_g1,c112121_g1,c93546_g1,c7622_g1,c43718_g1,c17915_g1,c41632_g1,c51930_g1,c105751_g1,c36573_g1,c50260_g1,c45182_g1,c19566_g1,c9292_g1,c11193_g1,c83401_g1,c3915_g1,c18485_g1,c61151_g1,c108021_g1,c55144_g1,c97609_g1,c53783_g1,c48699_g4,c94760_g1,c40746_g1,c7076_g1,c60627_g1,c93365_g1,c55924_g1,c84111_g1,c107131_g1,c98667_g1,c27920_g1,c28919_g1,c23902_g1,c49230_g2,c34843_g1,c47794_g1,c71662_g1,c43574_g1,c28119_g1,c102600_g1,c53821_g1,c57725_g1,c13993_g1,c30184_g1,c26875_g1,c17873_g1,c37300_g1,c26986_g1,c106096_g1,c47187_g1,c30868_g1,c42374_g1,c87756_g1,c7716_g1,c27820_g1,c77336_g1,c17178_g1,c31564_g1,c221_g1,c5914_g1,c17130_g1,c40269_g1,c82933_g1,c6234_g1,c50682_g1,c28233_g1,c17051_g1,c102717_g1,c55469_g1,c90632_g1,c38253_g1,c38150_g1,c72326_g1,c79310_g1,c41028_g2,c43403_g1,c39151_g1,c35091_g1,c56354_g1,c60324_g1,c81767_g1,c26063_g1,c92951_g1,c37689_g1,c103873_g1,c9519_g1,c36740_g1,c15085_g1,c46688_g2,c56900_g1,c59200_g1,c43754_g2,c44602_g1,c103526_g1,c44852_g1,c75790_g1,c23367_g1,c39968_g1,c36255_g1,c99303_g1,c34368_g1,c39769_g1,c27303_g1,c43095_g1,c98114_g1,c59129_g1,c72267_g1,c3963_g1,c27283_g1,c77529_g1,c78021_g1,c58221_g1,c74201_g1,c40724_g1,c4813_g1,c75936_g1,c83171_g1,c2494_g1,c30837_g1,c37697_g1,c42308_g1,c13025_g1,c74638_g1,c71294_g1,c39823_g1,c46465_g1,c78655_g1,c82318_g1,c95446_g1,c83855_g1,c109250_g1,c39484_g1,c43718_g2,c90700_g1,c29109_g1,c588_g1,c16093_g1,c4502_g1,c87945_g1,c43979_g1,c18447_g1,c71628_g1,c98002_g1,c93072_g1,c76881_g1,c28281_g1,c38523_g1,c92224_g1,c103367_g1,c9525_g1,c93723_g1,c52092_g1,c64712_g1,c9281_g1,c46242_g1,c52617_g1,c103686_g1,c43754_g1,c70598_g1,c26895_g1,c110997_g1,c4535_g1,c57541_g1,c74487_g1,c23517_g1,c33691_g1 |
| Regulation of actin cytoskeleton | ko04810 | 123 | c109525_g1,c4329_g1,c104533_g1,c85479_g1,c40168_g1,c96830_g1,c53399_g1,c53983_g1,c51495_g1,c38105_g1,c48247_g2,c41770_g1,c85476_g1,c54485_g1,c51432_g1,c54500_g1,c44759_g1,c41491_g1,c54671_g1,c45576_g1,c97388_g1,c75818_g1,c49571_g1,c61171_g1,c71920_g1,c65939_g1,c17409_g1,c93546_g1,c47840_g1,c51930_g1,c28733_g1,c41727_g1,c75097_g1,c45944_g1,c49307_g3,c41668_g1,c45587_g1,c100593_g1,c7076_g1,c93365_g1,c55924_g1,c27920_g1,c99764_g1,c88318_g1,c71662_g1,c50276_g3,c47762_g1,c25384_g1,c82433_g1,c73389_g1,c30868_g1,c72535_g1,c77336_g1,c46990_g2,c29612_g2,c49571_g2,c55469_g1,c38253_g1,c28071_g1,c96785_g1,c104685_g1,c49917_g2,c96169_g1,c40410_g1,c72326_g1,c21819_g1,c34934_g1,c40171_g1,c91460_g1,c72833_g1,c28439_g1,c37689_g1,c28799_g1,c24394_g1,c43796_g1,c58700_g1,c47441_g2,c32939_g1,c48330_g1,c65565_g1,c34368_g1,c40051_g1,c46805_g1,c103419_g1,c407_g1,c16097_g1,c94013_g1,c64109_g1,c83648_g1,c48318_g1,c9260_g1,c37697_g1,c59388_g1,c26247_g1,c29612_g1,c50791_g1,c78655_g1,c25443_g1,c29144_g1,c95446_g1,c83855_g1,c46989_g1,c46519_g1,c65315_g1,c45509_g1,c45108_g2,c30988_g1,c36591_g1,c76881_g1,c75170_g1,c28281_g1,c103367_g1,c105845_g1,c28605_g1,c52092_g1,c52617_g1,c43923_g1,c18207_g1,c26895_g1,c109715_g1,c105414_g1,c47729_g1,c104593_g1 |
| Regulation of autophagy | ko04140 | 60 | c61071_g1,c1323_g1,c92554_g1,c42570_g1,c47050_g1,c107397_g1,c37511_g1,c51170_g1,c103945_g1,c49016_g1,c36164_g1,c2859_g1,c47871_g1,c45758_g1,c29941_g1,c103771_g1,c11342_g1,c108570_g1,c47524_g2,c49550_g1,c30839_g1,c19559_g1,c53301_g1,c7955_g1,c49819_g4,c45182_g1,c50953_g1,c77996_g1,c78113_g1,c35198_g1,c35630_g1,c93738_g1,c111847_g1,c39542_g2,c50768_g1,c44565_g1,c44174_g1,c47524_g3,c83302_g1,c53653_g1,c39912_g1,c49357_g2,c83486_g1,c50605_g1,c38467_g1,c50102_g1,c50017_g1,c97640_g1,c51856_g1,c30268_g1,c32760_g1,c32229_g1,c41533_g1,c41835_g1,c47524_g1,c76613_g1,c16625_g1,c41153_g1,c48342_g1,c53757_g1 |
| Tight junction | ko04530 | 47 | c68732_g1,c26910_g1,c6097_g1,c30900_g1,c75900_g1,c92940_g1,c82152_g1,c42300_g1,c51984_g1,c98724_g1,c14649_g1,c47222_g1,c71535_g1,c47222_g2,c96913_g1,c26107_g1,c62391_g1,c111102_g1,c35496_g1,c38471_g1,c103876_g1,c66991_g1,c15924_g1,c17647_g1,c69554_g1,c28865_g1,c66689_g1,c52977_g1,c70407_g1,c50176_g1,c35907_g2,c16392_g1,c53847_g1,c42615_g1,c64363_g1,c51765_g1,c103481_g1,c62976_g1,c56627_g1,c47743_g2,c48724_g1,c56506_g1,c41813_g1,c95993_g1,c48456_g1,c43448_g1,c64976_g1 |
| >5. Organismal Systems |  |  |  |
| Adipocytokine signaling pathway | ko04920 | 54 | c61071_g1,c42570_g1,c49070_g1,c81516_g1,c90263_g1,c33552_g1,c43436_g1,c74441_g1,c13064_g1,c47670_g1,c40995_g1,c103771_g1,c47524_g2,c52467_g2,c49550_g1,c41724_g1,c7955_g1,c64000_g1,c50953_g1,c93852_g1,c47650_g1,c46926_g4,c44740_g1,c93738_g1,c44565_g1,c103655_g1,c44174_g1,c77643_g1,c47524_g3,c38500_g1,c39912_g1,c52393_g3,c83486_g1,c87271_g1,c73577_g1,c39240_g1,c16206_g1,c54118_g1,c51066_g1,c97640_g1,c9237_g1,c99976_g1,c30268_g1,c32760_g1,c32229_g1,c87138_g1,c47524_g1,c51162_g1,c59728_g1,c55937_g1,c41153_g1,c93069_g1,c32374_g2,c53757_g1 |
| Adrenergic signaling in cardiomyocytes | ko04261 | 111 | c76621_g1,c68732_g1,c6097_g1,c46305_g1,c86608_g1,c30900_g1,c3181_g1,c109525_g1,c92940_g1,c53983_g1,c51984_g1,c80845_g1,c50319_g1,c27434_g1,c33484_g1,c5974_g1,c14649_g1,c47222_g1,c53776_g1,c18431_g1,c54500_g1,c71535_g1,c47222_g2,c96913_g1,c21694_g1,c3185_g1,c50180_g1,c96128_g1,c26107_g1,c50398_g3,c9107_g1,c37692_g1,c35496_g1,c75097_g1,c9165_g1,c103876_g1,c1106_g1,c33253_g1,c43584_g2,c15924_g1,c29860_g1,c28252_g1,c91916_g1,c67391_g1,c50398_g4,c25760_g1,c84537_g1,c26129_g1,c97090_g1,c31260_g1,c27041_g1,c50276_g3,c34284_g1,c103279_g1,c41052_g1,c25384_g1,c69554_g1,c44244_g1,c111711_g1,c50398_g2,c66689_g1,c70407_g1,c85582_g1,c29612_g2,c24007_g1,c96785_g1,c56408_g1,c49917_g2,c96169_g1,c16392_g1,c34788_g1,c6980_g1,c53847_g1,c42615_g1,c65337_g1,c48330_g1,c5975_g1,c41047_g1,c40051_g1,c46805_g1,c103419_g1,c25469_g1,c31785_g1,c94013_g1,c103481_g1,c97340_g1,c6637_g1,c63909_g1,c9260_g1,c86481_g1,c92831_g1,c111069_g1,c59388_g1,c29612_g1,c82782_g1,c45509_g1,c62057_g1,c82119_g1,c44899_g1,c47743_g2,c15902_g1,c42680_g1,c85931_g1,c56506_g1,c28657_g2,c109172_g1,c68809_g1,c18207_g1,c48456_g1,c31195_g1,c48463_g1 |
| Aldosterone-regulated sodium reabsorption | ko04960 | 15 | c86608_g1,c50180_g1,c75097_g1,c9165_g1,c28252_g1,c91916_g1,c53373_g1,c27041_g1,c85582_g1,c40669_g1,c5975_g1,c40051_g1,c65911_g1,c82119_g1,c85931_g1 |
| Antigen processing and presentation | ko04612 | 203 | c8275_g1,c91578_g1,c81465_g1,c44465_g1,c87471_g1,c68830_g1,c4312_g2,c80400_g1,c40971_g1,c30744_g1,c27184_g1,c65806_g1,c51416_g1,c85895_g1,c68079_g1,c76236_g1,c46828_g2,c31761_g1,c95765_g1,c35896_g1,c29965_g1,c5979_g1,c77011_g1,c71297_g1,c84871_g1,c99424_g1,c90199_g1,c96066_g1,c85426_g1,c37866_g2,c63084_g1,c90291_g1,c59352_g1,c46828_g3,c60132_g1,c84491_g1,c46075_g1,c30224_g1,c88688_g1,c47248_g1,c41569_g1,c76196_g1,c97805_g1,c13933_g1,c73322_g1,c48563_g1,c83756_g1,c75604_g1,c104689_g1,c45475_g1,c82844_g1,c105751_g1,c91696_g1,c19117_g1,c104764_g1,c32804_g1,c27003_g1,c30486_g2,c48503_g2,c96524_g1,c98426_g1,c30486_g3,c49828_g1,c43036_g1,c75980_g1,c74112_g1,c53783_g1,c71422_g1,c36117_g1,c59360_g1,c108150_g1,c18330_g1,c43298_g1,c59458_g1,c84111_g1,c107131_g1,c110650_g1,c49641_g1,c44018_g1,c97101_g1,c56887_g1,c99321_g1,c83184_g1,c35887_g1,c53821_g1,c65797_g1,c37581_g1,c48306_g1,c8448_g1,c37300_g1,c47187_g1,c20191_g1,c87756_g1,c82688_g1,c32310_g2,c42033_g1,c92599_g1,c46406_g1,c16239_g1,c30486_g1,c31830_g1,c70327_g1,c39157_g2,c65057_g1,c24603_g1,c7457_g1,c86026_g1,c72443_g1,c103460_g1,c54833_g1,c68351_g1,c34581_g1,c42106_g2,c23873_g1,c96576_g1,c63003_g1,c53452_g1,c10943_g1,c10662_g1,c54723_g1,c30518_g1,c63919_g1,c107525_g1,c88796_g1,c104810_g1,c64498_g1,c79194_g1,c36740_g1,c15085_g1,c46688_g2,c103497_g1,c102073_g1,c18935_g1,c105070_g1,c36058_g1,c103526_g1,c62353_g1,c49679_g1,c44852_g1,c46828_g1,c72908_g1,c92324_g1,c41138_g1,c87049_g1,c73917_g1,c65754_g1,c3963_g1,c62342_g1,c99250_g1,c77529_g1,c34941_g2,c31546_g1,c58221_g1,c34589_g1,c35043_g1,c4813_g1,c99231_g1,c57085_g1,c44993_g1,c17193_g1,c32310_g1,c13172_g1,c40516_g1,c82349_g1,c109031_g1,c57663_g1,c105795_g1,c104243_g1,c75326_g1,c42572_g1,c9382_g1,c4455_g1,c48401_g2,c18508_g1,c95454_g1,c34941_g1,c72458_g1,c55706_g1,c83892_g1,c45542_g2,c96864_g1,c43036_g2,c61211_g1,c31001_g1,c42174_g1,c13411_g1,c56533_g1,c112008_g1,c17164_g1,c57174_g1,c110498_g1,c107618_g1,c44688_g1,c9281_g1,c74430_g1,c19301_g1,c20543_g1,c26798_g1,c102593_g1,c96211_g1,c69504_g1,c19423_g1,c45023_g1 |
| Axon guidance | ko04360 | 58 | c26595_g1,c31614_g1,c51771_g2,c4329_g1,c40168_g1,c53399_g1,c38105_g1,c48247_g2,c41770_g1,c85476_g1,c54485_g1,c96691_g1,c54671_g1,c46921_g2,c61171_g1,c93546_g1,c28733_g1,c75097_g1,c7076_g1,c93365_g1,c55924_g1,c27920_g1,c71662_g1,c82692_g1,c56501_g1,c45851_g1,c30868_g1,c77336_g1,c102213_g1,c13394_g1,c38253_g1,c28071_g1,c46921_g1,c41349_g1,c72326_g1,c21819_g1,c18161_g1,c86007_g1,c37689_g1,c43877_g1,c47867_g1,c34368_g1,c40051_g1,c407_g1,c37697_g1,c78655_g1,c94398_g1,c29144_g1,c95446_g1,c83855_g1,c46519_g1,c72709_g1,c75170_g1,c28281_g1,c27176_g1,c103367_g1,c26895_g1,c47729_g1 |
| B cell receptor signaling pathway | ko04662 | 48 | c26595_g1,c31614_g1,c51771_g2,c4329_g1,c48247_g2,c85476_g1,c54485_g1,c96691_g1,c75818_g1,c46921_g2,c61171_g1,c93546_g1,c75097_g1,c45587_g1,c7076_g1,c93365_g1,c55924_g1,c27920_g1,c71662_g1,c82692_g1,c56501_g1,c45851_g1,c30868_g1,c77336_g1,c102213_g1,c13394_g1,c38253_g1,c46921_g1,c41349_g1,c72326_g1,c18161_g1,c86007_g1,c37689_g1,c43877_g1,c47867_g1,c65565_g1,c34368_g1,c40051_g1,c37697_g1,c78655_g1,c94398_g1,c95446_g1,c83855_g1,c72709_g1,c28281_g1,c27176_g1,c103367_g1,c26895_g1 |
| Bile secretion | ko04976 | 90 | c30967_g1,c51676_g1,c45117_g1,c86608_g1,c82155_g1,c50441_g1,c83628_g1,c23574_g1,c50437_g2,c13454_g1,c52352_g2,c37057_g1,c83052_g1,c50437_g1,c63694_g1,c50180_g1,c80570_g1,c27421_g1,c103721_g1,c107405_g1,c9165_g1,c103565_g1,c94523_g1,c62577_g1,c47427_g1,c84185_g1,c71536_g1,c28252_g1,c51704_g1,c91916_g1,c79542_g1,c46513_g1,c65387_g1,c52783_g1,c83170_g1,c27041_g1,c64142_g1,c25215_g1,c46967_g1,c45785_g1,c99619_g1,c109396_g1,c111702_g1,c51124_g1,c85582_g1,c46144_g1,c5719_g2,c107734_g1,c86881_g1,c82892_g1,c105420_g1,c58762_g1,c103046_g1,c92812_g1,c30857_g1,c83460_g1,c40527_g1,c44657_g1,c58739_g1,c36294_g1,c63217_g1,c95427_g1,c83539_g1,c107454_g1,c1331_g1,c41708_g1,c5975_g1,c50342_g1,c60935_g1,c30952_g1,c88326_g1,c80963_g1,c13931_g1,c7563_g1,c64617_g1,c47001_g1,c51124_g2,c69734_g1,c51118_g1,c96792_g1,c64272_g1,c29338_g1,c82119_g1,c85931_g1,c105054_g1,c25557_g1,c10652_g1,c92839_g1,c59736_g1,c52260_g5 |
| Carbohydrate digestion and absorption | ko04973 | 18 | c86608_g1,c6348_g1,c50180_g1,c9165_g1,c28252_g1,c91916_g1,c48933_g1,c27041_g1,c100584_g1,c85582_g1,c41900_g1,c5975_g1,c50236_g1,c30673_g1,c82119_g1,c85931_g1,c52381_g3,c52381_g2 |
| Cardiac muscle contraction | ko04260 | 91 | c34735_g1,c95194_g1,c10168_g1,c17113_g1,c60032_g1,c86608_g1,c28591_g1,c86894_g1,c28409_g1,c30615_g1,c83916_g1,c53271_g1,c92603_g1,c11792_g1,c88393_g1,c50180_g1,c2296_g1,c1628_g1,c53271_g2,c61157_g1,c18647_g2,c78090_g1,c24691_g1,c9165_g1,c51278_g2,c24765_g1,c71754_g1,c39385_g2,c28252_g1,c91916_g1,c53040_g1,c14180_g1,c46944_g1,c27041_g1,c27328_g1,c24691_g2,c80156_g1,c40254_g1,c85582_g1,c61622_g1,c28591_g2,c107114_g1,c12872_g1,c106212_g1,c29868_g1,c112501_g1,c89605_g1,c16695_g1,c51278_g3,c107519_g1,c53932_g1,c101459_g1,c110399_g1,c102383_g1,c10633_g1,c86442_g1,c2421_g1,c71718_g1,c71787_g1,c5975_g1,c103267_g1,c18443_g1,c19567_g1,c61560_g1,c71873_g1,c75767_g1,c71632_g1,c28543_g1,c74972_g1,c104037_g1,c15019_g1,c104392_g1,c32556_g1,c95676_g1,c34225_g1,c108546_g1,c52015_g1,c9150_g1,c39644_g1,c1164_g1,c56674_g1,c76908_g1,c82119_g1,c39385_g1,c85931_g1,c39644_g2,c18647_g1,c73323_g1,c106734_g1,c88306_g1,c57606_g1 |
| Chemokine signaling pathway | ko04062 | 47 | c24927_g1,c31614_g1,c4329_g1,c48247_g2,c85476_g1,c54485_g1,c84183_g1,c96691_g1,c75818_g1,c61171_g1,c93546_g1,c75097_g1,c11484_g1,c97112_g1,c99632_g1,c45587_g1,c7076_g1,c93365_g1,c55924_g1,c27920_g1,c71662_g1,c56501_g1,c30868_g1,c72535_g1,c77336_g1,c63116_g1,c102213_g1,c38253_g1,c41349_g1,c72326_g1,c105077_g1,c18161_g1,c37689_g1,c65565_g1,c34368_g1,c40051_g1,c37697_g1,c78655_g1,c95446_g1,c83855_g1,c46989_g1,c45108_g2,c28281_g1,c27176_g1,c103367_g1,c10282_g1,c26895_g1 |
| Cholinergic synapse | ko04725 | 27 | c24927_g1,c82218_g1,c84183_g1,c75818_g1,c75097_g1,c11484_g1,c97112_g1,c99632_g1,c45587_g1,c108597_g1,c5515_g1,c65830_g1,c63116_g1,c105007_g1,c105077_g1,c76704_g1,c65565_g1,c62234_g1,c40051_g1,c95708_g1,c68060_g1,c44899_g1,c10282_g1,c68713_g1,c93218_g1,c110728_g1,c75534_g1 |
| Circadian entrainment | ko04713 | 49 | c76621_g1,c24927_g1,c3181_g1,c80845_g1,c50319_g1,c33484_g1,c5974_g1,c82683_g1,c53776_g1,c84183_g1,c3185_g1,c37692_g1,c75097_g1,c11484_g1,c43584_g2,c97112_g1,c29860_g1,c99632_g1,c67391_g1,c25760_g1,c84537_g1,c97090_g1,c31260_g1,c34284_g1,c103279_g1,c41052_g1,c44244_g1,c63116_g1,c97068_g1,c24007_g1,c105077_g1,c34788_g1,c6980_g1,c65337_g1,c41047_g1,c40051_g1,c31785_g1,c84986_g1,c6637_g1,c63909_g1,c86481_g1,c92831_g1,c82782_g1,c44899_g1,c10282_g1,c28657_g2,c68809_g1,c31195_g1,c48463_g1 |
| Circadian rhythm | ko04710 | 47 | c61071_g1,c42570_g1,c81371_g1,c29237_g1,c71757_g1,c33552_g1,c48377_g1,c25388_g1,c72530_g1,c103771_g1,c47524_g2,c49550_g1,c7955_g1,c44374_g1,c50953_g1,c86879_g1,c93738_g1,c5131_g1,c40631_g1,c24816_g1,c45808_g1,c26912_g1,c44565_g1,c44174_g1,c47524_g3,c38500_g1,c39912_g1,c50875_g1,c4631_g1,c39309_g1,c47810_g2,c83486_g1,c53981_g1,c60256_g1,c16206_g1,c97640_g1,c27179_g2,c30268_g1,c32760_g1,c32229_g1,c56658_g1,c47524_g1,c60393_g1,c41153_g1,c95392_g1,c64403_g1,c53757_g1 |
| Circadian rhythm - fly | ko04711 | 13 | c31614_g1,c71757_g1,c48377_g1,c96691_g1,c5131_g1,c40631_g1,c56501_g1,c102213_g1,c41349_g1,c18161_g1,c39309_g1,c53981_g1,c27176_g1 |
| Circadian rhythm - plant | ko04712 | 55 | c47440_g1,c27235_g1,c75900_g1,c50404_g1,c42460_g1,c99963_g1,c15063_g1,c98724_g1,c52257_g1,c39926_g1,c49733_g1,c17243_g1,c45207_g1,c50239_g1,c51991_g1,c111102_g1,c50477_g1,c52335_g1,c46041_g1,c50501_g1,c15653_g1,c54093_g1,c28865_g1,c52977_g1,c82899_g1,c48465_g2,c26164_g1,c36742_g1,c49524_g1,c33376_g1,c50343_g1,c35498_g1,c38157_g1,c52178_g1,c37669_g1,c52122_g1,c50343_g3,c64830_g1,c55301_g1,c89612_g1,c105985_g1,c62976_g1,c38231_g1,c56627_g1,c49895_g1,c40341_g1,c40956_g1,c47082_g1,c41813_g1,c47926_g1,c95993_g1,c9453_g1,c52178_g2,c43448_g1,c14380_g1 |
| Collecting duct acid secretion | ko04966 | 52 | c90042_g1,c9517_g1,c17211_g1,c6604_g1,c96152_g1,c104875_g1,c79303_g1,c18509_g1,c13073_g1,c73651_g1,c106656_g1,c7622_g1,c17915_g1,c50260_g1,c83401_g1,c3915_g1,c61151_g1,c60627_g1,c28919_g1,c23902_g1,c47794_g1,c13993_g1,c17178_g1,c31564_g1,c221_g1,c5914_g1,c17130_g1,c82933_g1,c102717_g1,c90632_g1,c41028_g2,c56354_g1,c26063_g1,c92951_g1,c44602_g1,c39769_g1,c27303_g1,c43095_g1,c98114_g1,c59129_g1,c27283_g1,c2494_g1,c42308_g1,c71294_g1,c39823_g1,c46465_g1,c43979_g1,c71628_g1,c9525_g1,c93723_g1,c46242_g1,c110997_g1 |
| Cytosolic DNA-sensing pathway | ko04623 | 26 | c43221_g1,c55119_g1,c11071_g1,c24358_g1,c35699_g1,c36570_g1,c40339_g1,c48395_g1,c47111_g1,c52074_g1,c61778_g1,c65126_g1,c46164_g2,c46200_g1,c110063_g1,c109205_g1,c82103_g1,c9491_g1,c38259_g1,c46159_g1,c83832_g1,c49231_g2,c51562_g1,c104305_g1,c27214_g1,c31515_g1 |
| Dopaminergic synapse | ko04728 | 117 | c76621_g1,c68732_g1,c24927_g1,c6097_g1,c31614_g1,c46305_g1,c30900_g1,c3181_g1,c109525_g1,c92940_g1,c53983_g1,c51984_g1,c80845_g1,c50319_g1,c27434_g1,c33484_g1,c5974_g1,c14649_g1,c47222_g1,c53776_g1,c18431_g1,c54500_g1,c84183_g1,c96691_g1,c71535_g1,c47222_g2,c96913_g1,c21694_g1,c3185_g1,c96128_g1,c26107_g1,c50398_g3,c9107_g1,c37692_g1,c35496_g1,c11484_g1,c103876_g1,c1106_g1,c33253_g1,c43584_g2,c97112_g1,c15924_g1,c29860_g1,c30133_g1,c99632_g1,c67391_g1,c50398_g4,c25760_g1,c84537_g1,c26129_g1,c97090_g1,c31260_g1,c50276_g3,c34284_g1,c103279_g1,c41052_g1,c25384_g1,c69554_g1,c44244_g1,c111711_g1,c56501_g1,c50398_g2,c66689_g1,c63116_g1,c70407_g1,c29612_g2,c102213_g1,c24007_g1,c96785_g1,c56408_g1,c49917_g2,c96169_g1,c41349_g1,c105077_g1,c18161_g1,c16392_g1,c47198_g1,c34788_g1,c6980_g1,c53847_g1,c42615_g1,c65337_g1,c48330_g1,c41047_g1,c46805_g1,c103419_g1,c25469_g1,c31785_g1,c94013_g1,c103481_g1,c97340_g1,c6637_g1,c63909_g1,c9260_g1,c86481_g1,c92831_g1,c111069_g1,c82743_g1,c59388_g1,c29612_g1,c82782_g1,c45509_g1,c62057_g1,c44899_g1,c47743_g2,c15902_g1,c27176_g1,c42680_g1,c56506_g1,c10282_g1,c28657_g2,c109172_g1,c68809_g1,c18207_g1,c48456_g1,c31195_g1,c48463_g1 |
| Dorso-ventral axis formation | ko04320 | 5 | c75818_g1,c75097_g1,c45587_g1,c65565_g1,c40051_g1 |
| Endocrine and other factor-regulated calcium reabsorption | ko04961 | 63 | c42436_g1,c94563_g1,c86608_g1,c28486_g1,c35724_g1,c108649_g1,c71762_g1,c82533_g1,c43256_g2,c50180_g1,c25568_g1,c92811_g1,c55792_g1,c28466_g1,c9165_g1,c28252_g1,c83842_g1,c91916_g1,c61765_g1,c63743_g1,c27041_g1,c82733_g1,c30817_g1,c31376_g1,c93474_g1,c67087_g1,c85582_g1,c63490_g1,c56780_g1,c27186_g2,c39133_g1,c95347_g1,c109918_g1,c40913_g2,c305_g1,c78773_g1,c43256_g1,c5975_g1,c93731_g1,c82348_g1,c76687_g1,c98715_g1,c71777_g1,c12821_g1,c16712_g1,c63703_g1,c28419_g1,c50232_g1,c75744_g1,c42436_g2,c95926_g1,c51908_g1,c61239_g1,c41037_g1,c82119_g1,c49004_g10,c19409_g1,c85931_g1,c34052_g1,c106030_g1,c13273_g1,c103303_g1,c94974_g1 |
| Estrogen signaling pathway | ko04915 | 185 | c76621_g1,c8275_g1,c91578_g1,c3181_g1,c81465_g1,c64341_g1,c60514_g1,c80845_g1,c87471_g1,c50319_g1,c68830_g1,c40971_g1,c30744_g1,c33484_g1,c65806_g1,c68079_g1,c76236_g1,c46828_g2,c21454_g1,c5974_g1,c95765_g1,c35896_g1,c45064_g1,c5979_g1,c53776_g1,c106726_g1,c84871_g1,c99424_g1,c90199_g1,c89738_g1,c96066_g1,c3185_g1,c85426_g1,c37866_g2,c63084_g1,c75818_g1,c59352_g1,c46828_g3,c60132_g1,c30224_g1,c88688_g1,c47248_g1,c76196_g1,c97805_g1,c13933_g1,c73322_g1,c75604_g1,c48114_g1,c104689_g1,c45475_g1,c82844_g1,c37692_g1,c19117_g1,c104764_g1,c75097_g1,c865_g1,c27003_g1,c30486_g2,c98426_g1,c30486_g3,c49828_g1,c43036_g1,c43584_g2,c75980_g1,c7804_g1,c29860_g1,c67391_g1,c45587_g1,c71422_g1,c26236_g1,c36117_g1,c59360_g1,c703_g1,c18330_g1,c43298_g1,c59458_g1,c73305_g1,c38832_g1,c54647_g1,c25760_g1,c110650_g1,c84537_g1,c97101_g1,c97090_g1,c56887_g1,c99321_g1,c101207_g1,c31260_g1,c83184_g1,c34284_g1,c103279_g1,c41052_g1,c44244_g1,c65797_g1,c48306_g1,c8448_g1,c20191_g1,c82688_g1,c32310_g2,c42033_g1,c92599_g1,c54634_g1,c95594_g1,c30486_g1,c31830_g1,c70327_g1,c39157_g2,c37958_g2,c65057_g1,c7457_g1,c77560_g1,c86026_g1,c24007_g1,c54819_g1,c103460_g1,c54833_g1,c34581_g1,c23873_g1,c96576_g1,c63003_g1,c10943_g1,c54723_g1,c30518_g1,c63919_g1,c34788_g1,c88796_g1,c104810_g1,c6980_g1,c54926_g1,c79194_g1,c103497_g1,c102073_g1,c98454_g1,c18935_g1,c105070_g1,c36058_g1,c65337_g1,c62353_g1,c46828_g1,c65565_g1,c41047_g1,c40051_g1,c31785_g1,c87049_g1,c65754_g1,c94352_g1,c62342_g1,c97219_g1,c6637_g1,c34941_g2,c63909_g1,c86481_g1,c99231_g1,c57085_g1,c17193_g1,c92831_g1,c32310_g1,c13172_g1,c40516_g1,c82349_g1,c109031_g1,c82782_g1,c105795_g1,c4455_g1,c50576_g1,c34941_g1,c55706_g1,c86000_g1,c96864_g1,c49610_g1,c43036_g2,c13411_g1,c56533_g1,c112008_g1,c57174_g1,c110498_g1,c107618_g1,c74430_g1,c28657_g2,c19301_g1,c68809_g1,c49648_g1,c102593_g1,c31195_g1,c48463_g1 |
| Fat digestion and absorption | ko04975 | 12 | c110883_g1,c41055_g1,c51298_g1,c41564_g1,c50389_g1,c104622_g1,c72822_g1,c1753_g1,c63138_g1,c104856_g1,c15699_g1,c63265_g1 |
| Fc epsilon RI signaling pathway | ko04664 | 32 | c4329_g1,c48247_g2,c85476_g1,c54485_g1,c75818_g1,c61171_g1,c93546_g1,c75097_g1,c45587_g1,c7076_g1,c93365_g1,c55924_g1,c27920_g1,c53373_g1,c71662_g1,c30868_g1,c77336_g1,c40669_g1,c38253_g1,c72326_g1,c37689_g1,c65565_g1,c34368_g1,c40051_g1,c37697_g1,c78655_g1,c95446_g1,c83855_g1,c65911_g1,c28281_g1,c103367_g1,c26895_g1 |
| Fc gamma R-mediated phagocytosis | ko04666 | 117 | c70915_g1,c51039_g2,c110547_g1,c4329_g1,c104533_g1,c85479_g1,c40168_g1,c96830_g1,c53399_g1,c50203_g2,c73569_g1,c29797_g1,c38105_g1,c63785_g1,c48247_g2,c41770_g1,c1591_g1,c85476_g1,c54485_g1,c51432_g1,c44759_g1,c96884_g1,c41491_g1,c54671_g1,c97388_g1,c75818_g1,c49571_g1,c61171_g1,c71920_g1,c17409_g1,c93546_g1,c47840_g1,c104688_g1,c28733_g1,c41727_g1,c75097_g1,c45944_g1,c49876_g1,c52888_g1,c41668_g1,c45587_g1,c100593_g1,c7076_g1,c93365_g1,c55924_g1,c27920_g1,c99764_g1,c88836_g1,c88318_g1,c42666_g1,c71662_g1,c94822_g1,c50203_g1,c82433_g1,c73389_g1,c30868_g1,c77336_g1,c61876_g1,c46990_g2,c49571_g2,c38253_g1,c50859_g1,c28071_g1,c104685_g1,c40410_g1,c72326_g1,c21819_g1,c67900_g1,c34934_g1,c40171_g1,c91460_g1,c74373_g1,c100612_g1,c72833_g1,c28439_g1,c37689_g1,c3957_g1,c30134_g1,c24394_g1,c43796_g1,c58700_g1,c32939_g1,c90282_g1,c65565_g1,c48602_g1,c34368_g1,c40051_g1,c407_g1,c16097_g1,c24808_g1,c64109_g1,c83648_g1,c48318_g1,c111499_g1,c76480_g1,c48692_g2,c37697_g1,c50791_g1,c78655_g1,c29144_g1,c95446_g1,c83855_g1,c46519_g1,c67782_g1,c45108_g2,c30988_g1,c44745_g1,c36591_g1,c75170_g1,c28281_g1,c103367_g1,c43923_g1,c26895_g1,c109715_g1,c47729_g1,c104593_g1,c51512_g4 |
| GABAergic synapse | ko04727 | 66 | c1323_g1,c45383_g1,c76387_g1,c24927_g1,c63330_g1,c107397_g1,c37511_g1,c83165_g1,c103945_g1,c1382_g1,c13965_g1,c36164_g1,c41212_g1,c2859_g1,c94513_g1,c52032_g1,c59098_g1,c84183_g1,c45589_g3,c29941_g1,c95320_g1,c30839_g1,c28114_g2,c19559_g1,c2590_g1,c11484_g1,c104819_g1,c28114_g1,c50971_g1,c97112_g1,c99632_g1,c102809_g1,c44181_g1,c35198_g1,c112258_g1,c57198_g1,c43714_g1,c30097_g1,c85429_g1,c35519_g1,c111847_g1,c39542_g2,c73295_g1,c63116_g1,c65617_g1,c83302_g1,c64671_g1,c105077_g1,c95064_g1,c55062_g1,c45816_g1,c94313_g1,c38467_g1,c8923_g1,c13393_g1,c3591_g1,c105908_g1,c34353_g1,c52918_g1,c41258_g1,c41835_g1,c76613_g1,c16625_g1,c10282_g1,c93711_g1,c61668_g1 |
| Gastric acid secretion | ko04971 | 46 | c76621_g1,c86608_g1,c3181_g1,c80845_g1,c50319_g1,c33484_g1,c5974_g1,c53776_g1,c3185_g1,c50180_g1,c37692_g1,c9165_g1,c43584_g2,c29860_g1,c28252_g1,c91916_g1,c67391_g1,c25760_g1,c84537_g1,c97090_g1,c31260_g1,c27041_g1,c34284_g1,c103279_g1,c41052_g1,c44244_g1,c85582_g1,c24007_g1,c34788_g1,c6980_g1,c65337_g1,c5975_g1,c41047_g1,c31785_g1,c6637_g1,c63909_g1,c86481_g1,c92831_g1,c82782_g1,c82119_g1,c44899_g1,c85931_g1,c28657_g2,c68809_g1,c31195_g1,c48463_g1 |
| Glutamatergic synapse | ko04724 | 52 | c24927_g1,c51039_g2,c26595_g1,c51771_g2,c50203_g2,c29797_g1,c63785_g1,c52032_g1,c59098_g1,c1591_g1,c84183_g1,c95320_g1,c46921_g2,c75097_g1,c11484_g1,c52888_g1,c97112_g1,c99632_g1,c102809_g1,c44181_g1,c112258_g1,c43714_g1,c82692_g1,c85429_g1,c50203_g1,c45851_g1,c73295_g1,c63116_g1,c13394_g1,c46921_g1,c64671_g1,c105077_g1,c95064_g1,c86007_g1,c55062_g1,c43877_g1,c3957_g1,c30134_g1,c47867_g1,c45816_g1,c48602_g1,c40051_g1,c8923_g1,c24808_g1,c3591_g1,c94398_g1,c72709_g1,c44745_g1,c10282_g1,c93711_g1,c61668_g1,c51512_g4 |
| GnRH signaling pathway | ko04912 | 54 | c76621_g1,c51039_g2,c3181_g1,c80845_g1,c50203_g2,c50319_g1,c29797_g1,c33484_g1,c63785_g1,c5974_g1,c1591_g1,c53776_g1,c3185_g1,c75818_g1,c37692_g1,c75097_g1,c52888_g1,c43584_g2,c29860_g1,c67391_g1,c45587_g1,c25760_g1,c84537_g1,c97090_g1,c31260_g1,c34284_g1,c103279_g1,c41052_g1,c44244_g1,c50203_g1,c24007_g1,c34788_g1,c6980_g1,c3957_g1,c30134_g1,c65337_g1,c65565_g1,c48602_g1,c41047_g1,c40051_g1,c31785_g1,c24808_g1,c6637_g1,c63909_g1,c86481_g1,c92831_g1,c82782_g1,c44745_g1,c44899_g1,c28657_g2,c68809_g1,c31195_g1,c51512_g4,c48463_g1 |
| Inflammatory mediator regulation of TRP channels | ko04750 | 66 | c76621_g1,c3181_g1,c109525_g1,c53983_g1,c80845_g1,c50319_g1,c33484_g1,c5974_g1,c53776_g1,c54500_g1,c44921_g1,c3185_g1,c28142_g1,c37692_g1,c43584_g2,c29860_g1,c67391_g1,c25760_g1,c84537_g1,c97090_g1,c31260_g1,c50276_g3,c34284_g1,c103279_g1,c41052_g1,c25384_g1,c44244_g1,c29612_g2,c24007_g1,c96785_g1,c49917_g2,c96169_g1,c107064_g1,c34788_g1,c47159_g1,c47901_g1,c6980_g1,c100363_g1,c65337_g1,c48330_g1,c41047_g1,c46805_g1,c103419_g1,c31785_g1,c94013_g1,c6637_g1,c63909_g1,c9260_g1,c86481_g1,c92831_g1,c59619_g1,c59388_g1,c29612_g1,c82782_g1,c45509_g1,c94735_g1,c44899_g1,c56694_g1,c47159_g2,c49345_g2,c28657_g2,c68809_g1,c18207_g1,c52376_g1,c31195_g1,c48463_g1 |
| Insulin secretion | ko04911 | 13 | c86608_g1,c39845_g1,c50180_g1,c9165_g1,c28252_g1,c91916_g1,c27041_g1,c85582_g1,c5975_g1,c47129_g1,c82119_g1,c44899_g1,c85931_g1 |
| Insulin signaling pathway | ko04910 | 196 | c70915_g1,c76621_g1,c61071_g1,c12991_g1,c42570_g1,c79060_g1,c9128_g1,c31614_g1,c110547_g1,c51829_g1,c84181_g1,c3181_g1,c109525_g1,c35418_g1,c52454_g1,c6348_g1,c48471_g1,c53983_g1,c49070_g1,c51037_g1,c80845_g1,c81516_g1,c50319_g1,c73569_g1,c25801_g1,c38622_g1,c33552_g1,c33484_g1,c40363_g1,c5974_g1,c67708_g1,c9619_g1,c84449_g1,c53776_g1,c54500_g1,c76639_g1,c103438_g1,c96884_g1,c96691_g1,c49803_g1,c75151_g1,c3185_g1,c103771_g1,c38999_g1,c75818_g1,c47524_g2,c94893_g1,c87327_g1,c49550_g1,c56460_g1,c99582_g1,c88495_g1,c7955_g1,c104688_g1,c27032_g1,c37692_g1,c75097_g1,c50953_g1,c52607_g2,c4084_g1,c43584_g2,c52728_g1,c86243_g1,c29860_g1,c67391_g1,c45587_g1,c58517_g1,c88836_g1,c25760_g1,c84537_g1,c97090_g1,c93738_g1,c53373_g1,c42666_g1,c31260_g1,c100492_g1,c50276_g3,c34284_g1,c103279_g1,c41052_g1,c25384_g1,c44244_g1,c56501_g1,c46440_g1,c43166_g1,c72535_g1,c75440_g1,c44565_g1,c95395_g1,c6288_g1,c44174_g1,c100584_g1,c61876_g1,c47524_g3,c38500_g1,c29612_g2,c40669_g1,c46451_g1,c102213_g1,c24007_g1,c50859_g1,c96785_g1,c49917_g2,c39912_g1,c96169_g1,c41349_g1,c28910_g1,c35047_g1,c36720_g1,c67900_g1,c44819_g1,c18161_g1,c6399_g1,c35246_g1,c74373_g1,c53150_g1,c100612_g1,c34788_g1,c6980_g1,c82018_g1,c57623_g1,c100057_g1,c83486_g1,c50256_g2,c65337_g1,c90282_g1,c57038_g1,c48330_g1,c65565_g1,c16206_g1,c8199_g1,c41047_g1,c40051_g1,c46805_g1,c103419_g1,c31785_g1,c6396_g1,c51571_g1,c50236_g1,c94013_g1,c67624_g1,c92791_g1,c7086_g1,c97640_g1,c6637_g1,c53702_g1,c111499_g1,c63909_g1,c50122_g1,c37107_g1,c109913_g1,c9260_g1,c26261_g1,c86481_g1,c39519_g1,c76480_g1,c92831_g1,c11862_g1,c54781_g1,c48692_g2,c30268_g1,c59388_g1,c32760_g1,c29612_g1,c32229_g1,c82782_g1,c30673_g1,c61032_g1,c46989_g1,c65911_g1,c108416_g1,c45509_g1,c67782_g1,c69630_g1,c65633_g1,c13193_g1,c50781_g1,c62790_g1,c48827_g1,c47524_g1,c76110_g1,c70281_g1,c27176_g1,c28657_g2,c52381_g3,c41153_g1,c68809_g1,c18207_g1,c52381_g2,c60648_g1,c31195_g1,c48463_g1,c78933_g1,c106858_g1,c13770_g1,c53757_g1 |
| Leukocyte transendothelial migration | ko04670 | 24 | c4329_g1,c48247_g2,c85476_g1,c54485_g1,c61171_g1,c93546_g1,c7076_g1,c93365_g1,c55924_g1,c27920_g1,c71662_g1,c30868_g1,c77336_g1,c38253_g1,c72326_g1,c37689_g1,c34368_g1,c37697_g1,c78655_g1,c95446_g1,c83855_g1,c28281_g1,c103367_g1,c26895_g1 |
| Long-term depression | ko04730 | 30 | c68732_g1,c6097_g1,c30900_g1,c92940_g1,c14649_g1,c82683_g1,c71535_g1,c96913_g1,c75818_g1,c26107_g1,c35496_g1,c75097_g1,c103876_g1,c15924_g1,c45587_g1,c72535_g1,c66689_g1,c70407_g1,c97068_g1,c16392_g1,c53847_g1,c42615_g1,c65565_g1,c40051_g1,c103481_g1,c84986_g1,c46989_g1,c47743_g2,c56506_g1,c48456_g1 |
| Long-term potentiation | ko04720 | 77 | c76621_g1,c26595_g1,c51771_g2,c3181_g1,c109525_g1,c53983_g1,c80845_g1,c50319_g1,c33484_g1,c5974_g1,c53776_g1,c54500_g1,c3185_g1,c75818_g1,c46921_g2,c37692_g1,c75097_g1,c43584_g2,c29860_g1,c67391_g1,c45587_g1,c25760_g1,c84537_g1,c97090_g1,c35383_g1,c31260_g1,c82692_g1,c50276_g3,c34284_g1,c103279_g1,c41052_g1,c25384_g1,c44244_g1,c51687_g3,c45851_g1,c72535_g1,c29612_g2,c13394_g1,c24007_g1,c46921_g1,c96785_g1,c49917_g2,c96169_g1,c86007_g1,c34788_g1,c6980_g1,c43877_g1,c47867_g1,c65337_g1,c48330_g1,c65565_g1,c41047_g1,c40051_g1,c46805_g1,c103419_g1,c31785_g1,c94013_g1,c6637_g1,c63909_g1,c9260_g1,c86481_g1,c92831_g1,c59388_g1,c51687_g2,c29612_g1,c82782_g1,c94398_g1,c46989_g1,c72709_g1,c45509_g1,c99310_g1,c44899_g1,c28657_g2,c68809_g1,c18207_g1,c31195_g1,c48463_g1 |
| Melanogenesis | ko04916 | 52 | c76621_g1,c31614_g1,c3181_g1,c80845_g1,c50319_g1,c33484_g1,c5974_g1,c53776_g1,c96691_g1,c3185_g1,c75818_g1,c37692_g1,c75097_g1,c43584_g2,c29860_g1,c67391_g1,c45587_g1,c25760_g1,c84537_g1,c97090_g1,c35383_g1,c31260_g1,c34284_g1,c103279_g1,c41052_g1,c44244_g1,c51687_g3,c56501_g1,c102213_g1,c24007_g1,c41349_g1,c18161_g1,c34788_g1,c6980_g1,c65337_g1,c65565_g1,c41047_g1,c40051_g1,c31785_g1,c6637_g1,c63909_g1,c86481_g1,c92831_g1,c51687_g2,c82782_g1,c99310_g1,c44899_g1,c27176_g1,c28657_g2,c68809_g1,c31195_g1,c48463_g1 |
| Mineral absorption | ko04978 | 38 | c2520_g1,c46154_g1,c30509_g1,c86608_g1,c44414_g1,c31630_g1,c99370_g1,c50180_g1,c26015_g1,c110731_g1,c9165_g1,c28252_g1,c91916_g1,c72121_g1,c27041_g1,c83891_g1,c45066_g1,c85582_g1,c47774_g1,c54071_g1,c50525_g1,c44103_g2,c27137_g1,c86044_g1,c21038_g1,c49856_g1,c40447_g1,c87225_g1,c5975_g1,c75237_g1,c45714_g1,c39449_g1,c29096_g1,c110905_g1,c82119_g1,c85931_g1,c214_g1,c52584_g1 |
| Natural killer cell mediated cytotoxicity | ko04650 | 43 | c26595_g1,c51771_g2,c4329_g1,c48247_g2,c85476_g1,c54485_g1,c75818_g1,c46921_g2,c61171_g1,c93546_g1,c75097_g1,c45587_g1,c7076_g1,c93365_g1,c55924_g1,c27920_g1,c71662_g1,c82692_g1,c45851_g1,c30868_g1,c72535_g1,c77336_g1,c13394_g1,c38253_g1,c46921_g1,c72326_g1,c86007_g1,c37689_g1,c43877_g1,c47867_g1,c65565_g1,c34368_g1,c40051_g1,c37697_g1,c78655_g1,c94398_g1,c95446_g1,c83855_g1,c46989_g1,c72709_g1,c28281_g1,c103367_g1,c26895_g1 |
| Neurotrophin signaling pathway | ko04722 | 405 | c76621_g1,c28479_g1,c104104_g1,c82662_g1,c8503_g1,c35897_g1,c42058_g1,c42455_g1,c20428_g1,c28372_g1,c31551_g1,c31284_g1,c31614_g1,c48654_g1,c63236_g1,c49133_g1,c90349_g1,c52047_g3,c3181_g1,c4329_g1,c19656_g1,c67256_g1,c85350_g1,c64798_g1,c36093_g1,c9338_g1,c50509_g1,c51362_g1,c46431_g1,c68166_g1,c51393_g2,c109082_g1,c41136_g1,c50433_g1,c80845_g1,c76045_g1,c50319_g1,c41452_g1,c77468_g1,c48850_g1,c68992_g1,c99939_g1,c67306_g1,c20958_g1,c59703_g1,c70554_g1,c25682_g1,c33484_g1,c55928_g1,c106501_g1,c51493_g1,c28106_g1,c50497_g1,c48247_g2,c87121_g1,c30825_g1,c4653_g1,c55823_g1,c5974_g1,c24811_g1,c44772_g1,c29500_g1,c59145_g1,c51579_g1,c21101_g1,c43041_g1,c19257_g1,c85476_g1,c54485_g1,c93320_g1,c72649_g1,c29983_g2,c43961_g1,c53776_g1,c52309_g1,c48726_g1,c51393_g1,c73826_g1,c20517_g1,c24966_g1,c96691_g1,c106847_g1,c20072_g1,c37055_g1,c77647_g1,c52385_g2,c3185_g1,c49940_g3,c43215_g1,c36334_g1,c99845_g1,c75818_g1,c28530_g1,c89573_g1,c16333_g1,c103884_g1,c106498_g1,c61171_g1,c64857_g1,c24563_g1,c52687_g3,c27364_g1,c49555_g1,c97243_g1,c60036_g1,c44871_g1,c28878_g1,c43552_g1,c93546_g1,c42662_g1,c12545_g1,c973_g1,c19670_g1,c46019_g1,c68322_g1,c99189_g1,c69108_g1,c50706_g2,c53264_g1,c49045_g1,c106613_g1,c37692_g1,c51250_g1,c85938_g1,c48076_g1,c85515_g1,c50542_g1,c75097_g1,c27417_g1,c105342_g1,c46894_g1,c65507_g1,c74335_g1,c43584_g2,c30663_g1,c5967_g1,c3969_g1,c89522_g1,c12183_g1,c109664_g1,c27559_g1,c33990_g1,c45585_g1,c31723_g1,c29860_g1,c50354_g1,c36550_g1,c67391_g1,c45587_g1,c51087_g1,c52810_g1,c44213_g2,c63994_g1,c7076_g1,c19545_g1,c93365_g1,c55924_g1,c27920_g1,c25760_g1,c102241_g1,c84537_g1,c56011_g1,c52221_g2,c86149_g1,c35128_g1,c25032_g1,c97090_g1,c31283_g1,c53373_g1,c31260_g1,c41811_g1,c93658_g1,c38149_g1,c63402_g1,c8983_g2,c71662_g1,c74219_g1,c31287_g1,c73710_g1,c34284_g1,c103279_g1,c41052_g1,c49457_g4,c33637_g1,c44244_g1,c25651_g1,c56501_g1,c2927_g1,c51933_g1,c60531_g1,c45078_g1,c30868_g1,c11780_g1,c72535_g1,c50217_g1,c43622_g1,c48112_g2,c99332_g1,c77336_g1,c35585_g2,c37345_g1,c17662_g1,c26955_g1,c39201_g1,c26842_g1,c48245_g1,c4652_g1,c52206_g3,c38021_g1,c34104_g1,c36833_g1,c26902_g1,c40669_g1,c49999_g4,c52084_g5,c4201_g1,c45277_g1,c102213_g1,c48988_g3,c24007_g1,c38253_g1,c11267_g1,c48713_g2,c47048_g2,c46096_g1,c41349_g1,c39520_g1,c72326_g1,c40803_g1,c38428_g2,c108116_g1,c35585_g1,c97045_g1,c25369_g1,c13250_g1,c44805_g1,c52646_g1,c42711_g1,c18161_g1,c37003_g1,c16958_g1,c65482_g1,c77087_g1,c45556_g1,c84103_g1,c55120_g1,c34788_g1,c51243_g7,c62405_g1,c11520_g1,c48972_g1,c37689_g1,c20000_g1,c48288_g1,c61555_g1,c57458_g1,c58309_g1,c45137_g1,c6980_g1,c38091_g2,c41208_g1,c77837_g1,c106258_g1,c35140_g1,c20246_g1,c87317_g1,c39725_g1,c25026_g1,c51314_g5,c48833_g1,c106429_g1,c34931_g1,c35002_g1,c40640_g1,c94737_g1,c77655_g1,c39243_g1,c51009_g1,c65337_g1,c23872_g1,c46394_g2,c26562_g1,c97184_g1,c12015_g1,c45152_g1,c17204_g1,c1970_g1,c65565_g1,c95560_g1,c109295_g1,c29517_g1,c48337_g1,c45705_g1,c40297_g1,c50480_g1,c71256_g1,c17979_g1,c41047_g1,c34368_g1,c40051_g1,c17204_g2,c45550_g1,c79424_g1,c31785_g1,c110657_g1,c43106_g1,c39874_g1,c16587_g1,c45705_g2,c29193_g1,c75439_g1,c71808_g1,c48425_g1,c41500_g1,c44725_g1,c6637_g1,c38710_g1,c96255_g1,c53988_g1,c44980_g1,c63909_g1,c49276_g4,c92119_g1,c46059_g2,c15250_g1,c86481_g1,c47535_g2,c52687_g2,c63601_g1,c32428_g1,c74294_g1,c63478_g1,c92831_g1,c2959_g1,c36767_g1,c37697_g1,c110032_g1,c104235_g1,c51379_g1,c31527_g1,c82782_g1,c52047_g1,c78655_g1,c33990_g2,c84912_g1,c14738_g1,c31781_g1,c48723_g1,c95446_g1,c83855_g1,c50422_g6,c46989_g1,c70565_g1,c65911_g1,c85278_g1,c27075_g1,c50171_g1,c5896_g1,c88625_g1,c41879_g1,c72114_g1,c12186_g1,c33668_g1,c44897_g1,c22716_g1,c15748_g1,c32004_g1,c27875_g1,c108348_g1,c61603_g1,c34370_g1,c107634_g1,c52773_g1,c29143_g1,c28281_g1,c37602_g1,c44899_g1,c48569_g1,c27176_g1,c5603_g1,c52449_g2,c103367_g1,c52687_g4,c54042_g1,c10126_g1,c62984_g1,c49158_g1,c44871_g2,c28657_g2,c68809_g1,c47048_g1,c26895_g1,c41963_g1,c31673_g1,c38805_g1,c48713_g1,c34410_g1,c33820_g1,c98046_g1,c31195_g1,c59828_g1,c48463_g1,c45364_g1,c607_g1,c86378_g1 |
| NOD-like receptor signaling pathway | ko04621 | 54 | c8275_g1,c64341_g1,c60514_g1,c49996_g1,c30744_g1,c68079_g1,c76236_g1,c21454_g1,c35896_g1,c89738_g1,c85426_g1,c59352_g1,c13933_g1,c40215_g1,c48114_g1,c45475_g1,c82844_g1,c104764_g1,c75097_g1,c30486_g2,c30486_g3,c49828_g1,c7804_g1,c59360_g1,c703_g1,c18330_g1,c43298_g1,c110650_g1,c56887_g1,c101207_g1,c83184_g1,c8448_g1,c82688_g1,c30486_g1,c31830_g1,c70327_g1,c77560_g1,c86026_g1,c54723_g1,c30518_g1,c54926_g1,c79194_g1,c40051_g1,c94352_g1,c97219_g1,c57085_g1,c82349_g1,c105795_g1,c50576_g1,c86000_g1,c96864_g1,c49610_g1,c112008_g1,c61945_g1 |
| Olfactory transduction | ko04740 | 38 | c76621_g1,c3181_g1,c80845_g1,c50319_g1,c33484_g1,c5974_g1,c82683_g1,c53776_g1,c3185_g1,c37692_g1,c43584_g2,c29860_g1,c67391_g1,c25760_g1,c84537_g1,c97090_g1,c31260_g1,c34284_g1,c103279_g1,c41052_g1,c44244_g1,c24007_g1,c34788_g1,c6980_g1,c65337_g1,c41047_g1,c31785_g1,c84986_g1,c6637_g1,c63909_g1,c86481_g1,c92831_g1,c82782_g1,c44899_g1,c28657_g2,c68809_g1,c31195_g1,c48463_g1 |
| Osteoclast differentiation | ko04380 | 45 | c26595_g1,c51771_g2,c4329_g1,c83807_g1,c48247_g2,c85476_g1,c54485_g1,c75818_g1,c46921_g2,c61171_g1,c93546_g1,c75097_g1,c45587_g1,c7076_g1,c93365_g1,c55924_g1,c27920_g1,c71662_g1,c82692_g1,c45851_g1,c30868_g1,c77336_g1,c13394_g1,c38253_g1,c46921_g1,c72326_g1,c86007_g1,c37689_g1,c43877_g1,c47867_g1,c65565_g1,c34368_g1,c40051_g1,c37697_g1,c78655_g1,c47149_g1,c94398_g1,c95446_g1,c83855_g1,c72709_g1,c26117_g1,c42623_g1,c28281_g1,c103367_g1,c26895_g1 |
| Ovarian Steroidogenesis | ko04913 | 29 | c51154_g3,c84190_g1,c52287_g1,c44921_g1,c28142_g1,c63904_g1,c32774_g1,c21934_g1,c29603_g1,c49343_g1,c37278_g2,c61878_g1,c52376_g2,c33960_g1,c107064_g1,c47159_g1,c47901_g1,c100363_g1,c105322_g1,c47780_g1,c98867_g1,c59619_g1,c94735_g1,c72293_g1,c28343_g1,c56694_g1,c47159_g2,c49345_g2,c52376_g1 |
| Oxytocin signaling pathway | ko04921 | 130 | c76621_g1,c61071_g1,c42570_g1,c84860_g1,c26595_g1,c110339_g1,c51771_g2,c3181_g1,c109525_g1,c53983_g1,c80845_g1,c50319_g1,c33552_g1,c33484_g1,c5974_g1,c85818_g1,c53776_g1,c54500_g1,c65306_g1,c82090_g1,c3185_g1,c103771_g1,c88711_g1,c75818_g1,c46921_g2,c47524_g2,c55218_g1,c95937_g1,c55314_g1,c49550_g1,c37045_g1,c7955_g1,c56537_g1,c37692_g1,c66419_g1,c75097_g1,c50953_g1,c77553_g1,c66026_g1,c43584_g2,c61208_g1,c29860_g1,c67391_g1,c45587_g1,c25760_g1,c84537_g1,c97090_g1,c93738_g1,c31260_g1,c82692_g1,c50276_g3,c34284_g1,c103279_g1,c41052_g1,c25384_g1,c44244_g1,c90079_g1,c45851_g1,c44565_g1,c84088_g1,c44174_g1,c50418_g1,c97068_g1,c47524_g3,c38500_g1,c29612_g2,c24420_g1,c13394_g1,c24007_g1,c112565_g1,c46921_g1,c96785_g1,c69404_g1,c49917_g2,c39912_g1,c96169_g1,c41684_g1,c94431_g1,c86007_g1,c69280_g1,c34788_g1,c71328_g1,c28831_g1,c6980_g1,c43877_g1,c83486_g1,c63363_g1,c47867_g1,c65337_g1,c48330_g1,c65565_g1,c39240_g1,c16206_g1,c41047_g1,c40051_g1,c46805_g1,c103419_g1,c31785_g1,c94300_g1,c94013_g1,c97640_g1,c6637_g1,c41684_g2,c63909_g1,c9260_g1,c86481_g1,c92831_g1,c30268_g1,c59388_g1,c99042_g1,c32760_g1,c29612_g1,c32229_g1,c82782_g1,c94398_g1,c109738_g1,c72709_g1,c45509_g1,c17368_g1,c47524_g1,c44899_g1,c28657_g2,c41153_g1,c41359_g1,c68809_g1,c18207_g1,c59130_g1,c31195_g1,c48463_g1,c53757_g1 |
| Pancreatic secretion | ko04972 | 70 | c42436_g1,c86608_g1,c4329_g1,c48247_g2,c85476_g1,c54485_g1,c41055_g1,c83307_g1,c18280_g1,c34551_g1,c110501_g1,c43256_g2,c50180_g1,c61171_g1,c93546_g1,c92811_g1,c28466_g1,c9165_g1,c28252_g1,c91916_g1,c7076_g1,c93365_g1,c55924_g1,c27920_g1,c74333_g1,c71662_g1,c27041_g1,c67359_g1,c30868_g1,c30817_g1,c31376_g1,c77336_g1,c85582_g1,c63490_g1,c38253_g1,c27186_g2,c39133_g1,c72326_g1,c109918_g1,c37689_g1,c40913_g2,c305_g1,c43256_g1,c95129_g1,c5975_g1,c34368_g1,c47509_g1,c71777_g1,c63703_g1,c37697_g1,c78655_g1,c95446_g1,c83855_g1,c42436_g2,c92985_g1,c64069_g1,c105274_g1,c7509_g1,c95905_g1,c28281_g1,c82119_g1,c19409_g1,c103367_g1,c85931_g1,c54846_g1,c34052_g1,c51960_g1,c106030_g1,c26895_g1,c103303_g1 |
| Phototransduction | ko04744 | 43 | c76621_g1,c24927_g1,c3181_g1,c80845_g1,c50319_g1,c33484_g1,c5974_g1,c53776_g1,c84183_g1,c3185_g1,c37692_g1,c11484_g1,c43584_g2,c97112_g1,c29860_g1,c99632_g1,c67391_g1,c25760_g1,c84537_g1,c97090_g1,c31260_g1,c34284_g1,c103279_g1,c41052_g1,c44244_g1,c63116_g1,c24007_g1,c105077_g1,c34788_g1,c6980_g1,c65337_g1,c41047_g1,c31785_g1,c6637_g1,c63909_g1,c86481_g1,c92831_g1,c82782_g1,c10282_g1,c28657_g2,c68809_g1,c31195_g1,c48463_g1 |
| Phototransduction - fly | ko04745 | 36 | c76621_g1,c3181_g1,c80845_g1,c50319_g1,c33484_g1,c5974_g1,c53776_g1,c3185_g1,c37692_g1,c43584_g2,c29860_g1,c67391_g1,c25760_g1,c84537_g1,c97090_g1,c31260_g1,c34284_g1,c103279_g1,c41052_g1,c44244_g1,c24007_g1,c34788_g1,c6980_g1,c65337_g1,c41047_g1,c31785_g1,c6637_g1,c63909_g1,c86481_g1,c92831_g1,c82782_g1,c44899_g1,c28657_g2,c68809_g1,c31195_g1,c48463_g1 |
| Plant-pathogen interaction | ko04626 | 361 | c76621_g1,c64386_g1,c69693_g1,c94998_g1,c47792_g1,c44051_g1,c106277_g1,c38110_g1,c96916_g1,c48957_g1,c58752_g1,c8275_g1,c12032_g1,c43333_g1,c60867_g1,c48412_g1,c110134_g1,c3181_g1,c31601_g1,c39777_g1,c105766_g1,c112522_g1,c64341_g1,c52746_g1,c8751_g1,c47191_g3,c52739_g1,c60514_g1,c49996_g1,c107206_g1,c80845_g1,c48965_g1,c98962_g1,c50319_g1,c70797_g1,c30538_g1,c47693_g1,c37470_g1,c28604_g1,c47336_g1,c52730_g1,c11103_g1,c30744_g1,c33484_g1,c77609_g1,c50774_g1,c68079_g1,c76236_g1,c42294_g1,c98120_g1,c21454_g1,c16658_g1,c5974_g1,c42444_g1,c47191_g4,c35896_g1,c96767_g1,c73966_g1,c53776_g1,c52129_g1,c18472_g1,c62282_g1,c540_g2,c42930_g1,c89738_g1,c87277_g1,c52163_g1,c3185_g1,c85426_g1,c106073_g1,c52847_g2,c76228_g1,c52573_g1,c75818_g1,c46333_g2,c35226_g1,c3965_g2,c59352_g1,c42065_g1,c112776_g1,c38427_g1,c56326_g1,c27208_g1,c50227_g1,c105231_g1,c93920_g1,c13933_g1,c50071_g3,c36022_g1,c40215_g1,c52681_g1,c46071_g1,c46333_g1,c45699_g2,c45713_g1,c65404_g1,c62222_g1,c48114_g1,c45475_g1,c82844_g1,c37692_g1,c104764_g1,c103375_g1,c53895_g1,c39946_g1,c102257_g1,c36226_g1,c61072_g1,c46153_g1,c30486_g2,c62619_g1,c42041_g1,c97473_g1,c35514_g1,c49383_g1,c18080_g1,c38674_g2,c30486_g3,c78715_g1,c13176_g1,c49828_g1,c43584_g2,c37818_g1,c7804_g1,c44227_g1,c105757_g1,c47274_g1,c40879_g1,c88067_g1,c29860_g1,c26982_g1,c67391_g1,c61653_g1,c45587_g1,c44457_g1,c59360_g1,c84245_g1,c703_g1,c68799_g1,c18330_g1,c48401_g1,c48743_g1,c43298_g1,c47383_g1,c34858_g1,c44503_g1,c45629_g1,c47847_g1,c25760_g1,c110650_g1,c97432_g1,c84537_g1,c58416_g1,c85925_g1,c110669_g1,c97090_g1,c56887_g1,c51708_g1,c101207_g1,c107077_g1,c75954_g1,c31260_g1,c38674_g1,c39754_g1,c83184_g1,c32274_g1,c34284_g1,c51443_g1,c103279_g1,c31965_g1,c41052_g1,c27030_g1,c72978_g1,c44244_g1,c103861_g1,c62945_g1,c39357_g1,c62538_g1,c48523_g1,c106140_g1,c52556_g2,c8448_g1,c108548_g1,c33834_g1,c73010_g1,c52183_g1,c82688_g1,c52556_g1,c37193_g2,c78009_g1,c31312_g1,c49142_g1,c30486_g1,c31830_g1,c70327_g1,c69815_g1,c48072_g1,c82191_g1,c46610_g1,c44603_g1,c52847_g1,c39094_g1,c26982_g2,c106926_g1,c27282_g1,c83327_g1,c77560_g1,c75360_g1,c87038_g1,c86026_g1,c87027_g1,c24007_g1,c50890_g1,c47191_g2,c18180_g1,c52733_g1,c40018_g1,c64045_g1,c35501_g1,c18500_g1,c46353_g1,c24483_g1,c49357_g1,c54723_g1,c30518_g1,c58339_g1,c18135_g1,c8997_g1,c34788_g1,c31308_g1,c47572_g2,c52785_g1,c6980_g1,c14060_g1,c54926_g1,c49415_g1,c79194_g1,c56463_g1,c104051_g1,c44114_g1,c43876_g1,c41760_g1,c42096_g1,c49361_g1,c65337_g1,c49714_g1,c41667_g1,c69772_g1,c37953_g1,c65565_g1,c49820_g1,c39270_g1,c41047_g1,c1630_g1,c95391_g1,c44075_g1,c52344_g3,c39332_g1,c103830_g1,c31785_g1,c18569_g1,c541_g1,c45514_g1,c34299_g1,c48264_g1,c6007_g1,c94352_g1,c97219_g1,c62907_g1,c42294_g2,c6637_g1,c50080_g2,c5567_g1,c51599_g1,c92789_g1,c23068_g1,c48662_g1,c100105_g1,c69154_g1,c63909_g1,c52519_g1,c28739_g1,c51453_g7,c51692_g1,c86481_g1,c42383_g2,c57085_g1,c92831_g1,c48929_g1,c98452_g1,c25117_g1,c47999_g1,c82349_g1,c9570_g1,c40506_g1,c75429_g1,c82782_g1,c105795_g1,c63502_g1,c36555_g2,c28612_g1,c33255_g1,c9118_g1,c71916_g1,c50576_g1,c48968_g3,c106778_g1,c36630_g1,c16617_g1,c10657_g1,c103564_g1,c49020_g1,c86000_g1,c34702_g1,c96864_g1,c72250_g1,c52344_g4,c7498_g1,c52398_g1,c82592_g1,c47368_g1,c49610_g1,c52279_g2,c48957_g3,c57037_g1,c47870_g1,c21151_g1,c41986_g1,c7770_g1,c56327_g1,c33019_g1,c31869_g1,c112008_g1,c17970_g1,c76140_g1,c42502_g1,c43204_g1,c54021_g1,c48058_g1,c42657_g2,c28657_g2,c59789_g1,c26243_g1,c37193_g1,c72578_g1,c10962_g1,c68809_g1,c42134_g1,c77106_g1,c36789_g1,c41156_g1,c45116_g1,c52841_g1,c61945_g1,c76545_g1,c76053_g1,c37839_g1,c31195_g1,c48463_g1,c34489_g1,c107510_g1 |
| Platelet activation | ko04611 | 23 | c109525_g1,c53983_g1,c82683_g1,c54500_g1,c75097_g1,c50276_g3,c25384_g1,c97068_g1,c29612_g2,c96785_g1,c49917_g2,c96169_g1,c48330_g1,c40051_g1,c46805_g1,c103419_g1,c94013_g1,c84986_g1,c9260_g1,c59388_g1,c29612_g1,c45509_g1,c18207_g1 |
| PPAR signaling pathway | ko03320 | 89 | c83586_g1,c27642_g1,c99472_g1,c90263_g1,c11103_g1,c27856_g1,c13064_g1,c11144_g1,c47670_g1,c31092_g1,c31403_g1,c37502_g1,c112776_g1,c52467_g2,c47272_g5,c41724_g1,c103085_g1,c62222_g1,c83786_g1,c64000_g1,c105841_g1,c93852_g1,c52044_g2,c47650_g1,c91515_g1,c85859_g1,c54787_g1,c90634_g1,c104011_g1,c46926_g4,c44740_g1,c53373_g1,c57773_g1,c47272_g3,c53626_g1,c90027_g1,c30483_g1,c103655_g1,c77643_g1,c109547_g1,c26124_g1,c40669_g1,c42337_g1,c46912_g1,c108175_g1,c46156_g1,c52393_g3,c6594_g1,c511_g1,c91334_g1,c107193_g1,c6536_g1,c56463_g1,c73577_g1,c50104_g1,c64873_g1,c52968_g1,c46318_g2,c98768_g1,c61703_g1,c18856_g1,c51066_g1,c47272_g2,c9237_g1,c47272_g4,c29399_g1,c40103_g1,c93690_g1,c103892_g1,c28671_g1,c71698_g1,c65911_g1,c82737_g1,c104444_g1,c87138_g1,c1019_g1,c106601_g1,c43660_g1,c51162_g1,c76322_g1,c57537_g1,c55937_g1,c110160_g1,c93069_g1,c51499_g2,c82089_g1,c86544_g1,c76545_g1,c26736_g1 |
| Progesterone-mediated oocyte maturation | ko04914 | 97 | c48547_g1,c36207_g1,c112711_g1,c803_g1,c8275_g1,c48410_g1,c111162_g1,c30744_g1,c89511_g1,c46254_g1,c72904_g1,c68079_g1,c76236_g1,c84030_g1,c35896_g1,c34649_g1,c85426_g1,c48742_g1,c75818_g1,c59352_g1,c28295_g1,c13933_g1,c35834_g1,c42109_g1,c62599_g1,c45475_g1,c82844_g1,c76554_g1,c48871_g2,c104764_g1,c75097_g1,c30486_g2,c14311_g1,c30486_g3,c49828_g1,c40424_g1,c45587_g1,c59360_g1,c18330_g1,c43298_g1,c110650_g1,c48170_g1,c49147_g1,c56887_g1,c55889_g1,c83184_g1,c40006_g1,c8448_g1,c104068_g1,c72535_g1,c84326_g1,c82688_g1,c38807_g1,c30486_g1,c31830_g1,c70327_g1,c76903_g1,c52168_g1,c34562_g1,c86026_g1,c18261_g2,c7519_g1,c75676_g1,c54723_g1,c30518_g1,c19565_g1,c32969_g1,c79194_g1,c52005_g5,c43462_g1,c53323_g1,c65565_g1,c40051_g1,c24876_g1,c96563_g1,c89998_g1,c57085_g1,c82349_g1,c105795_g1,c27311_g1,c51032_g1,c46989_g1,c72040_g1,c46780_g1,c63562_g1,c96864_g1,c73286_g1,c42461_g1,c94065_g1,c91439_g1,c50504_g1,c112008_g1,c105780_g1,c52168_g2,c52310_g2,c44104_g1,c88514_g1 |
| Prolactin signaling pathway | ko04917 | 17 | c31614_g1,c51154_g3,c96691_g1,c75818_g1,c75097_g1,c45587_g1,c56501_g1,c37278_g2,c102213_g1,c41349_g1,c33960_g1,c18161_g1,c65565_g1,c40051_g1,c105322_g1,c44019_g2,c27176_g1 |
| Protein digestion and absorption | ko04974 | 27 | c86608_g1,c82329_g1,c52032_g1,c49004_g9,c45957_g1,c50180_g1,c35225_g1,c81482_g1,c9165_g1,c42340_g2,c28252_g1,c91916_g1,c27041_g1,c42340_g1,c12111_g1,c85582_g1,c101056_g1,c5975_g1,c8923_g1,c55472_g1,c99337_g1,c68183_g1,c82326_g1,c110561_g1,c82119_g1,c85931_g1,c54602_g1 |
| Proximal tubule bicarbonate reclamation | ko04964 | 27 | c18240_g1,c86608_g1,c44498_g1,c42270_g1,c76146_g1,c83834_g1,c50180_g1,c9047_g1,c9165_g1,c40011_g2,c28252_g1,c91916_g1,c45909_g1,c37201_g1,c52142_g1,c27041_g1,c85582_g1,c104067_g1,c45058_g2,c54243_g1,c5975_g1,c45058_g1,c22770_g1,c40011_g1,c83077_g1,c82119_g1,c85931_g1 |
| Renin-angiotensin system | ko04614 | 1 | c10782_g1 |
| Retrograde endocannabinoid signaling | ko04723 | 26 | c24927_g1,c51448_g1,c63330_g1,c13965_g1,c41212_g1,c48966_g1,c94513_g1,c84183_g1,c45589_g3,c28114_g2,c75097_g1,c11484_g1,c28521_g1,c28114_g1,c97112_g1,c99632_g1,c48136_g1,c63116_g1,c11157_g1,c47592_g1,c105077_g1,c40051_g1,c105908_g1,c41258_g1,c10282_g1,c29108_g1 |
| RIG-I-like receptor signaling pathway | ko04622 | 17 | c57341_g1,c39247_g1,c29179_g1,c100646_g1,c49819_g4,c88723_g1,c35630_g1,c66821_g1,c38261_g1,c67912_g1,c989_g1,c29468_g1,c96721_g1,c11362_g1,c73204_g1,c56882_g1,c57054_g1 |
| Salivary secretion | ko04970 | 50 | c76621_g1,c31582_g1,c86608_g1,c3181_g1,c80845_g1,c50319_g1,c33484_g1,c5974_g1,c82683_g1,c53776_g1,c3185_g1,c50180_g1,c37692_g1,c9165_g1,c43584_g2,c29860_g1,c28252_g1,c91916_g1,c67391_g1,c25760_g1,c84537_g1,c97090_g1,c31260_g1,c27041_g1,c34284_g1,c103279_g1,c41052_g1,c44244_g1,c85582_g1,c97068_g1,c24007_g1,c34788_g1,c6980_g1,c65337_g1,c5975_g1,c41047_g1,c31785_g1,c84986_g1,c6637_g1,c63909_g1,c68052_g1,c86481_g1,c92831_g1,c82782_g1,c82119_g1,c85931_g1,c28657_g2,c68809_g1,c31195_g1,c48463_g1 |
| Serotonergic synapse | ko04726 | 27 | c24927_g1,c84183_g1,c44921_g1,c28142_g1,c75818_g1,c75097_g1,c11484_g1,c97112_g1,c99632_g1,c45587_g1,c72535_g1,c63116_g1,c105077_g1,c107064_g1,c47159_g1,c47901_g1,c100363_g1,c65565_g1,c40051_g1,c59619_g1,c46989_g1,c94735_g1,c56694_g1,c47159_g2,c10282_g1,c49345_g2,c52376_g1 |
| Synaptic vesicle cycle | ko04721 | 118 | c94563_g1,c90042_g1,c63330_g1,c9517_g1,c17211_g1,c6604_g1,c96152_g1,c104875_g1,c79303_g1,c28486_g1,c35724_g1,c13965_g1,c93133_g1,c18509_g1,c41212_g1,c108649_g1,c39845_g1,c94513_g1,c13073_g1,c71762_g1,c82533_g1,c73651_g1,c106656_g1,c45589_g3,c10768_g1,c25111_g1,c28114_g2,c25568_g1,c7622_g1,c17915_g1,c41632_g1,c72001_g1,c50260_g1,c55792_g1,c40965_g1,c9292_g1,c83401_g1,c3915_g1,c61151_g1,c28114_g1,c105145_g1,c83842_g1,c60627_g1,c61765_g1,c28919_g1,c23902_g1,c63743_g1,c47794_g1,c13993_g1,c82733_g1,c93474_g1,c17178_g1,c31564_g1,c221_g1,c5914_g1,c17130_g1,c82933_g1,c67087_g1,c50682_g1,c102717_g1,c90632_g1,c56780_g1,c41028_g2,c56354_g1,c95347_g1,c26063_g1,c92951_g1,c78210_g1,c43673_g1,c9519_g1,c78773_g1,c44602_g1,c75790_g1,c53753_g1,c36255_g1,c93731_g1,c47129_g1,c95834_g1,c13393_g1,c39769_g1,c27303_g1,c82348_g1,c76687_g1,c43095_g1,c98114_g1,c59129_g1,c98715_g1,c27283_g1,c12821_g1,c105908_g1,c16712_g1,c52843_g2,c2494_g1,c28419_g1,c48509_g1,c41083_g2,c50232_g1,c42308_g1,c45438_g1,c71294_g1,c39823_g1,c46465_g1,c75744_g1,c41258_g1,c95926_g1,c43979_g1,c51908_g1,c61239_g1,c71628_g1,c41037_g1,c49004_g10,c9525_g1,c93723_g1,c46242_g1,c13273_g1,c110997_g1,c108278_g1,c94974_g1 |
| T cell receptor signaling pathway | ko04660 | 27 | c26595_g1,c31614_g1,c51771_g2,c96691_g1,c75818_g1,c46921_g2,c75097_g1,c45587_g1,c53373_g1,c82692_g1,c56501_g1,c45851_g1,c40669_g1,c102213_g1,c13394_g1,c46921_g1,c41349_g1,c18161_g1,c86007_g1,c43877_g1,c47867_g1,c65565_g1,c40051_g1,c94398_g1,c65911_g1,c72709_g1,c27176_g1 |
| Taste transduction | ko04742 | 8 | c24927_g1,c84183_g1,c11484_g1,c97112_g1,c99632_g1,c63116_g1,c105077_g1,c10282_g1 |
| Thyroid hormone signaling pathway | ko04919 | 61 | c31614_g1,c86608_g1,c52413_g4,c19074_g1,c49070_g1,c81516_g1,c50636_g1,c19284_g1,c45289_g1,c47578_g1,c96691_g1,c50180_g1,c75818_g1,c7923_g1,c73214_g1,c38764_g1,c108494_g1,c45951_g1,c75097_g1,c9165_g1,c52371_g1,c28252_g1,c91916_g1,c45587_g1,c860_g1,c50987_g1,c28513_g1,c53373_g1,c35383_g1,c27041_g1,c51687_g3,c56501_g1,c48564_g1,c6288_g1,c85582_g1,c31251_g1,c65050_g1,c40669_g1,c102213_g1,c41349_g1,c18161_g1,c58019_g1,c51710_g1,c52413_g3,c52497_g4,c65565_g1,c5975_g1,c40051_g1,c45795_g1,c50837_g1,c51687_g2,c65911_g1,c108416_g1,c99310_g1,c64601_g1,c82119_g1,c27176_g1,c85931_g1,c4402_g1,c37114_g1,c4216_g1 |
| Thyroid hormone synthesis | ko04918 | 69 | c97556_g1,c86608_g1,c64341_g1,c60514_g1,c103464_g1,c27228_g1,c21454_g1,c62393_g1,c76398_g1,c39157_g1,c89738_g1,c50180_g1,c43524_g2,c1904_g1,c85090_g1,c48114_g1,c54761_g1,c84054_g1,c9165_g1,c33993_g1,c16419_g1,c41022_g2,c113262_g1,c109997_g1,c7804_g1,c28252_g1,c91916_g1,c703_g1,c88571_g1,c64653_g1,c77041_g1,c84044_g1,c101207_g1,c27041_g1,c96381_g1,c17391_g2,c37300_g1,c35201_g1,c85582_g1,c86069_g1,c41022_g3,c77560_g1,c57277_g1,c42999_g1,c71515_g1,c106600_g1,c54926_g1,c5476_g1,c88094_g1,c9569_g1,c41022_g1,c5975_g1,c43105_g1,c80850_g1,c54295_g1,c78106_g1,c94352_g1,c97219_g1,c84888_g1,c48922_g6,c4813_g1,c1102_g1,c50576_g1,c86000_g1,c49610_g1,c82119_g1,c21974_g1,c48997_g2,c85931_g1 |
| Toll-like receptor signaling pathway | ko04620 | 319 | c28479_g1,c104104_g1,c82662_g1,c8503_g1,c35897_g1,c42058_g1,c42455_g1,c20428_g1,c31551_g1,c31284_g1,c48654_g1,c63236_g1,c49133_g1,c90349_g1,c52047_g3,c4329_g1,c19656_g1,c67256_g1,c85350_g1,c64798_g1,c36093_g1,c9338_g1,c50509_g1,c51362_g1,c46431_g1,c68166_g1,c51393_g2,c109082_g1,c41136_g1,c50433_g1,c76045_g1,c41452_g1,c77468_g1,c48850_g1,c68992_g1,c99939_g1,c67306_g1,c20958_g1,c59703_g1,c70554_g1,c25682_g1,c51493_g1,c28106_g1,c50497_g1,c48247_g2,c87121_g1,c30825_g1,c4653_g1,c55823_g1,c24811_g1,c44772_g1,c29500_g1,c59145_g1,c51579_g1,c21101_g1,c43041_g1,c19257_g1,c85476_g1,c54485_g1,c93320_g1,c72649_g1,c29983_g2,c43961_g1,c52309_g1,c48726_g1,c51393_g1,c73826_g1,c20517_g1,c106847_g1,c20072_g1,c37055_g1,c77647_g1,c52385_g2,c49940_g3,c43215_g1,c99845_g1,c75818_g1,c89573_g1,c16333_g1,c103884_g1,c61171_g1,c64857_g1,c24563_g1,c52687_g3,c27364_g1,c49555_g1,c97243_g1,c60036_g1,c44871_g1,c28878_g1,c43552_g1,c93546_g1,c42662_g1,c12545_g1,c19670_g1,c46019_g1,c99189_g1,c69108_g1,c50706_g2,c49045_g1,c106613_g1,c51250_g1,c85938_g1,c48076_g1,c85515_g1,c50542_g1,c75097_g1,c27417_g1,c105342_g1,c46894_g1,c74335_g1,c30663_g1,c5967_g1,c89522_g1,c12183_g1,c27559_g1,c45585_g1,c31723_g1,c50354_g1,c36550_g1,c45587_g1,c51087_g1,c52810_g1,c44213_g2,c63994_g1,c7076_g1,c93365_g1,c55924_g1,c27920_g1,c102241_g1,c56011_g1,c52221_g2,c35128_g1,c25032_g1,c31283_g1,c41811_g1,c93658_g1,c38149_g1,c63402_g1,c71662_g1,c74219_g1,c31287_g1,c73710_g1,c49457_g4,c33637_g1,c25651_g1,c2927_g1,c51933_g1,c45078_g1,c30868_g1,c11780_g1,c50217_g1,c43622_g1,c48112_g2,c99332_g1,c77336_g1,c35585_g2,c37345_g1,c39201_g1,c48245_g1,c52206_g3,c38021_g1,c34104_g1,c36833_g1,c26902_g1,c49999_g4,c52084_g5,c45277_g1,c48988_g3,c38253_g1,c11267_g1,c48713_g2,c47048_g2,c46096_g1,c72326_g1,c40803_g1,c38428_g2,c108116_g1,c35585_g1,c97045_g1,c25369_g1,c13250_g1,c44805_g1,c52646_g1,c42711_g1,c16958_g1,c65482_g1,c77087_g1,c45556_g1,c84103_g1,c55120_g1,c51243_g7,c48972_g1,c37689_g1,c20000_g1,c48288_g1,c61555_g1,c57458_g1,c58309_g1,c45137_g1,c38091_g2,c41208_g1,c77837_g1,c106258_g1,c35140_g1,c20246_g1,c39725_g1,c25026_g1,c51314_g5,c48833_g1,c106429_g1,c34931_g1,c35002_g1,c40640_g1,c77655_g1,c39243_g1,c51009_g1,c23872_g1,c46394_g2,c26562_g1,c97184_g1,c12015_g1,c45152_g1,c17204_g1,c1970_g1,c65565_g1,c95560_g1,c109295_g1,c48337_g1,c45705_g1,c40297_g1,c50480_g1,c71256_g1,c17979_g1,c34368_g1,c40051_g1,c17204_g2,c79424_g1,c43106_g1,c39874_g1,c16587_g1,c45705_g2,c29193_g1,c75439_g1,c71808_g1,c48425_g1,c41500_g1,c38710_g1,c96255_g1,c53988_g1,c44980_g1,c49276_g4,c92119_g1,c46059_g2,c47535_g2,c52687_g2,c63601_g1,c32428_g1,c74294_g1,c63478_g1,c2959_g1,c36767_g1,c37697_g1,c110032_g1,c51379_g1,c31527_g1,c52047_g1,c78655_g1,c84912_g1,c14738_g1,c48723_g1,c95446_g1,c83855_g1,c50422_g6,c70565_g1,c85278_g1,c27075_g1,c50171_g1,c5896_g1,c88625_g1,c41879_g1,c72114_g1,c12186_g1,c33668_g1,c44897_g1,c22716_g1,c15748_g1,c32004_g1,c27875_g1,c108348_g1,c61603_g1,c34370_g1,c107634_g1,c52773_g1,c29143_g1,c28281_g1,c37602_g1,c48569_g1,c5603_g1,c52449_g2,c103367_g1,c52687_g4,c54042_g1,c10126_g1,c62984_g1,c49158_g1,c44871_g2,c47048_g1,c26895_g1,c41963_g1,c31673_g1,c38805_g1,c48713_g1,c34410_g1,c33820_g1,c59828_g1,c45364_g1,c607_g1,c86378_g1 |
| Vascular smooth muscle contraction | ko04270 | 62 | c76621_g1,c3181_g1,c109525_g1,c53983_g1,c80845_g1,c50319_g1,c33484_g1,c5974_g1,c41055_g1,c53776_g1,c54500_g1,c3185_g1,c75818_g1,c37692_g1,c75097_g1,c43584_g2,c29860_g1,c67391_g1,c45587_g1,c25760_g1,c84537_g1,c97090_g1,c31260_g1,c50276_g3,c34284_g1,c103279_g1,c41052_g1,c25384_g1,c44244_g1,c72535_g1,c97068_g1,c29612_g2,c24007_g1,c96785_g1,c49917_g2,c96169_g1,c34788_g1,c6980_g1,c65337_g1,c48330_g1,c65565_g1,c41047_g1,c40051_g1,c46805_g1,c103419_g1,c31785_g1,c94013_g1,c6637_g1,c63909_g1,c9260_g1,c86481_g1,c92831_g1,c59388_g1,c29612_g1,c82782_g1,c46989_g1,c45509_g1,c28657_g2,c68809_g1,c18207_g1,c31195_g1,c48463_g1 |
| Vasopressin-regulated water reabsorption | ko04962 | 50 | c42436_g1,c18265_g1,c105395_g1,c42462_g1,c43256_g2,c92811_g1,c28466_g1,c34843_g1,c8983_g2,c28119_g1,c26986_g1,c30817_g1,c31376_g1,c17662_g1,c26842_g1,c63490_g1,c46539_g1,c27186_g2,c39133_g1,c60324_g1,c109918_g1,c27271_g1,c62405_g1,c40913_g2,c24657_g1,c305_g1,c43256_g1,c104595_g1,c29517_g1,c41708_g1,c13393_g1,c110657_g1,c44979_g1,c71777_g1,c44725_g1,c72823_g1,c63703_g1,c30837_g1,c74638_g1,c42436_g2,c4502_g1,c18447_g1,c29525_g1,c92224_g1,c19409_g1,c34052_g1,c106030_g1,c38738_g1,c74487_g1,c103303_g1 |
| Vitamin digestion and absorption | ko04977 | 4 | c84121_g1,c105731_g1,c54126_g1,c38488_g1 |
| >6. Human Diseases |  |  |  |
| Acute myeloid leukemia | ko05221 | 26 | c70915_g1,c110547_g1,c49070_g1,c81516_g1,c73569_g1,c96884_g1,c75818_g1,c104688_g1,c75097_g1,c45587_g1,c88836_g1,c42666_g1,c72535_g1,c61876_g1,c50859_g1,c67900_g1,c74373_g1,c100612_g1,c90282_g1,c65565_g1,c40051_g1,c111499_g1,c76480_g1,c48692_g2,c46989_g1,c67782_g1 |
| African trypanosomiasis | ko05143 | 5 | c46587_g1,c88193_g1,c48440_g1,c10782_g1,c63799_g1 |
| Alcoholism | ko05034 | 112 | c76621_g1,c24927_g1,c36854_g1,c3181_g1,c109525_g1,c96970_g1,c20319_g1,c52413_g4,c53983_g1,c80845_g1,c50319_g1,c19284_g1,c33484_g1,c5974_g1,c53776_g1,c18498_g1,c54500_g1,c84183_g1,c3185_g1,c75818_g1,c67397_g1,c73214_g1,c27196_g1,c37692_g1,c108494_g1,c75097_g1,c11484_g1,c70785_g1,c102088_g1,c43584_g2,c97112_g1,c38995_g1,c42626_g1,c29860_g1,c99632_g1,c108594_g1,c67391_g1,c45587_g1,c43363_g1,c25760_g1,c84537_g1,c11134_g1,c44201_g1,c97090_g1,c31260_g1,c40694_g1,c50276_g3,c34284_g1,c103279_g1,c41052_g1,c25384_g1,c44244_g1,c18553_g1,c72535_g1,c10065_g1,c63116_g1,c31251_g1,c65050_g1,c29612_g2,c33965_g1,c24007_g1,c53823_g1,c96785_g1,c424_g1,c49917_g2,c96169_g1,c105077_g1,c58019_g1,c10659_g1,c52413_g3,c34788_g1,c47064_g1,c6980_g1,c49048_g4,c52886_g1,c65337_g1,c61527_g1,c48330_g1,c65565_g1,c39240_g1,c41047_g1,c40051_g1,c46805_g1,c24485_g1,c103419_g1,c31785_g1,c94013_g1,c11337_g1,c31598_g1,c6637_g1,c36348_g2,c63909_g1,c9260_g1,c86481_g1,c92831_g1,c49667_g4,c59388_g1,c46794_g1,c29612_g1,c82782_g1,c875_g1,c46989_g1,c18217_g1,c45509_g1,c10282_g1,c28657_g2,c68809_g1,c18207_g1,c73844_g1,c43507_g2,c31195_g1,c48463_g1 |
| Alzheimer's disease | ko05010 | 369 | c34735_g1,c76621_g1,c52710_g2,c104137_g1,c45645_g1,c95194_g1,c76954_g1,c26595_g1,c10168_g1,c31614_g1,c84193_g1,c17113_g1,c60032_g1,c23083_g1,c75973_g1,c61064_g1,c51771_g2,c3152_g1,c3181_g1,c88181_g1,c105427_g1,c81902_g1,c62196_g1,c16948_g1,c9214_g1,c37459_g1,c28591_g1,c101256_g1,c97900_g1,c86894_g1,c84323_g1,c80845_g1,c28409_g1,c50319_g1,c57794_g1,c99822_g1,c11910_g1,c97317_g1,c61602_g1,c108958_g1,c65926_g1,c36632_g1,c98483_g1,c52710_g3,c33484_g1,c105682_g1,c55308_g1,c48094_g1,c28894_g1,c30615_g1,c5974_g1,c106979_g1,c83916_g1,c75542_g1,c103930_g1,c53271_g1,c92603_g1,c105449_g1,c85837_g1,c48494_g1,c64920_g1,c53776_g1,c56181_g1,c11792_g1,c24966_g1,c46405_g1,c96691_g1,c31137_g1,c27333_g1,c34668_g1,c3185_g1,c88393_g1,c2296_g1,c62789_g1,c42048_g1,c46921_g2,c68666_g1,c44822_g1,c11135_g1,c11911_g1,c1628_g1,c53271_g2,c61157_g1,c49376_g1,c68426_g1,c28261_g1,c18647_g2,c65581_g1,c18990_g2,c78090_g1,c62118_g1,c69677_g1,c77072_g1,c68322_g1,c4249_g1,c110615_g1,c24691_g1,c17949_g1,c41066_g1,c14090_g1,c93047_g1,c37692_g1,c64608_g1,c52377_g3,c75097_g1,c104310_g1,c40208_g1,c78300_g1,c5725_g1,c97305_g1,c39227_g1,c30788_g1,c51278_g2,c36970_g1,c24765_g1,c51476_g1,c74445_g1,c46051_g1,c71754_g1,c5271_g1,c40240_g1,c10937_g1,c43584_g2,c106417_g1,c87061_g1,c12071_g2,c109587_g1,c39385_g2,c29860_g1,c67587_g1,c67391_g1,c53040_g1,c90597_g1,c46069_g1,c14180_g1,c108755_g1,c46944_g1,c39066_g1,c87608_g1,c6075_g1,c25760_g1,c84537_g1,c53377_g1,c28562_g1,c61990_g1,c94865_g1,c46847_g1,c97090_g1,c31260_g1,c60632_g1,c29366_g1,c88839_g1,c42167_g1,c82149_g1,c84114_g1,c82692_g1,c73772_g1,c34284_g1,c41812_g1,c103279_g1,c41052_g1,c51608_g2,c67359_g1,c44244_g1,c75573_g1,c96215_g1,c56501_g1,c27328_g1,c52241_g8,c49207_g1,c45851_g1,c72552_g1,c33826_g1,c103490_g1,c24691_g2,c53443_g1,c71045_g1,c10611_g1,c35073_g1,c31587_g1,c80156_g1,c12725_g1,c43157_g1,c29152_g1,c87592_g1,c40254_g1,c20780_g1,c86867_g1,c27013_g1,c29538_g1,c24947_g1,c55393_g1,c102213_g1,c92961_g1,c61622_g1,c13394_g1,c28591_g2,c24007_g1,c46921_g1,c107114_g1,c43644_g1,c41349_g1,c75828_g1,c84291_g1,c12872_g1,c17210_g1,c106212_g1,c69093_g1,c18990_g1,c66264_g1,c18161_g1,c54708_g1,c86007_g1,c92807_g1,c29868_g1,c112501_g1,c74003_g1,c34788_g1,c101824_g1,c62022_g1,c11520_g1,c24543_g1,c58011_g1,c86260_g1,c89605_g1,c6980_g1,c16695_g1,c43877_g1,c51278_g3,c107519_g1,c39227_g2,c53932_g1,c101459_g1,c10855_g1,c110399_g1,c95203_g1,c108293_g1,c33875_g1,c37192_g1,c102383_g1,c42052_g1,c105411_g1,c10633_g1,c99539_g1,c47867_g1,c18541_g1,c3998_g1,c86442_g1,c65337_g1,c89657_g1,c2421_g1,c71718_g1,c71787_g1,c103267_g1,c41047_g1,c46723_g1,c1141_g1,c40051_g1,c17283_g1,c45550_g1,c31785_g1,c48920_g1,c57755_g1,c47509_g1,c93556_g1,c79539_g1,c18443_g1,c19567_g1,c66467_g1,c52382_g3,c73196_g1,c38274_g1,c61560_g1,c6637_g1,c16834_g1,c40087_g2,c85012_g1,c85745_g1,c63909_g1,c106851_g1,c97646_g1,c71873_g1,c55559_g1,c75767_g1,c28291_g1,c71632_g1,c78097_g1,c86481_g1,c28543_g1,c74972_g1,c67598_g1,c107752_g1,c92831_g1,c97772_g1,c83089_g1,c24600_g1,c104037_g1,c15019_g1,c73802_g1,c104392_g1,c67688_g1,c77154_g1,c63898_g1,c4548_g1,c82782_g1,c22231_g1,c12814_g1,c32556_g1,c95676_g1,c94053_g1,c34812_g1,c11160_g1,c34225_g1,c51932_g6,c108546_g1,c104382_g1,c94398_g1,c11478_g1,c52015_g1,c72709_g1,c49174_g1,c86582_g1,c9150_g1,c39644_g1,c62331_g1,c29694_g1,c1164_g1,c95449_g1,c64069_g1,c105274_g1,c56674_g1,c76908_g1,c56271_g1,c73806_g1,c78711_g1,c39385_g1,c27176_g1,c82205_g1,c68443_g1,c54846_g1,c92160_g1,c39644_g2,c18647_g1,c28657_g2,c73323_g1,c106734_g1,c108219_g1,c80887_g1,c44924_g1,c51960_g1,c88306_g1,c11487_g1,c57606_g1,c68809_g1,c42608_g1,c73672_g1,c108352_g1,c82188_g1,c34164_g1,c94570_g1,c46852_g1,c30814_g1,c15911_g1,c68770_g1,c29335_g1,c31195_g1,c48463_g1,c45920_g1,c96545_g1 |
| Amoebiasis | ko05146 | 38 | c4107_g1,c57019_g1,c94825_g1,c82971_g1,c51105_g1,c83102_g1,c49048_g6,c94592_g1,c87308_g1,c51867_g1,c19566_g1,c48863_g1,c40746_g1,c34843_g1,c28119_g1,c40740_g1,c42210_g1,c17873_g1,c26986_g1,c40086_g1,c61501_g1,c847_g1,c17051_g1,c60324_g1,c56900_g1,c20467_g1,c45498_g1,c17523_g1,c55060_g1,c30837_g1,c41923_g1,c50294_g1,c107578_g1,c4502_g1,c18447_g1,c38523_g1,c47878_g1,c74487_g1 |
| Amphetamine addiction | ko05031 | 66 | c76621_g1,c26595_g1,c51771_g2,c3181_g1,c109525_g1,c53983_g1,c80845_g1,c50319_g1,c33484_g1,c5974_g1,c53776_g1,c54500_g1,c3185_g1,c46921_g2,c37692_g1,c43584_g2,c29860_g1,c67391_g1,c25760_g1,c84537_g1,c97090_g1,c31260_g1,c82692_g1,c50276_g3,c34284_g1,c103279_g1,c41052_g1,c25384_g1,c44244_g1,c45851_g1,c29612_g2,c13394_g1,c24007_g1,c46921_g1,c96785_g1,c49917_g2,c96169_g1,c86007_g1,c34788_g1,c6980_g1,c43877_g1,c47867_g1,c65337_g1,c48330_g1,c41047_g1,c46805_g1,c103419_g1,c31785_g1,c94013_g1,c6637_g1,c63909_g1,c9260_g1,c86481_g1,c92831_g1,c59388_g1,c29612_g1,c82782_g1,c94398_g1,c72709_g1,c45509_g1,c44899_g1,c28657_g2,c68809_g1,c18207_g1,c31195_g1,c48463_g1 |
| Amyotrophic lateral sclerosis (ALS) | ko05014 | 71 | c78634_g1,c26595_g1,c51771_g2,c4329_g1,c54766_g1,c93426_g1,c45734_g1,c94179_g1,c108958_g1,c44030_g1,c48247_g2,c46385_g1,c85476_g1,c54485_g1,c93591_g1,c105449_g1,c46921_g2,c61171_g1,c93546_g1,c94686_g1,c7076_g1,c94922_g1,c93365_g1,c55924_g1,c27920_g1,c6075_g1,c71662_g1,c82692_g1,c105364_g1,c45851_g1,c30868_g1,c71045_g1,c31587_g1,c77336_g1,c13394_g1,c38253_g1,c46921_g1,c62384_g1,c72326_g1,c44890_g1,c27256_g1,c86007_g1,c37689_g1,c43877_g1,c37339_g1,c42257_g1,c47867_g1,c49071_g1,c34368_g1,c100039_g1,c16834_g1,c73343_g1,c28048_g1,c55559_g1,c63784_g1,c51677_g2,c37697_g1,c54520_g1,c78655_g1,c11160_g1,c790_g1,c94398_g1,c95446_g1,c83855_g1,c93760_g1,c72709_g1,c28281_g1,c103367_g1,c54341_g1,c26895_g1,c96545_g1 |
| Bacterial invasion of epithelial cells | ko05100 | 64 | c4329_g1,c104533_g1,c85479_g1,c28486_g1,c108649_g1,c48247_g2,c85476_g1,c54485_g1,c71762_g1,c44759_g1,c49571_g1,c61171_g1,c17409_g1,c93546_g1,c55792_g1,c45944_g1,c7076_g1,c93365_g1,c55924_g1,c27920_g1,c63743_g1,c88318_g1,c71662_g1,c82433_g1,c73389_g1,c30868_g1,c93474_g1,c77336_g1,c34562_g1,c49571_g2,c38253_g1,c72326_g1,c34934_g1,c95347_g1,c72833_g1,c37689_g1,c78773_g1,c93731_g1,c34368_g1,c76687_g1,c16097_g1,c12821_g1,c83648_g1,c48318_g1,c16712_g1,c28419_g1,c37697_g1,c50232_g1,c78655_g1,c75744_g1,c95446_g1,c83855_g1,c45108_g2,c51908_g1,c30988_g1,c61239_g1,c91439_g1,c41037_g1,c28281_g1,c103367_g1,c26895_g1,c13273_g1,c109715_g1,c94974_g1 |
| Basal cell carcinoma | ko05217 | 15 | c31614_g1,c96691_g1,c47587_g1,c39543_g1,c34230_g1,c56501_g1,c48794_g1,c102213_g1,c41349_g1,c50269_g3,c18161_g1,c32354_g1,c52086_g1,c27176_g1,c55367_g1 |
| Bladder cancer | ko05219 | 10 | c19496_g1,c75818_g1,c75097_g1,c45587_g1,c72535_g1,c65565_g1,c40051_g1,c46989_g1,c79435_g1,c45253_g1 |
| Chagas disease (American trypanosomiasis) | ko05142 | 331 | c28479_g1,c104104_g1,c82662_g1,c68732_g1,c8503_g1,c35897_g1,c6097_g1,c42058_g1,c42455_g1,c20428_g1,c31551_g1,c31284_g1,c48654_g1,c63236_g1,c30900_g1,c49133_g1,c90349_g1,c52047_g3,c19656_g1,c67256_g1,c85350_g1,c64798_g1,c36093_g1,c9338_g1,c50509_g1,c92940_g1,c51362_g1,c46431_g1,c68166_g1,c51393_g2,c51984_g1,c109082_g1,c41136_g1,c50433_g1,c76045_g1,c41452_g1,c77468_g1,c88193_g1,c48850_g1,c68992_g1,c99939_g1,c67306_g1,c20958_g1,c59703_g1,c70554_g1,c25682_g1,c51493_g1,c28106_g1,c50497_g1,c87121_g1,c30825_g1,c4653_g1,c55823_g1,c24811_g1,c44772_g1,c29500_g1,c59145_g1,c51579_g1,c21101_g1,c43041_g1,c14649_g1,c19257_g1,c93320_g1,c72649_g1,c47222_g1,c29983_g2,c43961_g1,c52309_g1,c48726_g1,c51393_g1,c73826_g1,c48440_g1,c20517_g1,c106847_g1,c20072_g1,c37055_g1,c71535_g1,c77647_g1,c52385_g2,c47222_g2,c96913_g1,c49940_g3,c43215_g1,c99845_g1,c89573_g1,c16333_g1,c103884_g1,c64857_g1,c24563_g1,c52687_g3,c27364_g1,c49555_g1,c97243_g1,c26107_g1,c60036_g1,c44871_g1,c28878_g1,c43552_g1,c42662_g1,c12545_g1,c19670_g1,c46019_g1,c99189_g1,c69108_g1,c50706_g2,c49045_g1,c106613_g1,c51250_g1,c85938_g1,c35496_g1,c48076_g1,c85515_g1,c50542_g1,c75097_g1,c103876_g1,c27417_g1,c105342_g1,c46894_g1,c74335_g1,c30663_g1,c5967_g1,c89522_g1,c12183_g1,c27559_g1,c15924_g1,c45585_g1,c31723_g1,c50354_g1,c36550_g1,c53783_g1,c51087_g1,c63799_g1,c52810_g1,c44213_g2,c63994_g1,c107131_g1,c102241_g1,c56011_g1,c52221_g2,c35128_g1,c25032_g1,c31283_g1,c41811_g1,c93658_g1,c38149_g1,c63402_g1,c74219_g1,c31287_g1,c73710_g1,c49457_g4,c69554_g1,c33637_g1,c53821_g1,c25651_g1,c2927_g1,c51933_g1,c47187_g1,c45078_g1,c11780_g1,c50217_g1,c87756_g1,c66689_g1,c43622_g1,c48112_g2,c99332_g1,c35585_g2,c37345_g1,c70407_g1,c39201_g1,c48245_g1,c52206_g3,c38021_g1,c34104_g1,c36833_g1,c26902_g1,c49999_g4,c52084_g5,c45277_g1,c48988_g3,c11267_g1,c48713_g2,c47048_g2,c46096_g1,c40803_g1,c38428_g2,c108116_g1,c35585_g1,c97045_g1,c25369_g1,c13250_g1,c44805_g1,c52646_g1,c42711_g1,c16958_g1,c65482_g1,c16392_g1,c77087_g1,c45556_g1,c84103_g1,c55120_g1,c51243_g7,c48972_g1,c20000_g1,c48288_g1,c61555_g1,c57458_g1,c58309_g1,c45137_g1,c38091_g2,c41208_g1,c53847_g1,c77837_g1,c106258_g1,c35140_g1,c36740_g1,c46688_g2,c20246_g1,c39725_g1,c25026_g1,c51314_g5,c48833_g1,c106429_g1,c34931_g1,c42615_g1,c35002_g1,c40640_g1,c77655_g1,c39243_g1,c51009_g1,c23872_g1,c44852_g1,c46394_g2,c26562_g1,c97184_g1,c12015_g1,c45152_g1,c17204_g1,c1970_g1,c95560_g1,c109295_g1,c48337_g1,c45705_g1,c40297_g1,c50480_g1,c71256_g1,c17979_g1,c40051_g1,c17204_g2,c79424_g1,c43106_g1,c39874_g1,c16587_g1,c45705_g2,c29193_g1,c103481_g1,c75439_g1,c3963_g1,c71808_g1,c48425_g1,c41500_g1,c38710_g1,c96255_g1,c77529_g1,c53988_g1,c44980_g1,c58221_g1,c49276_g4,c92119_g1,c46059_g2,c47535_g2,c52687_g2,c63601_g1,c32428_g1,c74294_g1,c63478_g1,c2959_g1,c36767_g1,c110032_g1,c51379_g1,c31527_g1,c52047_g1,c84912_g1,c14738_g1,c48723_g1,c50422_g6,c70565_g1,c85278_g1,c27075_g1,c50171_g1,c5896_g1,c88625_g1,c41879_g1,c72114_g1,c12186_g1,c33668_g1,c44897_g1,c22716_g1,c15748_g1,c32004_g1,c27875_g1,c108348_g1,c61603_g1,c34370_g1,c107634_g1,c52773_g1,c29143_g1,c37602_g1,c47743_g2,c48569_g1,c5603_g1,c52449_g2,c52687_g4,c54042_g1,c56506_g1,c10126_g1,c9281_g1,c62984_g1,c49158_g1,c44871_g2,c47048_g1,c41963_g1,c31673_g1,c38805_g1,c48456_g1,c48713_g1,c34410_g1,c33820_g1,c59828_g1,c45364_g1,c607_g1,c86378_g1 |
| Chemical carcinogenesis | ko05204 | 75 | c48241_g1,c96098_g1,c104014_g1,c40882_g1,c71557_g1,c72152_g1,c39643_g1,c11873_g1,c68793_g1,c84190_g1,c42135_g1,c52287_g1,c93083_g1,c84047_g1,c38812_g1,c39643_g2,c9747_g1,c93937_g1,c63904_g1,c45101_g1,c32774_g1,c98338_g1,c9319_g1,c42729_g1,c60051_g1,c75473_g1,c45697_g1,c22216_g1,c8401_g1,c43652_g1,c106019_g1,c108090_g1,c42912_g1,c21934_g1,c29603_g1,c73597_g1,c49343_g1,c39602_g1,c19935_g2,c87967_g1,c92052_g1,c40100_g1,c61878_g1,c28793_g1,c49385_g1,c52376_g2,c107473_g1,c9390_g1,c78636_g1,c47211_g3,c33782_g2,c84629_g1,c17446_g1,c40_g1,c45977_g1,c47780_g1,c51598_g1,c37410_g1,c19829_g2,c98867_g1,c47211_g2,c37299_g1,c41401_g1,c71559_g1,c87656_g1,c9747_g2,c99066_g1,c26211_g1,c98527_g1,c72293_g1,c28343_g1,c81892_g1,c40395_g1,c94729_g1,c43824_g1 |
| Chronic myeloid leukemia | ko05220 | 18 | c52413_g4,c19284_g1,c19496_g1,c75818_g1,c73214_g1,c108494_g1,c75097_g1,c45587_g1,c72535_g1,c31251_g1,c65050_g1,c58019_g1,c52413_g3,c65565_g1,c40051_g1,c46989_g1,c79435_g1,c45253_g1 |
| Colorectal cancer | ko05210 | 55 | c31614_g1,c4329_g1,c108958_g1,c48247_g2,c85476_g1,c54485_g1,c105449_g1,c96691_g1,c52104_g1,c75818_g1,c61171_g1,c93546_g1,c51923_g1,c75097_g1,c51473_g2,c45587_g1,c51468_g1,c7076_g1,c93365_g1,c55924_g1,c27920_g1,c6075_g1,c71662_g1,c56501_g1,c30868_g1,c72535_g1,c71045_g1,c31587_g1,c77336_g1,c102213_g1,c38253_g1,c41349_g1,c72326_g1,c45156_g1,c108889_g1,c18161_g1,c37689_g1,c65565_g1,c34368_g1,c40051_g1,c16834_g1,c55559_g1,c37697_g1,c78655_g1,c11160_g1,c95446_g1,c83855_g1,c94736_g1,c46989_g1,c61341_g1,c28281_g1,c27176_g1,c103367_g1,c26895_g1,c96545_g1 |
| Endometrial cancer | ko05213 | 24 | c31614_g1,c96691_g1,c75818_g1,c75097_g1,c38471_g1,c45587_g1,c17647_g1,c53373_g1,c56501_g1,c72535_g1,c40669_g1,c102213_g1,c41349_g1,c45156_g1,c108889_g1,c18161_g1,c64363_g1,c65565_g1,c40051_g1,c46989_g1,c65911_g1,c48724_g1,c27176_g1,c64976_g1 |
| Epithelial cell signaling in Helicobacter pylori infection | ko05120 | 82 | c90042_g1,c9517_g1,c17211_g1,c6604_g1,c4329_g1,c96152_g1,c104875_g1,c79303_g1,c18509_g1,c48247_g2,c13073_g1,c85476_g1,c54485_g1,c73651_g1,c106656_g1,c61171_g1,c93546_g1,c7622_g1,c17915_g1,c41632_g1,c50260_g1,c9292_g1,c83401_g1,c3915_g1,c61151_g1,c7076_g1,c60627_g1,c93365_g1,c55924_g1,c27920_g1,c28919_g1,c23902_g1,c47794_g1,c71662_g1,c13993_g1,c30868_g1,c77336_g1,c17178_g1,c31564_g1,c221_g1,c5914_g1,c17130_g1,c82933_g1,c50682_g1,c102717_g1,c90632_g1,c38253_g1,c72326_g1,c41028_g2,c56354_g1,c26063_g1,c92951_g1,c37689_g1,c9519_g1,c44602_g1,c75790_g1,c36255_g1,c34368_g1,c39769_g1,c27303_g1,c43095_g1,c98114_g1,c59129_g1,c27283_g1,c2494_g1,c37697_g1,c42308_g1,c71294_g1,c39823_g1,c46465_g1,c78655_g1,c95446_g1,c83855_g1,c43979_g1,c71628_g1,c28281_g1,c103367_g1,c9525_g1,c93723_g1,c46242_g1,c26895_g1,c110997_g1 |
| Epstein-Barr virus infection | ko05169 | 364 | c43658_g1,c82662_g1,c36207_g1,c103271_g1,c803_g1,c28372_g1,c95531_g1,c31614_g1,c91578_g1,c51588_g2,c90349_g1,c55830_g1,c75900_g1,c81465_g1,c52413_g4,c64798_g1,c50516_g2,c1063_g1,c111162_g1,c49880_g1,c50327_g6,c44590_g1,c87471_g1,c43221_g1,c19284_g1,c48850_g1,c55141_g1,c68830_g1,c40971_g1,c41307_g1,c70554_g1,c55119_g1,c89511_g1,c61530_g1,c27195_g1,c55928_g1,c106501_g1,c98724_g1,c65806_g1,c95175_g1,c73454_g1,c46828_g2,c96199_g1,c95765_g1,c84030_g1,c5979_g1,c11071_g1,c97623_g1,c2892_g1,c84871_g1,c99424_g1,c90199_g1,c37563_g1,c66470_g1,c96691_g1,c96066_g1,c28859_g1,c37866_g2,c36334_g1,c63084_g1,c79969_g1,c90082_g1,c28530_g1,c98263_g1,c39082_g3,c106498_g1,c46828_g3,c27289_g1,c24563_g1,c60132_g1,c30224_g1,c95007_g1,c38201_g1,c88688_g1,c47248_g1,c33339_g1,c39120_g1,c73214_g1,c76196_g1,c97805_g1,c52628_g2,c44871_g1,c73322_g1,c75604_g1,c111102_g1,c973_g1,c45932_g1,c80928_g1,c39196_g1,c39195_g1,c62495_g1,c53264_g1,c104689_g1,c62599_g1,c31109_g1,c54134_g1,c76554_g1,c19117_g1,c108494_g1,c56060_g1,c78123_g1,c50327_g5,c27003_g1,c14311_g1,c98426_g1,c22358_g1,c65507_g1,c8277_g1,c74335_g1,c43036_g1,c52316_g2,c40424_g1,c3969_g1,c75980_g1,c109664_g1,c55321_g1,c34285_g1,c33990_g1,c31723_g1,c36550_g1,c3493_g1,c71422_g1,c36117_g1,c75650_g1,c37733_g1,c59458_g1,c19545_g1,c84078_g1,c24358_g1,c23121_g1,c1136_g1,c35699_g1,c36570_g1,c102241_g1,c97101_g1,c86149_g1,c47075_g1,c99321_g1,c49120_g1,c35383_g1,c48215_g1,c37391_g2,c73710_g1,c80599_g1,c28127_g1,c40339_g1,c33637_g1,c65797_g1,c28865_g1,c51687_g3,c56501_g1,c48395_g1,c40006_g1,c18430_g1,c111208_g1,c48306_g1,c90488_g1,c47111_g1,c60531_g1,c20191_g1,c84326_g1,c52977_g1,c7284_g1,c32310_g2,c44250_g1,c63359_g1,c42033_g1,c52074_g1,c92599_g1,c38807_g1,c5564_g1,c67885_g1,c26955_g1,c39157_g2,c76903_g1,c31251_g1,c61778_g1,c4652_g1,c83690_g1,c65050_g1,c65057_g1,c52168_g1,c7457_g1,c17095_g1,c39082_g2,c4201_g1,c45277_g1,c102213_g1,c65126_g1,c37894_g1,c89737_g1,c89524_g1,c103460_g1,c54833_g1,c34581_g1,c41349_g1,c39520_g1,c23873_g1,c96576_g1,c63003_g1,c25325_g1,c26379_g1,c106131_g1,c75676_g1,c18161_g1,c14104_g1,c10943_g1,c37003_g1,c58019_g1,c46164_g2,c57971_g1,c57919_g1,c63919_g1,c52413_g3,c105296_g1,c88796_g1,c28351_g1,c104810_g1,c38372_g1,c46200_g1,c110063_g1,c34285_g2,c103497_g1,c102073_g1,c109205_g1,c82103_g1,c87317_g1,c18935_g1,c105070_g1,c106429_g1,c59617_g1,c73528_g1,c9491_g1,c94737_g1,c77655_g1,c36058_g1,c53323_g1,c62353_g1,c50841_g1,c52628_g1,c48865_g3,c46828_g1,c4030_g1,c95560_g1,c100075_g1,c44286_g1,c38259_g1,c91936_g1,c24337_g1,c88069_g1,c10477_g1,c82162_g1,c46159_g1,c87049_g1,c65754_g1,c75439_g1,c62342_g1,c845_g1,c81470_g1,c60503_g1,c91919_g1,c34941_g2,c10096_g1,c16610_g1,c65079_g1,c11351_g1,c62976_g1,c15250_g1,c72071_g1,c66674_g1,c99231_g1,c17193_g1,c91598_g1,c83832_g1,c35240_g1,c32310_g1,c110885_g1,c13172_g1,c73057_g1,c40516_g1,c56627_g1,c109031_g1,c16188_g1,c104235_g1,c24911_g1,c51687_g2,c51064_g1,c77864_g1,c105046_g1,c33990_g2,c34592_g1,c4455_g1,c31781_g1,c69270_g1,c52316_g3,c50161_g1,c34941_g1,c55706_g1,c1334_g1,c94639_g1,c33613_g1,c12186_g1,c101900_g1,c27453_g1,c80710_g1,c72040_g1,c80587_g1,c11161_g1,c62985_g1,c22716_g1,c99310_g1,c46780_g1,c63562_g1,c14745_g1,c73286_g1,c49231_g2,c37713_g1,c43036_g2,c94065_g1,c107634_g1,c111047_g1,c28519_g1,c13411_g1,c56533_g1,c45291_g2,c103981_g1,c27176_g1,c57174_g1,c107967_g1,c76850_g1,c110498_g1,c107618_g1,c41813_g1,c51562_g1,c74430_g1,c44871_g2,c54548_g1,c111706_g1,c104305_g1,c40838_g1,c19301_g1,c95993_g1,c27214_g1,c52168_g2,c108467_g1,c40022_g1,c43998_g1,c83600_g1,c43160_g1,c102593_g1,c71554_g1,c31515_g1,c50149_g2,c41377_g1,c43448_g1,c87226_g1,c98046_g1,c41364_g1,c38595_g1,c24769_g1,c45364_g1,c50516_g1,c88514_g1,c77498_g1 |
| Glioma | ko05214 | 53 | c76621_g1,c3181_g1,c49070_g1,c80845_g1,c81516_g1,c50319_g1,c33484_g1,c5974_g1,c53776_g1,c19496_g1,c3185_g1,c75818_g1,c37692_g1,c75097_g1,c38471_g1,c43584_g2,c29860_g1,c67391_g1,c45587_g1,c17647_g1,c25760_g1,c84537_g1,c97090_g1,c31260_g1,c34284_g1,c103279_g1,c41052_g1,c44244_g1,c72535_g1,c24007_g1,c34788_g1,c6980_g1,c65337_g1,c64363_g1,c65565_g1,c41047_g1,c40051_g1,c31785_g1,c6637_g1,c63909_g1,c86481_g1,c92831_g1,c82782_g1,c46989_g1,c44899_g1,c79435_g1,c48724_g1,c45253_g1,c28657_g2,c68809_g1,c31195_g1,c48463_g1,c64976_g1 |
| Hepatitis B | ko05161 | 68 | c36207_g1,c803_g1,c82053_g2,c111162_g1,c39247_g1,c108958_g1,c89511_g1,c96041_g1,c63859_g1,c84030_g1,c105449_g1,c82246_g1,c19496_g1,c63101_g1,c75818_g1,c10987_g1,c100646_g1,c62599_g1,c76554_g1,c75097_g1,c38471_g1,c14311_g1,c40424_g1,c45587_g1,c17647_g1,c88723_g1,c6075_g1,c35383_g1,c51687_g3,c40006_g1,c84326_g1,c71045_g1,c31587_g1,c38807_g1,c76903_g1,c66821_g1,c52168_g1,c53875_g1,c75676_g1,c53323_g1,c64363_g1,c65565_g1,c40051_g1,c96721_g1,c16834_g1,c55559_g1,c100618_g1,c51687_g2,c11160_g1,c11362_g1,c73204_g1,c56882_g1,c72040_g1,c99310_g1,c46780_g1,c63562_g1,c73286_g1,c94065_g1,c77461_g1,c50038_g1,c79435_g1,c48724_g1,c92988_g1,c45253_g1,c52168_g2,c64976_g1,c88514_g1,c96545_g1 |
| Hepatitis C | ko05160 | 49 | c68732_g1,c6097_g1,c31614_g1,c30900_g1,c92940_g1,c51984_g1,c84503_g1,c14649_g1,c47222_g1,c96691_g1,c71535_g1,c28859_g1,c47222_g2,c96913_g1,c26107_g1,c20194_g2,c51595_g1,c35496_g1,c75097_g1,c103876_g1,c22358_g1,c15924_g1,c80552_g1,c53373_g1,c69554_g1,c56501_g1,c72535_g1,c66689_g1,c70407_g1,c40669_g1,c102213_g1,c41349_g1,c18161_g1,c16392_g1,c39145_g2,c53847_g1,c42615_g1,c49394_g1,c44286_g1,c40051_g1,c103481_g1,c12427_g1,c46989_g1,c65911_g1,c47743_g2,c27176_g1,c56506_g1,c39145_g1,c48456_g1 |
| Herpes simplex infection | ko05168 | 138 | c47635_g2,c27156_g1,c803_g1,c95531_g1,c81371_g1,c75900_g1,c109525_g1,c45771_g1,c111162_g1,c82150_g1,c53983_g1,c84503_g1,c108958_g1,c98724_g1,c84030_g1,c105449_g1,c54500_g1,c97623_g1,c47984_g1,c25388_g1,c51644_g1,c28859_g1,c72530_g1,c86211_g1,c100104_g1,c6193_g1,c52628_g2,c99697_g1,c111102_g1,c51595_g1,c62599_g1,c44374_g1,c34130_g1,c31782_g1,c14311_g1,c22358_g1,c52316_g2,c40424_g1,c36405_g1,c23121_g1,c6075_g1,c62848_g1,c35383_g1,c24816_g1,c2913_g1,c50276_g3,c25384_g1,c28865_g1,c51687_g3,c111208_g1,c26912_g1,c27452_g1,c84326_g1,c71045_g1,c52977_g1,c45596_g1,c31587_g1,c60570_g1,c18166_g1,c64893_g1,c29612_g2,c35619_g1,c49681_g1,c37894_g1,c51881_g2,c96785_g1,c47980_g1,c49917_g2,c96169_g1,c46420_g1,c76231_g1,c45369_g1,c26379_g1,c50875_g1,c75676_g1,c4631_g1,c14104_g1,c57919_g1,c17185_g1,c39145_g2,c40957_g1,c47810_g2,c71059_g1,c52628_g1,c48865_g3,c48330_g1,c100075_g1,c44286_g1,c50704_g2,c46805_g1,c103419_g1,c94013_g1,c16834_g1,c60503_g1,c62976_g1,c55559_g1,c44486_g3,c9260_g1,c82670_g1,c37011_g1,c27179_g2,c56627_g1,c42652_g1,c59388_g1,c51687_g2,c29612_g1,c51064_g1,c77864_g1,c34592_g1,c11160_g1,c25526_g1,c29016_g1,c1714_g1,c52316_g3,c51975_g6,c1334_g1,c33613_g1,c45509_g1,c72040_g1,c99310_g1,c63562_g1,c73286_g1,c94065_g1,c111047_g1,c92201_g1,c45291_g2,c14374_g1,c41813_g1,c39145_g1,c95993_g1,c18207_g1,c47434_g1,c50149_g2,c37842_g1,c43448_g1,c95392_g1,c88514_g1,c96545_g1 |
| HTLV-I infection | ko05166 | 172 | c112711_g1,c37132_g1,c52685_g5,c26595_g1,c31614_g1,c27141_g1,c51771_g2,c46744_g1,c50516_g2,c48410_g1,c112082_g1,c47513_g1,c42776_g1,c49295_g1,c99965_g1,c52836_g1,c52039_g1,c55141_g1,c45590_g1,c72904_g1,c63859_g1,c52736_g1,c35641_g1,c84587_g1,c34649_g1,c12089_g1,c71297_g1,c82246_g1,c19496_g1,c96691_g1,c63101_g1,c18148_g1,c2113_g1,c48742_g1,c46921_g2,c28295_g1,c44119_g1,c91611_g1,c52836_g3,c17569_g1,c6498_g1,c43971_g1,c21357_g1,c48871_g2,c16385_g1,c48503_g2,c51323_g1,c96524_g1,c45910_g1,c61358_g1,c27608_g1,c46040_g1,c53783_g1,c24002_g1,c107131_g1,c92670_g1,c35383_g1,c55889_g1,c82692_g1,c73069_g1,c40326_g1,c53821_g1,c18232_g1,c51687_g3,c56501_g1,c45851_g1,c37300_g1,c47187_g1,c104068_g1,c109560_g1,c75246_g1,c87756_g1,c52835_g1,c18014_g1,c33344_g1,c25114_g1,c40081_g1,c67885_g1,c66315_g1,c52486_g1,c56646_g1,c24603_g1,c94320_g1,c17095_g1,c1091_g1,c65860_g1,c102213_g1,c13394_g1,c46921_g1,c41349_g1,c42106_g2,c18261_g2,c18161_g1,c48988_g2,c40688_g1,c86007_g1,c52685_g8,c32969_g1,c43877_g1,c36740_g1,c46688_g2,c52685_g9,c37949_g1,c36262_g1,c47867_g1,c44852_g1,c52685_g6,c34493_g1,c45151_g1,c88069_g1,c57248_g1,c44669_g1,c73917_g1,c52199_g2,c3963_g1,c97532_g1,c77529_g1,c56860_g1,c96563_g1,c89998_g1,c58221_g1,c11351_g1,c72071_g1,c4813_g1,c83293_g1,c57770_g1,c52719_g7,c60888_g1,c100618_g1,c39689_g1,c51687_g2,c104243_g1,c34592_g1,c42572_g1,c27311_g1,c94398_g1,c44434_g1,c51032_g1,c29145_g1,c52685_g1,c18232_g2,c72709_g1,c40852_g1,c27083_g1,c16456_g1,c71735_g1,c91829_g1,c99310_g1,c41525_g1,c35801_g1,c50504_g1,c49341_g3,c65713_g1,c73371_g1,c79435_g1,c27176_g1,c107967_g1,c92988_g1,c4402_g1,c105780_g1,c13104_g1,c45253_g1,c9281_g1,c94111_g1,c26798_g1,c73623_g1,c50149_g2,c68374_g1,c50516_g1,c44104_g1,c49427_g1,c51114_g1 |
| Huntington's disease | ko05016 | 408 | c34735_g1,c57420_g1,c94563_g1,c37132_g1,c95194_g1,c76954_g1,c10168_g1,c95531_g1,c84193_g1,c17113_g1,c27141_g1,c60032_g1,c23083_g1,c75973_g1,c61064_g1,c3152_g1,c88181_g1,c105427_g1,c81902_g1,c46744_g1,c62196_g1,c16948_g1,c52413_g4,c9214_g1,c42776_g1,c37459_g1,c28591_g1,c49880_g1,c50327_g6,c97900_g1,c86894_g1,c84323_g1,c28486_g1,c35724_g1,c28409_g1,c99822_g1,c11910_g1,c19284_g1,c97317_g1,c61602_g1,c45734_g1,c94179_g1,c47785_g1,c108958_g1,c65926_g1,c36632_g1,c98483_g1,c108649_g1,c105682_g1,c55308_g1,c45289_g1,c28894_g1,c30615_g1,c106979_g1,c83916_g1,c75542_g1,c103930_g1,c53271_g1,c92603_g1,c105449_g1,c71762_g1,c48494_g1,c84587_g1,c82533_g1,c64920_g1,c11071_g1,c97623_g1,c12089_g1,c56181_g1,c11792_g1,c46405_g1,c31137_g1,c27333_g1,c88393_g1,c2113_g1,c82712_g1,c2296_g1,c62789_g1,c27245_g1,c44119_g1,c68666_g1,c91611_g1,c44822_g1,c17569_g1,c6498_g1,c11135_g1,c38201_g1,c11911_g1,c33339_g1,c1628_g1,c53271_g2,c21357_g1,c73214_g1,c61157_g1,c49376_g1,c68426_g1,c28261_g1,c25568_g1,c18647_g2,c65581_g1,c18990_g2,c78090_g1,c62118_g1,c69677_g1,c77072_g1,c39195_g1,c4249_g1,c110615_g1,c62495_g1,c24691_g1,c17949_g1,c41066_g1,c14090_g1,c93047_g1,c64608_g1,c108494_g1,c55792_g1,c16385_g1,c78300_g1,c5725_g1,c97305_g1,c50327_g5,c39227_g1,c30788_g1,c51278_g2,c36970_g1,c24765_g1,c51476_g1,c74445_g1,c46051_g1,c71754_g1,c5271_g1,c45910_g1,c40240_g1,c10937_g1,c61358_g1,c106417_g1,c87061_g1,c12071_g2,c109587_g1,c39385_g2,c83842_g1,c46040_g1,c67587_g1,c53040_g1,c90597_g1,c46069_g1,c14180_g1,c73920_g1,c108755_g1,c46944_g1,c94686_g1,c84521_g1,c37733_g1,c94922_g1,c24358_g1,c35699_g1,c26999_g1,c61765_g1,c6075_g1,c63743_g1,c53377_g1,c28562_g1,c61990_g1,c94865_g1,c46847_g1,c35383_g1,c60632_g1,c29366_g1,c88839_g1,c42167_g1,c73772_g1,c73069_g1,c41812_g1,c40326_g1,c51608_g2,c75573_g1,c96215_g1,c105364_g1,c18232_g1,c51687_g3,c48564_g1,c27328_g1,c72552_g1,c33826_g1,c103490_g1,c109560_g1,c24691_g2,c75246_g1,c71045_g1,c82733_g1,c10611_g1,c35073_g1,c31587_g1,c80156_g1,c93474_g1,c18014_g1,c12725_g1,c43157_g1,c29152_g1,c87592_g1,c25114_g1,c40254_g1,c20780_g1,c66315_g1,c86867_g1,c67087_g1,c31251_g1,c61778_g1,c27013_g1,c56646_g1,c65050_g1,c29538_g1,c94320_g1,c1091_g1,c24947_g1,c55393_g1,c92961_g1,c61622_g1,c28591_g2,c56780_g1,c107114_g1,c75828_g1,c84291_g1,c12872_g1,c26379_g1,c17210_g1,c106212_g1,c44890_g1,c18990_g1,c66264_g1,c27256_g1,c58019_g1,c54708_g1,c92807_g1,c95347_g1,c29868_g1,c57919_g1,c112501_g1,c52413_g3,c74003_g1,c108205_g1,c101824_g1,c62022_g1,c58011_g1,c38372_g1,c86260_g1,c89605_g1,c16695_g1,c51278_g3,c37339_g1,c107519_g1,c39227_g2,c53932_g1,c101459_g1,c10855_g1,c110399_g1,c95203_g1,c61340_g1,c108293_g1,c109205_g1,c42257_g1,c82103_g1,c78773_g1,c37192_g1,c102383_g1,c37949_g1,c42052_g1,c105411_g1,c10633_g1,c3998_g1,c86442_g1,c89657_g1,c2421_g1,c83387_g1,c71718_g1,c71787_g1,c49071_g1,c103267_g1,c93731_g1,c45151_g1,c46723_g1,c1141_g1,c17283_g1,c82348_g1,c48920_g1,c76687_g1,c57248_g1,c57755_g1,c44669_g1,c79539_g1,c18443_g1,c19567_g1,c66467_g1,c52382_g3,c98715_g1,c73196_g1,c38274_g1,c12821_g1,c61560_g1,c845_g1,c16834_g1,c97532_g1,c60503_g1,c91919_g1,c85012_g1,c28048_g1,c16610_g1,c97646_g1,c71873_g1,c55559_g1,c75767_g1,c16712_g1,c71632_g1,c78097_g1,c28543_g1,c74972_g1,c67598_g1,c107752_g1,c83293_g1,c97772_g1,c83089_g1,c83832_g1,c104037_g1,c54590_g1,c28419_g1,c15019_g1,c73802_g1,c104392_g1,c67688_g1,c77154_g1,c60888_g1,c4548_g1,c51687_g2,c22231_g1,c12814_g1,c51064_g1,c77864_g1,c105046_g1,c32556_g1,c95676_g1,c94053_g1,c34592_g1,c11160_g1,c34225_g1,c29016_g1,c51932_g6,c108546_g1,c104382_g1,c75744_g1,c790_g1,c11478_g1,c44434_g1,c95926_g1,c29145_g1,c52015_g1,c18232_g2,c86582_g1,c9150_g1,c39644_g1,c62331_g1,c29694_g1,c54622_g1,c1164_g1,c95449_g1,c91829_g1,c99310_g1,c56674_g1,c49231_g2,c51908_g1,c76908_g1,c56271_g1,c73806_g1,c61239_g1,c41037_g1,c78711_g1,c39385_g1,c49004_g10,c82205_g1,c68443_g1,c39644_g2,c18647_g1,c73323_g1,c104305_g1,c106734_g1,c40838_g1,c108219_g1,c80887_g1,c44924_g1,c88306_g1,c57606_g1,c27214_g1,c73672_g1,c13273_g1,c108352_g1,c82188_g1,c34164_g1,c31515_g1,c50149_g2,c30814_g1,c15911_g1,c68770_g1,c68374_g1,c28756_g1,c45920_g1,c4216_g1,c94974_g1,c96545_g1 |
| Hypertrophic cardiomyopathy (HCM) | ko05410 | 23 | c61071_g1,c42570_g1,c33552_g1,c103771_g1,c47524_g2,c49550_g1,c7955_g1,c50953_g1,c93738_g1,c44565_g1,c44174_g1,c47524_g3,c38500_g1,c39912_g1,c83486_g1,c16206_g1,c97640_g1,c30268_g1,c32760_g1,c32229_g1,c47524_g1,c41153_g1,c53757_g1 |
| Influenza A | ko05164 | 421 | c28479_g1,c104104_g1,c8503_g1,c35897_g1,c42058_g1,c42455_g1,c20428_g1,c31551_g1,c31284_g1,c31614_g1,c91578_g1,c48654_g1,c63236_g1,c49133_g1,c52047_g3,c81465_g1,c19656_g1,c62815_g1,c67256_g1,c85350_g1,c50516_g2,c36093_g1,c9338_g1,c50509_g1,c51362_g1,c46431_g1,c68166_g1,c51393_g2,c109082_g1,c41136_g1,c50433_g1,c31274_g1,c99965_g1,c87471_g1,c76045_g1,c14051_g1,c58723_g1,c41452_g1,c77468_g1,c68992_g1,c68830_g1,c84503_g1,c99939_g1,c67306_g1,c40971_g1,c20958_g1,c59703_g1,c108958_g1,c25682_g1,c51493_g1,c28106_g1,c65806_g1,c81287_g1,c50497_g1,c71995_g1,c46828_g2,c87121_g1,c30825_g1,c4653_g1,c55823_g1,c24811_g1,c44772_g1,c29500_g1,c59145_g1,c95765_g1,c51579_g1,c21101_g1,c43041_g1,c19257_g1,c93320_g1,c72649_g1,c105449_g1,c29983_g2,c43961_g1,c79325_g1,c5979_g1,c52309_g1,c48726_g1,c51393_g1,c84871_g1,c99424_g1,c73826_g1,c37435_g1,c90199_g1,c20517_g1,c96691_g1,c96066_g1,c106847_g1,c20072_g1,c37055_g1,c77647_g1,c52385_g2,c28859_g1,c49940_g3,c37866_g2,c43215_g1,c63084_g1,c99845_g1,c75818_g1,c89573_g1,c16333_g1,c103884_g1,c46828_g3,c64857_g1,c60132_g1,c30224_g1,c52687_g3,c27364_g1,c49555_g1,c88688_g1,c47248_g1,c97243_g1,c60036_g1,c76196_g1,c97805_g1,c73322_g1,c28878_g1,c75604_g1,c43552_g1,c42662_g1,c12545_g1,c51595_g1,c19670_g1,c46019_g1,c99189_g1,c69108_g1,c50706_g2,c104689_g1,c49045_g1,c106613_g1,c19117_g1,c101438_g1,c51250_g1,c85938_g1,c48076_g1,c85515_g1,c50542_g1,c75097_g1,c55047_g1,c91989_g1,c27003_g1,c27417_g1,c105342_g1,c98426_g1,c46894_g1,c22358_g1,c44883_g1,c43036_g1,c30663_g1,c5967_g1,c75980_g1,c89522_g1,c12183_g1,c27559_g1,c45585_g1,c50354_g1,c45587_g1,c71422_g1,c107740_g1,c36117_g1,c51087_g1,c52810_g1,c44213_g2,c63994_g1,c59458_g1,c6075_g1,c56011_g1,c52221_g2,c97101_g1,c35128_g1,c25032_g1,c31283_g1,c99321_g1,c35383_g1,c41811_g1,c93658_g1,c38149_g1,c43513_g1,c63402_g1,c74219_g1,c31287_g1,c49457_g4,c65797_g1,c25651_g1,c51687_g3,c56501_g1,c2927_g1,c48306_g1,c51933_g1,c31483_g1,c20191_g1,c45078_g1,c11780_g1,c50217_g1,c71045_g1,c43622_g1,c32310_g2,c31587_g1,c48112_g2,c99332_g1,c42033_g1,c92599_g1,c35585_g2,c37345_g1,c33344_g1,c103510_g1,c109598_g1,c39201_g1,c39157_g2,c48245_g1,c52206_g3,c38021_g1,c65057_g1,c34104_g1,c36833_g1,c26902_g1,c7457_g1,c53340_g1,c49999_g4,c52084_g5,c102213_g1,c48988_g3,c11267_g1,c48713_g2,c47048_g2,c103460_g1,c54833_g1,c46096_g1,c34581_g1,c41349_g1,c23873_g1,c40803_g1,c48899_g1,c96576_g1,c63003_g1,c38428_g2,c108116_g1,c35585_g1,c97045_g1,c25369_g1,c13250_g1,c44805_g1,c52646_g1,c42711_g1,c18161_g1,c10943_g1,c16958_g1,c65482_g1,c77087_g1,c45556_g1,c63919_g1,c84103_g1,c55120_g1,c39145_g2,c51243_g7,c88796_g1,c48972_g1,c20000_g1,c48288_g1,c61555_g1,c104810_g1,c57458_g1,c58309_g1,c45137_g1,c38091_g2,c41208_g1,c77837_g1,c84260_g1,c106258_g1,c35140_g1,c103497_g1,c102073_g1,c20246_g1,c39725_g1,c25026_g1,c51314_g5,c18935_g1,c48833_g1,c105070_g1,c34931_g1,c11124_g1,c35002_g1,c40640_g1,c36058_g1,c39243_g1,c51009_g1,c62353_g1,c23872_g1,c46828_g1,c46394_g2,c26562_g1,c97184_g1,c12015_g1,c45152_g1,c17204_g1,c72172_g1,c1970_g1,c65565_g1,c44286_g1,c109295_g1,c48337_g1,c45705_g1,c40297_g1,c50480_g1,c71256_g1,c17979_g1,c40051_g1,c17204_g2,c79424_g1,c43106_g1,c39874_g1,c16587_g1,c50764_g1,c87049_g1,c45705_g2,c65754_g1,c29193_g1,c71808_g1,c62342_g1,c45941_g3,c48425_g1,c41500_g1,c38710_g1,c16834_g1,c96255_g1,c63746_g1,c34941_g2,c53988_g1,c34711_g1,c44980_g1,c112428_g1,c49276_g4,c92119_g1,c55559_g1,c46059_g2,c47535_g2,c52687_g2,c63601_g1,c99231_g1,c32428_g1,c17193_g1,c74294_g1,c63478_g1,c2959_g1,c32310_g1,c13172_g1,c40516_g1,c36767_g1,c105218_g1,c109031_g1,c110032_g1,c51687_g2,c51379_g1,c31527_g1,c52047_g1,c84912_g1,c11160_g1,c4455_g1,c14738_g1,c48723_g1,c50422_g6,c70565_g1,c34941_g1,c85278_g1,c27075_g1,c55706_g1,c50171_g1,c5896_g1,c88625_g1,c41879_g1,c72114_g1,c33668_g1,c44897_g1,c17140_g1,c15748_g1,c99310_g1,c32004_g1,c41525_g1,c27875_g1,c108348_g1,c61603_g1,c43036_g2,c34370_g1,c35801_g1,c52773_g1,c29143_g1,c37602_g1,c73371_g1,c48569_g1,c13411_g1,c56533_g1,c27176_g1,c5603_g1,c52449_g2,c57174_g1,c107967_g1,c52687_g4,c54042_g1,c110498_g1,c107618_g1,c10126_g1,c62984_g1,c49158_g1,c74430_g1,c39145_g1,c19301_g1,c47048_g1,c41963_g1,c102593_g1,c52975_g1,c31673_g1,c38805_g1,c48713_g1,c34410_g1,c33820_g1,c59828_g1,c50516_g1,c607_g1,c86378_g1,c93659_g1,c96545_g1 |
| Legionellosis | ko05134 | 216 | c13238_g1,c30767_g1,c56814_g1,c30046_g1,c26011_g1,c91578_g1,c41134_g1,c110633_g1,c81465_g1,c5381_g1,c6040_g1,c87471_g1,c82163_g1,c97107_g1,c68830_g1,c40971_g1,c94546_g1,c108958_g1,c29979_g1,c65806_g1,c50317_g5,c46828_g2,c105045_g1,c95765_g1,c105449_g1,c38530_g1,c19306_g1,c51840_g8,c5979_g1,c25101_g1,c84871_g1,c103445_g1,c99424_g1,c90199_g1,c73840_g1,c96066_g1,c24701_g3,c68525_g1,c37866_g2,c63084_g1,c104026_g1,c46828_g3,c1291_g1,c111368_g1,c60132_g1,c30224_g1,c88688_g1,c47248_g1,c33971_g2,c76196_g1,c97805_g1,c43972_g1,c73322_g1,c107522_g1,c75604_g1,c97513_g1,c50317_g4,c28040_g1,c17231_g1,c67603_g1,c20451_g1,c104689_g1,c19117_g1,c36573_g1,c1039_g1,c27003_g1,c102648_g1,c98426_g1,c43036_g1,c75980_g1,c33706_g1,c54573_g1,c83541_g1,c71422_g1,c36117_g1,c59458_g1,c28034_g1,c50317_g2,c65384_g1,c6075_g1,c42911_g1,c107497_g1,c52228_g1,c96174_g1,c97101_g1,c46095_g1,c99321_g1,c55960_g1,c69639_g1,c98157_g1,c15959_g1,c65797_g1,c55300_g1,c43551_g1,c101865_g1,c50317_g3,c48306_g1,c20191_g1,c71045_g1,c28392_g1,c65958_g1,c37672_g1,c32310_g2,c31587_g1,c42033_g1,c92599_g1,c39157_g2,c65057_g1,c7457_g1,c83750_g1,c108127_g1,c103460_g1,c54833_g1,c34581_g1,c23873_g1,c96576_g1,c63003_g1,c49758_g1,c38910_g2,c2722_g1,c14355_g1,c46095_g2,c94623_g1,c10943_g1,c18552_g1,c63919_g1,c90874_g1,c43332_g1,c66013_g1,c88796_g1,c104810_g1,c56715_g1,c70527_g1,c50900_g1,c93792_g1,c104497_g1,c103497_g1,c102073_g1,c18935_g1,c105070_g1,c36058_g1,c62353_g1,c46828_g1,c88753_g1,c76939_g1,c6247_g1,c82065_g1,c47917_g1,c66085_g1,c87049_g1,c95297_g1,c65754_g1,c106274_g1,c51760_g1,c62342_g1,c38910_g1,c16834_g1,c76967_g1,c34941_g2,c24701_g2,c43308_g1,c46095_g3,c55559_g1,c64196_g1,c99231_g1,c26951_g1,c17193_g1,c33571_g1,c32310_g1,c24701_g1,c13172_g1,c40516_g1,c67091_g1,c109031_g1,c106932_g1,c64428_g1,c49243_g1,c18568_g1,c42117_g1,c76643_g1,c11160_g1,c4455_g1,c107255_g1,c57096_g1,c55108_g1,c103453_g1,c34941_g1,c19107_g1,c55706_g1,c86730_g1,c104287_g1,c35177_g1,c43036_g2,c3953_g1,c688_g1,c23544_g1,c13411_g1,c56533_g1,c76334_g1,c57174_g1,c110498_g1,c107618_g1,c107399_g1,c50173_g1,c11645_g1,c74430_g1,c17982_g1,c19301_g1,c52651_g1,c85883_g1,c102593_g1,c33571_g2,c60836_g1,c96545_g1,c16677_g1,c3699_g1 |
| Leishmaniasis | ko05140 | 293 | c28479_g1,c104104_g1,c82662_g1,c46587_g1,c8503_g1,c35897_g1,c42058_g1,c42455_g1,c20428_g1,c31551_g1,c31284_g1,c48654_g1,c63236_g1,c49133_g1,c90349_g1,c52047_g3,c19656_g1,c67256_g1,c85350_g1,c64798_g1,c36093_g1,c9338_g1,c50509_g1,c51362_g1,c46431_g1,c68166_g1,c51393_g2,c109082_g1,c41136_g1,c50433_g1,c76045_g1,c41452_g1,c77468_g1,c48850_g1,c68992_g1,c99939_g1,c67306_g1,c20958_g1,c59703_g1,c70554_g1,c25682_g1,c51493_g1,c28106_g1,c50497_g1,c87121_g1,c30825_g1,c4653_g1,c55823_g1,c24811_g1,c44772_g1,c29500_g1,c59145_g1,c51579_g1,c21101_g1,c43041_g1,c19257_g1,c93320_g1,c72649_g1,c29983_g2,c43961_g1,c52309_g1,c48726_g1,c51393_g1,c73826_g1,c20517_g1,c106847_g1,c20072_g1,c37055_g1,c77647_g1,c52385_g2,c49940_g3,c43215_g1,c99845_g1,c89573_g1,c16333_g1,c103884_g1,c64857_g1,c24563_g1,c52687_g3,c27364_g1,c49555_g1,c97243_g1,c60036_g1,c44871_g1,c28878_g1,c43552_g1,c42662_g1,c12545_g1,c19670_g1,c46019_g1,c99189_g1,c69108_g1,c50706_g2,c49045_g1,c106613_g1,c51250_g1,c85938_g1,c48076_g1,c85515_g1,c50542_g1,c75097_g1,c27417_g1,c105342_g1,c46894_g1,c74335_g1,c30663_g1,c5967_g1,c89522_g1,c12183_g1,c27559_g1,c45585_g1,c31723_g1,c50354_g1,c36550_g1,c51087_g1,c52810_g1,c44213_g2,c63994_g1,c102241_g1,c56011_g1,c52221_g2,c35128_g1,c25032_g1,c31283_g1,c41811_g1,c93658_g1,c38149_g1,c63402_g1,c74219_g1,c31287_g1,c73710_g1,c49457_g4,c33637_g1,c25651_g1,c2927_g1,c51933_g1,c45078_g1,c11780_g1,c50217_g1,c43622_g1,c48112_g2,c99332_g1,c35585_g2,c37345_g1,c39201_g1,c48245_g1,c52206_g3,c38021_g1,c34104_g1,c36833_g1,c26902_g1,c49999_g4,c52084_g5,c45277_g1,c48988_g3,c11267_g1,c48713_g2,c47048_g2,c46096_g1,c40803_g1,c38428_g2,c108116_g1,c35585_g1,c97045_g1,c25369_g1,c13250_g1,c44805_g1,c52646_g1,c42711_g1,c16958_g1,c65482_g1,c77087_g1,c45556_g1,c84103_g1,c55120_g1,c51243_g7,c48972_g1,c20000_g1,c48288_g1,c61555_g1,c57458_g1,c58309_g1,c45137_g1,c38091_g2,c41208_g1,c77837_g1,c106258_g1,c35140_g1,c20246_g1,c39725_g1,c25026_g1,c51314_g5,c48833_g1,c106429_g1,c34931_g1,c35002_g1,c40640_g1,c77655_g1,c39243_g1,c51009_g1,c23872_g1,c46394_g2,c26562_g1,c97184_g1,c12015_g1,c45152_g1,c17204_g1,c1970_g1,c95560_g1,c109295_g1,c48337_g1,c45705_g1,c40297_g1,c50480_g1,c71256_g1,c17979_g1,c40051_g1,c17204_g2,c79424_g1,c43106_g1,c39874_g1,c16587_g1,c45705_g2,c29193_g1,c75439_g1,c71808_g1,c48425_g1,c41500_g1,c38710_g1,c96255_g1,c53988_g1,c44980_g1,c49276_g4,c92119_g1,c46059_g2,c47535_g2,c52687_g2,c63601_g1,c32428_g1,c74294_g1,c63478_g1,c2959_g1,c36767_g1,c110032_g1,c51379_g1,c31527_g1,c52047_g1,c84912_g1,c14738_g1,c48723_g1,c50422_g6,c70565_g1,c85278_g1,c27075_g1,c50171_g1,c5896_g1,c88625_g1,c41879_g1,c72114_g1,c12186_g1,c33668_g1,c44897_g1,c22716_g1,c15748_g1,c32004_g1,c27875_g1,c108348_g1,c61603_g1,c34370_g1,c107634_g1,c52773_g1,c29143_g1,c37602_g1,c48569_g1,c5603_g1,c52449_g2,c52687_g4,c54042_g1,c10126_g1,c62984_g1,c49158_g1,c44871_g2,c47048_g1,c41963_g1,c31673_g1,c38805_g1,c48713_g1,c34410_g1,c33820_g1,c59828_g1,c45364_g1,c607_g1,c86378_g1 |
| Measles | ko05162 | 432 | c28479_g1,c104104_g1,c82662_g1,c8503_g1,c35897_g1,c42058_g1,c803_g1,c42455_g1,c20428_g1,c31551_g1,c31284_g1,c31614_g1,c91578_g1,c48654_g1,c63236_g1,c49133_g1,c90349_g1,c52047_g3,c75900_g1,c81465_g1,c19656_g1,c67256_g1,c85350_g1,c64798_g1,c36093_g1,c9338_g1,c50509_g1,c111162_g1,c51362_g1,c46431_g1,c68166_g1,c51393_g2,c109082_g1,c41136_g1,c50433_g1,c87471_g1,c76045_g1,c41452_g1,c77468_g1,c48850_g1,c68992_g1,c68830_g1,c84503_g1,c99939_g1,c67306_g1,c40971_g1,c20958_g1,c59703_g1,c70554_g1,c25682_g1,c51493_g1,c98724_g1,c28106_g1,c65806_g1,c50497_g1,c46828_g2,c74540_g1,c87121_g1,c30825_g1,c4653_g1,c55823_g1,c24811_g1,c44772_g1,c29500_g1,c59145_g1,c95765_g1,c51579_g1,c21101_g1,c43041_g1,c19257_g1,c84030_g1,c93320_g1,c72649_g1,c29983_g2,c43961_g1,c5979_g1,c52309_g1,c48726_g1,c51393_g1,c84871_g1,c99424_g1,c7300_g1,c73826_g1,c90199_g1,c20517_g1,c96691_g1,c96066_g1,c106847_g1,c20072_g1,c37055_g1,c77647_g1,c52385_g2,c28859_g1,c49940_g3,c37866_g2,c61369_g1,c43215_g1,c63084_g1,c99845_g1,c89573_g1,c16333_g1,c103884_g1,c46828_g3,c64857_g1,c24563_g1,c60132_g1,c30224_g1,c52687_g3,c27364_g1,c49555_g1,c88688_g1,c47248_g1,c97243_g1,c60036_g1,c76196_g1,c97805_g1,c44871_g1,c73322_g1,c28878_g1,c75604_g1,c43552_g1,c42662_g1,c107699_g1,c111102_g1,c12545_g1,c51595_g1,c19670_g1,c46019_g1,c99189_g1,c69108_g1,c50706_g2,c104689_g1,c62599_g1,c49045_g1,c106613_g1,c19117_g1,c44491_g1,c51250_g1,c85938_g1,c48076_g1,c85515_g1,c50542_g1,c30287_g1,c27003_g1,c14311_g1,c27417_g1,c105342_g1,c98426_g1,c46894_g1,c22358_g1,c74335_g1,c43036_g1,c30663_g1,c40424_g1,c5967_g1,c75980_g1,c89522_g1,c12183_g1,c27559_g1,c45585_g1,c31723_g1,c50354_g1,c36550_g1,c43798_g1,c71422_g1,c36117_g1,c51087_g1,c52810_g1,c44213_g2,c63994_g1,c59458_g1,c42638_g1,c102241_g1,c84894_g1,c56011_g1,c52221_g2,c97101_g1,c35128_g1,c25032_g1,c31283_g1,c99321_g1,c41811_g1,c93658_g1,c38149_g1,c63402_g1,c47007_g1,c74219_g1,c31287_g1,c73710_g1,c49457_g4,c33637_g1,c65797_g1,c25651_g1,c28865_g1,c56501_g1,c2927_g1,c48306_g1,c51933_g1,c20191_g1,c45078_g1,c11780_g1,c84326_g1,c50217_g1,c43622_g1,c52977_g1,c32310_g2,c48112_g2,c99332_g1,c42033_g1,c92599_g1,c35585_g2,c37345_g1,c39201_g1,c39157_g2,c48245_g1,c33209_g1,c52206_g3,c38021_g1,c65057_g1,c34104_g1,c36833_g1,c45042_g1,c26902_g1,c7457_g1,c49999_g4,c52084_g5,c45277_g1,c102213_g1,c48988_g3,c11267_g1,c48713_g2,c47048_g2,c103460_g1,c54833_g1,c46096_g1,c34581_g1,c41349_g1,c23873_g1,c40803_g1,c96576_g1,c63003_g1,c38428_g2,c108116_g1,c35585_g1,c97045_g1,c25369_g1,c13250_g1,c44805_g1,c52646_g1,c75676_g1,c42711_g1,c18161_g1,c10943_g1,c16958_g1,c82890_g1,c65482_g1,c77087_g1,c45556_g1,c63919_g1,c84103_g1,c55120_g1,c75958_g1,c39145_g2,c51243_g7,c88796_g1,c48972_g1,c20000_g1,c48288_g1,c61555_g1,c104810_g1,c57458_g1,c58309_g1,c45137_g1,c38091_g2,c41208_g1,c77837_g1,c106258_g1,c35140_g1,c103497_g1,c102073_g1,c20246_g1,c39725_g1,c25026_g1,c51314_g5,c18935_g1,c48833_g1,c105070_g1,c106429_g1,c34931_g1,c11178_g1,c35002_g1,c40640_g1,c77655_g1,c36058_g1,c39243_g1,c51009_g1,c62353_g1,c23872_g1,c46828_g1,c46394_g2,c26562_g1,c97184_g1,c12015_g1,c45152_g1,c17204_g1,c1970_g1,c95560_g1,c44286_g1,c109295_g1,c48337_g1,c45705_g1,c40297_g1,c50480_g1,c71256_g1,c17979_g1,c17204_g2,c79424_g1,c43106_g1,c61821_g1,c39874_g1,c16587_g1,c42378_g1,c87049_g1,c45705_g2,c65754_g1,c29193_g1,c75439_g1,c75375_g1,c71808_g1,c62342_g1,c48425_g1,c41500_g1,c38710_g1,c96255_g1,c34941_g2,c53988_g1,c44980_g1,c49276_g4,c92119_g1,c62976_g1,c46059_g2,c47535_g2,c52687_g2,c63601_g1,c99231_g1,c32428_g1,c17193_g1,c74294_g1,c63478_g1,c2959_g1,c32310_g1,c13172_g1,c40516_g1,c36767_g1,c56627_g1,c109031_g1,c110032_g1,c51379_g1,c31527_g1,c61935_g1,c52047_g1,c82617_g1,c84912_g1,c4455_g1,c14738_g1,c48723_g1,c50422_g6,c70565_g1,c34941_g1,c85278_g1,c27075_g1,c55706_g1,c50171_g1,c5896_g1,c88625_g1,c41879_g1,c72114_g1,c12186_g1,c33668_g1,c72040_g1,c44897_g1,c22716_g1,c15748_g1,c63562_g1,c32004_g1,c73286_g1,c27875_g1,c108348_g1,c61603_g1,c43036_g2,c94065_g1,c34370_g1,c107634_g1,c52773_g1,c29143_g1,c37602_g1,c48569_g1,c13411_g1,c56533_g1,c27176_g1,c5603_g1,c52449_g2,c57174_g1,c52687_g4,c54042_g1,c110498_g1,c107618_g1,c41813_g1,c10126_g1,c62984_g1,c49158_g1,c74430_g1,c44871_g2,c39145_g1,c19301_g1,c95993_g1,c47048_g1,c41963_g1,c100017_g1,c102593_g1,c31673_g1,c38805_g1,c65684_g1,c48713_g1,c34410_g1,c43448_g1,c33820_g1,c59828_g1,c45364_g1,c607_g1,c86378_g1,c88514_g1 |
| Melanoma | ko05218 | 15 | c19496_g1,c75818_g1,c75097_g1,c38471_g1,c45587_g1,c17647_g1,c72535_g1,c64363_g1,c65565_g1,c40051_g1,c46989_g1,c79435_g1,c48724_g1,c45253_g1,c64976_g1 |
| MicroRNAs in cancer | ko05206 | 101 | c30967_g1,c51676_g1,c35418_g1,c23574_g1,c50437_g2,c49070_g1,c52836_g1,c81516_g1,c47072_g1,c74393_g1,c49911_g1,c37057_g1,c83052_g1,c52736_g1,c50437_g1,c76639_g1,c19496_g1,c104832_g1,c77918_g1,c63694_g1,c80570_g1,c75818_g1,c15664_g1,c52836_g3,c27421_g1,c103721_g1,c49662_g1,c107405_g1,c38471_g1,c52270_g1,c106447_g1,c47427_g1,c84185_g1,c71536_g1,c45587_g1,c109512_g1,c17647_g1,c53284_g1,c67135_g1,c46513_g1,c65387_g1,c84121_g1,c51776_g1,c52280_g1,c35383_g1,c83170_g1,c48946_g1,c64142_g1,c51687_g3,c25215_g1,c46967_g1,c79179_g1,c45785_g1,c52472_g1,c99619_g1,c109396_g1,c51124_g1,c5719_g2,c107734_g1,c82892_g1,c105420_g1,c58762_g1,c103046_g1,c83460_g1,c40527_g1,c44657_g1,c75607_g1,c52205_g2,c40688_g1,c58739_g1,c63217_g1,c50000_g1,c83539_g1,c107454_g1,c64363_g1,c65565_g1,c50342_g1,c52720_g2,c105731_g1,c52199_g2,c54126_g1,c88326_g1,c80963_g1,c13931_g1,c63610_g1,c64617_g1,c47001_g1,c51124_g2,c51687_g2,c51118_g1,c99310_g1,c64272_g1,c51711_g1,c79435_g1,c48724_g1,c43531_g1,c45253_g1,c10652_g1,c92839_g1,c59736_g1,c64976_g1 |
| Morphine addiction | ko05032 | 17 | c24927_g1,c63330_g1,c13965_g1,c41212_g1,c94513_g1,c84183_g1,c45589_g3,c28114_g2,c11484_g1,c28114_g1,c97112_g1,c99632_g1,c63116_g1,c105077_g1,c105908_g1,c41258_g1,c10282_g1 |
| Nicotine addiction | ko05033 | 9 | c63330_g1,c13965_g1,c41212_g1,c94513_g1,c45589_g3,c28114_g2,c28114_g1,c105908_g1,c41258_g1 |
| Non-alcoholic fatty liver disease (NAFLD) | ko04932 | 271 | c34735_g1,c61071_g1,c52710_g2,c42570_g1,c95194_g1,c76954_g1,c10168_g1,c31614_g1,c17113_g1,c60032_g1,c23083_g1,c75973_g1,c3152_g1,c105427_g1,c4329_g1,c81902_g1,c16948_g1,c9214_g1,c37459_g1,c28591_g1,c97900_g1,c86894_g1,c84323_g1,c28409_g1,c99822_g1,c11910_g1,c97317_g1,c84503_g1,c61602_g1,c108958_g1,c33552_g1,c65926_g1,c52710_g3,c105682_g1,c55308_g1,c48247_g2,c43436_g1,c28894_g1,c30615_g1,c83916_g1,c85476_g1,c75542_g1,c103930_g1,c53271_g1,c54485_g1,c92603_g1,c105449_g1,c85837_g1,c64920_g1,c11792_g1,c40995_g1,c96691_g1,c31137_g1,c88393_g1,c103771_g1,c2296_g1,c62789_g1,c42048_g1,c47524_g2,c61171_g1,c68666_g1,c49550_g1,c1628_g1,c53271_g2,c61157_g1,c68426_g1,c28261_g1,c18647_g2,c93546_g1,c65581_g1,c78090_g1,c51595_g1,c7955_g1,c24691_g1,c41066_g1,c93047_g1,c64608_g1,c52377_g3,c40208_g1,c50953_g1,c5725_g1,c30788_g1,c51278_g2,c36970_g1,c24765_g1,c51476_g1,c74445_g1,c46051_g1,c71754_g1,c40240_g1,c10937_g1,c12071_g2,c109587_g1,c39385_g2,c67587_g1,c53040_g1,c46069_g1,c14180_g1,c46944_g1,c7076_g1,c93365_g1,c55924_g1,c27920_g1,c39066_g1,c6075_g1,c61990_g1,c93738_g1,c60632_g1,c71662_g1,c73772_g1,c41812_g1,c75573_g1,c96215_g1,c56501_g1,c27328_g1,c33826_g1,c103490_g1,c24691_g2,c30868_g1,c53443_g1,c71045_g1,c44565_g1,c10611_g1,c35073_g1,c31587_g1,c80156_g1,c77336_g1,c44174_g1,c29152_g1,c87592_g1,c40254_g1,c20780_g1,c86867_g1,c27013_g1,c47524_g3,c38500_g1,c29538_g1,c55393_g1,c102213_g1,c92961_g1,c61622_g1,c28591_g2,c38253_g1,c107114_g1,c39912_g1,c41349_g1,c75828_g1,c72326_g1,c84291_g1,c12872_g1,c17210_g1,c106212_g1,c66264_g1,c18161_g1,c54708_g1,c29868_g1,c112501_g1,c39145_g2,c101824_g1,c62022_g1,c37689_g1,c86260_g1,c89605_g1,c16695_g1,c51278_g3,c107519_g1,c53932_g1,c101459_g1,c10855_g1,c110399_g1,c83486_g1,c87271_g1,c37192_g1,c102383_g1,c105411_g1,c10633_g1,c3998_g1,c86442_g1,c89657_g1,c2421_g1,c71718_g1,c71787_g1,c103267_g1,c16206_g1,c54118_g1,c1141_g1,c34368_g1,c17283_g1,c48920_g1,c79539_g1,c18443_g1,c19567_g1,c66467_g1,c97640_g1,c52382_g3,c73196_g1,c38274_g1,c61560_g1,c16834_g1,c85012_g1,c106851_g1,c97646_g1,c71873_g1,c55559_g1,c75767_g1,c71632_g1,c28543_g1,c74972_g1,c107752_g1,c97772_g1,c104037_g1,c15019_g1,c73802_g1,c104392_g1,c37697_g1,c4548_g1,c30268_g1,c32760_g1,c32229_g1,c12814_g1,c32556_g1,c95676_g1,c94053_g1,c34812_g1,c78655_g1,c11160_g1,c34225_g1,c51932_g6,c108546_g1,c104382_g1,c11478_g1,c95446_g1,c83855_g1,c52015_g1,c86582_g1,c9150_g1,c39644_g1,c29694_g1,c1164_g1,c56674_g1,c76908_g1,c73806_g1,c47524_g1,c28281_g1,c59728_g1,c39385_g1,c27176_g1,c103367_g1,c82205_g1,c39644_g2,c18647_g1,c39145_g1,c73323_g1,c41153_g1,c106734_g1,c108219_g1,c80887_g1,c88306_g1,c57606_g1,c42608_g1,c26895_g1,c32374_g2,c82188_g1,c34164_g1,c46852_g1,c30814_g1,c68770_g1,c29335_g1,c45920_g1,c96545_g1,c53757_g1 |
| Non-small cell lung cancer | ko05223 | 14 | c19496_g1,c75818_g1,c75097_g1,c45587_g1,c53373_g1,c72535_g1,c35517_g1,c40669_g1,c65565_g1,c40051_g1,c46989_g1,c65911_g1,c79435_g1,c45253_g1 |
| Pancreatic cancer | ko05212 | 36 | c4329_g1,c48247_g2,c85476_g1,c54485_g1,c19496_g1,c75818_g1,c61171_g1,c93546_g1,c75097_g1,c45587_g1,c7076_g1,c93365_g1,c55924_g1,c27920_g1,c71662_g1,c52597_g1,c30868_g1,c72535_g1,c77336_g1,c38253_g1,c72326_g1,c37689_g1,c65565_g1,c34368_g1,c40051_g1,c37697_g1,c78655_g1,c95446_g1,c83855_g1,c46989_g1,c19158_g1,c28281_g1,c79435_g1,c103367_g1,c45253_g1,c26895_g1 |
| Parkinson's disease | ko05012 | 358 | c34735_g1,c37132_g1,c95194_g1,c76954_g1,c10168_g1,c84193_g1,c17113_g1,c27141_g1,c60032_g1,c23083_g1,c75973_g1,c105321_g1,c61064_g1,c3152_g1,c88181_g1,c105427_g1,c86494_g1,c81902_g1,c46744_g1,c35059_g1,c62196_g1,c94748_g1,c16948_g1,c9214_g1,c42776_g1,c37459_g1,c28591_g1,c51453_g8,c97900_g1,c86894_g1,c84323_g1,c28409_g1,c99822_g1,c11910_g1,c97317_g1,c61602_g1,c52218_g2,c108958_g1,c65926_g1,c36632_g1,c98483_g1,c105682_g1,c55308_g1,c28894_g1,c30615_g1,c106979_g1,c83916_g1,c75542_g1,c103930_g1,c53271_g1,c38747_g1,c92603_g1,c105449_g1,c98089_g1,c48494_g1,c84587_g1,c64920_g1,c12089_g1,c56181_g1,c11792_g1,c46405_g1,c31137_g1,c27333_g1,c88393_g1,c2113_g1,c2296_g1,c62789_g1,c49423_g2,c44119_g1,c68666_g1,c91611_g1,c44822_g1,c17569_g1,c6498_g1,c11135_g1,c11911_g1,c1628_g1,c53271_g2,c21357_g1,c6086_g1,c61157_g1,c49376_g1,c68426_g1,c28261_g1,c57137_g1,c78985_g1,c18647_g2,c65581_g1,c18990_g2,c61656_g1,c78090_g1,c62118_g1,c69677_g1,c77072_g1,c4249_g1,c110615_g1,c24691_g1,c17949_g1,c41066_g1,c14090_g1,c93047_g1,c101000_g1,c64608_g1,c16385_g1,c78300_g1,c5725_g1,c97305_g1,c63324_g1,c39227_g1,c30788_g1,c51278_g2,c36970_g1,c24765_g1,c52382_g6,c51476_g1,c74445_g1,c46051_g1,c71754_g1,c5271_g1,c45910_g1,c40240_g1,c10937_g1,c61358_g1,c31127_g1,c107724_g1,c106417_g1,c87061_g1,c12071_g2,c109587_g1,c39385_g2,c52382_g1,c46040_g1,c67587_g1,c72555_g1,c53040_g1,c90597_g1,c57404_g1,c41754_g1,c46069_g1,c14180_g1,c108755_g1,c46944_g1,c6075_g1,c61909_g1,c53377_g1,c28562_g1,c53622_g1,c61990_g1,c94865_g1,c46847_g1,c60632_g1,c29366_g1,c88839_g1,c42167_g1,c73772_g1,c73069_g1,c41812_g1,c40326_g1,c51608_g2,c75573_g1,c52218_g3,c96215_g1,c105318_g1,c18232_g1,c82203_g1,c27328_g1,c79468_g1,c72305_g1,c72552_g1,c33826_g1,c103490_g1,c109560_g1,c24691_g2,c75246_g1,c71045_g1,c10611_g1,c35073_g1,c31587_g1,c80156_g1,c18014_g1,c12725_g1,c43157_g1,c29152_g1,c87592_g1,c25114_g1,c40254_g1,c20780_g1,c66315_g1,c86867_g1,c54734_g1,c27013_g1,c98456_g1,c56646_g1,c29538_g1,c94320_g1,c66057_g1,c1091_g1,c24947_g1,c58824_g1,c55393_g1,c92961_g1,c61622_g1,c28591_g2,c107114_g1,c95365_g1,c75828_g1,c84291_g1,c12872_g1,c17210_g1,c106212_g1,c52317_g5,c18990_g1,c66264_g1,c62084_g1,c54701_g1,c54708_g1,c92807_g1,c29868_g1,c112501_g1,c49338_g2,c74003_g1,c101824_g1,c62022_g1,c58011_g1,c86260_g1,c89605_g1,c16695_g1,c51278_g3,c107519_g1,c39227_g2,c72316_g1,c53932_g1,c101459_g1,c10855_g1,c31199_g1,c52382_g5,c110399_g1,c95203_g1,c108293_g1,c37192_g1,c102383_g1,c37949_g1,c42052_g1,c105411_g1,c10633_g1,c106377_g1,c3998_g1,c86442_g1,c17294_g1,c89657_g1,c2421_g1,c71718_g1,c76089_g1,c57022_g1,c71787_g1,c103267_g1,c71912_g1,c45151_g1,c46723_g1,c1141_g1,c87513_g1,c17283_g1,c48920_g1,c57248_g1,c57755_g1,c44669_g1,c79539_g1,c18443_g1,c19567_g1,c66467_g1,c52382_g3,c73196_g1,c38274_g1,c61560_g1,c16834_g1,c97532_g1,c52908_g1,c85012_g1,c97646_g1,c71873_g1,c55559_g1,c75767_g1,c71632_g1,c78097_g1,c28543_g1,c74972_g1,c67598_g1,c107752_g1,c83293_g1,c86414_g1,c97772_g1,c83089_g1,c104037_g1,c15019_g1,c73802_g1,c104392_g1,c67688_g1,c77154_g1,c60888_g1,c4548_g1,c93349_g1,c22231_g1,c12814_g1,c32556_g1,c95676_g1,c94053_g1,c6502_g1,c11160_g1,c34225_g1,c51932_g6,c108546_g1,c104382_g1,c11478_g1,c44434_g1,c29145_g1,c52015_g1,c18232_g2,c86582_g1,c9150_g1,c39644_g1,c62331_g1,c29694_g1,c1164_g1,c95449_g1,c91829_g1,c56674_g1,c76908_g1,c56271_g1,c73806_g1,c78711_g1,c39385_g1,c72030_g1,c82205_g1,c68443_g1,c100608_g1,c51453_g3,c39644_g2,c18647_g1,c73323_g1,c106734_g1,c82717_g1,c108219_g1,c80887_g1,c44924_g1,c88306_g1,c57606_g1,c73672_g1,c108352_g1,c82188_g1,c4027_g1,c34164_g1,c16903_g1,c30814_g1,c15911_g1,c68770_g1,c68374_g1,c45920_g1,c96545_g1 |
| Pathogenic Escherichia coli infection | ko05130 | 89 | c49789_g3,c104533_g1,c85479_g1,c73933_g1,c31798_g1,c27491_g1,c71974_g1,c110717_g1,c50778_g1,c44759_g1,c43208_g1,c61362_g1,c23517_g2,c89389_g1,c78193_g1,c4467_g1,c22729_g1,c98214_g1,c49571_g1,c37426_g1,c17874_g1,c17409_g1,c33691_g2,c112121_g1,c43718_g1,c45944_g1,c11193_g1,c18485_g1,c108021_g1,c97609_g1,c94760_g1,c98667_g1,c88318_g1,c43574_g1,c102600_g1,c82433_g1,c73389_g1,c42374_g1,c7716_g1,c27820_g1,c40269_g1,c6234_g1,c49571_g2,c79310_g1,c34934_g1,c43403_g1,c39151_g1,c35091_g1,c81767_g1,c72833_g1,c103873_g1,c59200_g1,c43754_g2,c61240_g1,c63592_g1,c23367_g1,c39968_g1,c50515_g2,c99303_g1,c16097_g1,c83648_g1,c78021_g1,c48318_g1,c74201_g1,c40724_g1,c75936_g1,c83171_g1,c49991_g2,c13025_g1,c82318_g1,c109250_g1,c39484_g1,c43718_g2,c90700_g1,c29109_g1,c588_g1,c87945_g1,c45108_g2,c30988_g1,c98002_g1,c93072_g1,c103686_g1,c43754_g1,c70598_g1,c109715_g1,c57541_g1,c23517_g1,c33691_g1,c34655_g1 |
| Pathways in cancer | ko05200 | 176 | c803_g1,c8275_g1,c31614_g1,c29237_g1,c4329_g1,c52413_g4,c9460_g1,c64341_g1,c111162_g1,c60514_g1,c49070_g1,c81516_g1,c19284_g1,c108958_g1,c30744_g1,c68079_g1,c76236_g1,c48247_g2,c21454_g1,c65074_g1,c85476_g1,c54485_g1,c84030_g1,c35896_g1,c105449_g1,c88843_g1,c58217_g1,c19496_g1,c89738_g1,c96691_g1,c52104_g1,c85426_g1,c75818_g1,c59352_g1,c94369_g1,c61171_g1,c9460_g2,c73214_g1,c13933_g1,c43484_g1,c93546_g1,c47587_g1,c48114_g1,c62599_g1,c45475_g1,c82844_g1,c104764_g1,c51923_g1,c108494_g1,c75097_g1,c86879_g1,c38471_g1,c30486_g2,c14311_g1,c51473_g2,c30486_g3,c49828_g1,c40424_g1,c7804_g1,c88505_g1,c45587_g1,c17647_g1,c59360_g1,c51468_g1,c703_g1,c18330_g1,c7076_g1,c43298_g1,c93365_g1,c55924_g1,c27920_g1,c6075_g1,c110650_g1,c56887_g1,c101207_g1,c35383_g1,c83184_g1,c71662_g1,c39543_g1,c34230_g1,c26946_g1,c51687_g3,c56501_g1,c48794_g1,c8448_g1,c52597_g1,c30868_g1,c72535_g1,c84326_g1,c71045_g1,c82688_g1,c31587_g1,c77336_g1,c30486_g1,c31830_g1,c70327_g1,c49145_g1,c31251_g1,c65050_g1,c77560_g1,c102213_g1,c86026_g1,c38253_g1,c19042_g1,c41349_g1,c72326_g1,c50269_g3,c45156_g1,c108889_g1,c75676_g1,c18161_g1,c86489_g1,c58019_g1,c54723_g1,c30518_g1,c52413_g3,c37689_g1,c54926_g1,c79194_g1,c64363_g1,c60256_g1,c65565_g1,c100075_g1,c34368_g1,c40051_g1,c94352_g1,c97219_g1,c16834_g1,c32354_g1,c55559_g1,c52086_g1,c57085_g1,c82349_g1,c37697_g1,c51687_g2,c77164_g1,c105795_g1,c78655_g1,c26398_g1,c11160_g1,c56658_g1,c95446_g1,c83855_g1,c94736_g1,c46989_g1,c50576_g1,c1334_g1,c86000_g1,c72040_g1,c19158_g1,c99310_g1,c97398_g1,c63562_g1,c96864_g1,c73286_g1,c61341_g1,c49610_g1,c94065_g1,c28281_g1,c79435_g1,c45291_g2,c48724_g1,c76864_g1,c112008_g1,c27176_g1,c103367_g1,c45253_g1,c84830_g1,c83975_g1,c26895_g1,c55367_g1,c104506_g1,c64976_g1,c88514_g1,c64403_g1,c96545_g1 |
| Pertussis | ko05133 | 340 | c76621_g1,c28479_g1,c104104_g1,c82662_g1,c8503_g1,c35897_g1,c42058_g1,c42455_g1,c20428_g1,c31551_g1,c31284_g1,c48654_g1,c63236_g1,c49133_g1,c90349_g1,c52047_g3,c3181_g1,c19656_g1,c67256_g1,c85350_g1,c64798_g1,c36093_g1,c9338_g1,c50509_g1,c40168_g1,c51362_g1,c53399_g1,c46431_g1,c68166_g1,c51393_g2,c109082_g1,c41136_g1,c50433_g1,c80845_g1,c76045_g1,c50319_g1,c41452_g1,c77468_g1,c48850_g1,c68992_g1,c99939_g1,c67306_g1,c20958_g1,c59703_g1,c70554_g1,c38105_g1,c25682_g1,c33484_g1,c51493_g1,c28106_g1,c50497_g1,c87121_g1,c30825_g1,c4653_g1,c55823_g1,c5974_g1,c41770_g1,c24811_g1,c44772_g1,c29500_g1,c59145_g1,c51579_g1,c21101_g1,c43041_g1,c19257_g1,c93320_g1,c72649_g1,c29983_g2,c43961_g1,c53776_g1,c52309_g1,c48726_g1,c51393_g1,c73826_g1,c20517_g1,c54671_g1,c106847_g1,c20072_g1,c37055_g1,c77647_g1,c52385_g2,c3185_g1,c49940_g3,c43215_g1,c99845_g1,c89573_g1,c16333_g1,c103884_g1,c64857_g1,c24563_g1,c52687_g3,c27364_g1,c49555_g1,c97243_g1,c60036_g1,c44871_g1,c28878_g1,c43552_g1,c42662_g1,c12545_g1,c19670_g1,c46019_g1,c99189_g1,c69108_g1,c50706_g2,c49045_g1,c28733_g1,c106613_g1,c37692_g1,c51250_g1,c85938_g1,c48076_g1,c85515_g1,c50542_g1,c75097_g1,c27417_g1,c105342_g1,c46894_g1,c74335_g1,c43584_g2,c30663_g1,c5967_g1,c89522_g1,c12183_g1,c27559_g1,c45585_g1,c31723_g1,c29860_g1,c50354_g1,c36550_g1,c67391_g1,c51087_g1,c52810_g1,c44213_g2,c63994_g1,c25760_g1,c102241_g1,c84537_g1,c56011_g1,c52221_g2,c35128_g1,c25032_g1,c97090_g1,c31283_g1,c31260_g1,c41811_g1,c93658_g1,c38149_g1,c63402_g1,c74219_g1,c31287_g1,c73710_g1,c34284_g1,c103279_g1,c41052_g1,c49457_g4,c33637_g1,c44244_g1,c25651_g1,c2927_g1,c51933_g1,c45078_g1,c11780_g1,c50217_g1,c43622_g1,c48112_g2,c99332_g1,c35585_g2,c37345_g1,c39201_g1,c48245_g1,c52206_g3,c38021_g1,c34104_g1,c36833_g1,c26902_g1,c49999_g4,c52084_g5,c45277_g1,c48988_g3,c24007_g1,c11267_g1,c48713_g2,c47048_g2,c28071_g1,c46096_g1,c21819_g1,c40803_g1,c38428_g2,c108116_g1,c35585_g1,c97045_g1,c25369_g1,c13250_g1,c44805_g1,c52646_g1,c42711_g1,c16958_g1,c65482_g1,c77087_g1,c45556_g1,c84103_g1,c55120_g1,c34788_g1,c51243_g7,c48972_g1,c20000_g1,c48288_g1,c61555_g1,c57458_g1,c58309_g1,c45137_g1,c6980_g1,c38091_g2,c41208_g1,c77837_g1,c106258_g1,c35140_g1,c20246_g1,c39725_g1,c25026_g1,c51314_g5,c48833_g1,c106429_g1,c34931_g1,c35002_g1,c40640_g1,c77655_g1,c39243_g1,c51009_g1,c65337_g1,c23872_g1,c46394_g2,c26562_g1,c97184_g1,c12015_g1,c45152_g1,c17204_g1,c1970_g1,c95560_g1,c109295_g1,c48337_g1,c45705_g1,c40297_g1,c50480_g1,c71256_g1,c17979_g1,c41047_g1,c40051_g1,c17204_g2,c79424_g1,c407_g1,c31785_g1,c43106_g1,c39874_g1,c16587_g1,c45705_g2,c29193_g1,c75439_g1,c71808_g1,c48425_g1,c41500_g1,c6637_g1,c38710_g1,c96255_g1,c53988_g1,c44980_g1,c63909_g1,c49276_g4,c92119_g1,c46059_g2,c86481_g1,c47535_g2,c52687_g2,c63601_g1,c32428_g1,c74294_g1,c63478_g1,c92831_g1,c2959_g1,c36767_g1,c110032_g1,c51379_g1,c31527_g1,c82782_g1,c52047_g1,c84912_g1,c14738_g1,c48723_g1,c29144_g1,c50422_g6,c70565_g1,c46519_g1,c85278_g1,c27075_g1,c50171_g1,c5896_g1,c88625_g1,c41879_g1,c72114_g1,c12186_g1,c33668_g1,c44897_g1,c22716_g1,c15748_g1,c32004_g1,c27875_g1,c108348_g1,c61603_g1,c34370_g1,c107634_g1,c52773_g1,c29143_g1,c75170_g1,c37602_g1,c48569_g1,c5603_g1,c52449_g2,c52687_g4,c54042_g1,c10126_g1,c62984_g1,c49158_g1,c44871_g2,c28657_g2,c68809_g1,c47048_g1,c41963_g1,c31673_g1,c38805_g1,c48713_g1,c34410_g1,c47729_g1,c33820_g1,c31195_g1,c59828_g1,c48463_g1,c45364_g1,c607_g1,c86378_g1 |
| Primary immunodeficiency | ko05340 | 5 | c19372_g1,c44576_g1,c83003_g1,c40460_g1,c61973_g1 |
| Prion diseases | ko05020 | 41 | c97556_g1,c105914_g1,c49227_g1,c45734_g1,c94179_g1,c109575_g1,c62393_g1,c76398_g1,c39157_g1,c57588_g1,c43524_g2,c75818_g1,c1904_g1,c75097_g1,c84054_g1,c41022_g2,c109997_g1,c45587_g1,c94686_g1,c94922_g1,c90324_g1,c64653_g1,c84044_g1,c64975_g1,c105364_g1,c17391_g2,c35201_g1,c41022_g3,c44890_g1,c27256_g1,c37339_g1,c42257_g1,c41022_g1,c65565_g1,c49071_g1,c80850_g1,c40051_g1,c28048_g1,c108541_g1,c95174_g1,c790_g1 |
| Prostate cancer | ko05215 | 93 | c803_g1,c8275_g1,c31614_g1,c64341_g1,c111162_g1,c60514_g1,c49070_g1,c81516_g1,c30744_g1,c68079_g1,c76236_g1,c21454_g1,c84030_g1,c35896_g1,c19496_g1,c89738_g1,c96691_g1,c85426_g1,c75818_g1,c59352_g1,c13933_g1,c48114_g1,c62599_g1,c45475_g1,c82844_g1,c104764_g1,c75097_g1,c38471_g1,c30486_g2,c14311_g1,c30486_g3,c49828_g1,c40424_g1,c7804_g1,c45587_g1,c17647_g1,c59360_g1,c703_g1,c18330_g1,c43298_g1,c110650_g1,c56887_g1,c53373_g1,c101207_g1,c35383_g1,c83184_g1,c51687_g3,c56501_g1,c8448_g1,c72535_g1,c84326_g1,c82688_g1,c30486_g1,c31830_g1,c70327_g1,c77560_g1,c40669_g1,c102213_g1,c86026_g1,c41349_g1,c75676_g1,c18161_g1,c54723_g1,c30518_g1,c54926_g1,c79194_g1,c64363_g1,c65565_g1,c40051_g1,c94352_g1,c97219_g1,c57085_g1,c82349_g1,c51687_g2,c105795_g1,c46989_g1,c50576_g1,c65911_g1,c86000_g1,c72040_g1,c99310_g1,c63562_g1,c96864_g1,c73286_g1,c49610_g1,c94065_g1,c79435_g1,c48724_g1,c112008_g1,c27176_g1,c45253_g1,c64976_g1,c88514_g1 |
| Proteoglycans in cancer | ko05205 | 115 | c70915_g1,c110547_g1,c35102_g1,c57048_g1,c109525_g1,c4329_g1,c87214_g1,c8820_g1,c47273_g1,c53983_g1,c49070_g1,c81516_g1,c73569_g1,c25801_g1,c51416_g1,c48247_g2,c85476_g1,c54485_g1,c54500_g1,c37374_g1,c96884_g1,c75818_g1,c104643_g1,c61171_g1,c48563_g1,c93546_g1,c104688_g1,c27032_g1,c41850_g1,c105751_g1,c75097_g1,c109771_g1,c52404_g2,c45587_g1,c7076_g1,c93365_g1,c55924_g1,c84111_g1,c27920_g1,c88836_g1,c53373_g1,c42666_g1,c71662_g1,c49684_g1,c50276_g3,c25384_g1,c46440_g1,c71068_g1,c79323_g1,c30868_g1,c72535_g1,c77336_g1,c61876_g1,c99954_g1,c8028_g1,c29612_g2,c40669_g1,c38253_g1,c50859_g1,c96785_g1,c61688_g1,c51321_g1,c49917_g2,c96169_g1,c72326_g1,c67900_g1,c44819_g1,c38276_g1,c24994_g1,c74373_g1,c100612_g1,c37689_g1,c61158_g1,c15085_g1,c103526_g1,c90282_g1,c48330_g1,c65565_g1,c10753_g1,c8199_g1,c44293_g1,c34368_g1,c40051_g1,c46805_g1,c103419_g1,c94013_g1,c40603_g1,c53890_g1,c111499_g1,c9260_g1,c26261_g1,c61421_g1,c76480_g1,c11862_g1,c54781_g1,c48692_g2,c109754_g1,c37697_g1,c59388_g1,c29612_g1,c78655_g1,c95446_g1,c83855_g1,c46989_g1,c65911_g1,c59050_g1,c45509_g1,c67782_g1,c62790_g1,c28281_g1,c44899_g1,c19310_g1,c103367_g1,c18207_g1,c26895_g1 |
| Renal cell carcinoma | ko05211 | 56 | c29237_g1,c4329_g1,c9460_g1,c48247_g2,c65074_g1,c85476_g1,c54485_g1,c88843_g1,c58217_g1,c75818_g1,c61171_g1,c9460_g2,c93546_g1,c75097_g1,c86879_g1,c88505_g1,c45587_g1,c7076_g1,c93365_g1,c55924_g1,c27920_g1,c35383_g1,c71662_g1,c26946_g1,c51687_g3,c30868_g1,c72535_g1,c77336_g1,c49145_g1,c38253_g1,c19042_g1,c72326_g1,c86489_g1,c37689_g1,c60256_g1,c65565_g1,c34368_g1,c40051_g1,c37697_g1,c51687_g2,c77164_g1,c78655_g1,c56658_g1,c95446_g1,c83855_g1,c46989_g1,c99310_g1,c97398_g1,c28281_g1,c76864_g1,c103367_g1,c84830_g1,c83975_g1,c26895_g1,c104506_g1,c64403_g1 |
| Rheumatoid arthritis | ko05323 | 68 | c90042_g1,c9517_g1,c17211_g1,c6604_g1,c96152_g1,c104875_g1,c83807_g1,c79303_g1,c18509_g1,c51416_g1,c13073_g1,c73651_g1,c106656_g1,c48563_g1,c7622_g1,c17915_g1,c41632_g1,c105751_g1,c50260_g1,c9292_g1,c83401_g1,c3915_g1,c61151_g1,c60627_g1,c84111_g1,c28919_g1,c23902_g1,c47794_g1,c13993_g1,c17178_g1,c31564_g1,c221_g1,c5914_g1,c17130_g1,c82933_g1,c50682_g1,c102717_g1,c90632_g1,c41028_g2,c56354_g1,c26063_g1,c92951_g1,c9519_g1,c15085_g1,c44602_g1,c103526_g1,c75790_g1,c36255_g1,c39769_g1,c27303_g1,c43095_g1,c98114_g1,c59129_g1,c27283_g1,c2494_g1,c42308_g1,c71294_g1,c39823_g1,c46465_g1,c47149_g1,c43979_g1,c26117_g1,c71628_g1,c42623_g1,c9525_g1,c93723_g1,c46242_g1,c110997_g1 |
| Salmonella infection | ko05132 | 59 | c4329_g1,c104533_g1,c85479_g1,c48247_g2,c85476_g1,c54485_g1,c44759_g1,c49048_g6,c49571_g1,c61171_g1,c17409_g1,c93546_g1,c75097_g1,c19566_g1,c45944_g1,c40746_g1,c7076_g1,c93365_g1,c55924_g1,c27920_g1,c88318_g1,c71662_g1,c82433_g1,c73389_g1,c17873_g1,c30868_g1,c77336_g1,c17051_g1,c49571_g2,c38253_g1,c72326_g1,c34934_g1,c72833_g1,c37689_g1,c28799_g1,c56900_g1,c34368_g1,c40051_g1,c16097_g1,c83648_g1,c48318_g1,c37697_g1,c26247_g1,c74638_g1,c78655_g1,c25443_g1,c95446_g1,c83855_g1,c45108_g2,c30988_g1,c28281_g1,c38523_g1,c92224_g1,c103367_g1,c105845_g1,c28605_g1,c26895_g1,c109715_g1,c105414_g1 |
| Shigellosis | ko05131 | 57 | c4329_g1,c104533_g1,c85479_g1,c48247_g2,c85476_g1,c54485_g1,c44759_g1,c49571_g1,c61171_g1,c17409_g1,c93546_g1,c75097_g1,c45944_g1,c7076_g1,c93365_g1,c55924_g1,c27920_g1,c85401_g1,c35630_g1,c88318_g1,c71662_g1,c82433_g1,c73389_g1,c39455_g1,c30868_g1,c77336_g1,c40571_g1,c34562_g1,c49571_g2,c38253_g1,c72326_g1,c34934_g1,c69492_g1,c72833_g1,c37689_g1,c28799_g1,c34368_g1,c40051_g1,c16097_g1,c83648_g1,c48318_g1,c37697_g1,c26247_g1,c78655_g1,c25443_g1,c95446_g1,c83855_g1,c45108_g2,c30988_g1,c91439_g1,c28281_g1,c103367_g1,c105845_g1,c28605_g1,c26895_g1,c109715_g1,c105414_g1 |
| Small cell lung cancer | ko05222 | 36 | c803_g1,c111162_g1,c108958_g1,c84030_g1,c105449_g1,c19496_g1,c94369_g1,c62599_g1,c38471_g1,c14311_g1,c40424_g1,c17647_g1,c6075_g1,c84326_g1,c71045_g1,c31587_g1,c35517_g1,c75676_g1,c64363_g1,c100075_g1,c16834_g1,c55559_g1,c26398_g1,c11160_g1,c1334_g1,c72040_g1,c63562_g1,c73286_g1,c94065_g1,c79435_g1,c45291_g2,c48724_g1,c45253_g1,c64976_g1,c88514_g1,c96545_g1 |
| Systemic lupus erythematosus | ko05322 | 35 | c36854_g1,c96970_g1,c20319_g1,c18498_g1,c67397_g1,c43024_g1,c27196_g1,c102088_g1,c7474_g1,c38995_g1,c42626_g1,c11134_g1,c40694_g1,c18553_g1,c10065_g1,c82591_g1,c33965_g1,c424_g1,c10659_g1,c47064_g1,c38037_g1,c49048_g4,c52886_g1,c61527_g1,c16547_g1,c24485_g1,c11337_g1,c31598_g1,c16380_g1,c36348_g2,c46861_g1,c46794_g1,c875_g1,c18217_g1,c73844_g1 |
| Thyroid cancer | ko05216 | 8 | c75818_g1,c43484_g1,c75097_g1,c45587_g1,c72535_g1,c65565_g1,c40051_g1,c46989_g1 |
| Toxoplasmosis | ko05145 | 426 | c28479_g1,c104104_g1,c82662_g1,c8503_g1,c35897_g1,c42058_g1,c42455_g1,c20428_g1,c31551_g1,c31284_g1,c91578_g1,c48654_g1,c43333_g1,c63236_g1,c48412_g1,c49133_g1,c90349_g1,c52047_g3,c81465_g1,c19656_g1,c67256_g1,c85350_g1,c64798_g1,c36093_g1,c9338_g1,c50509_g1,c51362_g1,c46431_g1,c68166_g1,c51393_g2,c109082_g1,c41136_g1,c50433_g1,c87471_g1,c76045_g1,c70797_g1,c41452_g1,c77468_g1,c48850_g1,c68992_g1,c68830_g1,c99939_g1,c67306_g1,c40971_g1,c20958_g1,c59703_g1,c108958_g1,c70554_g1,c25682_g1,c51493_g1,c77609_g1,c28106_g1,c65806_g1,c50497_g1,c46828_g2,c87121_g1,c30825_g1,c4653_g1,c55823_g1,c16658_g1,c24811_g1,c44772_g1,c29500_g1,c59145_g1,c95765_g1,c51579_g1,c21101_g1,c43041_g1,c19257_g1,c42444_g1,c93320_g1,c72649_g1,c105449_g1,c29983_g2,c43961_g1,c5979_g1,c52309_g1,c48726_g1,c51393_g1,c84871_g1,c99424_g1,c73826_g1,c90199_g1,c20517_g1,c96066_g1,c106847_g1,c20072_g1,c37055_g1,c77647_g1,c52385_g2,c49940_g3,c37866_g2,c43215_g1,c63084_g1,c99845_g1,c89573_g1,c16333_g1,c103884_g1,c46828_g3,c64857_g1,c24563_g1,c60132_g1,c30224_g1,c52687_g3,c27364_g1,c50227_g1,c49555_g1,c88688_g1,c47248_g1,c97243_g1,c60036_g1,c76196_g1,c97805_g1,c44871_g1,c73322_g1,c28878_g1,c75604_g1,c43552_g1,c42662_g1,c12545_g1,c19670_g1,c46019_g1,c99189_g1,c69108_g1,c50706_g2,c104689_g1,c49045_g1,c106613_g1,c19117_g1,c51250_g1,c85938_g1,c48076_g1,c85515_g1,c50542_g1,c75097_g1,c27003_g1,c27417_g1,c105342_g1,c98426_g1,c46894_g1,c13176_g1,c74335_g1,c43036_g1,c30663_g1,c5967_g1,c75980_g1,c89522_g1,c12183_g1,c27559_g1,c45585_g1,c31723_g1,c50354_g1,c36550_g1,c26982_g1,c44457_g1,c71422_g1,c36117_g1,c51087_g1,c52810_g1,c44213_g2,c63994_g1,c59458_g1,c6075_g1,c102241_g1,c56011_g1,c52221_g2,c97101_g1,c35128_g1,c25032_g1,c31283_g1,c53373_g1,c99321_g1,c75954_g1,c41811_g1,c93658_g1,c38149_g1,c63402_g1,c32274_g1,c74219_g1,c31287_g1,c73710_g1,c49457_g4,c33637_g1,c65797_g1,c25651_g1,c39357_g1,c2927_g1,c48306_g1,c51933_g1,c33834_g1,c20191_g1,c45078_g1,c11780_g1,c50217_g1,c71045_g1,c43622_g1,c32310_g2,c31587_g1,c48112_g2,c99332_g1,c42033_g1,c92599_g1,c35585_g2,c37345_g1,c39201_g1,c39157_g2,c48245_g1,c52206_g3,c38021_g1,c65057_g1,c26982_g2,c34104_g1,c36833_g1,c26902_g1,c7457_g1,c40669_g1,c75360_g1,c87038_g1,c49999_g4,c52084_g5,c45277_g1,c48988_g3,c11267_g1,c48713_g2,c47048_g2,c103460_g1,c54833_g1,c46096_g1,c34581_g1,c23873_g1,c40803_g1,c96576_g1,c63003_g1,c38428_g2,c108116_g1,c35585_g1,c97045_g1,c25369_g1,c13250_g1,c46353_g1,c44805_g1,c52646_g1,c42711_g1,c10943_g1,c16958_g1,c49357_g1,c65482_g1,c77087_g1,c45556_g1,c63919_g1,c84103_g1,c55120_g1,c51243_g7,c88796_g1,c48972_g1,c20000_g1,c48288_g1,c61555_g1,c104810_g1,c57458_g1,c58309_g1,c45137_g1,c38091_g2,c14060_g1,c41208_g1,c77837_g1,c106258_g1,c35140_g1,c103497_g1,c102073_g1,c20246_g1,c39725_g1,c25026_g1,c51314_g5,c18935_g1,c48833_g1,c105070_g1,c106429_g1,c34931_g1,c35002_g1,c40640_g1,c77655_g1,c36058_g1,c39243_g1,c51009_g1,c62353_g1,c23872_g1,c46828_g1,c41667_g1,c46394_g2,c26562_g1,c97184_g1,c12015_g1,c45152_g1,c17204_g1,c1970_g1,c95560_g1,c109295_g1,c48337_g1,c45705_g1,c40297_g1,c39270_g1,c50480_g1,c71256_g1,c17979_g1,c40051_g1,c17204_g2,c79424_g1,c43106_g1,c39874_g1,c16587_g1,c87049_g1,c45705_g2,c65754_g1,c29193_g1,c75439_g1,c71808_g1,c62342_g1,c48425_g1,c41500_g1,c38710_g1,c16834_g1,c96255_g1,c92789_g1,c34941_g2,c53988_g1,c44980_g1,c49276_g4,c92119_g1,c52519_g1,c55559_g1,c46059_g2,c47535_g2,c52687_g2,c63601_g1,c99231_g1,c32428_g1,c17193_g1,c74294_g1,c63478_g1,c2959_g1,c32310_g1,c13172_g1,c40516_g1,c36767_g1,c109031_g1,c110032_g1,c40506_g1,c51379_g1,c31527_g1,c52047_g1,c84912_g1,c11160_g1,c4455_g1,c14738_g1,c48723_g1,c50422_g6,c70565_g1,c34941_g1,c65911_g1,c85278_g1,c27075_g1,c48968_g3,c55706_g1,c50171_g1,c106778_g1,c5896_g1,c103564_g1,c49020_g1,c88625_g1,c41879_g1,c72114_g1,c12186_g1,c33668_g1,c44897_g1,c34702_g1,c22716_g1,c15748_g1,c32004_g1,c27875_g1,c82592_g1,c108348_g1,c47368_g1,c52279_g2,c61603_g1,c43036_g2,c34370_g1,c107634_g1,c52773_g1,c29143_g1,c56327_g1,c33019_g1,c37602_g1,c48569_g1,c13411_g1,c56533_g1,c5603_g1,c52449_g2,c57174_g1,c52687_g4,c54042_g1,c110498_g1,c107618_g1,c10126_g1,c62984_g1,c49158_g1,c74430_g1,c44871_g2,c26243_g1,c19301_g1,c47048_g1,c41963_g1,c102593_g1,c31673_g1,c38805_g1,c36789_g1,c45116_g1,c48713_g1,c34410_g1,c33820_g1,c59828_g1,c45364_g1,c607_g1,c86378_g1,c34489_g1,c96545_g1 |
| Transcriptional misregulation in cancers | ko05202 | 60 | c36854_g1,c57048_g1,c87214_g1,c20319_g1,c52413_g4,c8820_g1,c47628_g1,c52836_g1,c19284_g1,c45289_g1,c52736_g1,c37374_g1,c104643_g1,c38868_g1,c52836_g3,c73214_g1,c94103_g1,c23987_g1,c27196_g1,c108494_g1,c109771_g1,c102088_g1,c38995_g1,c52404_g2,c49684_g1,c48564_g1,c29607_g2,c71068_g1,c79323_g1,c46102_g2,c31251_g1,c99954_g1,c65050_g1,c8028_g1,c30173_g1,c61688_g1,c93256_g1,c38276_g1,c58019_g1,c24994_g1,c40688_g1,c10659_g1,c51710_g1,c52413_g3,c61158_g1,c10753_g1,c44293_g1,c52199_g2,c40603_g1,c107848_g1,c53890_g1,c61421_g1,c109754_g1,c46794_g1,c18217_g1,c59050_g1,c62625_g1,c19310_g1,c73844_g1,c4216_g1 |
| Tuberculosis | ko05152 | 451 | c76621_g1,c28479_g1,c104104_g1,c82662_g1,c8503_g1,c90042_g1,c35897_g1,c42058_g1,c42455_g1,c26595_g1,c20428_g1,c31551_g1,c17211_g1,c31284_g1,c6604_g1,c48654_g1,c63236_g1,c110633_g1,c49133_g1,c90349_g1,c52047_g3,c51771_g2,c3181_g1,c44465_g1,c19656_g1,c67256_g1,c85350_g1,c64798_g1,c36093_g1,c9338_g1,c50509_g1,c96152_g1,c104875_g1,c51362_g1,c46431_g1,c68166_g1,c51393_g2,c109082_g1,c41136_g1,c50433_g1,c80845_g1,c76045_g1,c50319_g1,c41452_g1,c77468_g1,c48850_g1,c97107_g1,c68992_g1,c99939_g1,c67306_g1,c94546_g1,c20958_g1,c59703_g1,c108958_g1,c70554_g1,c25682_g1,c33484_g1,c51493_g1,c28106_g1,c50497_g1,c87121_g1,c30825_g1,c4653_g1,c55823_g1,c5974_g1,c24811_g1,c44772_g1,c105045_g1,c29500_g1,c59145_g1,c51579_g1,c21101_g1,c43041_g1,c19257_g1,c29965_g1,c93320_g1,c72649_g1,c105449_g1,c29983_g2,c43961_g1,c51840_g8,c25101_g1,c53776_g1,c52309_g1,c48726_g1,c71297_g1,c106656_g1,c51393_g1,c73826_g1,c73840_g1,c20517_g1,c106847_g1,c20072_g1,c37055_g1,c77647_g1,c52385_g2,c3185_g1,c49940_g3,c43215_g1,c49048_g6,c99845_g1,c104026_g1,c46921_g2,c89573_g1,c16333_g1,c103884_g1,c108570_g1,c1291_g1,c64857_g1,c111368_g1,c24563_g1,c52687_g3,c27364_g1,c49555_g1,c97243_g1,c41569_g1,c60036_g1,c44871_g1,c28878_g1,c43552_g1,c42662_g1,c12545_g1,c19670_g1,c28040_g1,c46019_g1,c67603_g1,c99189_g1,c41632_g1,c69108_g1,c50706_g2,c49045_g1,c106613_g1,c37692_g1,c51250_g1,c85938_g1,c48076_g1,c85515_g1,c50542_g1,c75097_g1,c45182_g1,c32804_g1,c19566_g1,c9292_g1,c83401_g1,c48503_g2,c96524_g1,c27417_g1,c61151_g1,c105342_g1,c46894_g1,c49876_g1,c74335_g1,c43584_g2,c30663_g1,c5967_g1,c89522_g1,c12183_g1,c27559_g1,c45585_g1,c31723_g1,c29860_g1,c50354_g1,c36550_g1,c67391_g1,c51087_g1,c52810_g1,c44213_g2,c63994_g1,c40746_g1,c28034_g1,c28919_g1,c6075_g1,c25760_g1,c102241_g1,c52228_g1,c84537_g1,c96174_g1,c56011_g1,c52221_g2,c35128_g1,c25032_g1,c97090_g1,c31283_g1,c34843_g1,c69639_g1,c35383_g1,c31260_g1,c41811_g1,c47794_g1,c93658_g1,c38149_g1,c63402_g1,c74219_g1,c31287_g1,c73710_g1,c82692_g1,c34284_g1,c28119_g1,c103279_g1,c35887_g1,c41052_g1,c94822_g1,c49457_g4,c33637_g1,c44244_g1,c25651_g1,c13993_g1,c51687_g3,c2927_g1,c45851_g1,c17873_g1,c51933_g1,c26986_g1,c45078_g1,c11780_g1,c50217_g1,c71045_g1,c43622_g1,c31587_g1,c48112_g2,c99332_g1,c16239_g1,c35585_g2,c37345_g1,c221_g1,c17130_g1,c50682_g1,c39201_g1,c48245_g1,c52206_g3,c17051_g1,c38021_g1,c34104_g1,c36833_g1,c26902_g1,c24603_g1,c102717_g1,c90632_g1,c49999_g4,c52084_g5,c45277_g1,c48988_g3,c13394_g1,c24007_g1,c11267_g1,c48713_g2,c47048_g2,c46921_g1,c46096_g1,c42106_g2,c40803_g1,c38428_g2,c108116_g1,c49758_g1,c35585_g1,c97045_g1,c25369_g1,c13250_g1,c41028_g2,c44805_g1,c52646_g1,c42711_g1,c16958_g1,c10662_g1,c86007_g1,c56354_g1,c60324_g1,c65482_g1,c77087_g1,c26063_g1,c45556_g1,c90874_g1,c84103_g1,c55120_g1,c34788_g1,c51243_g7,c48972_g1,c20000_g1,c48288_g1,c61555_g1,c64498_g1,c57458_g1,c58309_g1,c45137_g1,c6980_g1,c38091_g2,c41208_g1,c77837_g1,c43877_g1,c106258_g1,c9519_g1,c35140_g1,c20246_g1,c56900_g1,c39725_g1,c25026_g1,c51314_g5,c48833_g1,c106429_g1,c34931_g1,c35002_g1,c40640_g1,c77655_g1,c39243_g1,c51009_g1,c47867_g1,c65337_g1,c23872_g1,c75790_g1,c46394_g2,c26562_g1,c97184_g1,c12015_g1,c45152_g1,c17204_g1,c1970_g1,c95560_g1,c109295_g1,c36255_g1,c48337_g1,c45705_g1,c40297_g1,c50480_g1,c71256_g1,c17979_g1,c72908_g1,c41047_g1,c40051_g1,c17204_g2,c79424_g1,c39769_g1,c31785_g1,c41138_g1,c27303_g1,c43106_g1,c39874_g1,c16587_g1,c43095_g1,c45705_g2,c98114_g1,c73917_g1,c29193_g1,c75439_g1,c71808_g1,c48425_g1,c41500_g1,c6637_g1,c38710_g1,c16834_g1,c96255_g1,c53988_g1,c44980_g1,c63909_g1,c49276_g4,c92119_g1,c55559_g1,c46059_g2,c35043_g1,c86481_g1,c47535_g2,c52687_g2,c63601_g1,c64196_g1,c32428_g1,c44993_g1,c74294_g1,c63478_g1,c92831_g1,c2959_g1,c30837_g1,c36767_g1,c110032_g1,c42308_g1,c51687_g2,c51379_g1,c31527_g1,c82782_g1,c52047_g1,c71294_g1,c104243_g1,c42117_g1,c84912_g1,c11160_g1,c42572_g1,c9382_g1,c14738_g1,c94398_g1,c48723_g1,c50422_g6,c70565_g1,c85278_g1,c27075_g1,c72709_g1,c50171_g1,c5896_g1,c88625_g1,c41879_g1,c72114_g1,c12186_g1,c33668_g1,c44897_g1,c4502_g1,c104287_g1,c22716_g1,c15748_g1,c99310_g1,c32004_g1,c18447_g1,c27875_g1,c108348_g1,c61603_g1,c34370_g1,c107634_g1,c52773_g1,c29143_g1,c38523_g1,c37602_g1,c44899_g1,c48569_g1,c5603_g1,c52449_g2,c9525_g1,c52687_g4,c54042_g1,c93723_g1,c10126_g1,c62984_g1,c49158_g1,c44871_g2,c28657_g2,c20543_g1,c26798_g1,c68809_g1,c85883_g1,c47048_g1,c41963_g1,c31673_g1,c38805_g1,c69504_g1,c48713_g1,c74487_g1,c34410_g1,c33820_g1,c31195_g1,c59828_g1,c48463_g1,c45364_g1,c607_g1,c86378_g1,c45023_g1,c96545_g1 |
| Type I diabetes mellitus | ko04940 | 30 | c45383_g1,c110633_g1,c97107_g1,c94546_g1,c105045_g1,c51840_g8,c25101_g1,c73840_g1,c104026_g1,c1291_g1,c111368_g1,c28040_g1,c67603_g1,c2590_g1,c50971_g1,c28034_g1,c52228_g1,c96174_g1,c69639_g1,c30097_g1,c65617_g1,c49758_g1,c90874_g1,c94313_g1,c64196_g1,c34353_g1,c52918_g1,c42117_g1,c104287_g1,c85883_g1 |
| Type II diabetes mellitus | ko04930 | 31 | c48127_g2,c6348_g1,c48844_g2,c49070_g1,c81516_g1,c52344_g5,c27600_g1,c19297_g1,c61871_g1,c37799_g1,c46151_g1,c51735_g1,c75097_g1,c47668_g1,c48132_g2,c103573_g1,c47074_g1,c57387_g1,c100584_g1,c79843_g1,c82523_g1,c40051_g1,c50236_g1,c50016_g1,c30673_g1,c38423_g1,c58199_g1,c26284_g1,c52381_g3,c58304_g1,c52381_g2 |
| Vibrio cholerae infection | ko05110 | 102 | c30767_g1,c90042_g1,c26011_g1,c17158_g1,c9517_g1,c17211_g1,c6604_g1,c4018_g1,c96152_g1,c104875_g1,c79303_g1,c84465_g1,c18509_g1,c50317_g5,c13073_g1,c77978_g1,c73651_g1,c106656_g1,c103445_g1,c24701_g3,c4911_g1,c7622_g1,c50317_g4,c17915_g1,c41632_g1,c50260_g1,c9292_g1,c83401_g1,c3915_g1,c1039_g1,c61151_g1,c55144_g1,c48699_g4,c60627_g1,c50317_g2,c28919_g1,c23902_g1,c107497_g1,c49230_g2,c38067_g1,c47794_g1,c57725_g1,c13993_g1,c50317_g3,c30184_g1,c26875_g1,c106096_g1,c28392_g1,c17178_g1,c31564_g1,c221_g1,c5914_g1,c17130_g1,c82933_g1,c50682_g1,c28233_g1,c102717_g1,c90632_g1,c38150_g1,c41028_g2,c56354_g1,c26063_g1,c43332_g1,c92951_g1,c66013_g1,c82967_g1,c93792_g1,c9519_g1,c44602_g1,c75790_g1,c76939_g1,c36255_g1,c6247_g1,c39769_g1,c27303_g1,c43095_g1,c98114_g1,c59129_g1,c72267_g1,c27283_g1,c102275_g1,c76967_g1,c24701_g2,c24701_g1,c2494_g1,c42308_g1,c106932_g1,c71294_g1,c39823_g1,c46465_g1,c16093_g1,c43979_g1,c71628_g1,c46736_g1,c9525_g1,c93723_g1,c64712_g1,c46242_g1,c73470_g1,c110997_g1,c4535_g1,c3699_g1 |
| Viral carcinogenesis | ko05203 | 215 | c36207_g1,c90042_g1,c803_g1,c28372_g1,c32329_g1,c48127_g2,c4329_g1,c52413_g4,c31541_g1,c112082_g1,c96152_g1,c111162_g1,c96830_g1,c39247_g1,c48844_g2,c49295_g1,c19284_g1,c52344_g5,c89511_g1,c55928_g1,c106501_g1,c27600_g1,c45590_g1,c96041_g1,c48247_g2,c85476_g1,c19297_g1,c54485_g1,c84030_g1,c51432_g1,c61871_g1,c45652_g1,c37799_g1,c36334_g1,c79969_g1,c18148_g1,c97388_g1,c28530_g1,c106498_g1,c61171_g1,c27289_g1,c71920_g1,c67397_g1,c10987_g1,c95007_g1,c73214_g1,c52628_g2,c46151_g1,c93546_g1,c973_g1,c45932_g1,c80928_g1,c51735_g1,c100646_g1,c53264_g1,c62599_g1,c76554_g1,c108494_g1,c41727_g1,c75097_g1,c70785_g1,c56060_g1,c14311_g1,c61151_g1,c65507_g1,c52316_g2,c35127_g1,c40424_g1,c3969_g1,c27608_g1,c109664_g1,c47668_g1,c42626_g1,c33990_g1,c50281_g2,c41668_g1,c108594_g1,c88723_g1,c48132_g2,c100593_g1,c7076_g1,c19545_g1,c93365_g1,c55924_g1,c101381_g1,c23121_g1,c27920_g1,c99764_g1,c103573_g1,c86149_g1,c11134_g1,c44201_g1,c49147_g1,c93012_g1,c49120_g1,c35383_g1,c48215_g1,c71662_g1,c80599_g1,c47074_g1,c51687_g3,c40006_g1,c111208_g1,c60531_g1,c30868_g1,c84326_g1,c44250_g1,c63359_g1,c77336_g1,c38807_g1,c57387_g1,c26955_g1,c76903_g1,c31251_g1,c4652_g1,c83690_g1,c65050_g1,c66821_g1,c52168_g1,c4201_g1,c37894_g1,c38253_g1,c104685_g1,c40410_g1,c53875_g1,c79843_g1,c39520_g1,c72326_g1,c1497_g1,c75676_g1,c82523_g1,c14104_g1,c37003_g1,c58019_g1,c56354_g1,c38755_g1,c52413_g3,c105296_g1,c37689_g1,c47064_g1,c49649_g1,c24394_g1,c87317_g1,c43796_g1,c58700_g1,c59617_g1,c94737_g1,c53323_g1,c52628_g1,c48865_g3,c100075_g1,c24337_g1,c34368_g1,c40051_g1,c24485_g1,c96721_g1,c64109_g1,c15250_g1,c49667_g4,c50016_g1,c37697_g1,c104235_g1,c24911_g1,c51687_g2,c71294_g1,c49211_g2,c78655_g1,c33990_g2,c34592_g1,c11362_g1,c73204_g1,c31781_g1,c875_g1,c95446_g1,c83855_g1,c56882_g1,c52316_g3,c38423_g1,c1334_g1,c94639_g1,c33613_g1,c27083_g1,c16456_g1,c101900_g1,c72040_g1,c11161_g1,c99310_g1,c46780_g1,c63562_g1,c105022_g1,c73286_g1,c94065_g1,c58199_g1,c36591_g1,c111047_g1,c28281_g1,c77461_g1,c50038_g1,c45291_g2,c103367_g1,c4402_g1,c93723_g1,c26284_g1,c110043_g1,c58304_g1,c52168_g2,c26895_g1,c40022_g1,c50149_g2,c45230_g2,c104593_g1,c98046_g1,c43507_g2,c88514_g1,c77498_g1 |
| Viral myocarditis | ko05416 | 40 | c4329_g1,c56873_g1,c108958_g1,c79316_g1,c48247_g2,c85476_g1,c54485_g1,c51982_g3,c105449_g1,c61171_g1,c93546_g1,c7076_g1,c93365_g1,c55924_g1,c27920_g1,c6075_g1,c71662_g1,c30868_g1,c71045_g1,c31587_g1,c77336_g1,c53812_g1,c38253_g1,c72326_g1,c37667_g1,c37689_g1,c78027_g1,c34368_g1,c16834_g1,c55559_g1,c37697_g1,c78655_g1,c11160_g1,c95446_g1,c83855_g1,c28281_g1,c103367_g1,c110549_g1,c26895_g1,c96545_g1 |
