# Supplementary material for: Transcriptome analysis and identification of key genes involved in 1-deoxynojirimycin biosynthesis of mulberry (Morus alba L.)
Source: PeerJ. 2018 Aug 23;6:e5443. doi: 10.7717/peerj.5443 (PMC6109587; doi:10.7717/peerj.5443)
Supplement: Supplemental Information 7 [file peerj-06-5443-s007.doc]

**Table S4 Expression statistics of transcripts between M7 and M11 libraries**

| Class | Number(#) of Transcripts | % |
| --- | --- | --- |
| Total transcripts | 112,481 |  |
| Expressed transcripts | 89,606 | 79.66 |
| Expressed in M7 | 68,235 | 76.15 |
| Expressed in M11 | 66,751 | 74.49 |
| Expressed both in M7 and M11 | 42,994 | 47.98 |
| Expressed only in M7 | 25,241 | 28.17 |
| Expressed only in M11 | 23,757 | 26.51 |
| Differentially expressed transcripts  (p≤0.01 & (ratio≥2 or ratio≤0.5) | Total # | 11,318 |
|  | Up # | 6,606 |
|  | Down # | 4,712 |
