# Supplementary material for: Transcriptome analysis and identification of key genes involved in 1-deoxynojirimycin biosynthesis of mulberry (Morus alba L.)
Source: PeerJ. 2018 Aug 23;6:e5443. doi: 10.7717/peerj.5443 (PMC6109587; doi:10.7717/peerj.5443)
Supplement: Supplemental Information 8 [file peerj-06-5443-s008.doc]

**Table S5 Significantly differentially expressed transcripts assigned to 294 KEGG pathways between the two mulberry libraries.**

| **Pathway** | **ko ID** | **Total #** | **Differently expressed transcripts** | | | | | |
| --- | --- | --- | --- | --- | --- | --- | --- | --- |
|  |  |  | **Total** | **P** | **Up** | **P** | **Down** | **P** |
| **>1. Metabolism** |  |  |  |  |  |  |  |  |
| 2-Oxocarboxylic acid metabolism | ko01210 | 177 | 23 | 0.9618318 | 6 | 0.99408 | 17 | 0.605605 |
| Acridone alkaloid biosynthesis | ko01058 | 1 | 1 | 0.1760398 | 1 | 0.076157 |  |  |
| Alanine | ko00250 | 177 | 17 | 0.9991079 | 4 | 0.999525 | 13 | 0.90968 |
| alpha-Linolenic acid metabolism | ko00592 | 94 | 21 | 0.1417412 | 10 | 0.177132 | 11 | 0.336412 |
| Amino sugar and nucleotide sugar metabolism | ko00520 | 175 | 19 | 0.9953024 | 8 | 0.961512 | 11 | 0.968881 |
| Aminobenzoate degradation | ko00627 | 45 | 18 | 0.0003324 | 4 | 0.451379 | 14 | 7.73E-05 |
| Anthocyanin biosynthesis | ko00942 | 3 | 1 | 0.4406399 | 1 | 0.211532 |  |  |
| Arachidonic acid metabolism | ko00590 | 39 | 8 | 0.3786554 | 6 | 0.07256 | 2 | 0.912469 |
| Arginine and proline metabolism | ko00330 | 204 | 27 | 0.9635995 | 5 | 0.999636 | 22 | 0.384744 |
| Ascorbate and aldarate metabolism | ko00053 | 90 | 16 | 0.5262531 | 3 | 0.971927 | 13 | 0.110896 |
| Benzoate degradation | ko00362 | 29 | 4 | 0.7769294 |  |  | 4 | 0.328085 |
| beta-Alanine metabolism | ko00410 | 171 | 22 | 0.963522 | 10 | 0.84822 | 12 | 0.930436 |
| Biosynthesis of amino acids | ko01230 | 636 | 88 | 0.9965059 | 27 | 0.999865 | 61 | 0.655329 |
| Biosynthesis of ansamycins | ko01051 | 15 | 1 | 0.9453436 | 1 | 0.695488 |  |  |
| Biosynthesis of unsaturated fatty acids | ko01040 | 94 | 18 | 0.387508 | 5 | 0.852722 | 13 | 0.14186 |
| Biotin metabolism | ko00780 | 47 | 4 | 0.9758512 | 1 | 0.976049 | 3 | 0.861709 |
| Bisphenol degradation | ko00363 | 38 | 14 | 0.003716 | 4 | 0.327869 | 10 | 0.003305 |
| Brassinosteroid biosynthesis | ko00905 | 21 | 6 | 0.1500716 | 1 | 0.810841 | 5 | 0.051748 |
| Butanoate metabolism | ko00650 | 71 | 4 | 0.9992872 | 1 | 0.996462 | 3 | 0.977856 |
| C5-Branched dibasic acid metabolism | ko00660 | 31 | 1 | 0.9975517 |  |  | 1 | 0.961887 |
| Caffeine metabolism | ko00232 | 17 | 3 | 0.5970465 |  |  | 3 | 2.38E-01 |
| Carbon fixation in photosynthetic organisms | ko00710 | 177 | 43 | 0.014407 | 25 | 0.001912 | 18 | 0.504996 |
| Carbon fixation pathways in prokaryotes | ko00720 | 139 | 14 | 0.9955373 | 2 | 0.999807 | 12 | 0.74489 |
| Carbon metabolism | ko01200 | 763 | 106 | 0.9982491 | 45 | 0.976345 | 61 | 9.78E-01 |
| Carotenoid biosynthesis | ko00906 | 41 | 10 | 0.1722892 | 9 | 0.00314 | 1 | 0.986745 |
| Chloroalkane and chloroalkene degradation | ko00625 | 52 | 7 | 0.83347 | 2 | 0.914585 | 5 | 0.603975 |
| Chlorocyclohexane and chlorobenzene degradation | ko00361 | 4 | 1 | 0.5391384 |  |  | 1 | 0.343601 |
| Citrate cycle (TCA cycle) | ko00020 | 220 | 18 | 0.9999829 | 1 | 1 | 17 | 0.896864 |
| Cutin | ko00073 | 28 | 8 | 0.1047909 | 3 | 0.360439 | 5 | 0.141325 |
| Cyanoamino acid metabolism | ko00460 | 91 | 20 | 0.167155 | 12 | 0.042675 | 8 | 0.700202 |
| Cysteine and methionine metabolism | ko00270 | 198 | 29 | 0.8864042 | 10 | 0.941724 | 19 | 0.609182 |
| Degradation of aromatic compounds | ko01220 | 34 | 2 | 0.9886653 | 1 | 0.932645 | 1 | 0.972234 |
| D-Glutamine and D-glutamate metabolism | ko00471 | 9 | 3 | 0.200638 | 1 | 0.509931 | 2 | 0.224722 |
| Diterpenoid biosynthesis | ko00904 | 33 | 10 | 0.0522682 | 9 | 0.000599 | 1 | 0.969141 |
| Drug metabolism - cytochrome P450 | ko00982 | 64 | 15 | 0.144041 | 6 | 0.360849 | 9 | 0.185087 |
| Drug metabolism - other enzymes | ko00983 | 61 | 4 | 0.99674 |  |  | 4 | 0.871189 |
| Ether lipid metabolism | ko00565 | 26 | 7 | 0.1594752 | 3 | 0.317537 | 4 | 0.258224 |
| Fatty acid biosynthesis | ko00061 | 74 | 7 | 0.9835562 | 2 | 0.980096 | 5 | 0.874097 |
| Fatty acid degradation | ko00071 | 134 | 16 | 0.9724588 | 3 | 0.9983 | 13 | 0.587218 |
| Fatty acid elongation | ko00062 | 45 | 11 | 0.1554991 | 3 | 0.676817 | 8 | 0.074846 |
| Fatty acid metabolism | ko01212 | 191 | 25 | 0.963925 | 7 | 0.992246 | 18 | 0.639456 |
| Flavone and flavonol biosynthesis | ko00944 | 12 | 2 | 0.6511824 | 1 | 0.61368 | 1 | 0.717331 |
| Flavonoid biosynthesis | ko00941 | 58 | 22 | 0.000189 | 15 | 1.97E-05 | 7 | 0.358433 |
| Fluorobenzoate degradation | ko00364 | 4 | 1 | 0.5391384 |  |  | 1 | 0.343601 |
| Folate biosynthesis | ko00790 | 28 | 4 | 0.7520537 | 1 | 0.891501 | 3 | 0.540031 |
| Fructose and mannose metabolism | ko00051 | 116 | 23 | 0.2984918 | 9 | 0.527358 | 14 | 0.266945 |
| Galactose metabolism | ko00052 | 99 | 15 | 0.7778844 | 5 | 0.881834 | 10 | 0.534488 |
| Glucosinolate biosynthesis | ko00966 | 7 | 5 | 0.0025775 | 4 | 0.000969 | 1 | 0.521376 |
| Glutathione metabolism | ko00480 | 151 | 25 | 0.6662952 | 7 | 0.94779 | 18 | 0.247911 |
| Glycerolipid metabolism | ko00561 | 125 | 24 | 0.3538882 | 8 | 0.745256 | 16 | 0.180543 |
| Glycerophospholipid metabolism | ko00564 | 129 | 19 | 0.8362022 | 5 | 0.972535 | 14 | 0.413357 |
| Glycine | ko00260 | 175 | 34 | 0.2898593 | 10 | 0.86699 | 24 | 0.067505 |
| Glycolysis / Gluconeogenesis | ko00010 | 311 | 49 | 0.8272478 | 17 | 0.946283 | 32 | 0.457103 |
| Glycosaminoglycan degradation | ko00531 | 34 | 3 | 0.953412 | 2 | 0.743201 | 1 | 0.972234 |
| Glycosphingolipid biosynthesis - ganglio series | ko00604 | 16 | 1 | 0.9549794 | 1 | 0.718712 |  |  |
| Glycosphingolipid biosynthesis - globo series | ko00603 | 19 | 5 | 0.2325476 | 3 | 0.172148 | 2 | 0.579289 |
| Glycosylphosphatidylinositol(GPI)-anchor biosynthesis | ko00563 | 24 | 3 | 0.8207682 | 3 | 0.274721 |  |  |
| Glyoxylate and dicarboxylate metabolism | ko00630 | 156 | 31 | 0.2557742 | 10 | 0.75926 | 21 | 0.096376 |
| Histidine metabolism | ko00340 | 56 | 7 | 0.8852745 | 1 | 0.988303 | 6 | 0.492559 |
| Indole alkaloid biosynthesis | ko00901 | 4 | 1 | 0.5391384 |  |  | 1 | 0.343601 |
| Inositol phosphate metabolism | ko00562 | 82 | 14 | 0.5959316 | 6 | 0.60117 | 8 | 0.582502 |
| Isoquinoline alkaloid biosynthesis | ko00950 | 64 | 18 | 0.0247175 | 12 | 0.002881 | 6 | 0.626757 |
| Limonene and pinene degradation | ko00903 | 69 | 19 | 0.0265599 | 5 | 0.611997 | 14 | 0.007428 |
| Linoleic acid metabolism | ko00591 | 36 | 14 | 0.0020651 | 9 | 0.001189 | 5 | 0.288208 |
| Lipoic acid metabolism | ko00785 | 13 | 3 | 0.4079785 | 2 | 0.260214 | 1 | 0.745598 |
| Lysine biosynthesis | ko00300 | 30 | 4 | 0.799788 |  |  | 4 | 0.351695 |
| Lysine degradation | ko00310 | 85 | 10 | 0.9471715 | 2 | 0.990664 | 8 | 0.62415 |
| Metabolism of xenobiotics by cytochrome P450 | ko00980 | 76 | 18 | 0.1089725 | 8 | 0.220188 | 10 | 0.224493 |
| Methane metabolism | ko00680 | 178 | 33 | 0.4009984 | 14 | 0.4907 | 19 | 0.41561 |
| Naphthalene degradation | ko00626 | 21 | 2 | 0.906184 | 1 | 0.810841 | 1 | 0.890531 |
| N-Glycan biosynthesis | ko00510 | 82 | 3 | 0.9999795 | 1 | 0.99853 | 2 | 0.998244 |
| Nicotinate and nicotinamide metabolism | ko00760 | 43 | 7 | 0.6524925 | 3 | 0.646258 | 4 | 0.634732 |
| Nitrogen metabolism | ko00910 | 79 | 12 | 0.7573604 | 7 | 0.396315 | 5 | 0.906788 |
| Novobiocin biosynthesis | ko00401 | 11 | 2 | 0.6019876 | 1 | 0.581797 | 1 | 0.685927 |
| One carbon pool by folate | ko00670 | 49 | 8 | 0.6511983 | 1 | 0.979574 | 7 | 0.213438 |
| Other glycan degradation | ko00511 | 51 | 5 | 0.9595283 | 2 | 0.908957 | 3 | 0.896183 |
| Oxidative phosphorylation | ko00190 | 427 | 90 | 0.0335109 | 43 | 0.03523 | 47 | 0.258896 |
| Pantothenate and CoA biosynthesis | ko00770 | 70 | 6 | 0.989858 | 2 | 0.973895 | 4 | 0.929027 |
| Pentose and glucuronate interconversions | ko00040 | 138 | 29 | 0.1711354 | 7 | 0.909261 | 22 | 0.018199 |
| Pentose phosphate pathway | ko00030 | 135 | 21 | 0.7678875 | 11 | 0.453623 | 10 | 0.878556 |
| Phenylalanine | ko00400 | 90 | 11 | 0.9370048 | 4 | 0.919621 | 7 | 8.08E-01 |
| Phenylalanine metabolism | ko00360 | 160 | 34 | 0.1331254 | 16 | 0.159006 | 18 | 3.33E-01 |
| Phenylpropanoid biosynthesis | ko00940 | 227 | 55 | 0.0066346 | 36 | 1.86E-05 | 19 | 0.824166 |
| Photosynthesis | ko00195 | 51 | 21 | 6.574E-05 | 18 | 1.55E-08 | 3 | 8.96E-01 |
| Photosynthesis - antenna proteins | ko00196 | 15 | 6 | 0.0349483 | 4 | 0.023134 | 2 | 0.450435 |
| Polycyclic aromatic hydrocarbon degradation | ko00624 | 42 | 14 | 0.0101714 | 4 | 0.398902 | 10 | 0.00713 |
| Porphyrin and chlorophyll metabolism | ko00860 | 90 | 17 | 0.4164717 | 12 | 0.039674 | 5 | 0.9538 |
| Propanoate metabolism | ko00640 | 194 | 18 | 0.999672 | 9 | 0.964734 | 9 | 0.998047 |
| Purine metabolism | ko00230 | 321 | 43 | 0.9839859 | 11 | 0.999505 | 32 | 0.532995 |
| Pyrimidine metabolism | ko00240 | 230 | 31 | 0.9634583 | 9 | 0.993 | 22 | 0.618617 |
| Pyruvate metabolism | ko00620 | 272 | 30 | 0.9991141 | 7 | 0.999916 | 23 | 0.830295 |
| Retinol metabolism | ko00830 | 50 | 10 | 0.3833218 | 5 | 0.331933 | 5 | 0.568123 |
| Riboflavin metabolism | ko00740 | 21 | 2 | 0.906184 | 1 | 0.810841 | 1 | 0.890531 |
| Selenocompound metabolism | ko00450 | 50 | 3 | 0.9958461 | 2 | 0.902983 | 1 | 0.994881 |
| Sesquiterpenoid and triterpenoid biosynthesis | ko00909 | 28 | 12 | 0.0016016 | 8 | 0.000859 | 4 | 0.304572 |
| Sphingolipid metabolism | ko00600 | 65 | 6 | 0.9810523 | 1 | 0.994291 | 5 | 0.79095 |
| Starch and sucrose metabolism | ko00500 | 318 | 64 | 0.1310692 | 32 | 0.063119 | 32 | 0.510399 |
| Steroid biosynthesis | ko00100 | 52 | 10 | 0.4345307 | 4 | 0.566699 | 6 | 0.419741 |
| Steroid hormone biosynthesis | ko00140 | 28 | 6 | 0.3697172 | 2 | 0.64035 | 4 | 0.304572 |
| Stilbenoid | ko00945 | 65 | 20 | 0.0065953 | 9 | 0.056711 | 11 | 0.055716 |
| Streptomycin biosynthesis | ko00521 | 20 | 1 | 0.9792795 | 1 | 0.795215 |  |  |
| Styrene degradation | ko00643 | 23 | 4 | 0.5960045 |  |  | 4 | 0.191983 |
| Sulfur metabolism | ko00920 | 79 | 8 | 0.9777826 | 4 | 0.861663 | 4 | 0.962322 |
| Synthesis and degradation of ketone bodies | ko00072 | 18 | 2 | 0.851756 |  |  | 2 | 0.549245 |
| Taurine and hypotaurine metabolism | ko00430 | 14 | 4 | 0.2224234 | 1 | 0.670348 | 3 | 0.157841 |
| Terpenoid backbone biosynthesis | ko00900 | 90 | 25 | 0.0108575 | 13 | 0.018297 | 12 | 0.184539 |
| Tetracycline biosynthesis | ko00253 | 18 | 1 | 0.9694561 | 1 | 0.759989 |  |  |
| Thiamine metabolism | ko00730 | 30 | 4 | 0.799788 | 3 | 0.402918 | 1 | 0.957644 |
| Toluene degradation | ko00623 | 4 | 1 | 0.5391384 |  |  | 1 | 0.343601 |
| Tropane | ko00960 | 58 | 13 | 0.2101504 | 5 | 0.455418 | 8 | 0.218828 |
| Tryptophan metabolism | ko00380 | 115 | 25 | 0.147655 | 8 | 0.656858 | 17 | 0.064139 |
| Tyrosine metabolism | ko00350 | 117 | 22 | 0.4032511 | 13 | 0.107855 | 9 | 0.838741 |
| Ubiquinone and other terpenoid-quinone biosynthesis | ko00130 | 77 | 15 | 0.3771477 | 10 | 0.066216 | 5 | 0.89472 |
| Valine | ko00280 | 227 | 29 | 0.9815089 | 9 | 0.99193 | 20 | 0.756805 |
| Valine | ko00290 | 62 | 5 | 0.9901907 | 1 | 0.992748 | 4 | 0.879192 |
| Vitamin B6 metabolism | ko00750 | 30 | 5 | 0.6283488 | 3 | 0.402918 | 2 | 0.816194 |
| Zeatin biosynthesis | ko00908 | 27 | 4 | 0.7250951 | 3 | 0.339011 | 1 | 0.941867 |
| **>2. Genetic Information Processing** |  |  |  |  |  |  |  |  |
| Aminoacyl-tRNA biosynthesis | ko00970 | 164 | 5 | 1 | 3 | 0.999778 | 2 | 1 |
| Basal transcription factors | ko03022 | 47 | 4 | 0.9758512 | 1 | 0.976049 | 3 | 0.861709 |
| Base excision repair | ko03410 | 69 | 6 | 0.9884859 | 1 | 0.99585 | 5 | 0.832163 |
| DNA replication | ko03030 | 79 | 6 | 0.9968833 | 2 | 0.985864 | 4 | 0.962322 |
| Fanconi anemia pathway | ko03460 | 68 | 7 | 0.9672288 | 1 | 0.995506 | 6 | 0.685545 |
| Homologous recombination | ko03440 | 61 | 7 | 0.9303703 | 2 | 0.952403 | 5 | 0.742239 |
| Mismatch repair | ko03430 | 65 | 4 | 0.9982119 |  |  | 4 | 0.900662 |
| mRNA surveillance pathway | ko03015 | 234 | 22 | 0.9998798 | 6 | 0.999776 | 16 | 0.964507 |
| Non-homologous end-joining | ko03450 | 9 | 2 | 0.4884261 | 1 | 0.509931 | 1 | 0.612277 |
| Nucleotide excision repair | ko03420 | 98 | 10 | 0.9856437 | 2 | 0.996251 | 8 | 0.775422 |
| Proteasome | ko03050 | 162 | 27 | 0.6559349 |  |  | 27 | 0.005309 |
| Protein export | ko03060 | 91 | 14 | 0.7520685 | 6 | 7.01E-01 | 8 | 0.700202 |
| Protein processing in endoplasmic reticulum | ko04141 | 486 | 61 | 0.9992491 | 22 | 0.998123 | 39 | 0.94374 |
| Ribosome | ko03010 | 1010 | 235 | 8.711E-07 | 86 | 0.14219 | 149 | 2.84E-07 |
| Ribosome biogenesis in eukaryotes | ko03008 | 122 | 10 | 0.9990956 | 3 | 9.96E-01 | 7 | 0.96655 |
| RNA degradation | ko03018 | 183 | 23 | 0.9753873 | 9 | 0.944656 | 14 | 0.886018 |
| RNA polymerase | ko03020 | 69 | 12 | 0.5682367 | 3 | 0.905048 | 9 | 0.248598 |
| RNA transport | ko03013 | 338 | 38 | 0.9996119 | 11 | 0.999795 | 27 | 0.91319 |
| SNARE interactions in vesicular transport | ko04130 | 37 | 6 | 0.6547707 | 2 | 0.784468 | 4 | 0.512835 |
| Spliceosome | ko03040 | 442 | 40 | 0.9999999 | 20 | 0.997175 | 20 | 0.999995 |
| Ubiquitin mediated proteolysis | ko04120 | 203 | 29 | 0.913889 | 7 | 0.995836 | 22 | 0.375642 |
| **>3. Environmental Information Processing** |  |  |  |  |  |  |  |  |
| ABC transporters | ko02010 | 121 | 17 | 0.8776256 | 7 | 0.824392 | 10 | 0.780439 |
| Bacterial secretion system | ko03070 | 26 | 5 | 0.4930215 | 4 | 0.131603 | 1 | 0.935398 |
| Calcium signaling pathway | ko04020 | 101 | 28 | 0.0075231 | 8 | 0.507682 | 20 | 0.002095 |
| cGMP - PKG signaling pathway | ko04022 | 134 | 33 | 0.0243282 | 12 | 0.322293 | 21 | 0.02463 |
| FoxO signaling pathway | ko04068 | 134 | 22 | 0.6765146 | 12 | 0.322293 | 10 | 0.872977 |
| Hedgehog signaling pathway | ko04340 | 27 | 3 | 0.8780855 | 3 | 0.339011 |  |  |
| HIF-1 signaling pathway | ko04066 | 127 | 20 | 0.7440383 | 7 | 0.859667 | 13 | 0.506379 |
| Hippo signaling pathway | ko04390 | 86 | 8 | 0.9899806 | 3 | 0.964318 | 5 | 0.940009 |
| Hippo signaling pathway -fly | ko04391 | 70 | 5 | 0.9967408 | 1 | 0.996168 | 4 | 0.929027 |
| Jak-STAT signaling pathway | ko04630 | 5 | 1 | 0.6203001 |  |  | 1 | 0.40919 |
| MAPK signaling pathway | ko04010 | 138 | 20 | 0.8607829 | 13 | 0.251638 | 7 | 0.987862 |
| mTOR signaling pathway | ko04150 | 81 | 11 | 0.86654 | 5 | 0.748798 | 6 | 0.832191 |
| Neuroactive ligand-receptor interaction | ko04080 | 11 | 1 | 0.8812948 | 1 | 0.581797 |  |  |
| NF-kappa B signaling pathway | ko04064 | 311 | 86 | 5.447E-06 | 68 | 6.36E-16 | 18 | 0.997304 |
| Notch signaling pathway | ko04330 | 22 | 3 | 0.7708705 | 1 | 0.825277 | 2 | 0.660444 |
| Phosphatidylinositol signaling system | ko04070 | 87 | 22 | 0.0447392 | 7 | 0.497401 | 15 | 2.46E-02 |
| PI3K-Akt signaling pathway | ko04151 | 252 | 24 | 0.9999105 | 8 | 0.999135 | 16 | 0.984838 |
| Plant hormone signal transduction | ko04075 | 253 | 79 | 6.493E-08 | 33 | 0.00159 | 46 | 4.02E-05 |
| Rap1 signaling pathway | ko04015 | 72 | 22 | 0.004949 | 5 | 0.649907 | 17 | 0.000577 |
| Ras signaling pathway | ko04014 | 98 | 24 | 0.0521997 | 8 | 0.472951 | 16 | 0.032889 |
| TGF-beta signaling pathway | ko04350 | 62 | 4 | 0.9971916 |  |  | 4 | 0.879192 |
| Two-component system | ko02020 | 61 | 8 | 0.8647776 | 2 | 0.952403 | 6 | 0.57876 |
| VEGF signaling pathway | ko04370 | 43 | 6 | 0.7929923 | 2 | 0.849835 | 4 | 0.634732 |
| Wnt signaling pathway | ko04310 | 98 | 13 | 0.9010605 | 3 | 0.982779 | 10 | 0.521105 |
| **>4. Cellular Processes** |  |  |  |  |  |  |  |  |
| Adherens junction | ko04520 | 42 | 5 | 0.8846041 | 1 | 0.964346 | 4 | 0.615835 |
| Apoptosis | ko04210 | 318 | 87 | 7.624E-06 | 69 | 5.83E-16 | 18 | 0.998156 |
| Cell cycle | ko04110 | 187 | 25 | 0.952623 | 10 | 0.912347 | 15 | 0.848951 |
| Cell cycle - Caulobacter | ko04112 | 26 | 1 | 0.9935344 |  |  | 1 | 0.935398 |
| Cell cycle - yeast | ko04111 | 125 | 16 | 0.9427379 | 6 | 0.922 | 10 | 0.813069 |
| Endocytosis | ko04144 | 237 | 30 | 0.9854251 | 19 | 0.442463 | 11 | 0.999239 |
| Focal adhesion | ko04510 | 64 | 6 | 0.9785928 | 3 | 0.87525 | 3 | 0.961281 |
| Gap junction | ko04540 | 74 | 19 | 0.0516372 | 4 | 0.82519 | 15 | 0.005752 |
| Lysosome | ko04142 | 203 | 35 | 0.5837733 | 7 | 0.995836 | 28 | 0.048516 |
| Meiosis - yeast | ko04113 | 124 | 18 | 0.8483521 | 10 | 0.473387 | 8 | 0.93723 |
| Oocyte meiosis | ko04114 | 196 | 34 | 0.5678498 | 11 | 0.88978 | 23 | 0.236128 |
| p53 signaling pathway | ko04115 | 58 | 12 | 0.3173553 | 4 | 0.653961 | 8 | 0.218828 |
| Peroxisome | ko04146 | 183 | 30 | 0.6973608 | 9 | 0.944656 | 21 | 0.282739 |
| Phagosome | ko04145 | 213 | 45 | 0.1032626 | 9 | 0.984596 | 36 | 0.001103 |
| Regulation of actin cytoskeleton | ko04810 | 123 | 23 | 0.4106955 | 8 | 0.728942 | 15 | 0.244527 |
| Regulation of autophagy | ko04140 | 60 | 8 | 0.8523418 | 4 | 0.680284 | 4 | 0.862733 |
| Tight junction | ko04530 | 47 | 2 | 0.9987895 |  |  | 2 | 0.956122 |
| **>5. Organismal Systems** |  |  |  |  |  |  |  |  |
| Adipocytokine signaling pathway | ko04920 | 54 | 9 | 0.6276484 | 5 | 0.393932 | 4 | 0.80151 |
| Adrenergic signaling in cardiomyocytes | ko04261 | 111 | 21 | 0.3954051 | 6 | 0.858619 | 15 | 0.139555 |
| Aldosterone-regulated sodium reabsorption | ko04960 | 15 | 2 | 0.7698917 |  |  | 2 | 0.450435 |
| Antigen processing and presentation | ko04612 | 203 | 38 | 0.364889 | 16 | 0.481152 | 22 | 0.375642 |
| Axon guidance | ko04360 | 58 | 11 | 0.4455523 | 4 | 0.653961 | 7 | 0.358433 |
| B cell receptor signaling pathway | ko04662 | 48 | 6 | 0.8719524 | 2 | 0.889924 | 4 | 0.71988 |
| Bile secretion | ko04976 | 90 | 19 | 0.226032 | 6 | 0.691146 | 13 | 0.110896 |
| Carbohydrate digestion and absorption | ko04973 | 18 | 3 | 0.6377214 |  |  | 3 | 0.265539 |
| Cardiac muscle contraction | ko04260 | 91 | 27 | 0.0031437 | 12 | 0.042675 | 15 | 0.035293 |
| Chemokine signaling pathway | ko04062 | 47 | 4 | 0.9758512 | 1 | 0.976049 | 3 | 0.861709 |
| Cholinergic synapse | ko04725 | 27 | 3 | 0.8780855 |  |  | 3 | 0.514803 |
| Circadian entrainment | ko04713 | 49 | 16 | 0.0077582 | 4 | 0.518802 | 12 | 0.002595 |
| Circadian rhythm | ko04710 | 47 | 8 | 0.6017733 | 5 | 0.286044 | 3 | 0.861709 |
| Circadian rhythm - fly | ko04711 | 13 | 1 | 0.9194485 | 1 | 0.643136 |  |  |
| Circadian rhythm - plant | ko04712 | 55 | 23 | 2.214E-05 | 13 | 0.000189 | 10 | 0.043671 |
| Collecting duct acid secretion | ko04966 | 52 | 11 | 0.3013448 |  |  | 11 | 0.01226 |
| Cytosolic DNA-sensing pathway | ko04623 | 26 | 5 | 0.4930215 |  |  | 5 | 0.11115 |
| Dopaminergic synapse | ko04728 | 117 | 20 | 0.5961161 | 6 | 0.889723 | 14 | 0.277608 |
| Endocrine and other factor-regulated calcium reabsorption | ko04961 | 63 | 7 | 0.943515 | 1 | 0.993304 | 6 | 0.61111 |
| Estrogen signaling pathway | ko04915 | 185 | 35 | 0.3468873 | 17 | 0.243418 | 18 | 0.583777 |
| Fat digestion and absorption | ko04975 | 12 | 3 | 0.3560139 | 2 | 0.23108 | 1 | 0.717331 |
| Fc epsilon RI signaling pathway | ko04664 | 32 | 3 | 0.9381995 | 1 | 0.921041 | 2 | 0.843515 |
| Fc gamma R-mediated phagocytosis | ko04666 | 117 | 17 | 0.8416139 | 7 | 0.797246 | 10 | 0.743921 |
| GABAergic synapse | ko04727 | 66 | 11 | 0.6298689 | 4 | 0.750663 | 7 | 0.493074 |
| Gastric acid secretion | ko04971 | 46 | 17 | 0.0013901 | 4 | 0.468557 | 13 | 0.000402 |
| Glutamatergic synapse | ko04724 | 52 | 9 | 0.5795052 | 3 | 0.768262 | 6 | 0.419741 |
| GnRH signaling pathway | ko04912 | 54 | 18 | 0.0038244 | 6 | 0.226427 | 12 | 0.00606 |
| Inflammatory mediator regulation of TRP channels | ko04750 | 66 | 21 | 0.0035181 | 9 | 0.061444 | 12 | 0.02878 |
| Insulin secretion | ko04911 | 13 | 3 | 0.4079785 |  |  | 3 | 0.133415 |
| Insulin signaling pathway | ko04910 | 196 | 38 | 0.2807595 | 17 | 0.323565 | 21 | 0.400811 |
| Leukocyte transendothelial migration | ko04670 | 24 | 3 | 0.8207682 | 1 | 0.85093 | 2 | 0.707231 |
| Long-term depression | ko04730 | 30 | 1 | 0.9970267 |  |  | 1 | 0.957644 |
| Long-term potentiation | ko04720 | 77 | 22 | 0.0115977 | 7 | 0.370777 | 15 | 0.008391 |
| Melanogenesis | ko04916 | 52 | 16 | 0.0142584 | 4 | 0.566699 | 12 | 0.00439 |
| Mineral absorption | ko04978 | 38 | 9 | 0.2142805 | 3 | 0.561232 | 6 | 0.173719 |
| Natural killer cell mediated cytotoxicity | ko04650 | 43 | 6 | 0.7929923 | 2 | 0.849835 | 4 | 0.634732 |
| Neurotrophin signaling pathway | ko04722 | 405 | 103 | 3.512E-05 | 72 | 5.32E-12 | 31 | 0.957915 |
| NOD-like receptor signaling pathway | ko04621 | 54 | 6 | 0.931849 | 3 | 0.790158 | 3 | 0.916745 |
| Olfactory transduction | ko04740 | 38 | 15 | 0.0012078 | 4 | 0.327869 | 11 | 0.000891 |
| Osteoclast differentiation | ko04380 | 45 | 8 | 0.5490475 | 3 | 0.676817 | 5 | 0.471978 |
| Ovarian Steroidogenesis | ko04913 | 29 | 8 | 0.1234954 | 5 | 0.065358 | 3 | 0.564486 |
| Oxytocin signaling pathway | ko04921 | 130 | 32 | 0.0264442 | 13 | 0.189915 | 19 | 0.057819 |
| Pancreatic secretion | ko04972 | 70 | 12 | 0.5897306 | 4 | 0.790567 | 8 | 0.399802 |
| Phototransduction | ko04744 | 43 | 16 | 0.0017542 | 4 | 0.416524 | 12 | 0.000756 |
| Phototransduction - fly | ko04745 | 36 | 15 | 0.0006172 | 4 | 0.292537 | 11 | 0.000536 |
| Plant-pathogen interaction | ko04626 | 361 | 90 | 0.0002283 | 57 | 8.1E-08 | 33 | 0.73316 |
| Platelet activation | ko04611 | 23 | 3 | 0.7971004 | 2 | 0.531905 | 1 | 0.911349 |
| PPAR signaling pathway | ko03320 | 89 | 17 | 0.3971909 | 3 | 0.970183 | 14 | 0.057412 |
| Progesterone-mediated oocyte maturation | ko04914 | 97 | 10 | 0.984042 | 4 | 0.944094 | 6 | 0.93154 |
| Prolactin signaling pathway | ko04917 | 17 | 2 | 0.8279744 |  |  | 2 | 0.517722 |
| Protein digestion and absorption | ko04974 | 27 | 2 | 0.9638656 |  |  | 2 | 0.767204 |
| Proximal tubule bicarbonate reclamation | ko04964 | 27 | 7 | 0.1845715 | 1 | 0.882532 | 6 | 0.046602 |
| Retrograde endocannabinoid signaling | ko04723 | 26 | 6 | 0.3021483 | 3 | 0.317537 | 3 | 0.488853 |
| Salivary secretion | ko04970 | 50 | 18 | 0.0014423 | 5 | 0.331933 | 13 | 0.000962 |
| Serotonergic synapse | ko04726 | 27 | 4 | 0.7250951 | 3 | 0.339011 | 1 | 0.941867 |
| Synaptic vesicle cycle | ko04721 | 118 | 18 | 0.7841127 | 3 | 0.995145 | 15 | 0.197375 |
| T cell receptor signaling pathway | ko04660 | 27 | 3 | 0.8780855 | 1 | 0.882532 | 2 | 0.767204 |
| Taste transduction | ko04742 | 8 | 1 | 0.7876775 |  |  | 1 | 0.569215 |
| Thyroid hormone signaling pathway | ko04919 | 61 | 9 | 0.7699551 | 2 | 0.952403 | 7 | 0.409263 |
| Thyroid hormone synthesis | ko04918 | 69 | 10 | 0.7965916 | 3 | 0.905048 | 7 | 0.541625 |
| Toll-like receptor signaling pathway | ko04620 | 319 | 84 | 5E-05 | 67 | 8.7E-15 | 17 | 0.999177 |
| Vascular smooth muscle contraction | ko04270 | 62 | 19 | 0.0083684 | 7 | 0.190663 | 12 | 0.018228 |
| Vasopressin-regulated water reabsorption | ko04962 | 50 | 11 | 0.2559743 | 3 | 0.744539 | 8 | 0.121015 |
| **>6. Human Diseases** |  |  |  |  |  |  |  |  |
| African trypanosomiasis | ko05143 | 5 | 2 | 0.2144917 |  |  | 2 | 0.081247 |
| Alcoholism | ko05034 | 112 | 30 | 0.0097488 | 7 | 0.758992 | 23 | 0.000595 |
| Alzheimer's disease | ko05010 | 369 | 75 | 0.0936333 | 30 | 0.38075 | 45 | 0.090799 |
| Amoebiasis | ko05146 | 38 | 12 | 0.0258298 | 1 | 0.950992 | 11 | 0.000891 |
| Amphetamine addiction | ko05031 | 66 | 21 | 0.0035181 | 7 | 0.23531 | 14 | 0.004927 |
| Amyotrophic lateral sclerosis (ALS) | ko05014 | 71 | 15 | 0.259187 | 4 | 0.799704 | 11 | 0.093063 |
| Bacterial invasion of epithelial cells | ko05100 | 64 | 4 | 0.9979202 | 1 | 0.993817 | 3 | 0.961281 |
| Basal cell carcinoma | ko05217 | 15 | 2 | 0.7698917 | 2 | 0.318392 |  |  |
| Chagas disease (American trypanosomiasis) | ko05142 | 331 | 87 | 3.966E-05 | 68 | 1.72E-14 | 19 | 0.998149 |
| Chemical carcinogenesis | ko05204 | 75 | 17 | 0.15753 | 8 | 0.20989 | 9 | 0.332597 |
| Chronic myeloid leukemia | ko05220 | 18 | 1 | 0.9694561 | 1 | 0.759989 |  |  |
| Colorectal cancer | ko05210 | 55 | 3 | 0.9981285 | 1 | 0.987333 | 2 | 0.978468 |
| Epithelial cell signaling in Helicobacter pylori infection | ko05120 | 82 | 15 | 0.4800506 | 1 | 0.99853 | 14 | 0.031516 |
| Epstein-Barr virus infection | ko05169 | 364 | 49 | 0.9875756 | 14 | 0.999082 | 35 | 0.621976 |
| Glioma | ko05214 | 53 | 15 | 0.0362935 | 4 | 0.582068 | 11 | 0.014116 |
| Hepatitis B | ko05161 | 68 | 6 | 0.9869402 | 1 | 0.995506 | 5 | 0.822532 |
| Hepatitis C | ko05160 | 49 | 1 | 0.9999261 |  |  | 1 | 0.99431 |
| Herpes simplex infection | ko05168 | 138 | 22 | 0.7303691 | 5 | 0.98293 | 17 | 0.214283 |
| HTLV-I infection | ko05166 | 172 | 32 | 0.3950276 | 15 | 0.330329 | 17 | 0.556369 |
| Huntington's disease | ko05016 | 408 | 71 | 0.5646643 | 27 | 0.806546 | 44 | 0.31592 |
| Hypertrophic cardiomyopathy (HCM) | ko05410 | 23 | 4 | 0.5960045 | 3 | 0.253527 | 1 | 0.911349 |
| Influenza A | ko05164 | 421 | 100 | 0.0006724 | 77 | 1.99E-13 | 23 | 0.999764 |
| Legionellosis | ko05134 | 216 | 34 | 0.7912633 | 18 | 0.380332 | 16 | 0.923182 |
| Leishmaniasis | ko05140 | 293 | 82 | 5.46E-06 | 66 | 3.63E-16 | 16 | 0.9983 |
| Measles | ko05162 | 432 | 102 | 0.0007429 | 76 | 2.25E-12 | 26 | 0.998959 |
| MicroRNAs in cancer | ko05206 | 101 | 13 | 0.9218928 | 4 | 0.954814 | 9 | 0.690383 |
| Morphine addiction | ko05032 | 17 | 4 | 0.3502209 | 2 | 0.375423 | 2 | 0.517722 |
| Nicotine addiction | ko05033 | 9 | 3 | 0.200638 | 2 | 0.146033 | 1 | 0.612277 |
| Non-alcoholic fatty liver disease (NAFLD) | ko04932 | 271 | 42 | 0.8422752 | 22 | 0.409161 | 20 | 0.944947 |
| Pancreatic cancer | ko05212 | 36 | 3 | 0.9650651 | 1 | 0.942545 | 2 | 0.887375 |
| Parkinson's disease | ko05012 | 358 | 71 | 0.1456937 | 38 | 0.022664 | 33 | 0.715556 |
| Pathogenic Escherichia coli infection | ko05130 | 89 | 19 | 0.2109525 | 4 | 0.91543 | 15 | 0.029575 |
| Pathways in cancer | ko05200 | 176 | 14 | 0.9999328 | 6 | 0.993752 | 8 | 0.99753 |
| Pertussis | ko05133 | 340 | 101 | 1.78E-08 | 72 | 5.12E-16 | 29 | 0.842644 |
| Primary immunodeficiency | ko05340 | 5 | 1 | 0.6203001 |  |  | 1 | 0.40919 |
| Prion diseases | ko05020 | 41 | 8 | 0.4365258 | 1 | 0.961394 | 7 | 0.109252 |
| Prostate cancer | ko05215 | 93 | 8 | 0.995667 | 2 | 0.994665 | 6 | 0.913385 |
| Proteoglycans in cancer | ko05205 | 115 | 16 | 0.8810234 | 9 | 0.516671 | 7 | 0.949215 |
| Renal cell carcinoma | ko05211 | 56 | 4 | 0.9932039 | 1 | 0.988303 | 3 | 0.928314 |
| Rheumatoid arthritis | ko05323 | 68 | 15 | 0.2060189 | 2 | 0.970132 | 13 | 0.01583 |
| Salmonella infection | ko05132 | 59 | 7 | 0.9146181 | 1 | 0.990789 | 6 | 0.54511 |
| Shigellosis | ko05131 | 57 | 8 | 0.8095055 | 1 | 0.989198 | 7 | 0.341584 |
| Small cell lung cancer | ko05222 | 36 | 2 | 0.9919167 |  |  | 2 | 0.887375 |
| Systemic lupus erythematosus | ko05322 | 35 | 11 | 0.0332678 |  |  | 11 | 0.000408 |
| Toxoplasmosis | ko05145 | 426 | 104 | 0.0001815 | 81 | 4.67E-15 | 23 | 0.999822 |
| Transcriptional misregulation in cancers | ko05202 | 60 | 13 | 0.2485108 | 6 | 0.305615 | 7 | 0.392305 |
| Tuberculosis | ko05152 | 451 | 117 | 3.782E-06 | 73 | 3.51E-10 | 44 | 0.590946 |
| Type I diabetes mellitus | ko04940 | 30 | 6 | 0.4377405 | 3 | 0.402918 | 3 | 0.588129 |
| Type II diabetes mellitus | ko04930 | 31 | 5 | 0.6585505 |  |  | 5 | 0.19229 |
| Vibrio cholerae infection | ko05110 | 102 | 20 | 0.3349441 | 1 | 0.999703 | 19 | 0.005417 |
| Viral carcinogenesis | ko05203 | 215 | 33 | 0.8332779 | 8 | 0.994061 | 25 | 0.238638 |
| Viral myocarditis | ko05416 | 40 | 4 | 0.9391156 | 1 | 0.958198 | 3 | 0.777045 |
