# Supplementary material for: Transcriptome analysis and identification of key genes involved in 1-deoxynojirimycin biosynthesis of mulberry (Morus alba L.)
Source: PeerJ. 2018 Aug 23;6:e5443. doi: 10.7717/peerj.5443 (PMC6109587; doi:10.7717/peerj.5443)
Supplement: Supplemental Information 9 [file peerj-06-5443-s009.doc]

**Table S6 The FPKM of transcripts involve in DNJ biosynthesis pathway**

| **Transcripts** | **M7 FPKM** | **M11 FPKM** | **P Value** | **Mark** |
| --- | --- | --- | --- | --- |
| LysC | aspartate kinase (EC:2.7.2.4) | | |  |
| c19834_g1 | 22.43 | 13.19 | 0.23769311 |  |
| c14545_g1 | 0.1 | 0 | 0.10421207 |  |
| c50019_g1 | 32.58 | 32.88 | 0.66972593 |  |
| c97209_g1 | 0 | 0.5 |  |  |
| c8863_g1 | 11.68 | 4.3 | 1.16E-05 | Down |
| c69203_g1 | 0.34 | 0.08 | 0.1892919 |  |
| c46556_g1 | 50.27 | 64.54 | 1.49E-06 |  |
| c14803_g1 | 0.05 | 0.09 | 0.62861019 |  |
| c95128_g1 | 28.47 | 25.2 | 0.56544223 |  |
| c40311_g1 | 33.54 | 14.41 | 4.24E-64 | Down |
| ASD | aspartate-semialdehyde dehydrogenase (EC:1.2.1.11) | | |  |
| c79123_g1 | 1.21 | 0 | 0.49179438 |  |
| c39238_g1 | 42.22 | 31.82 | 2.17E-15 |  |
| DapA | 4-hydroxy-tetrahydrodipicolinate synthase (EC:4.3.3.7) | | |  |
| c868_g1 | 36.61 | 32.64 | 0.00042313 |  |
| c73282_g1 | 0.15 | 0.33 | 0.27079286 |  |
| c79871_g1 | 0.16 | 0.09 | 0.76842432 |  |
| c43595_g1 | 1.39 | 0 | 2.24E-10 | Down |
| DapB | 4-hydroxy-tetrahydrodipicolinate reductase (EC:1.17.1.8) | | |  |
| c45119_g1 | 23.5 | 22.74 | 0.16672937 |  |
| ALD/AGD | LL-diaminopimelate aminotransferase (EC:2.6.1.83) | | |  |
| c44849_g1 | 12.31 | 2.47 | 2.28E-49 | Down |
| c50115_g1 | 64.31 | 92.65 | 1.89E-11 |  |
| c107446_g1 | 0.75 | 0 | 0.49179438 |  |
| DapF | diaminopimelate epimerase (EC:5.1.1.7) | | |  |
| c87345_g1 | 0.12 | 0 |  |  |
| c83928_g1 | 0.18 | 0 | 0.49179438 |  |
| c29316_g1 | 51.21 | 38.21 | 5.20E-20 |  |
| Lys A | diaminopimelate decarboxylase (EC:4.1.1.20) | | |  |
| c106552_g1 | 0.38 | 0.31 | 0.95743763 |  |
| c35146_g1 | 75.26 | 47.12 | 4.50E-78 |  |
| LdcC/cadA | lysine decarboxylase (EC:4.1.1.18) | | |  |
| c83396_g1 | 11.73 | 5.01 | 1.88E-07 | Down |
| AOC2/3 | primary-amine oxidase (EC:1.4.3.21) | | |  |
| c47185_g1 | 48.21 | 12.94 | 0 | Down |
| c50870_g1 | 3.43 | 3 | 0.17463812 |  |
| c36513_g1 | 12.64 | 17.03 | 3.10E-07 |  |
| c39997_g1 | 1.44 | 0.69 | 0.02087522 |  |
| c56783_g1 | 0.25 | 0 | 0.49179438 |  |
| c40850_g1 | 18.57 | 16.58 | 0.03364688 |  |
| c72626_g1 | 0 | 0 |  |  |
| c103084_g1 | 0.32 | 0 |  |  |
| c48882_g1 | 10.71 | 3.06 | 4.47E-61 | Down |
| c47618_g1 | 32.06 | 252.8 | 7.32E-11 | Up |
| c32423_g1 | 1.87 | 0.57 | 1.92E-05 | Down |
| c51868_g1 | 5.43 | 5.41 | 0.86294967 |  |
