# Supplementary material for: Transcriptome analysis and identification of key genes involved in 1-deoxynojirimycin biosynthesis of mulberry (Morus alba L.)
Source: PeerJ. 2018 Aug 23;6:e5443. doi: 10.7717/peerj.5443 (PMC6109587; doi:10.7717/peerj.5443)
Supplement: Supplemental Information 10 [file peerj-06-5443-s010.doc]

**Table S7 CYP450 related gene statistics in *Morus alba* L. leaves** transcriptome data

| **Gene_ID** | **KO** | **KO_name** | **KO_description** | **EC** | **M7 FPKM** | **M11 FPKM** | **M7 vs M11** |
| --- | --- | --- | --- | --- | --- | --- | --- |
| c35900_g1 | K07437 | CYP26A | cytochrome P450, family 26, subfamily A | - | 0.88 | 0.23 | - |
| c51154_g3 | K00512 | CYP17A | steroid 17alpha-monooxygenase / 17alpha-hydroxyprogesterone aldolase | - | 46.46 | 33.4 | - |
| c83202_g1 | K10717 | CYP735A | cytokinin trans-hydroxylase | - | 0.38 | 0 | - |
| c491_g1 | K13407 | CYP94A1 | fatty acid omega-hydroxylase | EC:1.14.-.- | 4.88 | 2.61 | - |
| c52682_g1 | K17961 | CYP82G1 | cytochrome P450, family 82, subfamily G, polypeptide 1 | EC:1.14.-.- | 37.83 | 82.16 | Up |
| c50289_g1 | K09755 | CYP84A, F5H | ferulate-5-hydroxylase | EC:1.14.-.- | 0.6 | 7.65 | Up |
| c47881_g1 | K15639 | CYP734A1, AS1 | PHYB activation tagged suppressor 1 | EC:1.14.-.- | 1.95 | 0 | Down |
| c84190_g1 | K07408 | CYP1A1 | cytochrome P450, family 1, subfamily A, polypeptide 1 | EC:1.14.14.1 | 0.25 | 0 | - |
| c87450_g1 | K09754 | CYP98A3, C3'H | coumaroylquinate(coumaroylshikimate) 3'-monooxygenase | EC:1.14.13.36 | 0.25 | 0 | - |
| c52287_g1 | K07408 | CYP1A1 | cytochrome P450, family 1, subfamily A, polypeptide 1 | EC:1.14.14.1 | 9.54 | 52.04 | Up |
| c48201_g1 | K15747 | LUT5, CYP97A3 | beta-ring hydroxylase | EC:1.14.-.- | 25.71 | 40.33 | - |
| c71902_g1 | K17961 | CYP82G1 | cytochrome P450, family 82, subfamily G, polypeptide 1 | EC:1.14.-.- | 0 | 0 | - |
| c10574_g1 | K15747 | LUT5, CYP97A3 | beta-ring hydroxylase | EC:1.14.-.- | 0.52 | 0 | - |
| c39749_g1 | K09587 | CYP90B1,DWF4 | steroid 22-alpha-hydroxylase | EC:1.14.13.- | 7.22 | 11.34 | - |
| c76713_g1 | K09755 | CYP84A, F5H | ferulate-5-hydroxylase | EC:1.14.-.- | 0.67 | 0 | - |
| c44921_g1 | K07418 | CYP2J | cytochrome P450, family 2, subfamily J | EC:1.14.14.1 | 1.49 | 8.13 | Up |
| c28142_g1 | K07418 | CYP2J | cytochrome P450, family 2, subfamily J | EC:1.14.14.1 | 0 | 0.91 | - |
| c30282_g1 | K15747 | LUT5, CYP97A3 | beta-ring hydroxylase | EC:1.14.-.- | 0.46 | 0 | - |
| c16252_g1 | K15402 | CYP86B1 | fatty acid omega-hydroxylase | EC:1.14.-.- | 0.64 | 0.53 | - |
| c82835_g1 | K04122 | GA3, CYP701 | ent-kaurene oxidase | EC:1.14.13.78 | 6.23 | 12.14 | - |
| c4186_g1 | K09832 | CYP710A | cytochrome P450, family 710, subfamily A | - | 6.66 | 3.54 | - |
| c41596_g2 | K12153 | CYP79A2 | phenylalanine N-monooxygenase | EC:1.14.13.124 | 0.02 | 1.05 | Up |
| c4543_g1 | K15639 | CYP734A1 | PHYB activation tagged suppressor 1 | EC:1.14.-.- | 2.64 | 1.86 | - |
| c63904_g1 | K07408 | CYP1A1 | cytochrome P450, family 1, subfamily A, polypeptide 1 | EC:1.14.14.1 | 0 | 0.3 | - |
| c54745_g1 | K12156 | CYP83A1 | cytochrome P450, family 83, subfamily A, polypeptide 1 | EC:1.14.-.- | 0.77 | 0 | - |
| c75438_g1 | K05917 | CYP51 | sterol 14-demethylase | EC:1.14.13.70 | 0.24 | 0 | - |
| c32774_g1 | K07408 | CYP1A1 | cytochrome P450, family 1, subfamily A, polypeptide 1 | EC:1.14.14.1 | 3.35 | 0.08 | - |
| c100671_g1 | K15402 | CYP86B1 | fatty acid omega-hydroxylase | EC:1.14.-.- | 0 | 0 | - |
| c39525_g1 | K12639 | CYP724B1, D11 | cytochrome P450, family 724, subfamily B, polypeptide 1 | EC:1.14.13.- | 4.94 | 2.01 | Down |
| c50094_g1 | K15398 | CYP86A4S | fatty acid omega-hydroxylase | EC:1.14.-.- | 54.42 | 34.96 | - |
| c51919_g2 | K05917 | CYP51 | sterol 14-demethylase | EC:1.14.13.70 | 108.36 | 60.56 | - |
| c51428_g3 | K00487 | CYP73A | trans-cinnamate 4-monooxygenase | EC:1.14.13.11 | 0.03 | 0.35 | - |
| c530_g1 | K15639 | CYP734A1,BAS1 | PHYB activation tagged suppressor 1 | EC:1.14.-.- | 0.54 | 0 | - |
| c40867_g1 | K15639 | CYP734A1,BAS1 | PHYB activation tagged suppressor 1 | EC:1.14.-.- | 8.37 | 6.44 | - |
| c47420_g2 | K15639 | CYP734A1,BAS1 | PHYB activation tagged suppressor 1 | EC:1.14.-.- | 3.37 | 1.86 | - |
| c87710_g1 | K12639 | CYP724B1, D11 | cytochrome P450, family 724, subfamily B, polypeptide 1 | EC:1.14.13.- | 0.29 | 0 | - |
| c21934_g1 | K07408 | CYP1A1 | cytochrome P450, family 1, subfamily A, polypeptide 1 | EC:1.14.14.1 | 1.72 | 0.81 | - |
| c29603_g1 | K07408 | CYP1A1 | cytochrome P450, family 1, subfamily A, polypeptide 1 | EC:1.14.14.1 | 34.71 | 15.85 | Down |
| c59387_g1 | K09837 | LUT1, CYP97C1 | carotene epsilon-monooxygenase | EC:1.14.99.45 | 0.64 | 0 | - |
| c25481_g1 | K15639 | CYP734A1,BAS1 | PHYB activation tagged suppressor 1 | EC:1.14.-.- | 0.12 | 0.71 | - |
| c51006_g1 | K12638 | CYP90D1 | 3-epi-6-deoxocathasterone 23-monooxygenase | EC:1.14.13.112 | 7.54 | 10.03 | - |
| c47981_g2 | K12637 | CYP90C1, ROT3 | 3-epi-6-deoxocathasterone 23-monooxygenase | EC:1.14.13.112 | 2.95 | 6.01 | Up |
| c49343_g1 | K07408 | CYP1A1 | cytochrome P450, family 1, subfamily A, polypeptide 1 | EC:1.14.14.1 | 6.95 | 7 | - |
| c31168_g1 | K09754 | CYP98A3, C3'H | coumaroylquinate(coumaroylshikimate) 3'-monooxygenase | EC:1.14.13.36 | 156.27 | 192.75 | - |
| c37278_g2 | K00512 | CYP17A | steroid 17alpha-monooxygenase / 17alpha-hydroxyprogesterone aldolase | [EC:1.14.99.9 4.1.2.30] | 19.77 | 23.46 | - |
| c61878_g1 | K07408 | CYP1A1 | cytochrome P450, family 1, subfamily A, polypeptide 1 | EC:1.14.14.1 | 0.09 | 0.43 | - |
| c109597_g1 | K15639 | CYP734A1,BAS1 | PHYB activation tagged suppressor 1 | EC:1.14.-.- | 0.4 | 0.09 | - |
| c83737_g1 | K15402 | CYP86B1 | fatty acid omega-hydroxylase | EC:1.14.-.- | 0.65 | 0 | - |
| c105913_g1 | K15639 | CYP734A1,BAS1 | PHYB activation tagged suppressor 1 | EC:1.14.-.- | 0.42 | 0 | - |
| c46601_g1 | K10717 | CYP735A | cytokinin trans-hydroxylase | - | 1.07 | 0 | Down |
| c83751_g1 | K07437 | CYP26A | cytochrome P450, family 26, subfamily A | - | 0.43 | 0.17 | - |
| c26819_g2 | K17961 | CYP82G1 | cytochrome P450, family 82, subfamily G, polypeptide 1 | EC:1.14.-.- | 0 | 0.75 | - |
| c52376_g2 | K07408 | CYP1A1 | cytochrome P450, family 1, subfamily A, polypeptide 1 | EC:1.14.14.1 | 134.28 | 89.42 | - |
| c33960_g1 | K00512 | CYP17A | steroid 17alpha-monooxygenase / 17alpha-hydroxyprogesterone aldolase | [EC:1.14.99.9 4.1.2.30] | 6.5 | 1.43 | Down |
| c103522_g1 | K00487 | CYP73A | trans-cinnamate 4-monooxygenase | EC:1.14.13.11 | 114.77 | 154.41 | - |
| c107064_g1 | K07418 | CYP2J | cytochrome P450, family 2, subfamily J | EC:1.14.14.1 | 0.94 | 0 | - |
| c101524_g1 | K12640 | CYP85A2, BR6OX2 | brassinosteroid-6-oxidase 2 | EC:1.14.-.- | 0 | 0 | - |
| c68074_g1 | K05917 | CYP51 | sterol 14-demethylase | EC:1.14.13.70 | 0.22 | 0 | - |
| c83163_g1 | K09754 | CYP98A3, C3'H | coumaroylquinate(coumaroylshikimate) 3'-monooxygenase | EC:1.14.13.36 | 0.47 | 0 | - |
| c85697_g1 | K10717 | CYP735A | cytokinin trans-hydroxylase | - | 0.36 | 0 | - |
| c47159_g1 | K07418 | CYP2J | cytochrome P450, family 2, subfamily J | EC:1.14.14.1 | 0.35 | 0.92 | Up |
| c47901_g1 | K07418 | CYP2J | cytochrome P450, family 2, subfamily J | EC:1.14.14.1 | 0.51 | 3.23 | Up |
| c72777_g1 | K09754 | CYP98A3, C3'H | coumaroylquinate(coumaroylshikimate) 3'-monooxygenase | EC:1.14.13.36 | 0.31 | 0 | - |
| c51106_g7 | K12640 | CYP85A2, R6OX2 | brassinosteroid-6-oxidase 2 | EC:1.14.-.- | 73.11 | 34.43 | Down |
| c76981_g1 | K15747 | LUT5, CYP97A3 | beta-ring hydroxylase | EC:1.14.-.- | 0.53 | 0 | - |
| c4116_g1 | K15639 | CYP734A1, BAS1 | PHYB activation tagged suppressor 1 | EC:1.14.-.- | 0.75 | 0 | - |
| c346_g1 | K00487 | CYP73A | trans-cinnamate 4-monooxygenase | EC:1.14.13.11 | 0.08 | 0.81 | - |
| c100363_g1 | K07418 | CYP2J | cytochrome P450, family 2, subfamily J | EC:1.14.14.1 | 0.67 | 0 | - |
| c93311_g1 | K15398 | CYP86A4S | fatty acid omega-hydroxylase | EC:1.14.-.- | 22.78 | 7.05 | Down |
| c42019_g1 | K09588 | CYP90A1, CPD | cytochrome P450, family 90, subfamily A, polypeptide 1 | EC:1.14.-.- | 73.18 | 28.44 | Down |
| c39327_g1 | K07437 | CYP26A | cytochrome P450, family 26, subfamily A | - | 1.07 | 2.23 | Up |
| c87733_g1 | K09755 | CYP84A, F5H | ferulate-5-hydroxylase | EC:1.14.-.- | 0.3 | 0 | - |
| c43910_g1 | K09837 | LUT1, CYP97C1 | carotene epsilon-monooxygenase | EC:1.14.99.45 | 14.71 | 27.22 | - |
| c104225_g1 | K15401 | CYP86A1 | fatty acid omega-hydroxylase | EC:1.14.-.- | 0.17 | 0.78 | Up |
| c14751_g1 | K00487 | CYP73A | trans-cinnamate 4-monooxygenase | EC:1.14.13.11 | 0.4 | 0 | - |
| c105322_g1 | K00512 | CYP17A | steroid 17alpha-monooxygenase / 17alpha- hydroxyprogesterone aldolase [EC:1.14.99.9 4.1.2.30] | - | 3.61 | 0.68 | Down |
| c47780_g1 | K07408 | CYP1A1 | cytochrome P450, family 1, subfamily A, polypeptide 1 | EC:1.14.14.1 | 1.89 | 6.72 | Up |
| c26780_g1 | K09837 | LUT1, CYP97C1 | carotene epsilon-monooxygenase | EC:1.14.99.45 | 0.34 | 0 | - |
| c98867_g1 | K07408 | CYP1A1 | cytochrome P450, family 1, subfamily A, polypeptide 1 | EC:1.14.14.1 | 0 | 0 | - |
| c9924_g1 | K10717 | CYP735A | cytokinin trans-hydroxylase | - | 0.38 | 0 | - |
| c94262_g1 | K15639 | CYP734A1,BAS1 | PHYB activation tagged suppressor 1 | EC:1.14.-.- | 1.22 | 0 | - |
| c85902_g1 | K15402 | CYP86B1 | fatty acid omega-hydroxylase | EC:1.14.-.- | 0.03 | 0 | - |
| c68044_g1 | K10717 | CYP735A | cytokinin trans-hydroxylase | - | 0.45 | 0 | - |
| c35949_g1 | K00487 | CYP73A | trans-cinnamate 4-monooxygenase | EC:1.14.13.11 | 87.3 | 130.67 | - |
| c59619_g1 | K07418 | CYP2J | cytochrome P450, family 2, subfamily J | EC:1.14.14.1 | 0 | 2.89 | - |
| c89692_g1 | K15402 | CYP86B1 | fatty acid omega-hydroxylase | EC:1.14.-.- | 0.18 | 0.6 | - |
| c109686_g1 | K15402 | CYP86B1 | fatty acid omega-hydroxylase | EC:1.14.-.- | 0.22 | 0.56 | - |
| c74253_g1 | K09754 | CYP98A3, C3'H | coumaroylquinate(coumaroylshikimate) 3'-monooxygenase | EC:1.14.13.36 | 0 | 1.08 | - |
| c83161_g1 | K09754 | CYP98A3, C3'H | coumaroylquinate(coumaroylshikimate) 3'-monooxygenase | EC:1.14.13.36 | 1.28 | 0 | - |
| c94735_g1 | K07418 | CYP2J | cytochrome P450, family 2, subfamily J | EC:1.14.14.1 | 0.51 | 0 | - |
| c26819_g1 | K17961 | CYP82G1 | cytochrome P450, family 82, subfamily G, polypeptide 1 | EC:1.14.-.- | 0.24 | 0.81 | - |
| c72293_g1 | K07408 | CYP1A1 | cytochrome P450, family 1, subfamily A, polypeptide 1 | EC:1.14.14.1 | 0.16 | 0 | - |
| c28343_g1 | K07408 | CYP1A1 | cytochrome P450, family 1, subfamily A, polypeptide 1 | EC:1.14.14.1 | 0.69 | 0.94 | - |
| c100747_g1 | K12639 | CYP724B1, D11 | cytochrome P450, family 724, subfamily B, polypeptide 1 | EC:1.14.13.- | 0.94 | 0 | - |
| c25385_g1 | K09567 | PPIH, CYPH | peptidyl-prolyl isomerase H (cyclophilin H) | EC:5.2.1.8 | 106.07 | 88.91 | - |
| c62392_g1 | K09754 | CYP98A3, C3'H | coumaroylquinate(coumaroylshikimate) 3'-monooxygenase | EC:1.14.13.36 | 0 | 1.06 | - |
| c17108_g1 | K00487 | CYP73A | trans-cinnamate 4-monooxygenase | EC:1.14.13.11 | 154.85 | 147.16 | - |
| c56694_g1 | K07418 | CYP2J | cytochrome P450, family 2, subfamily J | EC:1.14.14.1 | 0 | 0 | - |
| c47159_g2 | K07418 | CYP2J | cytochrome P450, family 2, subfamily J | EC:1.14.14.1 | 0.8 | 1.57 | - |
| c49345_g2 | K07418 | CYP2J | cytochrome P450, family 2, subfamily J | EC:1.14.14.1 | 18.86 | 22.41 | - |
| c79937_g1 | K10717 | CYP735A | cytokinin trans-hydroxylase | - | 0.65 | 0 | - |
| c52376_g1 | K07418 | CYP2J | cytochrome P450, family 2, subfamily J | EC:1.14.14.1 | 0.93 | 3.42 | - |
| c19275_g1 | K10717 | CYP735A | cytokinin trans-hydroxylase | - | 0.12 | 0.42 | - |
