# Supplementary material for: Transcriptome analysis and identification of key genes involved in 1-deoxynojirimycin biosynthesis of mulberry (Morus alba L.)
Source: PeerJ. 2018 Aug 23;6:e5443. doi: 10.7717/peerj.5443 (PMC6109587; doi:10.7717/peerj.5443)
Supplement: Supplemental Information 11 [file peerj-06-5443-s011.doc]

**Table S8 Methyltransferase related gene statistics in *Morus alba* L.** transcriptome data

| **Gene_ID** | **KO** | **KO_name** | **KO_ description** | **EC** | **M7 FPKM** | **M11 FPKM** | **M7 vs M11** |
| --- | --- | --- | --- | --- | --- | --- | --- |
| c96286_g1 | K00549 | metE | 5-methyltetrahydropteroyltriglutamate--homocysteine methyltransferase | EC:2.1.1.14 | 0 | 0.52 | - |
| c92695_g1 | K00548 | metH, MTR | 5-methyltetrahydrofolate--homocysteine methyltransferase | EC:2.1.1.13 | 1.18 | 0 | - |
| c40930_g1 | K05929 | NMT | phosphoethanolamine N-methyltransferase | EC:2.1.1.103 | 25.56 | 11.85 | Down |
| c52381_g4 | K00591 | COQ3 | hexaprenyldihydroxybenzoate methyltransferase | EC:2.1.1.114 | 289.03 | 144.78 | - |
| c31636_g1 | K00600 | glyA, SHMT | glycine hydroxymethyltransferase | EC:2.1.2.1 | 37.68 | 45.4 |  |
| c73942_g1 | K00549 | metE | 5-methyltetrahydropteroyltriglutamate--homocysteine methyltransferase | EC:2.1.1.14 | 0.17 | 0.43 | - |
| c41333_g1 | K00559 | SMT1, ERG6 | sterol 24-C-methyltransferase | EC:2.1.1.41 | 106.67 | 47.68 | Down |
| c59715_g1 | K00600 | glyA, SHMT | glycine hydroxymethyltransferase | EC:2.1.2.1 | 1.06 | 0 | - |
| c74027_g1 | K13066 | E2.1.1.68, COMT | caffeic acid 3-O-methyltransferase | EC:2.1.1.68 | 0.29 | 0.24 | - |
| c47072_g1 | K00558 | DNMT1, dcm | DNA (cytosine-5)-methyltransferase 1 | EC:2.1.1.37 | 76.15 | 82.52 | - |
| c99899_g1 | K14563 | NOP1, FBL | rRNA 2'-O-methyltransferase fibrillarin | EC:2.1.1.- |  |  |  |
| c41514_g1 | K08241 | E2.1.1.141 | jasmonate O-methyltransferase | EC:2.1.1.141 | 0.31 | 0.2 | - |
| c98363_g1 | K00600 | glyA, SHMT | glycine hydroxymethyltransferase | EC:2.1.2.1 |  |  |  |
| c61297_g1 | K12502 | VTE3, APG1 | MPBQ/MSBQ methyltransferase | - | 69.7 | 175.93 | Up |
| c50367_g1 | K11433 | SETMAR | histone-lysine N-methyltransferase SETMAR | EC:2.1.1.43 | 19.22 | 9.76 |  |
| c100948_g1 | K00605 | gcvT, AMT | aminomethyltransferase | EC:2.1.2.10 | 3.07 | 0 | - |
| c39743_g2 | K13066 | COMT | caffeic acid 3-O-methyltransferase | EC:2.1.1.68 | 7.02 | 7.04 | - |
| c43782_g1 | K05929 | NMT | phosphoethanolamine N-methyltransferase | EC:2.1.1.103 | 29.8 | 15.77 | - |
| c55130_g1 | K00605 | gcvT, AMT | aminomethyltransferase | EC:2.1.2.10 | 0.21 | 0 | - |
| c52752_g1 | K11420 | EHMT | euchromatic histone-lysine N-methyltransferase | EC:2.1.1.43 | 9.29 | 9.38 | - |
| c85612_g1 | K00547 | mmuM | homocysteine S-methyltransferase | EC:2.1.1.10 | 0.95 | 0 | - |
| c67720_g1 | K11420 | EHMT | euchromatic histone-lysine N-methyltransferase | EC:2.1.1.43 | 0.71 | 0 | - |
| c34733_g1 | K00600 | glyA, SHMT | glycine hydroxymethyltransferase | EC:2.1.2.1 | 12.76 | 21.61 | - |
| c1035_g1 | K00547 | mmuM | homocysteine S-methyltransferase | EC:2.1.1.10 | 0.75 | 0 | - |
| c67913_g1 | K11420 | EHMT | euchromatic histone-lysine N-methyltransferase | EC:2.1.1.43 | 0.27 | 0 | - |
| c48154_g1 | K11420 | EHMT | euchromatic histone-lysine N-methyltransferase | EC:2.1.1.43 | 22.77 | 16.99 | - |
| c46782_g1 | K02516 | PRMT5, HSL7 | protein arginine N-methyltransferase 5 | EC:2.1.1.125 | 18.42 | 27.47 | - |
| c109012_g1 | K00600 | glyA, SHMT | glycine hydroxymethyltransferase | EC:2.1.2.1 |  |  | - |
| c84258_g1 | K00549 | metE | 5-methyltetrahydropteroyltriglutamate--homocysteine methyltransferase | EC:2.1.1.14 |  |  | - |
| c98726_g1 | K00591 | COQ3 | hexaprenyldihydroxybenzoate methyltransferase | EC:2.1.1.114 |  |  | - |
| c87086_g1 | K00548 | metH, MTR | 5-methyltetrahydrofolate--homocysteine methyltransferase | EC:2.1.1.13 | 0.13 | 0.07 | - |
| c67873_g1 | K14563 | NOP1, FBL | rRNA 2'-O-methyltransferase fibrillarin | EC:2.1.1.- | 0.26 | 0 | - |
| c112630_g1 | K00549 | metE | 5-methyltetrahydropteroyltriglutamate--homocysteine methyltransferase | EC:2.1.1.14 |  |  | - |
| c35141_g1 | K00547 | mmuM | homocysteine S-methyltransferase | EC:2.1.1.10 | 0.39 | 0.37 | - |
| c109512_g1 | K00558 | DNMT1, dcm | DNA (cytosine-5)-methyltransferase 1 | EC:2.1.1.37 |  |  | - |
| c46898_g1 | K00605 | gcvT, AMT | aminomethyltransferase | EC:2.1.2.10 | 115.67 | 196.99 | - |
| c16508_g1 | K08241 | E2.1.1.141 | jasmonate O-methyltransferase | EC:2.1.1.141 | 61.79 | 215.6 | Up |
| c67135_g1 | K00558 | DNMT1, dcm | DNA (cytosine-5)-methyltransferase 1 | EC:2.1.1.37 | 0.08 | 0.13 | - |
| c45319_g1 | K13066 | COMT | caffeic acid 3-O-methyltransferase | EC:2.1.1.68 | 7.13 | 5.57 | - |
| c9389_g1 | K14563 | NOP1, FBL | rRNA 2'-O-methyltransferase fibrillarin | EC:2.1.1.- | 209.18 | 137.38 | - |
| c49564_g1 | K00547 | mmuM | homocysteine S-methyltransferase | EC:2.1.1.10 | 6.25 | 8.86 | - |
| c42239_g1 | K00600 | glyA, SHMT | glycine hydroxymethyltransferase | EC:2.1.2.1 | 21.47 | 24.65 | - |
| c48946_g1 | K11430 | EZH2 | histone-lysine N-methyltransferase EZH2 | EC:2.1.1.43 | 11.88 | 8.83 | - |
| c103972_g1 | K14568 | EMG1, NEP1 | rRNA small subunit pseudouridine methyltransferase Nep1 | EC:2.1.1.260 | 32.75 | 20.63 | - |
| c82266_g1 | K00605 | gcvT, AMT | aminomethyltransferase | EC:2.1.2.10 | 0.58 | 0 | - |
| c330_g1 | K13066 | E2.1.1.68, COMT | caffeic acid 3-O-methyltransferase | EC:2.1.1.68 | 0 | 2.3 | Up |
| c14995_g1 | K00600 | glyA, SHMT | glycine hydroxymethyltransferase | EC:2.1.2.1 | 14.4 | 3.29 | Down |
| c50526_g2 | K00549 | metE | 5-methyltetrahydropteroyltriglutamate--homocysteine methyltransferase | EC:2.1.1.14 | 422.99 | 520.23 | - |
| c50458_g2 | K11434 | PRMT1 | protein arginine N-methyltransferase 1 | EC:2.1.1.- | 70.98 | 48.74 | - |
| c52472_g1 | K00558 | DNMT1, dcm | DNA (cytosine-5)-methyltransferase 1 | EC:2.1.1.37 | 22.04 | 17.13 | - |
| c41159_g1 | K05928 | E2.1.1.95 | tocopherol O-methyltransferase | EC:2.1.1.95 | 10.81 | 31.68 | Up |
| c85195_g1 | K00600 | glyA, SHMT | glycine hydroxymethyltransferase | EC:2.1.2.1 | 0 | 2.41 | - |
| c50995_g1 | K00587 | ICMT, STE14 | protein-S-isoprenylcysteine O-methyltransferase | EC:2.1.1.100 | 24.43 | 10.44 | Down |
| c15006_g1 | K00600 | glyA, SHMT | glycine hydroxymethyltransferase | EC:2.1.2.1 | 171.04 | 61.27 | Down |
| c1939_g1 | K00600 | glyA, SHMT | glycine hydroxymethyltransferase | EC:2.1.2.1 | 0.25 | 0 | - |
| c52752_g2 | K11420 | EHMT | euchromatic histone-lysine N-methyltransferase | EC:2.1.1.43 | 4.47 | 2.65 | - |
| c66141_g1 | K00549 | metE | 5-methyltetrahydropteroyltriglutamate--homocysteine methyltransferase | EC:2.1.1.14 | 0.7 | 0 | - |
| c51217_g2 | K12502 | VTE3, APG1 | MPBQ/MSBQ methyltransferase | - | 36.05 | 55.45 | - |
| c75607_g1 | K00558 | DNMT1, dcm | DNA (cytosine-5)-methyltransferase 1 | EC:2.1.1.37 |  |  | - |
| c52205_g2 | K00558 | DNMT1, dcm | DNA (cytosine-5)-methyltransferase 1 | EC:2.1.1.37 | 29.43 | 36.53 | - |
| c68624_g1 | K00605 | gcvT, AMT | aminomethyltransferase | EC:2.1.2.10 |  |  | - |
| c83740_g1 | K11434 | PRMT1 | protein arginine N-methyltransferase 1 | EC:2.1.1.- | 0.19 | 0 | - |
| c98122_g1 | K00600 | glyA, SHMT | glycine hydroxymethyltransferase | EC:2.1.2.1 | 0 | 0.77 | - |
| c50000_g1 | K17398 | DNMT3A | DNA (cytosine-5)-methyltransferase 3A | EC:2.1.1.37 | 17.74 | 12.79 | - |
| c9503_g1 | K00547 | mmuM | homocysteine S-methyltransferase | EC:2.1.1.10 | 7.95 | 5.69 | - |
| c41260_g1 | K06127 | COQ5 | ubiquinone biosynthesis methyltransferase | EC:2.1.1.201 | 6.22 | 12.14 | - |
| c44966_g1 | K13066 | COMT | caffeic acid 3-O-methyltransferase | EC:2.1.1.68 | 1.14 | 1.16 | - |
| c56114_g1 | K00548 | metH, MTR | 5-methyltetrahydrofolate--homocysteine methyltransferase | EC:2.1.1.13 | 0.64 | 0 | - |
| c88808_g1 | K00549 | metE | 5-methyltetrahydropteroyltriglutamate--homocysteine methyltransferase | EC:2.1.1.14 | 0 | 1.2 | - |
| c9559_g1 | K11420 | EHMT | euchromatic histone-lysine N-methyltransferase | EC:2.1.1.43 | 13.72 | 5.31 | Down |
| c50526_g3 | K00549 | metE | 5-methyltetrahydropteroyltriglutamate--homocysteine methyltransferase | EC:2.1.1.14 | 235.34 | 313.88 | - |
| c83962_g1 | K00565 | RNMT | mRNA (guanine-N7-)-methyltransferase | EC:2.1.1.56 |  |  | - |
| c44723_g1 | K05929 | E2.1.1.103, NMT | phosphoethanolamine N-methyltransferase | EC:2.1.1.103 | 48.65 | 67.77 | - |
| c110536_g1 | K06127 | COQ5 | ubiquinone biosynthesis methyltransferase | EC:2.1.1.201 |  |  | - |
| c26220_g1 | K08242 | E2.1.1.143 | 24-methylenesterol C-methyltransferase | EC:2.1.1.143 | 126.62 | 95.69 | - |
| c74218_g1 | K08241 | E2.1.1.141 | jasmonate O-methyltransferase | EC:2.1.1.141 | 0.75 | 0 | - |
| c46824_g1 | K00565 | RNMT | mRNA (guanine-N7-)-methyltransferase | EC:2.1.1.56 | 19.31 | 14.86 | - |
| c44887_g1 | K13066 | E2.1.1.68, COMT | caffeic acid 3-O-methyltransferase | EC:2.1.1.68 | 46.46 | 24.78 | - |
| c54124_g1 | K00600 | glyA, SHMT | glycine hydroxymethyltransferase | EC:2.1.2.1 | 0.45 | 0 | - |
| c101370_g1 | K00549 | metE | 5-methyltetrahydropteroyltriglutamate--homocysteine methyltransferase | EC:2.1.1.14 | 0.26 | 0.44 | - |
| c49082_g2 | K08241 | E2.1.1.141 | jasmonate O-methyltransferase | EC:2.1.1.141 | 6.72 | 15.39 | Up |
| c51779_g2 | K11423 | SETD2, SET2 | histone-lysine N-methyltransferase SETD2 | EC:2.1.1.43 | 22.89 | 58.96 | Up |
| c50321_g3 | K00565 | RNMT | mRNA (guanine-N7-)-methyltransferase | EC:2.1.1.56 | 25.41 | 15.25 | - |
| c62156_g1 | K00600 | glyA, SHMT | glycine hydroxymethyltransferase | EC:2.1.2.1 |  |  | - |
| c82743_g1 | K00545 | COMT | catechol O-methyltransferase | EC:2.1.1.6 | 1.07 | 0 | - |
| c100338_g1 | K14563 | NOP1, FBL | rRNA 2'-O-methyltransferase fibrillarin | EC:2.1.1.- | 1.76 | 0 | - |
| c96705_g1 | K08241 | E2.1.1.141 | jasmonate O-methyltransferase | EC:2.1.1.141 | 0.19 | 0 | - |
| c91339_g1 | K00591 | COQ3 | hexaprenyldihydroxybenzoate methyltransferase | EC:2.1.1.114 |  |  | - |
| c53926_g1 | K11434 | PRMT1 | protein arginine N-methyltransferase 1 | EC:2.1.1.- | 0 | 0.76 | - |
| c28557_g1 | K00606 | panB | 3-methyl-2-oxobutanoate hydroxymethyltransferase | EC:2.1.2.11 | 9.18 | 4.41 | Down |
| c42701_g1 | K11434 | PRMT1 | protein arginine N-methyltransferase 1 | EC:2.1.1.- | 48.67 | 43.71 | - |
| c78472_g1 | K11423 | SETD2, SET2 | histone-lysine N-methyltransferase SETD2 | EC:2.1.1.43 | 1.2 | 0 | - |
| c45960_g1 | K00600 | glyA, SHMT | glycine hydroxymethyltransferase | EC:2.1.2.1 | 148.51 | 328.1 | Up |
| c46335_g1 | K11423 | SETD2, SET2 | histone-lysine N-methyltransferase SETD2 | EC:2.1.1.43 | 7.7 | 10.66 | - |
| c95600_g1 | K14563 | NOP1, FBL | rRNA 2'-O-methyltransferase fibrillarin | EC:2.1.1.- |  |  | - |
| c13468_g1 | K00588 | E2.1.1.104 | caffeoyl-CoA O-methyltransferase | EC:2.1.1.104 | 32.92 | 32.25 | - |
| c51711_g1 | K11430 | EZH2 | histone-lysine N-methyltransferase EZH2 | EC:2.1.1.43 | 10.62 | 15.58 | - |
| c110288_g1 | K11434 | PRMT1 | protein arginine N-methyltransferase 1 | EC:2.1.1.- | 0 | 0.69 | - |
| c73114_g1 | K13066 | E2.1.1.68, COMT | caffeic acid 3-O-methyltransferase | EC:2.1.1.68 | 0 | 0.77 | - |
| c53973_g1 | K11434 | PRMT1 | protein arginine N-methyltransferase 1 | EC:2.1.1.- | 2.12 | 0 | - |
| c45319_g2 | K13066 | E2.1.1.68, COMT | caffeic acid 3-O-methyltransferase | EC:2.1.1.68 | 0 | 2.47 | Up |
| c36604_g2 | K11420 | EHMT | euchromatic histone-lysine N-methyltransferase | EC:2.1.1.43 | 24.25 | 21.72 | - |
| c29198_g1 | K00600 | glyA, SHMT | glycine hydroxymethyltransferase | EC:2.1.2.1 | 203.96 | 185.36 | - |
| c85083_g1 | K00606 | panB | 3-methyl-2-oxobutanoate hydroxymethyltransferase | EC:2.1.2.11 | 0.13 | 0.11 | - |
| c16410_g1 | K00547 | mmuM | homocysteine S-methyltransferase | EC:2.1.1.10 | 0.49 | 0 | Down |
| c92463_g1 | K13066 | E2.1.1.68, COMT | caffeic acid 3-O-methyltransferase | EC:2.1.1.68 |  |  | - |
| c46605_g1 | K03428 | E2.1.1.11, chlM, bchM | magnesium-protoporphyrin O-methyltransferase | EC:2.1.1.11 | 64.62 | 95.66 | - |
| c43454_g1 | K00600 | glyA, SHMT | glycine hydroxymethyltransferase | EC:2.1.2.1 | 1.35 | 0 | Down |
| c52226_g2 | K08247 | E2.1.1.12 | methionine S-methyltransferase | EC:2.1.1.12 | 22.83 | 15.2 | - |
| c45458_g1 | K13384 | E2.1.1.140 | (S)-coclaurine N-methyltransferase | EC:2.1.1.140 | 26.1 | 15.75 | - |
