# Supplementary material for: Transcriptome analysis and identification of key genes involved in 1-deoxynojirimycin biosynthesis of mulberry (Morus alba L.)
Source: PeerJ. 2018 Aug 23;6:e5443. doi: 10.7717/peerj.5443 (PMC6109587; doi:10.7717/peerj.5443)
Supplement: Supplemental Information 12 [file peerj-06-5443-s012.doc]

**Table S9** **All differentially expressed transcripts involved in DNJ biosynthesis transcripts**

| **Transcripts** | **M7** | **M11** | **log2FoldChange** | **P Value** | **Mark** |
| --- | --- | --- | --- | --- | --- |
| **LysC** | **aspartate kinase (EC:2.7.2.4)** | | | |  |
| c8863_g1 | 11.68 | 4.3 | -1.439688926 | 1.16E-05 | Down |
| c40311_g1 | 33.54 | 14.41 | -1.202189612 | 4.24E-64 | Down |
| **DapA** | **4-hydroxy-tetrahydrodipicolinate synthase (EC:4.3.3.7)** | | | |  |
| c43595_g1 | 1.39 | 0 |  | 2.24E-10 | Down |
| **ALD/AGD** | **LL-diaminopimelate aminotransferase (EC:2.6.1.83)** | | | |  |
| c44849_g1 | 12.31 | 2.47 | -2.317217462 | 2.28E-49 | Down |
| **LdcC/cadA** | **lysine decarboxylase (EC:1.4.3.21)** | | | |  |
| c83396_g1 | 11.73 | 5.01 | -0.763150444 | 1.88E-07 | Down |
| **AOC2/3** | **primary-amine oxidase (EC:1.4.3.21)** | | | |  |
| c47185_g1 | 48.21 | 12.94 | -1.896855008 | 0 | Down |
| c48882_g1 | 10.71 | 3.06 | -1.807420711 | 4.47E-61 | Down |
| c47618_g1 | 32.06 | 252.8 | 1.931823003 | 7.32E-11 | Up |
| c32423_g1 | 1.87 | 0.57 | -1.734949071 | 1.92E-05 | Down |
