# Supplementary material for: Transcriptome analysis and identification of key genes involved in 1-deoxynojirimycin biosynthesis of mulberry (Morus alba L.)
Source: PeerJ. 2018 Aug 23;6:e5443. doi: 10.7717/peerj.5443 (PMC6109587; doi:10.7717/peerj.5443)
Supplement: Supplemental Information 13 [file peerj-06-5443-s013.doc]

The sequencing reads were submitted to NCBI and we have an e-mail from NCBI to release the raw data currently.

The raw data information of M7 and M11:

STUDY: PRJNA427492 (SRP127713)

SAMPLE: M7 (SRS2797649)

EXPERIMENT: M7 (SRX3520883)

RUN: M7_combined_R2.fastq.gz (SRR6429236)

SAMPLE: Mulberry leaves (M11) (SRS2797648)

EXPERIMENT: M11 (SRX3520882)

RUN: M11_combined_R1.fastq.gz (SRR6429237)

The raw data information of Ma7 and Ma11:

STUDY: PRJNA438058 (SRP135584)

SAMPLE: Ma7 (SRS3040006)

EXPERIMENT: Ma7 (SRX3787207)

RUN: Ma7_combined_R1.fastq.gz (SRR6831124)

SAMPLE: Mulberry leaves (Ma11) (SRS3040007)

EXPERIMENT: M11 (SRX3787206)

RUN: M11_combined_R2.fastq.gz (SRR6831125)
